# A phenotype-based AI pipeline outperforms human experts in differentially diagnosing rare diseases using EHRs

**DOI:** 10.1038/s41746-025-01452-1

**Published:** 2025-01-28

**Authors:** Xiaohao Mao, Yu Huang, Ye Jin, Lun Wang, Xuanzhong Chen, Honghong Liu, Xinglin Yang, Haopeng Xu, Xiaodong Luan, Ying Xiao, Siqin Feng, Jiahao Zhu, Xuegong Zhang, Rui Jiang, Shuyang Zhang, Ting Chen

**Affiliations:** 1 Department of Computer Science and Technology & Institute for Artificial Intelligence & BNRist, Tsinghua University, Beijing, China; 2 Tencent Jarvis Lab, Shenzhen, China; 3 Medical Research Center, State Key Laboratory of Complex Severe and Rare Diseases, Peking Union Medical College Hospital, Chinese Academy of Medical Sciences & Peking Union Medical College, Beijing, China; 4 Department of Internal Medicine, Peking Union Medical College Hospital, Chinese Academy of Medical Sciences & Peking Union Medical College, Beijing, China; 5 Department of Cardiology, Peking Union Medical College Hospital, Chinese Academy of Medical Sciences & Peking Union Medical College, Beijing, China; 6 Nuffield Department of Medicine, University of Oxford, Oxford, United Kingdom; 7 State Key Laboratory of Complex Severe and Rare Diseases, Peking Union Medical College Hospital, Chinese Academy of Medical Sciences & Peking Union Medical College, Beijing, China; 8 Department of Geriatrics, Peking Union Medical College Hospital, Chinese Academy of Medical Sciences & Peking Union Medical College, Beijing, China; 9 Department of Cardiology, State Key Laboratory of Complex Severe and Rare Diseases, Peking Union Medical College Hospital, Chinese Academy of Medical Sciences & Peking Union Medical College, Beijing, China; 10 Department of Automation & BNRist, Tsinghua University, Beijing, China

**Keywords:** Diagnosis, Diseases

## Abstract

Rare diseases, affecting ~350 million people worldwide, pose significant challenges in clinical diagnosis due to the lack of experienced physicians and the complexity of differentiating between numerous rare diseases. To address these challenges, we introduce PhenoBrain, a fully automated artificial intelligence pipeline. PhenoBrain utilizes a BERT-based natural language processing model to extract phenotypes from clinical texts in EHRs and employs five new diagnostic models for differential diagnoses of rare diseases. The AI system was developed and evaluated on diverse, multi-country rare disease datasets, comprising 2271 cases with 431 rare diseases. In 1936 test cases, PhenoBrain achieved an average predicted top-3 recall of 0.513 and a top-10 recall of 0.654, surpassing 13 leading prediction methods. In a human-computer study with 75 cases, PhenoBrain exhibited exceptional performance with a top-3 recall of 0.613 and a top-10 recall of 0.813, surpassing the performance of 50 specialist physicians and large language models like ChatGPT and GPT-4. Combining PhenoBrain’s predictions with specialists increased the top-3 recall to 0.768, demonstrating its potential to enhance diagnostic accuracy in clinical workflows.

## Introduction

Rare disease refers to conditions that affect a small percentage of a population. In the United States, a rare disease is defined as one with a population prevalence of no more than one in 1500; in Japan and the European Union, the prevalence threshold is no more than one in 2500 and 2000, respectively^1^. Despite their low prevalence, rare diseases affect ~350 million people worldwide^2^. Most rare diseases are genetic in origin and can be life-threatening or chronically debilitating, contributing to a high disease burden^3,4^. Recent studies have shown that rare diseases are frequently misdiagnosed or delayed-diagnosed by years; such an uncertain and unpredictable period is often referred to as a diagnostic odyssey^5–7^. The clinical diagnosis rates for rare diseases remain unsatisfactory. One challenge is that physicians may not be able to recognize rare diseases and their associated phenotypes correctly because they have never seen such diseases before. Another challenge is that to make accurate diagnoses, physicians may have to differentiate between a large number of rare diseases (and common diseases), each of which may be characterized by multiple phenotypes frequently shared by many diseases. In addition, a rare disease may exhibit different, partially overlapping combinations of phenotypes in different individuals. Some rare diseases may affect multiple systems or organs and exhibit a wide range of phenotypes, so the diagnosis and treatment would require a multi-disciplinary team (MDT) of physicians. All these factors complicate the diagnosis of rare diseases.

Efforts have been made to improve the diagnosis of rare diseases, including building knowledgebases such as the Online Mendelian Inheritance in Man (OMIM)^8^ and Orphanet^9^, standardizing disease-phenotype terminology in Human phenotype ontology (HPO)^10,11^, building rare disease patient databases such as NRDRS^12^. These efforts enable the development of computational methods for predicting or ranking diseases and disease genes based on phenotype information. Current computational methods can be broadly classified into four categories. The first category includes Phen2Disease^13^, LIRICAL^14^, Xrare^15^, and Phrank^16^, which prioritize candidates for both diseases and genes based on associations between phenotypes, diseases, and genes. Their main differences are in how they calculate the similarity between patient phenotypes and annotated disease-associated phenotypes. The second category includes Phen2Gene^17^, EHR-Phenolyzer^18^, AMELIE2^19^, and ClinPhen^20^, which predict pathogenic genes rather than diseases. The third category prioritizes rare diseases rather than genes based on similarity measures between patient phenotypes and disease phenotypes. Many statistical or machine learning-based methods^21–30^, including Phenomizer^21^, RDAD^28^, RDD^26^, and MinIC^25^, have been developed or applied to take advantage of the hierarchical structure of HPO phenotypes. The fourth category includes using advanced deep learning models, such as large language models (LLM), for rare disease diagnosis^31–33^. However, this category is still in its early stages.

The aforementioned computational methods require direct input of phenotypes obtained from electronic health records. Natural language processing (NLP) methods such as EHR-Phenolyzer^18^, ClinPhen^20^, PhenoTagger^34^, and PhenoBERT^35^ have been developed to map clinical texts into standardized HPO terms. However, clinicians may document patient phenotypes in clinical records in a variety of ways, with varying details and terminologies, posing a great challenge for computational methods to map or infer phenotypes from clinical texts accurately. For some diseases, a specific examination may be required to obtain key phenotypes in order to differentiate or confirm this disease. If a clinician lacks this knowledge, a patient’s phenotype description will be incomplete. Computational methods will struggle to make an accurate diagnosis. Finally, most NLP tools were developed for English EHRs, while similar tools for other languages are mostly lacking.

To address these challenges, we developed a fully automated AI pipeline for differential diagnosis of rare diseases. The main contributions include (1) the development of novel computational models for rare disease prediction, (2) the development of a comprehensive pipeline that involves PBTagger, a Chinese-oriented HPO phenotype automatic extraction framework, and (3) a comprehensive, comparative analysis of rare disease prediction methods, including a human-computer comparison. Moreover, the experimental results, which explored large language models such as GPT-4 and ChatGPT for rare disease diagnosis, underscore the promising potential of integrating these models into the clinical diagnostic process, opening up exciting possibilities for future advancements in this field.

## Results

### Overview of PhenoBrain

**Fig. 1:**
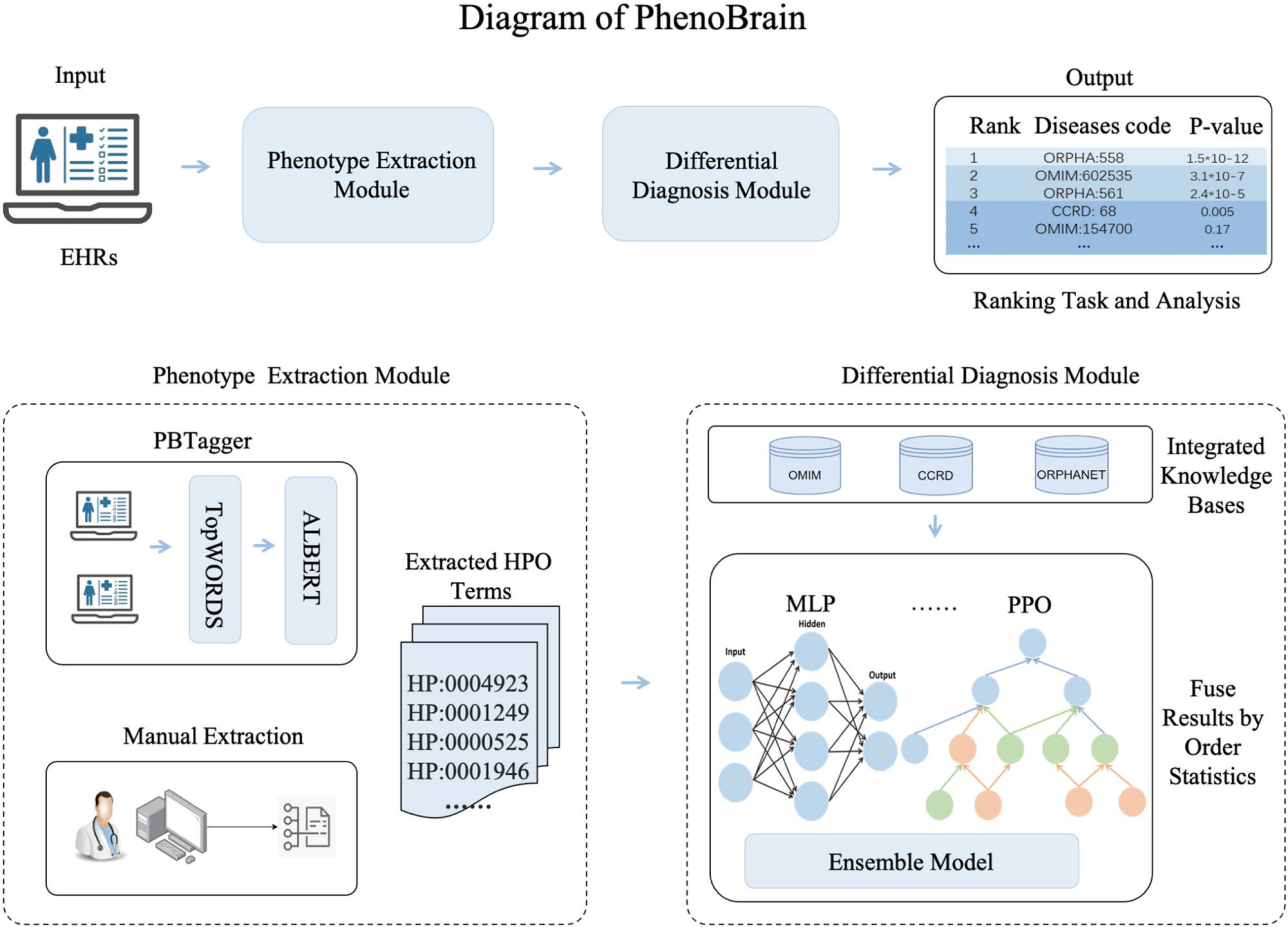
Workflow of PhenoBrain. PhenoBrain takes input from an electronic health record (EHR) and predicts a ranked list of rare diseases by using two modules: “Phenotype extraction module” and “Differential diagnosis module”. In the “Phenotype extraction module”, PhenoBrain extracts medical symptoms from EHRs using TopWords, and then maps these symptoms to HPO terms using an ALBERT NLP model. This module also allows medical texts to be manually annotated with HPO terms. In the “Differential diagnosis module”, the extracted HPO terms are fed into an ensemble learning model using order statistics for differential diagnosis of rare diseases.

PhenoBrain (http://www.phenobrain.cs.tsinghua.edu.cn/pc**)** is comprised of two modules, as shown in Fig. 1. The first was a phenotype extraction module automated by PBTagger, an NLP pipeline based on deep learning (Supplementary Note 2). PBTagger first identified medical terms from EHRs using TopWORDS^36^, an unsupervised word discovery and segmentation method. Then, it applies an HPO linking model that we developed using a twin ALBERT network^37,38^ and Discriminative Deep Metric Learning (DDML)^39^ to map these medical terms into HPO standard phenotypes. The HPO linking model takes in a pair of medical texts and returns a matching score indicating whether they are the same or not. The overall architecture is shown in Supplementary Fig. 1.

The second module of PhenoBrain was a differential diagnosis module that employed extracted HPO phenotypes to predict a ranking of rare diseases, with the most probable disease at the top (Supplementary Note 3). The prediction was based on phenotype annotations of 9260 rare diseases (see Supplementary Table 1) that were created by integrating OMIM, Orphanet, and CCRD. PhenoBrain implemented 17 machine learning methods for differential diagnosis, including 12 benchmark methods from previous works and five new computational methods we developed. These five methods include a semantic similarity measure model (ICTO), a probabilistic graph-based Bayesian model (PPO), two machine learning approaches using few-shot techniques, and an ensemble learning model based on order statistics^40^. It should be noted that most of these 17 methods rely solely on disease-phenotype annotations for model development, with a few models requiring hyperparameter tuning using a small dataset.

### Rare disease datasets

**Fig. 2:**
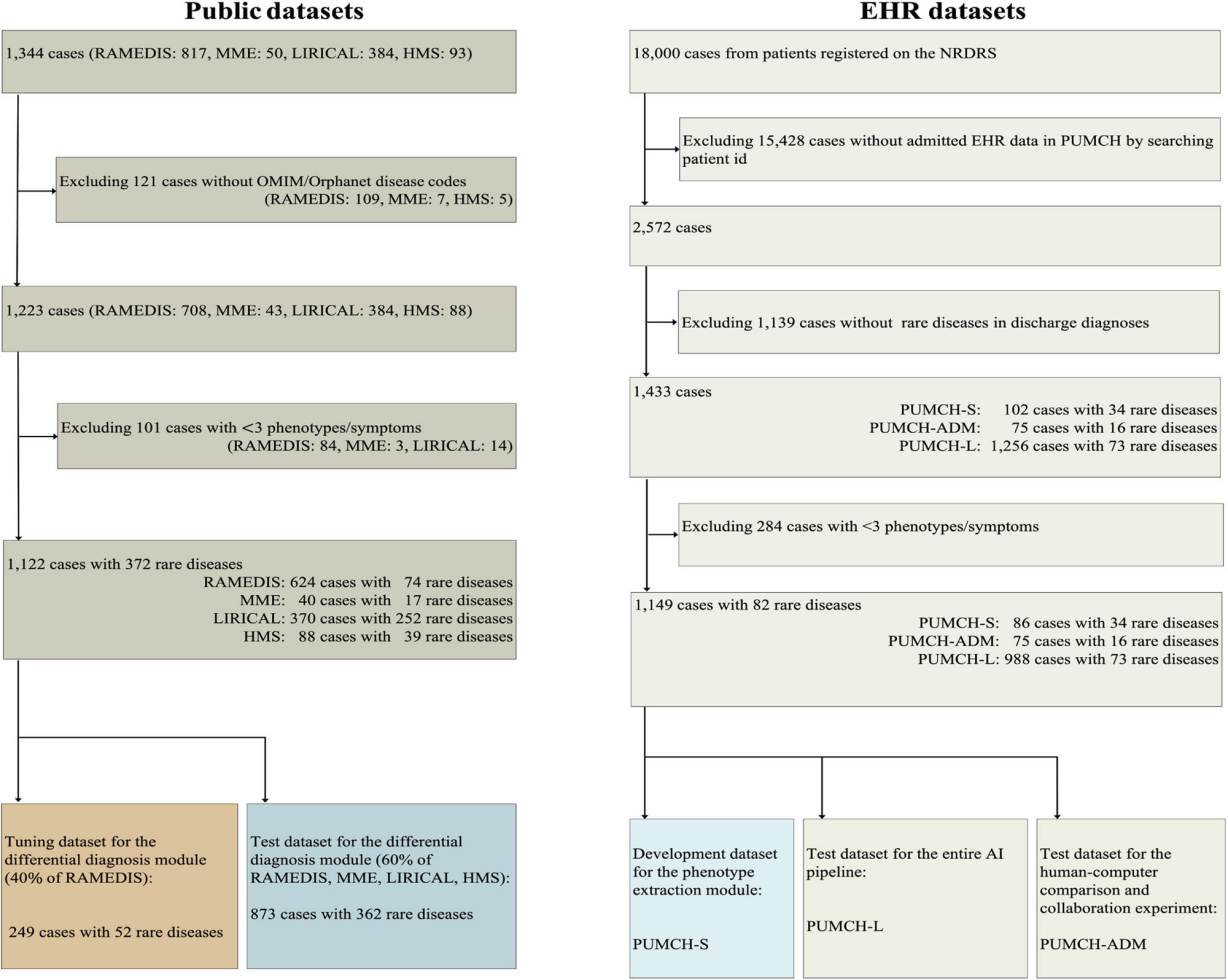
Preprocessing rare disease datasets. The Public datasets were preprocessed for the tuning and testing of the differential diagnosis module: 40% of RAMEDIS cases randomly selected were used to tune hyper-parameters of the differential diagnosis methods, while the remaining cases (60% of RAMEDIS) and the other smaller public datasets (MME, HMS, LIRICAL) serve as the inner and external test datasets, respectively. The EHR datasets were preprocessed for developing and testing the phenotype extraction module, and the whole AI pipeline, PUMCH-S, was used for developing and testing the phenotype extraction module. PUMCH-ADM was used for the Human-Computer comparison and simulated collaboration experiment. PUMCH-L was used to test the whole AI pipeline.

This work divides the dataset into two categories: publicly available datasets and PUMCH/National Rare Diseases Registry System (NRDRS) datasets, as outlined in Table 1 and Fig. 2. For further insight, we have provided an overview of the phenotype information and the top 5 categories of rare diseases in each dataset (see Supplementary Tables 2 and 3).

**Table 1:** Basic characteristics of rare disease test sets

	Public test set				EHR test set	
Subsets	RAMEDIS	MME	LIRICAL	HMS	PUMCH-L^a^	PUMCH-ADM
Countries/regions	European	Canada	Multi-Country	Germany	China	China
Number of cases included	375	40	370	88	988	75
Total	873				1063	
Categories of diseases	63	17	252	39	73	16
Total	362				80	
Age, years: median (average)	12 days (4.7)	NA	9 (14.5)	44 (43.4)	34 (35.4)	29 (31.6)
Female (%)	200 (53.3%)	NA	174 (47.0%)	54 (61.4%)	512 (51.8%)	36 (48%)
Number of cases per disease						
Minimum	1	1	1	1	1	3
Median	2	1	1	1	1	5
Maximum	82	11	19	11	200	8
Number of cases diagnosed						
OMIM	375	40	370	69	723	70
ORPHANET	375	39	227	81	983	75
CCRD	257	1	6	13	529	75
Number of HPO terms per case						
Minimum	3	3	3	5	3	3
Median	9	10.5	11	17.5	31	16
Maximum	46	26	95	54	101	47

^a^In the PUMCH-L dataset, the HPO terms were extracted by PBTagger. In contrast, in all other PUMCH datasets, HPO terms were manually annotated. The PUMCH-ADM dataset was used for the Human-computer experiment.

From 18,000 patient records registered in the NRDRS in China between 1 January 2016, and 13 November 2019, we obtained 2572 EHR data from patients admitted to PUMCH. Among the 2572 admitted records, we selected 1433 cases based on diagnoses that included rare diseases in corresponding discharge records, resulting in the initial PUMCH dataset. The PUMCH dataset contains complete electronic medical records, allowing them to be used for both phenotype extraction and diagnostic tasks. As shown in Fig. 2, the dataset was then partitioned into three subsets for three tasks: (1) PUMCH-S, a subset for hyperparameter tuning (102 cases); (2) PUMCH-ADM, a subset for human-AI comparison (75 cases); and (3) PUMCH-L, a subset for testing the entire AI pipeline (1256 cases). To ensure the validity of our analysis, cases with fewer than three HPO phenotypes were excluded, resulting in 1149 EHR cases with 82 rare diseases(Fig. 2), distributed as 86 cases for PUMCH-S, 75 cases for PUMCH-ADM, and 988 cases for PUMCH-L, respectively. To develop the NLP model and the entire AI pipeline, we randomly selected 40% of the cases from PUMCH-S for training, while the remaining 60% were utilized for testing. PUMCH-L was a separate dataset for testing the NLP model and the entire AI pipeline, and it differed from PUMCH-S in that the phenotypes in PUMCH-S were extracted manually, but not in PUMCH-L. PUMCH-ADM was used to compare the performance of PhenoBrain against that of 50 experienced specialist physicians, as well as ChatGPT and GPT-4. It should be noted that with the exception of PUMCH-L, phenotypes were extracted manually from these datasets. Disease Names and Number of Cases for two PUMCH Datasets are shown in Supplementary Table 4, all the EHR Test sets have been consolidated into an Excel file named Supplementary Dataset 1 and uploaded to the NPJ website (see Supplementary Data).

The public datasets provide only phenotype information, mostly in HPO terms, and confirmed disease diagnoses in OMIM/Orphanet disease codes. Therefore, they can only be used to evaluate diagnostic performance. Initially, we collected 1344 cases with phenotypic information and rare disease diagnoses from public sources or other relevant addresses, including RAMEDIS^41^, MME^41^, HMS^30^, and LIRICAL^14^. RAMEDIS^41^ is a public Rare Metabolic Diseases Database developed collaboratively by scientists from Bielefeld University and the Children’s Hospital of Reutlingen in Germany. MME^42^ is a public dataset compiled collaboratively by scientists from the Children’s Hospital of Eastern Ontario in Canada, SickKids, and the University of Toronto. HMS^30^ is a public dataset collected from the outpatient clinic of Hannover Medical School. LIRICAL^14^ consists of a published case report set collected by scientists from the Jackson laboratory. We have obtained explicit permission from the owners of these datasets to use them in this study. To ensure the validity of our analysis, cases with fewer than three HPO phenotypes or lacking a confirmed diagnosis were excluded. This resulted in 1122 cases with 372 rare diseases that were used to develop and test the performance of diagnostic methods. Since some of the diagnostic models require hyperparameter tuning, we set 40% of the RAMEDIS cases for training and tuning, while the remaining 60% serve as an inner test dataset. The other three smaller public datasets were combined to form an external test dataset. As shown in Table 1, to avoid a complex description of these datasets, we present the experimental results on the entire public test set (Public test set, Table 1) in the Results section. More information can be found in the Method section. All the Public Test sets have been consolidated into a PDF file named Supplementary Dataset 2 and uploaded to the NPJ website (see Supplementary Data).

### Performance evaluation of differential diagnosis module

The diagnostic methods were evaluated using the Public Test Set (Table 1). All five computational methods showed greater performance than all 12 benchmark methods. Notably, PPO, a graph-based probabilistic model, achieved a median rank of 4.0, a top-3 recall of 0.467 (0.434–0.501, Supplementary Table 5), and a top-10 recall of 0.613 (0.581–0.645). The ensemble method achieved a median rank of 4.0, a top-3 recall of 0.483 (0.450–0.517, Table 2 and Supplementary Table 5), and a top-10 recall of 0.640 (0.608–0.672). In comparison, the best of the 12 benchmark methods, MinIC, had a median rank of 6.0, a top-3 recall of 0.424 (0.391–0.457), and a top-10 recall of 0.593 (0.561–0.625).

**Table 2:** Performance of PhenoBrain on rare disease datasets

	Metrics	Public test set (95% CI)	PUMCH-L (95% CI)	PUMCH-ADM (95% CI)	Average
PhenoBrain (Ensemble method)	Top-1 recall	0.304 (0.273–0.334)	0.315 (0.286–0.344)	0.453 (0.347–0.573)	0.357 (0.317–0.397)
	Top-3 recall	0.483 (0.450–0.517)	0.468 (0.436–0.499)	0.587 (0.480–0.693)	0.513 (0.471–0.552)
	Top-10 recall	0.640 (0.608–0.672)	0.630 (0.599–0.660)	0.693 (0.587–0.800)	0.654 (0.616–0.691)
	Median rank	4.0	4.0	2.0	3.3
Best of 12 benchmark methods	Top-1 recall	0.257 (0.229–0.286)	0.294 (0.265–0.322)	0.307 (0.200–0.413)	0.261 (0.226–0.297)
	Top-3 recall	0.424 (0.391–0.457)	0.424 (0.394–0.454)	0.467 (0.360–0.573)	0.441 (0.400–0.480)
	Top-10 recall	0.593 (0.561–0.625)	0.585 (0.554–0.615)	0.653 (0.547–0.760)	0.618 (0.580–0.656)
	Median Rank	6.0	6.0	4.0	5.3

Top-1, Top-3, Top-10 recall rates, and median ranks by PhenoBrain and comparisons with the best results from the 12 benchmark methods using the complete annotation of 9260 rare diseases on three rare disease datasets: the Public Test Set, PUMCH-L, and PUMCH-ADM. For PUMCH-ADM, HPO terms were manually extracted. It should be noted that different benchmark methods performed best on each individual dataset. Complete results can be found in Supplementary Tables 5, 12, 14 and 15.

When the two EHR datasets, PUMCH-L and PUMCH-ADM, were combined, the ensemble method achieved an average median rank of 3.3, a top-3 recall of 0.513 (0.471–0.552), and a top-10 recall of 0.654 (0.616–0.691, Table 2 and Supplementary Table 15). The best performance of the 12 benchmark methods had an average median rank of 5.3, a top-3 recall of 0.441(0.400–0.480), and a top-10 recall of 0.618 (0.580–0.656). Across all datasets, the ensemble method consistently outperformed all benchmark methods. Based on the signed-rank test, it outperformed MinIC on the Public Test Set (*p* < 0.0001; Supplementary Table 20), PUMCH-L set (*p* < 0.0001), and Human-Computer set (*p* < 0.05). Given its superior performance across all datasets, the ensemble method was chosen as the standard method for PhenoBrain. The complete results for each diagnostic method are presented in the Supplementary Tables 5, 12, 14, and 15.

We also conducted a comparative analysis between the ensemble method and three diagnostic tools, Phenomizer^21^, The Likelihood Ratio Interpretation of Clinical Abnormalities (LIRICAL)^14^, and Phen2Disease^13^. Phenomizer is widely recognized as the pioneering phenotype-based diagnostic tool for rare diseases, while LIRICAL and Phen2Disease represent the recent advancement in this domain. Because the diagnostic scopes of the Phenomizer and LIRICAL differ—8012 diseases for Phenomizer and 8167 diseases for LIRICAL—we compared PhenoBrain with each of them separately (see Supplementary Discussion 1). Supplementary Table 6 and Supplementary Table 25 compare the diagnostic performance of PhenoBrain and Phenomizer on the Public Test Set. The ensemble method of PhenoBrain achieved an average median rank of 4.0, a top-3 recall of 0.486 (0.450–0.522), and a top-10 recall of 0.651 (0.616–0.685), outperforming Phenomizer, which achieved a median rank of 15.0, a top-3 recall of 0.256 (0.226–0.288), and a top-10 recall of 0.434 (0.398–0.470). Supplementary Table 7 and Supplementary Table 26 present the diagnostic results of PhenoBrain and LIRICAL on the Public Test set. LIRICAL achieved a median rank of 6.0, a top-3 recall of 0.407 (0.374–0.440), and a top-10 recall of 0.560 (0.526–0.593, Supplementary Table 7), indicating a lower performance compared to the ensemble method of PhenoBrain. Additionally, Phen2Disease was also tested on the entire LIRICAL dataset (384 cases, 262 rare diseases), and it was outperformed by PhenoBrain. Specifically, the ensemble method successfully made 131 and 237 cases for top-1 and top-10 predictions, respectively, surpassing Phen2Disease with counts of 111 and 221 cases. (Supplementary Table 8).

### Performance evaluation of phenotype extraction module and whole AI pipeline

We then assessed the performance of the phenotype extraction method, PBTagger, using a dataset of 86 cases with EHRs from PUMCH-S. As there were no existing extraction tools designed explicitly for HPO on the Chinese data, we compared the results with those obtained from standard dictionary-based NLP approaches using the Chinese HPO (CHPO, https://www.chinahpo.net/) and the UMLS^43^. In this dataset, five experienced physicians from the PUMCH manually annotated HPO phenotypes, which served as the gold standard. Among those methods, PBTagger achieves the highest recall of 0.824 and an F1 score of 0.732. It outperforms both CHPO and UMLS, which achieve a recall of 0.549 and 0.647 and F1 scores of 0.683 and 0.701, respectively (Supplementary Table 24). Furthermore, when using the phenotypes extracted by PBTagger, PhenoBrain achieved a median rank of 3.5, which was slightly inferior to the predicted median rank of 2.0 that used manually extracted phenotypes by physicians. Nonetheless, it was superior to the predicted median ranks of 10.5 and 6.0 achieved by the dictionary-based approaches using CHPO and UMLS, respectively (Supplementary Table 9 and Supplementary Table 10). The improvement in disease prediction results achieved by PBTagger is largely attributed to its enhanced phenotype recall. Although there is a slight decrease in precision, the majority of rare disease prediction algorithms are robust to cases with imprecise or unrelated phenotypes, resulting in an overall improvement in the accuracy of rare disease prediction.

The performance of the PhenoBrain AI pipeline was also evaluated using PUMCH-L. By employing the phenotypes extracted by PBTagger and the ensemble method for diagnosis, PhenoBrain achieved a median rank of 4.0, a top-3 recall of 0.468 (0.436–0.499), and a top-10 recall of 0.630 (0.599–0.660, Table 2 and Supplementary Table 12). Substituting PBTagger with dictionary-based NLP approaches using either CHPO or UMLS resulted in decreased median ranks of 10.5 and 8.0 (Supplementary Table 11), respectively. Additionally, when using the extracted phenotypes from each of the aforementioned phenotype extraction methods, the ensemble method consistently outperformed all other diagnostic methods (*p* < 0.0001), highlighting the superior performance of both PBTagger and the ensemble method. Supplementary Table 13 summarizes the difference in median ranks using various medical text processing methods on the PUMCH-S and PUMCH-L datasets.

### Human-computer comparison in rare diseases diagnosis

As shown in Table 3, the Human-Computer test set includes 75 admitted cases with 16 rare diseases from five clinical departments of PUMCH: Pediatrics, Neurology, Renal, Cardiology, and Hematology, with precisely 15 cases per department. Using this dataset, we conducted a computer versus human experiment to compare the diagnostic performance of PhenoBrain to that of specialist physicians.

**Table 3:** Characteristics of the human-computer test set

	Rare diseases^a^
Number of cases	75
Number of departments	5
Number of diseases	16
Age, years: median (average)	29 (31.6)
Female (%)	36 (48%)
Department: pediatrics	
Number of diseases	3
Number of cases	15
Age, years: median (average)	7.42 (7.40)
Number of physicians	10
Department: neurology	
Number of diseases	3
Number of cases	15
Age, years: median (average)	51 (49.1)
Number of physicians	10
Department: renal	
Number of diseases	3
Number of cases	15
Age, years: median (average)	24 (24.4)
Number of physicians	10
Department: cardiology	
Number of diseases	4
Number of cases	15
Age, years: median (average)	40 (39.1)
Number of physicians	10
Department: hematology	
Number of diseases	3
Number of cases	15
Age, years: median (average)	46 (38)
Number of physicians	10
Physicians	
Total Number of physicians	50
Experience, years: median (average)	11 (12.9)
Number of diagnoses per case: median (average)	3 (2.67)

^a^The 16 rare diseases were: Prader-Willi syndrome (PWS), Hepatolenticular degeneration (HD), McCune-Albright syndrome (MAS), Multiple system atrophy (MSA), Amyotrophic lateral sclerosis (ALS), Generalized myasthenia gravis (GMG), Alport syndrome (AS), Fabry disease (FD), Gitelman syndrome (GS), Marfan syndrome (MFS), Arrhythmogenic right ventricular dysplasia/cardiomyopathy (ARVD/C), Brugada syndrome (BS), Restrictive cardiomyopathy (RCM), POEMS syndrome (POS), Paroxysmal nocturnal hemoglobinuria (PNH), and Niemann-Pick disease (NPD). More details of rare diseases are provided in Supplementary Table 4.

**Fig. 3:**
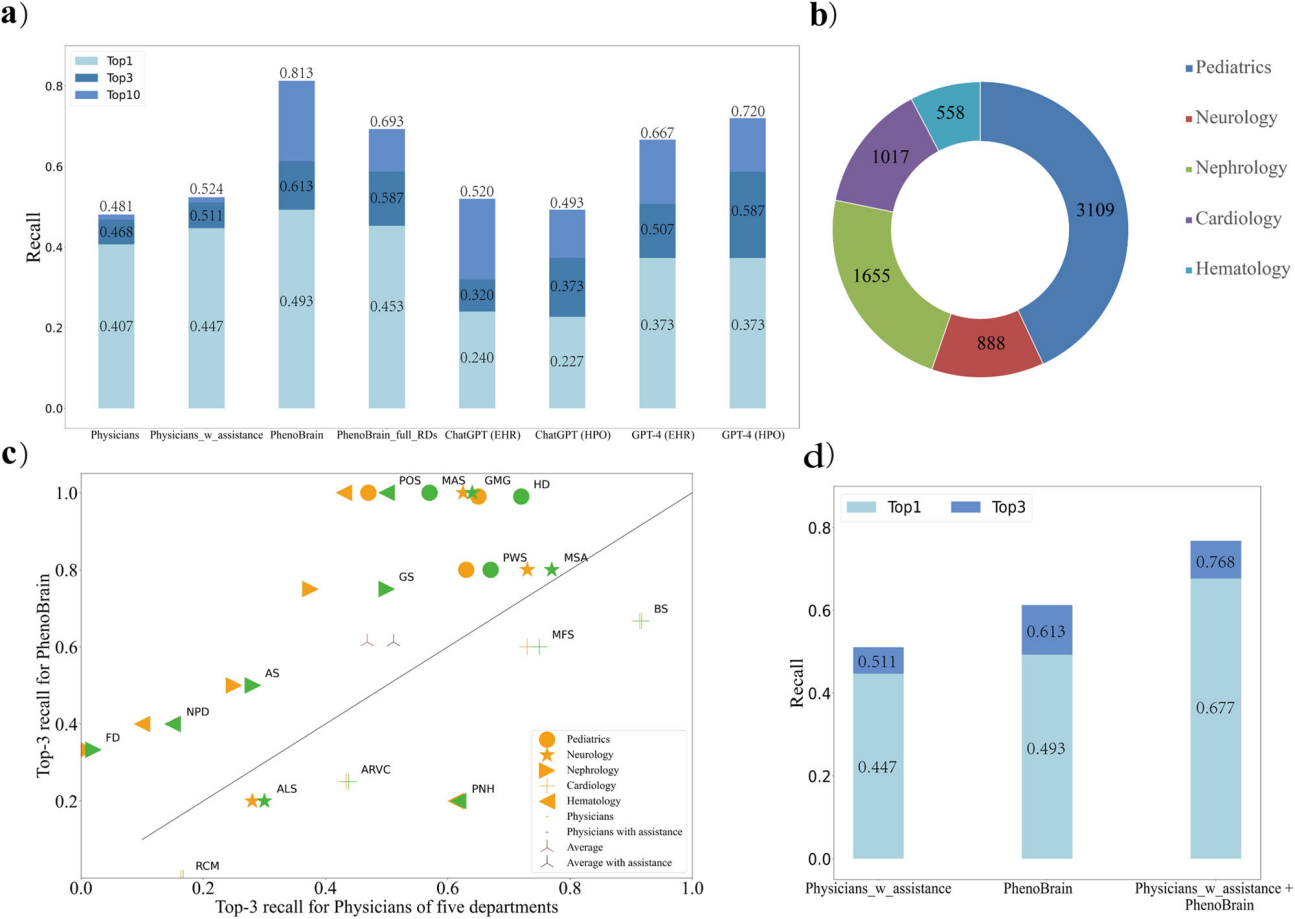
Human-computer performance comparisons and simulated collaborations. **a** Top-1, top-3, and top-10 recall rates for physicians, PhenoBrain, and large language models on 75 admitted cases with 16 rare diseases (RDs) from 5 hospital departments. For each case, physicians made two diagnoses, the first based on their own knowledge and experience (labeled “Physicians”) and the second based on external assistance (labeled “Physicians_w_assistance”). For each case, PhenoBrain provided two results: a disease ranking restricted to a disease subgroup of a department (labeled “PhenoBrain”) and a disease ranking based on all 9260 rare diseases (labeled “PhenoBrain_full_RDs”). For each case, large language models provided two results: a disease ranking based on the input of Electronic Health Record (EHR) data (labeled “EHR”) and a disease ranking based on the input of extracted Human Phenotype Ontology (HPO) terms (labeled “HPO”). **b** The number of RDs for each of the five departments (disease subgroups). **c** Top-3 recall rates of PhenoBrain (restricted to disease subgroups) and physicians on 75 admitted cases. Each dot represents one type of rare disease. The circle, star, right triangle, plus, and left triangle each represent one of the five hospital departments: Pediatrics, Neurology, Renal, Cardiology, and Hematology, in that order. Orange and green represent physicians’ diagnostic accuracy without and with external assistance, respectively, while brown and dark blue represent their average performance, respectively. For convenience, we only added notes to cases diagnosed by physicians with external assistance. **d** Performance of simulated human-computer collaboration by integrating PhenoBrain (restricted to disease subgroups) into clinical workflow.

The performance of PhenoBrain and physicians on PUMCH-ADM is summarized in Table 4 and Fig. 3. We found no statistically significant difference between the performance of the junior and senior groups in diagnosing rare diseases (*p* > 0.05 for both with and without assistance). Consequently, the results of all 50 physicians were combined for the subsequent analysis of this study. However, physicians exhibited improved overall diagnostic accuracy with external assistance, as evidenced by an average top-3 recall increase from 0.468(0.386–0.551) to 0.511(0.428–0.594) (Table 4 and Fig. 3a).

**Table 4:** Performance of PhenoBrain, physicians, and large language model on human-computer test set in five clinical departments

	Recall	Pediatrics	Neurology	Nephrology	Cardiology	Hematology	Average (95%CI)
PhenoBrain (Ensemble method)	Top-1	0.733	**0.600**	**0.533**	0.200	**0.400**	**0.493 (0.387**–**0.600)**
	Top-3	**0.933**	**0.667**	0.533	0.400	**0.533**	**0.613 (0.507**–**0.720)**
	Top-10	**1.000**	**0.867**	0.800	0.533	**0.867**	**0.813 (0.720**–**0.907)**
Physicians	Top-1	0.533	0.533	0.117	0.533	0.317	0.407 (0.323–0.490)
	Top-3	0.567	0.583	0.233	0.583	0.372	0.468 (0.386–0.551)
	Top-10	0.567	0.583	0.267	0.600	0.389	0.481 (0.400–0.563)
Physicians with assistance	Top-1	0.617	0.550	0.150	0.533	0.383	0.447 (0.363–0.530)
	Top-3	0.650	0.600	0.283	0.583	0.439	0.511 (0.428–0.594)
	Top-10	0.650	0.600	0.317	0.600	0.456	0.524 (0.441–0.607)
ChatGPT (EHR)	Top-1	0.400	0.067	0.133	0.467	0.133	0.240 (0.147–0.333)
	Top-3	0.667	0.067	0.200	0.533	0.133	0.320 (0.213–0.427)
	Top-10	0.667	0.400	0.733	0.667	0.133	0.520 (0.413–0.627)
ChatGPT (HPO)	Top-1	0.667	0.067	0.133	0.133	0.133	0.227 (0.133–0.320)
	Top-3	0.800	0.200	0.467	0.200	0.200	0.373 (0.267–0.480)
	Top-10	0.867	0.400	0.667	0.267	0.267	0.493 (0.387–0.600)
GPT-4 (EHR)	Top-1	0.667	0.267	0.267	0.533	0.133	0.373 (0.267–0.480)
	Top-3	0.867	0.333	0.400	0.600	0.333	0.507 (0.400–0.613)
	Top-10	0.933	0.533	0.800	0.667	0.400	0.667 (0.560–0.773)
GPT-4 (HPO)	Top-1	0.800	0.200	0.467	0.267	0.133	0.373 (0.267–0.480)
	Top-3	0.933	0.467	0.667	0.533	0.333	0.587 (0.480–0.693)
	Top-10	1.000	0.667	0.867	0.533	0.533	0.720 (0.613–0.813)

Top-1, Top-3, and Top-10 recall rates on the Human-Computer test set (PUMCH-ADM) in five clinical departments by PhenoBrain (using the disease subgroup for each department), physicians (with or without external assistance), ChatGPT-3.5 (using EHRs or HPO terms, version 2023.06.01), and GPT-4 (using EHRs or HPO terms, version 2023.06.01). For PhenoBrain, HPO terms were manually extracted. ChatGPT-3.5 and GPT-4 input comprise electronic medical records or human phenotype ontology (HPO) terms. We conducted multiple experiments, designing some hard prompts (https://github.com/dair-ai/Prompt-Engineering-Guide), which included Few-Shot prompting, Chain-of-Thought prompting, and Self-Consistency. We selected the one that yielded the best outcomes.

The numbers in bold represent better performance than the best benchmark.

For PhenoBrain, we partitioned the integrated rare disease knowledge base into subgroups according to the clinical departments where rare disease cases would typically be admitted (Fig. 3b). This approach ensured that PhenoBrain diagnosed each case using its subgroup-specific knowledge base, enabling a fair comparison. Figure 3a illustrates that PhenoBrain achieved average top-3 and top-10 recalls of 0.613 (0.507–0.720) and 0.813 (0.720–0.907), respectively, outperforming physicians using external assistance with a *p* value < 0.0001 (Supplementary Table 21). The latter achieved average top-3 and top-10 recalls of 0.511 (0.428–0.594) and 0.524 (0.441–0.607, Supplementary Table 22), respectively.

Additionally, we conducted a comparative analysis between PhenoBrain and two large language models, GPT-4 (version 2023.06.01) and ChatGPT (version 2023.06.01). The results demonstrated that PhenoBrain was superior to both large language models when using EHRs as inputs, with *p* values < 0.05. GPT-4 achieved top-3 and top-10 recalls of 0.507 (0.400–0.613) and 0.667 (0.560–0.773), respectively, while ChatGPT achieved top-3 and top-10 recalls of 0.320 (0.213–0.427) and 0.520 (0.413–0.627), respectively (Table 4 and Supplementary Table 22).

Furthermore, Physicians diagnosed with an average of 2.67 and 2.63 diseases per case without and with external assistance, respectively. When using the top-3 recall rate as the metric, PhenoBrain surpassed physicians in 10 of 16 diseases (Fig. 3c). Notably, in the five departments at PUMCH, PhenoBrain achieved an impressive top-3 recall of 0.933 in pediatrics, compared to 0.650 for physicians with external assistance. In addition, PhenoBrain demonstrated a top-3 recall of 0.533 in nephrology, compared to 0.283 for physicians with external assistance. However, it is worth mentioning that the performance of PhenoBrain varied across multiple rare disease categories (Table 4).

If the subgroup restriction was removed, allowing each case to be diagnosed among the full spectrum of 9260 rare diseases, PhenoBrain achieved a top-3 recall of 0.587 (0.480–0.693) (Table 2, Supplementary Table 14), thereby continuing to outperform physicians with external assistance, who achieved a top-3 recall of 0.511(0.428–0.594). Furthermore, PhenoBrain achieved a top-10 recall rate of 0.693 (0.587–0.800), significantly exceeding the to-10 recall rate of 0.481 (0.400–0.563) and 0.524 (0.441–0.607) obtained by physicians without and with external assistance, respectively (Supplementary Table 22).

### Human-computer collaboration in rare disease diagnosis

Finally, we investigated how PhenoBrain could be integrated into clinical workflow to improve the diagnostic accuracy of rare diseases. Specifically, we explored the possibility of combining the diagnostic results of both physicians and PhenoBrain to achieve a more accurate diagnosis. We simulated a Human-Computer collaboration experiment in which the diagnosis for each case was determined by merging the physicians’ diagnosis with the prediction of PhenoBrain, followed by re-ranking the diagnosed diseases. We observed compelling outcomes that this merging and re-ranking approach yielded an impressive top-1 recall of 0.677 (0.580–0.770, Supplementary Table 22) and a top-3 recall of 0.768 (0.679–0.856) when physicians were provided with external assistance (Fig. 3d, see Supplementary Table 16 for detailed results). These results exceeded the individual performance of physicians or PhenoBrain alone. The substantial improvement can be attributed to the fact that PhenoBrain and physicians each performed well in different categories of rare diseases, thereby complementing each other in the diagnostic process.

## Discussion

This study aimed to develop and validate PhenoBrain, addressing two major challenges in the field of rare disease diagnosis: the lack of experienced physicians and the difficulty of differentiating between numerous diseases. The AI pipeline of PhenoBrain and its modules for phenotype extraction and differential diagnosis have been extensively evaluated using diverse datasets from multiple countries, showing robust performance. In clinical practice, physicians often encounter difficulties in differentiating among a large number of rare diseases. Therefore, PhenoBrain proves its value by substantially reducing the scope of candidate rare diseases for physicians at the outset of the differential diagnosis process, thus aiding them in achieving faster and more precise diagnoses.

In this study, we conducted a comprehensive search on Google Scholar and PubMed, unrestricted by language or date, to gather relevant research on phenotype-based machine learning methods for rare disease diagnosis. The search criteria included specific terms, such as “machine learning” or “deep learning,” “phenotype” or “human phenotype ontology,” and “diagnose rare disease” or “rank rare diseases.” We identified multiple machine learning models and ontology-based semantic similarity measures, with the most recent research published on May 29, 2023. We chose the 13 best and most representative diagnostic methods and two tools from these methods, including Phenomizer, LIRICAL, and Phen2Disease. We then compared them with our models.

The phenotype extraction module of PhenoBrain employed efficient deep learning-based NLP methods, taking advantage of their ability to encode contextual information through data-driven approaches. In EHR datasets, namely PUMCH-S and PUMCH-L, PBTagger demonstrated superior performance compared to dictionary-based NLP methods when it came to extracting disease-related phenotypes. Although PBTagger was specifically trained to analyze Chinese medical texts, the pipeline itself is generalizable and can be trained to recognize HPO terms in other languages if similar medical texts are provided for training.

However, we observed two challenges related to phenotype extraction in cases where it failed. First, the current AI method can extract relevant phenotypes from the original text content but lacks the ability to infer phenotypes from the text using medical knowledge, whereas experienced physicians can easily accomplish this. For example, the presence of phenotypes such as abnormal upper motor neuron morphology (HP:0002127) and abnormal lower motor neuron morphology (HP:0002366) serve as essential clinical diagnostic criteria for amyotrophic lateral sclerosis (ALS) cases. However, deducing these knowledge-condensed HPO phenotypes requires a comprehensive analysis of specific clinical manifestations, physical examinations, and electrophysiological tests instead of straightforward extraction from the plain text of EHRs. PhenoBrain’s inability to deduce these two phenotypes from EHRs resulted in low diagnostic accuracy in all five ALS cases. Had these two phenotypes been deduced successfully, PhenoBrain would have ranked ALS as the top disease in one out of five cases and among the top-3 diseases in three cases (Supplementary Table 17).

Furthermore, another challenge faced by PhenoBrain is to accurately map specific denotative symptoms to HPO vocabulary, despite its ability to deduce HPO phenotypes from various medical data sources, such as medical reports and laboratory test results. This issue was evident in the suboptimal performance of PhenoBrain in the Human-Computer test set for Paroxysmal nocturnal hemoglobinuria (PNH) cases. The AI system struggled to analyze suggestive laboratory test results, such as a positive Ham test and a positive sucrose hemolysis test, which affected its diagnostic accuracy for PNH cases. To overcome these challenges, it is essential to develop advanced machine-learning models that can incorporate more extensive domain knowledge.

Incomplete disease-phenotype annotations in rare disease knowledgebases pose another major challenge in rare disease diagnosis. Although some of the incompleteness may only be addressed by conducting additional research on the diseases, integrating existing knowledgebases offers a quick solution to obtain more comprehensive disease-phenotype annotations. In this study, we merged three rare disease knowledgebases, OMIM, Orphanet, and CCRD, into a unified knowledge base and evaluated various combinations using the Public Test Set. The results demonstrated that integrating these knowledge bases produced more accurate predictions than any individual knowledge base alone (Supplementary Table 18). The integration significantly enhanced the disease-phenotype annotations, improving disease prediction outcomes. For instance, the integrated knowledge base provided annotations for 72 phenotypes associated with methylmalonic academia, whereas Orphanet, OMIM, and CCRD individually annotated 24, 26, and 46 phenotypes, respectively. In 24 cases diagnosed with methylmalonic academia, all diagnostic methods based on the integrated knowledge base outperformed those based solely on Orphanet or OMIM (Supplementary Table 19). Therefore, the completeness of the disease-phenotype annotations played a critical role in prediction accuracy. Moreover, even though the current version of CCRD contained only 144 diseases, its disease-phenotype annotations were of exceptional quality, and their integration with OMIM or Orphanet significantly improved the performance of all 17 diagnostic methods. While the current knowledge base provides a comprehensive set of disease-phenotype annotations, many rare diseases still lack computationally tractable diagnostic evidence. This is because the phenotype annotations for many diseases are not specific enough to differentiate them from others. We counted the number of diseases with non-specific phenotype annotations, where the phenotype annotation is identical to another disease. There are 441 such diseases in total. Additionally, nearly 2000 diseases have fewer than 5 phenotype annotations.

Among the 17 diagnostic methods implemented in PhenoBrain, the ensemble method emerged as the most accurate predictor across all rare disease datasets. This outcome aligns with expectations, as the ensemble model effectively reduces variance, thereby improving accuracy. In addition, the ensemble method demonstrates another significant advantage: its average performance exhibits greater robustness, making it more generalizable to diverse rare disease datasets. It is important to note that diagnostic accuracies can be influenced by factors beyond the model itself, such as the completeness of the disease-phenotype annotations, the presence of identified phenotypes in the cases, and the specificity of phenotypes to specific diseases. The ensemble method, along with other diagnostic methods, demonstrated varying performance across multiple datasets. For instance, the top-3 recall for the ensemble method was 0.483(0.450–0.517) for the Public Test Set, 0.468 (0.436–0.499) for PUMCH-L, and 0.587 (0.480–0.693) for PUMCH-ADM. However, the top-10 recall of the ensemble method was comparatively more consistent: 0.640 (0.608–0.672) for the Public Test Set, 0.630 (0.599–0.660) for PUMCH-L, and 0.693 (0.587–0.800) for PUMCH-ADM. These results demonstrate that while the ensemble method effectively differentiates actual diseases from most other rare diseases, it may encounter challenges in differentiating between similar rare diseases. For example, for cases with only one or two HPO terms, PPO achieved the most robust performance (Supplementary Table 27). One possible explanation is that PPO utilizes more precise and comprehensive probability information, thereby enhancing its robustness, especially for cases with fewer symptoms/phenotypes.

Prior studies evaluated the performance of rare disease diagnostic methods often employed simulated cases due to the limited availability of sizeable rare disease datasets. To enable a comparison with established benchmark methods, we generated four simulated datasets based on phenotype frequencies observed in clinical datasets (Supplementary Data, Supplementary Table 2). Four simulated datasets contained cases with “noise” phenotypes unrelated to the diseases or “imprecise” phenotypes being more general terms in the HPO structure. The ensemble method outperformed all other methods across these simulated datasets (Supplementary Table 23). Four simulated dataset files have been uploaded to GitHub.

The entire AI pipeline of PhenoBrain was evaluated on the PUMCH-L datasets, demonstrating consistent performance. The AI pipeline achieved a top-3 recall of 0.468 (0.436–0.499) and a top-10 recall of 0.630 (0.599–0.660). For the more challenging cases within PUMCH-L, diagnosed by PUMCH’s MDT comprising of physicians from multiple departments, which included 27 cases with 23 rare diseases (Supplementary Table 4), the AI pipeline achieved a top-3 recall of 0.444 (0.259–0.630) and a top-10 recall of 0.593 (0.407–0.778), which were remarkably favorable results. These findings can be attributed to the fact that MDT cases often contain more precise and conclusive plain texts that enable accurate phenotype extraction for disease prediction. In summary, all the results demonstrate that PhenoBrain is an independent and valuable system, offering an accurate top-k candidate list of potential rare diseases.

A recent work assessed the performance of GPT-4 in two tests, one using 75 cases with original narrative texts and the other using a simplified version of these cases with only clinical features represented by HPO terms^33^. The experimental results demonstrated that generative large language models like GPT-4 performed significantly worse on feature-based queries than on text narrative queries. Similarly, our study also explored the applicability of large language models in the domain of rare disease diagnosis. Inspired by using GPT-4 as an AI chatbot^32^, we utilized prompt-engineering techniques to manually design some challenging prompts (https://github.com/dair-ai/Prompt-Engineering-Guide), which included Few-Shot prompting, Chain-of-Thought prompting, and Self-Consistency. These prompts were used to elicit responses from large language models (ChatGPT and GPT-4) regarding patients diagnosed with rare diseases and their corresponding hospital departments. Additionally, we employed GPT-4 to generate instructions and provide in-context information for the experiments. We observed and evaluated the outcomes in the PUMCH-ADM dataset through a series of experiments using these prompts. Based on the results obtained, we identified the most effective prompt for our research purposes (Supplementary Discussion 1). GPT-4 shows remarkable capabilities in predicting potential rare diseases, owing to its extensive training on a vast corpus of data, including health-related information. Nevertheless, it is crucial to acknowledge the limitations and potential risks associated with such models, as they can occasionally generate responses that are entirely unrelated to the given input, commonly referred to as “hallucinations.”

This study is subject to certain limitations. The current version of PhenoBrain does not incorporate genetic information into the diagnostic process. Genetic analysis techniques such as exome sequencing and gene panel analysis are commonly employed to obtain genetic information for disease diagnosis. If a genetic variant is identified as a known disease mutation or a missense mutation located within a known disease gene, it can serve as strong evidence for disease diagnosis when the patient’s phenotypes match those of the disease. However, it is important to note that the genetic causes of many rare diseases remain unknown, with nearly half of the diseases listed in the OMIM database lacking identified genetic causes^44^. Even among diseases with known genetic causes, the available genetic information may be incomplete. Although computational methods^15,18,45,46^, such as Phenolyzer^45^, Xrare^15^, Phen2Gene^17^, and Phen2Disease^13^, can assist in identifying potential disease genes directly from phenotypic data, they rely on existing knowledge of disease-gene associations to infer gene-phenotype relationships. Our diagnostic models can be extended to incorporate genetic information, which is provided as a set of potential disease genes $G$ with variants obtained from DNA sequencing. Using our probabilistic Bayesian model PPO as an example, it computes the probability of diagnosing a disease ${d}_{k}$ given a patient phenotype set $Q$ and an additional set of disease genes $G$, $P({d}_{k}{|Q},G)$, which is proportional to $P\left({d}_{k}\right)P\left(Q,G|{d}_{k}\right),$ according to the Bayesian model. The probability $P\left(Q,G|{d}_{k}\right)={\prod }_{q\in Q}P\left(q|{d}_{k}\right){\prod }_{g\in G}P\left(g|{d}_{k}\right)$ assuming conditional independence between phenotypes and between genes of disease ${d}_{k}$. The added term $P\left(g|{d}_{k}\right)$, the probability of observing a variant in gene $g$ in disease ${d}_{k}$, can be calculated from disease-gene associations and also weighted by the severity of its variant, and the rest will follow the original model. This will be an ongoing effort to improve rare disease diagnosis.

Furthermore, because many rare diseases have many similarities and overlaps in symptoms and signs, the final differential diagnosis is primarily based on the results of objective auxiliary examinations, such as laboratory tests, imaging examinations, and electrophysiological assessments. Therefore, it is difficult to make correct diagnoses relying solely on inpatient text information. However, the current version of PhenoBrain is designed to extract relevant features for diagnosis from EHRs or plain texts derived from laboratory test results, medical imaging diagnostic results, and specialists’ notes. Moreover, PhenoBrain does not differentiate between rare diseases and more common diseases. Finally, the validation study used a retrospective design. It included cases with comprehensive phenotype information, whereas cases with insufficient phenotypic information were not investigated using PhenoBrain.

Due to limitations in the available data, our exploration efforts have primarily focused on the Chinese version of PBTagger. However, to accommodate English users, we have seamlessly integrated existing English HPO extraction tools into PhenoBrain, such as PhenoBERT^35^ and PhenoTagger^34^. In a recent study, PhenoBCBERT and PhenoGPT models were proposed to identify clinical phenotypes in clinical texts from pediatric patients, including some critical phenotypes not included in HPO^47^. There are similarities between Phenobrain’s phenotype extraction module and PhenoBCBERT and PhenoGPT. Both approaches use pre-training models like BERT and fine-tune with limited expert-annotated data. Our methodologies, however, differ significantly from theirs. First, we designed the “Phenotype Linking Model,” a twin structure based on ALBERT and DDML. Second, because some of our medical texts are in Chinese, we were confronted with the challenge of lacking HPO phenotype automatic extraction tools for Chinese medical texts. To address this, we constructed a unified Chinese Medical Meta Thesaurus. Finally, unlike PhenoGPT, which uses annotated clinical notes for training, our training or fine-tuning data consists of annotated synonyms or antonym pairs. Meanwhile, we believe that an HPO extraction tool based on a large language model, such as GPT-4, can offer unified support for multiple languages. This advanced tool will soon be available on the PhenoBrain website, providing enhanced HPO phenotype extraction capabilities across multiple languages.

In conclusion, we developed PhenoBrain, a fully automated AI pipeline for the differential diagnosis of rare diseases. PhenoBrain exhibits the capability to diagnose a wide range of rare diseases and has demonstrated robust performance across datasets from multiple countries. Moreover, we have demonstrated that PhenoBrain outperforms and complements the expertize of specialist physicians, indicating its potential for integration into clinical workflows to improve disease diagnosis.

## Methods

### HPO

HPO^11,48,49^ is a comprehensive and standardized vocabulary encompassing phenotypic abnormalities encountered in human diseases. Within HPO, phenotypic terms are organized in a directed acyclic graph (DAG) structure, interconnected by directed “is-a” (subclass-of) edges. The HPO team annotated all diseases in OMIM with relevant phenotypes sourced from HPO, and they have included information from medical literature, Orphanet, and DECIPHER. For this study, we downloaded the updated version of HPO as of 05 April 2023, which included an extensive collection of over 13,000 phenotypic terms and over 156,000 annotations. Furthermore, given that our dataset comprises inpatient medical records from Peking Union Medical College Hospital, all EHR cases were recorded in Chinese. Therefore, we utilized the Chinese version of the Human Phenotype Ontology (CHPO, corresponding to HPO version 2022-10-05, available at https://www.chinahpo.net/chpo/) as the standard phenotype vocabulary.

### Integrated rare disease knowledge base

To establish a comprehensive knowledge base, we manually extracted associations between rare diseases and phenotypes from the Compendium of China’s First List of Rare Diseases. This resulted in the creation of a new knowledge base called the Compendium of China’s Rare Diseases (CCRD). CCRD included 144 diseases with 4258 disease-phenotype annotations. We integrated three existing knowledgebases: OMIM, Orphanet, and CCRD, and merged disease codes that refer to the same concept. For instance, *Phenylketonuria* was represented as *[OMIM:261600], [ORPHA:716]*, and *[CCRD:90]*, and we consolidated these codes to *[OMIM:261600, ORPHA:716, CCRD:90]*. As a result, we built a comprehensive list of 9260 rare diseases with 168,780 disease-phenotype annotations. It should be noted that more than half of these annotations also provided frequency information. We have uploaded the integrated knowledge base to GitHub to enhance accessibility and ease of use.

### Data description

This study was approved by the Ethics Committees at Peking Union Medical College Hospital, Peking Union Medical College, and the Chinese Academy of Medical Sciences (approval number S-K2051). Before collecting the EHR datasets from PUMCH, patients provided written informed consent during their initial hospital visit. All EHRs have been de-identified, ensuring the removal of sensitive patient information.

RAMEDIS is a public Rare Metabolic Diseases Database developed by scientists from Bielefeld University and Children’s Hospital of Reutlingen in Germany. We initially downloaded 817 medical records with diagnoses mapped to OMIM codes. Subsequently, we manually mapped the clinical terms in the “Symptoms” and “Lab findings” report cards to HPO terms. We impose some reasonable filtering criteria to identify suspicious cases, such as those with uncertain or imprecise diagnoses and those with insufficient relevant information, i.e., fewer than three phenotypes. Finally, we obtained 624 patients with 74 rare diseases (RDs) who had at least three HPO terms and confirmed diagnoses (“Diagnosis confirmed”=“y”). The RAMEDIS dataset was partitioned with a 40% to 60% ratio into a development set and an internal test set, while the other three datasets, MME, HMS, and LIRCAL, were used as external test datasets.

MME is a public Matchmaker Exchange dataset (https://github.com/ga4gh/mme-apis/tree/master/testing) created by the FORGE Canada Consortium and the Care4Rare Canada project (http://care4rare.ca/). This benchmark dataset consists of 50 test cases compiled from the literature by researchers at the Hospital for Sick Children in Canada. Cases with fewer than three HPO phenotypes or lacking a confirmed diagnosis were excluded. Finally, we obtained a dataset containing 40 cases with 17 categories of rare diseases.

HMS, a public dataset from Hannover Medical School in Hannover, Germany, contained 93 adult rare disease cases with pathological symptoms and diagnosed disorders. Cases with fewer than three HPO phenotypes or lacking a confirmed diagnosis were excluded. we manually mapped disease names to disease codes (OMIM, CCRD, and Orphanet) and symptoms to HPO phenotypes, resulting in 88 cases with 39 rare diseases.

LIRICAL, is a collection of published case reports from the scientists of the Jackson Laboratory for Genomic Medicine in the USA, containing 384 case reports comprising 262 rare diseases. We downloaded all 384 cases with phenotypic information and diagnosed disorders. Cases with fewer than three HPO phenotypes or lacking a confirmed diagnosis were excluded. we manually mapped disease names to disease codes (OMIM, CCRD, and Orphanet), resulting in 370 cases with 252 rare diseases.

The initial PUMCH dataset consisted of 1433 cases. We observed an imbalanced distribution of disease types. Specifically, 43 diseases had two or fewer cases, and 34 diseases had at least five cases. From this PUMCH dataset, we chose a small set, PUMCH-S, to train and tune the hyper-parameters of the PBTagger algorithm, leaving the rest for testing. We randomly selected three cases from each of the 34 diseases with at least five cases, and then, five clinicians from PUMCH manually extracted HPO terms from the electronic health records (EHRs) of these cases using a text annotation tool called Brat (http://brat.nlplab.org). In the end, 86 patients with at least three phenotypes were selected for PUMCH-S.

Next, we chose another small set, PUMCH-ADM, to compare the diagnostic performance of physicians to that of our algorithm. The selection criteria include the following factors: (1) that the dataset should be diverse enough to cover a wide range of human organ systems, (2) that there shall be multiple diseases for each system, and (3) that there shall be multiple cases for each disease in order to assess the level of difficulty in diagnosing this disease. Another important consideration is experimental feasibility, which includes workload and data availability. Initially, we observed that diseases with at least 5 cases were predominantly concentrated in five departments (Pediatrics, Neurology, Nephrology, Cardiology, and Hematology). Subsequently, we randomly selected 16 diseases from these five departments. Aside from the Cardiology Department, which included 4 selected diseases, each of the other four departments featured 3 rare diseases. To ensure a standardized evaluation, we maintained a constant number of cases selected in each department, totaling 15 cases. This entire process underwent quality control by two physicians from Peking Union Medical College Hospital in Beijing.

To ensure the integrity of the diagnostic process, any information within the EHRs that could potentially provide clues about the underlying diseases was removed. For example, in cases diagnosed with PNH, all instances of diagnostic terms such as “paroxysmal nocturnal hemoglobinuria” or the abbreviation “PNH” were systematically removed from the clinical text. To maintain consistency, specific exclusion criteria were applied to each case. These criteria included (1) the presence of a sentence containing the full name or abbreviation of a disease, (2) the inclusion of a particular test and its description linked to a rare disease, and (3) the occurrence of a sentence containing genetic information. Medical test figures and tables were retained to facilitate the physicians’ diagnostic process, but they were not used in PhenoBrain due to its inability to process such data. The final discharged diagnosis for each case, as determined by experienced physicians at PUMCH, served as the reference standard for diagnosis. Two expert physicians at PUMCH meticulously verified the accuracy of case information. Further details regarding these cases can be found in Table 3 and Supplementary Table 4. This entire process underwent quality control by two physicians from Peking Union Medical College Hospital in Beijing.

From the remaining cases in the PUMCH dataset, we applied PBTagger to extract standardized HPO phenotype terms. After excluding cases with fewer than three phenotypes, we formed the PUMCH-L dataset, which included 988 cases and 73 rare diseases and was used to assess the entire AI pipeline.

### Diagnostic methods in PhenoBrain

In this study, we proposed five new diagnostic methods, ICTO, a graph-based Bayesian method (PPO), two machine learning methods (CNB and MLP), and an ensemble learning method integrating the results of the first four methods to rank rare diseases effectively. Each method employed a distinct calculation of similarity or probability scores between a given case and all diseases, using these scores to rank the diseases accordingly. Assume $Q$ to be a list of query phenotypes associated with a case, ${H}_{k}$ to be a set of HPO phenotypes associated with the $k$-th ${d}_{k}$ disease in the knowledge base, $A\left({H}_{k}\right)$ to denote the phenotype set including all phenotypes in ${H}_{k}$ and their ancestors in the HPO hierarchy, and ${f}_{{ch}}\left(t\right)$, which correspond to the children set of an HPO term $t$.

- ICTO, a model for measuring semantic similarity, calculates a similarity score by summing the information content (IC) of the overlapping phenotypes between $$Q$$ and $${H}_{k}$$. Our analysis of various clinical datasets shows that patients often have many phenotypes that are not annotated by their diagnosed diseases (Supplementary Table 2). In other words, a patient may have many phenotypes (in $$Q)$$ that are not present in $${H}_{k}$$. Previous methods, such as Res, MinIC, Lin, and JC, are far too sensitive to these phenotypes to diagnose accurately. ICTO, on the other hand, is insensitive to these phenotypes and performs better in these patients. The one-side similarity score is defined as the Eq. (1):
  1 $$S{im}\left(Q\to {H}_{k}\right)=\sum _{t\in \left(Q\cap A\left({H}_{k}\right)\right)}{IC}\left(t\right)$$
  Where ${IC}\left(t\right)=-\log P(t)$ and $P(t)$ is the fraction of all diseases that are annotated with phenotype $t$. Thus, the symmetric version of the similarity score averages the two one-side scores as Eq. (2):
  2 $$S{im}\left(Q,{H}_{k}\right)=\frac{1}{2}\left[{Sim}\left(Q\to {H}_{k}\right)+{Sim}\left({H}_{k}\to Q\right)\right]$$
- PPO (Probability Propagation in Ontology), is a probabilistic Bayesian model that computes the probability of diagnosing a disease ${d}_{k}$ given a patient phenotype set $Q$ using the Bayes’ rule in the following Eq. (3):
  3 $$P\left({d}_{k}|Q\right)\propto P\left({d}_{k}\right)P\left(Q|{d}_{k}\right)=P\left({d}_{k}\right)\prod _{t\in Q}P\left(t|{d}_{k}\right)$$
  assuming conditional independence of phenotypes in $Q$ given disease ${d}_{k}$. In other words, if a patient has a disease, then all symptoms associated with the disease are independent from one another. The model parameters, $P\left(t|{d}_{k}\right)$, the probability of observing a phenotype $t$ in disease ${d}_{k}$, can be either derived from the existing phenotype frequency information in disease annotations, or inferred based on the ontology structure of HPO and the phenotype annotations.
  Specifically, $P\left(t|{d}_{k}\right)$, is defined using a recursive function in the following Eq. (4):
  4 $$P\left(t|{d}_{k}\right)=\left\{\begin{array}{l}{p}_{{tk}}\,{\rm{or}}\,{dp}\qquad{\rm{if}}\,t\in {H}_{k}\\ {f}_{{pp}}\left(t,{d}_{k}\right),\qquad{\rm{if}}\,t\in A\left({H}_{k}\right)\\ P\left(t\right),\qquad\qquad{\rm{otherwise}}\end{array}\right.{{\backslash }}{H}_{k}$$
  If $t\in {H}_{k}$, we use the phenotype frequency provided by the knowledge base, ${p}_{{tk}}$, or a default probability ${dp}$ if ${p}_{{tk}}$ is unknown. If $t$ is a more general ancestor phenotype for ${H}_{k}$ but excluding ${H}_{k}$,$t\in A\left({H}_{k}\right)\backslash {H}_{k}$, we propagate the frequencies of its descendant phenotypes upward recursively using ${f}_{{pp}}$. If $t$ is unrelated to disease $k$, we use the background probability $P\left(t\right)$, the fraction of all diseases annotated with phenotype $t$. Let ${{\rm{C}}}_{k}$ denote the intersection between the children of $t$ and the ancestors of ${H}_{k}$:
  5 $${C}_{k}={f}_{{\!ch}}\left(t\right)\cap A\left({H}_{k}\right)$$
  Then three recursive functions for ${f}_{{pp}}$ are calculated as the Eqs. (6)–(8):
  6 $${f}_{\max }\left(t,{d}_{k}\right)=\mathop{\max }\limits_{t\in {C}_{k}}P\left(t|{d}_{k}\right)$$
  7 $${f}_{{ind}}\left(t,{d}_{k}\right)=1-\prod _{t\in {C}_{k}}\left[1-P\left(t|{d}_{k}\right)\right]$$
  8 $${f}_{{sum}}\left(t,{d}_{k}\right)=\min \left(1,{\sum }_{t\in {C}_{k}}P\left(t|{d}_{k}\right)\right)$$
  where ${f}_{\max }$ calculates the probability $P\left(t|{d}_{k}\right)$ by using the most informative children of $t$ in ${C}_{k}$, and by contrast, ${f}_{{ind}}$ and ${f}_{{sum}}$ use information of all children of $t$ in ${C}_{k},$ assuming the information multipliable and additive, respectively.
  For convenience, we assumed $P\left({d}_{k}\right)$ to be a constant for all rare diseases and calculated the logarithm of the conditional probability $P\left({d}_{k}|Q\right)$ as the Eq. (9):
  9 $$\log P\left({d}_{k}|Q\right)\propto \sum _{t\in Q}\log P\left(t|{d}_{k}\right)$$
  The computation of PPO requires knowledge of the frequency of each phenotype under each disease. However, in the HPO phenotype annotation data, probabilities are often provided only for the most specific phenotypes associated with a disease. The probabilities for more generalized (higher-level) phenotype nodes are unknown. PPO addresses this issue recursively. Compared to methods using information content (IC), PPO utilizes more precise and comprehensive probability information. The experimental results demonstrate its robustness across all datasets.
- Few-shot techniques: Machine learning methods can train a multi-class disease classification model but require a sufficient amount of training data per class. However, there are no publicly accessible rare disease datasets that can cover all rare diseases; the only complete “data” we have are the disease-phenotype annotations provided by rare disease knowledgebases. This machine learning problem is known as few-shot learning, where the training data typically contains between 1 and 5 cases per class. Each disease with its associated phenotype annotations is considered a “standard case”, and the training data initially consisted of a single case for each class. Then, additional training data can be generated using two data augmentation techniques, Mixup^50^ and Random Perturbation. Mixup generates new samples by linearly interpolating two randomly selected cases. In contrast, Random Perturbation substitutes a portion of phenotypes of a case with either more general phenotypes (ancestors) or unrelated phenotypes (noises). After evaluating a variety of machine learning models, we identified two effective methods: Complement Naive Bayesian (CNB)^51^ and Multilayer Perceptron (MLP)^52^.
  For each disease ${d}_{k}$ and its phenotype annotation ${H}_{k}$, we construct a case $({x}_{i},{y}_{i})$, where ${x}_{i}\in {\left\{\mathrm{0,1}\right\}}^{M}$ is a multi-hot vector representing the presence or absence of each of the $M$ phenotypes (including the ancestors) associated with the disease$:$
  10 $${x}_{{ij}}=\left\{\begin{array}{l}1,{\rm{if}}{t}_{j}\in A\left({H}_{k}\right)\\ 0,{\rm{otherwise}}\end{array}\right.$$
  and ${y}_{i}=k$ is the label for disease $k$. In this way, we establish a training set $D=[\left({x}_{1},{y}_{1}\right),\ldots ,\left({x}_{T},{y}_{T}\right)$], and we also covert each query case $Q$ into a multi-hot phenotype vector $q=\left({q}_{1},\ldots ,{q}_{M}\right)$.
- CNB, or Complement Naïve Bayesian, is a variant of the multinomial naïve Bayesian method that calculates a probability $P({H}_{k}{|Q})$ using statistics from the complement of each class. First, the fraction of cases without disease $k$ while having phenotype ${t}_{j}$ is defined as Eq. (11):
  11 $${\theta }_{{kj}}=\frac{{{\rm{\alpha }}}_{j}+{\sum }_{{y}_{i}\ne k}{x}_{{ij}}}{{\rm{\alpha }}+{\sum }_{{y}_{i}\ne k}{\sum }_{j{\prime} =1}^{M}{x}_{{ij}{\prime} }}$$
  where ${\alpha }_{j}$ is a smoothing hypermeter and $\alpha ={\sum }_{j=1}^{M}{a}_{j}.$
  Given a query case $Q$ with a phenotype vector $q=({q}_{1},\ldots ,{q}_{M})$, the probability that it is not classified as a disease $k$ as the Eq. (12):
  12 $$P\left({y}_{i}\,\ne\, k|{x}_{i}\right)=\frac{P\left({y}_{i}\,\ne\, k\right)P\left({x}_{i}|{y}_{i}\,\ne\, k\right)}{P\left({x}_{i}\right)}$$
  $$=\frac{P\left({y}_{i}\,\ne\, k\right)}{P\left({x}_{i}\right)}\mathop{\prod }\limits_{j=1}^{M}P{\left({x}_{{ij}}=1|{y}_{i}\,\ne\, k\right)}^{{x}_{{ij}}}$$
  $$=\frac{P\left({y}_{i}\,\ne\, k\right)}{P\left({x}_{i}\right)}\mathop{\prod }\limits_{j=1}^{M}{{\rm{\theta }}}_{{kj}}^{{x}_{{ij}}}=c\cdot \mathop{\prod }\limits_{j=1}^{M}{{\rm{\theta }}}_{{kj}}^{{x}_{{ij}}}$$
  Here we assume the prior probability $P\left(y\,\ne\, k\right)$ is the same constant for all diseases. Since our goal is to rank the diseases in descending order according to the values of $P\left(y\,=\,{\rm{k}}|q\right)=1-P\left(y\,\ne\, k|q\right)$ for $k=\mathrm{1,2},\ldots ,$, we just compute $\mathop{\prod }\nolimits_{j=1}^{M}{{\rm{\theta }}}_{{kj}}^{{q}_{j}}$ for $k=\mathrm{1,2},\ldots$, and arrange them into ascending order. For convenience, we compute the logarithm instead:
  13 $$\log \mathop{\prod }\limits_{j=1}^{M}{\theta }_{{kj}}^{{q}_{j}}=\mathop{\sum }\limits_{j=1}^{M}{q}_{j}{\log (\theta }_{{kj}})$$
- MLP, or Multilayer Perceptron, is a feedforward artificial neural network that calculates a probability of ${H}_{k}$ given $Q$. It usually consists of an input layer, one or multiple hidden layers, and an output layer. In the context of few-shot learning for rare diseases, where the training data is limited, we adopt an MLP model with a single hidden layer. The MLP model is trained using the backpropagation algorithm, which minimizes the binary cross entropy loss function with $l2$ regularization on the weights. The goal is to optimize the model parameters to achieve accurate classification of diseases based on the given phenotypes. To increase the training data, we use two strategies for data argumentation: Mixup and Random Perturbation. Mixup^50^ applies linear interpolation to construct new samples from pairs of randomly selected samples in the training set. Given two samples $\left({x}_{i},{y}_{i}\right)$ and $({x}_{j},{y}_{j})$, a new sample $\left(\widetilde{x},\widetilde{y}\right)$ is generated by combining the phenotypes and labels with a mixing coefficient λ:
  14 $$\widetilde{x}=\lambda {x}_{i}+\left(1-\lambda \right){x}_{j},\widetilde{y}=\lambda {y}_{i}+\left(1-\lambda \right){y}_{j}$$
  where $$\lambda \sim {Beta}\left(\alpha ,\alpha \right)$$ with $$\alpha \in \left(0,\infty \right)$$.
  Random Perturbation introduces variations to the phenotypes of a training sample $\left({x}_{i},{y}_{i}\right)$. It randomly selects $K$ phenotypes from the phenotype set ${x}_{i}$ and applies a mixture of operations such as removal, replacement with more general (ancestor) phenotypes, replacement with more specific (descendant) phenotypes, or addition of “noise” phenotypes that are neither ancestors nor descendants. This process generates new samples with different combinations of phenotypes, expanding the training set and improving the model’s ability to handle diverse cases.
- The Ensemble model. We observed that a single assumption or model would not completely capture the characteristics of the diverse datasets. Hence, we developed the Ensemble model by combining predictions from multiple methods using order statistics, and it achieved better results. The Ensemble model calculates one overall prediction by integrating the rankings of the previous four methods using order statistics. Suppose the number of methods is $N$. First, the Ensemble method normalizes the ranking of diseases within each method to obtain ranking ratios. It then calculates a $Z$ statistic, which measures the likelihood that the observed ranking ratios are solely due to chance factors. It calculates the probability of obtaining ranking ratios through random factors that are smaller than the currently observed ranking ratios. Under the null hypothesis, the position of each disease in the overall ranking is random. In other words, for two diseases, the one with a smaller $Z$ statistic is more likely to have a top rank. The joint cumulative distribution of an N-dimensional order statistic is used to calculate the $Z$ statistics^53^:

15 $$Z\left({r}_{1},{r}_{2},\ldots ,{r}_{N}\right)=N!{\int }_{\!\!0}^{{r}_{1}}{\int }_{{\!\!s}_{1}}^{{r}_{2}}...{\int }_{{\!\!s}_{N-1}}^{{r}_{N}}d{s}_{N}\,d{s}_{N-1}\ldots d{s}_{1}$$

where ${r}_{i}$ is the rank ratio by the $i$-th method, and ${r}_{0}=0$. Due to its high complexity, we implemented a faster recursive formula to compute the above integral as previously done^40^.

16 $${V}_{k}=\mathop{\sum }\limits_{i=1}^{k}{\left(-1\right)}^{i-1}\frac{{V}_{k-i}}{i!}{r}_{N-K+1}^{i}$$

17 $$Z\left({r}_{1},{r}_{2},\ldots ,{r}_{N}\right)=N!{V}_{N}$$

where ${V}_{0}=1$, and ${r}_{i}$ is the rank ratio by the $i$-th method.

Then, the cumulative distribution function of the fitted distribution based on the $Z$ statistic provides an approximate P-value for disease ranking. When $N\le 5$, the $Z$ statistic is approximated to follow a Beta distribution; when $N\, >\, 5$, a Gamma distribution is used.

Moreover, as part of our study, we reproduced and compared 12 benchmark methods, which can be categorized into two distinct groups. The first category contained six previously developed algorithms for rare disease predictions, including Res^21^, BOQA^22^, RDD^26^, GDDP^29^, RBP^27^, and MinIC^25^. The second category contained commonly used ontology-based semantic similarity measures^54^, including Lin^55^, JC^56^, SimGIC^57^, SimUI (Jaccard index)^58^, TO (Term Overlap)^59^, and Cosine. We compared our proposed methods with these 12 benchmark methods for a comprehensive evaluation of the performance of our methods.

To optimize the performance of PhenoBrain, we fine-tuned the hyper-parameters of the aforementioned methods using randomly selected 249 public cases. This process involved adjusting the default probability ${dp}$ of PPO, the smoothing factor ${{\rm{\alpha }}}_{{\rm{j}}}$ of CNB, and the learning rate $\lambda$ of MLP. Furthermore, we also utilized this subset to fine-tune specific hyper-parameters for baseline models.

### HPO phenotype extraction method in PhenoBrain

PBTagger, the phenotype extraction module in PhenoBrain, extracted symptoms (medical entities) from clinical notes before mapping them into HPO phenotypes. Due to the absence of a unified medical thesaurus such as UMLS^43^ and medical text analyzing tools such as MetaMap^60^ and cTAKES^61^ for the Chinese language, we compiled a unified Chinese medical thesaurus by collecting and integrating several existing medical thesauruses, including Chinese HPO, ICD-10^62^, SNOMED-CT^63^, MeSH^64^, and the Chinese translation of UMLS. Further details regarding the Unified Chinese Medical Thesaurus and the construction of the training dataset can be found in Supplementary Note 2.

First, PBTagger extracts suspective medical entities from a given clinical text using the TopWORDs algorithm. Second, the HPO linker (Supplementary Fig. 1), a trained BERT-based deep learning model, maps the entities to the HPO vocabulary based on the mentioned unified Chinese medical thesaurus. Specifically, we built the HPO linker by adopting ALBERT^38^, a lite version of the most popular NLP model BERT, which reduced the size of BERT while maintaining its performance. The model transforms a medical entity $t$ into a semantic vector ${V}_{t}={G}_{e}\left(t,{W}_{e}\right)$, where ${G}_{e}$ represents the embedding network, and ${W}_{e}$ represents the corresponding network parameters. If two medical entities, ${t}_{i}$ and ${t}_{j}$, belong to the same concept, their embedded distance, $d({V}_{{t}_{i},}{V}_{{t}_{j}}),$ will be smaller than if they do not.

Before applying the ALBERT model, we first compiled a training set using unified Chinese medical thesaurus and the HPO vocabulary. Then we trained the model using the loss function, which is used in discriminative deep metric learning^39^:

18 $${Loss}=\mathop{\sum}\limits_{{t}_{i},{t}_{j}}{\left[1-{l}_{{t}_{i},{t}_{j}}\left({\rm{\tau }}-{d}^{2}\left({v}_{{t}_{i}},{v}_{{t}_{j}}\right)\right)\right]}_{+}$$

where ${l}_{{t}_{i},{t}_{j}}$ is set to 1 if ${t}_{i}$ and ${t}_{j}$ belong to the same concept, and -1 otherwise, $\tau$ is a hyperparameter, and $d$ denotes the Euclidean distance. To compute the similarity score between a text ${t}_{s}$ and a standard HPO phenotype term ${t}_{h}$, we use the embedded vectors ${v}_{{t}_{s}}$ and ${v}_{{t}_{h}}$ computed by ${G}_{e}.$ The similarity score is calculated by:

19 $$S\left({t}_{s},{t}_{h}\right)=-{d}^{2}\left({v}_{{t}_{s}},{v}_{{t}_{h}}\right)$$

The HPO phenotype terms are scored against the text, and subsequently ranked according to their similarity scores.

The ALBERT model was implemented using the framework of Tensorflow, while the TopWORDS was implemented using C + + and Python. Details of the implementation are given in Supplementary Note 2.

### Implementation of a human-computer comparison experiment

A total of 50 physicians participated in this study, including 25 senior physicians and 25 junior physicians from 23 Class-A tertiary hospitals in China. The senior physicians averaged 16 years of clinical experience, whereas the junior physicians averaged 9.6 years.

In this experiment, each record was comprehensive, including the patient’s medical history, auxiliary examinations, physical assessments, and laboratory results, thereby offering enough information for preliminary diagnoses. Each physician was assigned six cases admitted to their respective department. For each case, four physicians provided a diagnosis separately. Physicians were instructed to make two diagnoses for each case: the first without external assistance and the second with external assistance, such as online resources, medical literature, or medical records of patients with similar symptoms. Multiple diagnoses were permitted for each case, with the order representing the confidence level of each diagnosis, from highest to lowest.

In addition, we also inputted each case into both ChatGPT and GPT-4, using either EHRs or the extracted HPO terms for each case. We designed and tested multiple prompts, and the one that produced the best predictions was selected for further analysis.

### Implementation of a human-computer collaboration experiment

We conducted a comprehensive simulation study to investigate the potential integration of the AI system into the clinical workflow, with the aim of improving diagnostic accuracy. We used order statistics to merge the disease rankings generated by physicians and PhenoBrain. The detailed result can be found in the Supplementary Table 16.

### Statistical metrics and analyses

In evaluating the performance of each method, we applied several metrics (see Supplementary Note 1), including Median Rank, Recall, and top-k Recall. The top-k Recall (hit@k) measures the correctness of the classification by considering a case as correctly diagnosed if the actual disease is ranked among the top-k in the predicted list. The Median Rank is the median of the ranks of the correct diagnoses in the predictions across all cases. For cases diagnosed with multiple diseases, metrics were calculated based on the top-ranked (first-hit) disease. In other words, the final rank result is determined by the best rank among all target diseases.

To determine the statistical significance and assess the deviation of prediction rankings from a normal distribution, we applied a nonparametric method called bootstrapping, which, recommended by Dror et al.^65^, computes with 10,000 repetitions to calculate confidence intervals. In addition, we performed a signed-rank test to evaluate the statistical significance of one method outperforming another, specifically in terms of the Median Rank metric. A *p* value of <0.05 was considered significant.

## Supplementary information

SUPPLEMENTAL MATERIAL

Supplementary Data 1

Supplementary Data 2

## Data Availability

The de-identified patient data presented in Table 1 can be accessed on Zenodo (https://zenodo.org/records/10774650). Four simulated dataset files have been uploaded to GitHub. (https://github.com/xiaohaomao/timgroup_disease_diagnosis/data/simulated). Further data on research design is available upon request from the corresponding author (tingchen@mail.tsinghua.edu.cn).

## Code availability

The Pre-trained ALBERT model used in this study is available at https://github.com/google-research/ALBERT. The PhenoBrain source code and model parameters are available at https://github.com/xiaohaomao/timgroup_disease_diagnosis.
