# Supplementary material for: A phenotype-based AI pipeline outperforms human experts in differentially diagnosing rare diseases using EHRs
Source: NPJ Digit Med. 2025 Jan 28;8:68. doi: 10.1038/s41746-025-01452-1 (PMC11775211; doi:10.1038/s41746-025-01452-1)
Supplement: Supplementary file 1 — SUPPLEMENTAL MATERIAL [file 41746_2025_1452_MOESM1_ESM.docx]

**Supplementary**

**A Phenotype Based AI Pipeline Outperforms Human Experts in Differentially Diagnosing Rare Diseases Using EHRs**

Xiaohao Mao^1^, Yu Huang^1,2^, Ting Chen^1^

^1^ Department of Computer Science and Technology & Institute for Artificial Intelligence & BNRist, Tsinghua University, Beijing, China

^2^ Tencent Jarvis Lab, Shenzhen, China

TABLE OF CONTENTS

[Supplementary Note 1 4](#_Toc183255617)

[Metrics Notation 4](#_Toc183255618)

[Metrics Used to Evaluate Phenotype Extraction Performance 4](#_Toc183255619)

[Metrics Used to Evaluate Differential Diagnosis Performance 4](#_Toc183255620)

[Supplementary Note 2 5](#_Toc183255621)

[PBTagger 5](#_Toc183255622)

[TopWORDS 5](#_Toc183255623)

[Model Structure of HPO Linker 6](#_Toc183255624)

[Implementation Details 6](#_Toc183255625)

[Training Set of PBTagger 6](#_Toc183255626)

[Supplementary Note 3 7](#_Toc183255627)

[Implementation Details of Differential Diagnosis 7](#_Toc183255628)

[Supplementary Data 7](#_Toc183255629)

[Simulated Datasets 7](#_Toc183255630)

[Public Test Sets 8](#_Toc183255631)

[EHR Test Sets 8](#_Toc183255632)

[Supplementary Discussion 1 9](#_Toc183255633)

[Comparison with Phenomizer and LIRICAL 9](#_Toc183255634)

[Merged different rare disease knowledgebases 9](#_Toc183255635)

[Prompt Example 10](#_Toc183255636)

[Supplementary Figure 1 12](#_Toc183255637)

[Supplementary Tables 12](#_Toc183255638)

[Supplementary Table 1. Characteristics of rare disease knowledgebases 12](#_Toc183255639)

[Supplementary Table 2. Ratios of case phenotypes matched with disease phenotypes in rare disease datasets 12](#_Toc183255640)

[Supplementary Table 3. Top 5 diseases in rare disease datasets 14](#_Toc183255641)

[Supplementary Table 4. Disease Names and Number of Cases for two PUMCH Datasets 16](#_Toc183255642)

[Supplementary Table 5. Performance of 17 diagnostic methods on the Public Test Set (873 cases) 17](#_Toc183255643)

[Supplementary Table 6. Performance of Phenomizer and 5 proposed methods on Public Test Set* (753 cases) 18](#_Toc183255644)

[Supplementary Table 7. Performance of LIRICAL tool and 5 proposed methods on Public Test Set* (850 cases) 18](#_Toc183255645)

[Supplementary Table 8. Performance of PhenoBrain and various methods of Phen2Disease on Cohort 1* (384 cases) 18](#_Toc183255646)

[Supplementary Table 9. Median ranks of 17 diagnostic methods on test subsets of PUMCH-S (34 cases)using various medical text processing tools 19](#_Toc183255647)

[Supplementary Table 10. Performance of 17 diagnostic methods on test subsets of PUMCH-S (34 cases) using PBTagger 19](#_Toc183255648)

[Supplementary Table 11. Median ranks of 17 diagnostic methods on PUMCH-L using various medical text processing tools 20](#_Toc183255649)

[Supplementary Table 12. Performance of 17 diagnostic methods on PUMCH-L 20](#_Toc183255650)

[Supplementary Table 13. Difference of median ranks using various medical text processing methods* 21](#_Toc183255651)

[Supplementary Table 14. Performance of 17 diagnostic methods on PUMCH-ADM and full 9260 rare diseases 22](#_Toc183255652)

[Supplementary Table 15. Average Performance of 17 diagnostic methods on Public Test Set, PUMCH-L and PUMCH-ADM 22](#_Toc183255653)

[Supplementary Table 16. Diagnostic Results of Simulated Human-Computer Collaborations 23](#_Toc183255654)

[Supplementary Table 17. Median ranks of 5 ALS cases by PhenoBrain 24](#_Toc183255655)

[Supplementary Table 18. Median ranks of 17 diagnostic methods using various knowledgebases on the Public Test Set 25](#_Toc183255656)

[Supplementary Table 19 Median ranks among 17 diagnostic methods using various knowledgebases on 24 Methylmalonic academia cases 25](#_Toc183255657)

[Supplementary Table 20. P-values for comparing the Ensemble method against the 12 diagnostic methods on rare disease datasets* 26](#_Toc183255658)

[Supplementary Table 21. P-values for comparing ensemble method against physicians and large language models on Human-Computer Test Set* 26](#_Toc183255659)

[Supplementary Table 22. Performance of ensemble method and physicians and large language models on Human-Computer Test Set 26](#_Toc183255660)

[Supplementary Table 23. Performance of 17 diagnostic methods on simulated datasets 27](#_Toc183255661)

[Supplementary Table 24. Performance of various medical text processing methods on test subsets of PUMCH-S (34 cases) 29](#_Toc183255662)

[Supplementary Table 25. Performance of Phenomizer and 5 proposed methods on Public Test Set* (753 cases) Using Phenomizer's KnowledgeBase 29](#_Toc183255663)

[Supplementary Table 26. Performance of LIRICAL tool and 5 proposed methods on Public Test Set* (850 cases) Using LIRICAL's KnowledgeBase 29](#_Toc183255664)

[Supplementary Table 27. Performance of 17 diagnostic methods and 2 tools on the 101 cases (public set) with less than 3 phenotypes 30](#_Toc183255665)

[Supplementary References 31](#_Toc183255666)

# Supplementary Note 1

## Metrics Notation

HPO terms, which describe phenotypic abnormalities, are arranged in a hierarchical structure, with more general phenotypes at the higher level (parent terms) and more specific phenotypes at the lower level (child terms). To describe the relationships within this hierarchy, we define three functions: $f_{a}\left( t \right), f_{pa}\left( t \right),$ and$f_{ch}\left( t \right)$, which correspond to the ancestor set, parent set, children set of an HPO term $t$, respectively. For a given set of HPO terms $T={\{t}_{1},t_{2},\ldots,t_{m}\}$, we define $A\left( T \right)$ to denote this set along with all its ancestors:

|  | $A\left( T \right)=T\cup f_{a}\left( t_{1} \right)\cup f_{a}\left( t_{2} \right)\cup\ldots\cup f_{a}\left( t_{m} \right)$ | (1) |
| --- | --- | --- |
|  |  |  |

## Metrics Used to Evaluate Phenotype Extraction Performance

Let $T_{p}$ denote the gold standard phenotypes extracted by physicians, and $T$ denote the set of phenotypes extracted by an NLP method. The following definitions are used to calculate true positive $TP,$ false positive $FP$, and false negative $FN$:

|  | $TP=\left\vert A\left( T \right)\cap A\left( T_{p} \right) \right\vert, FP=\left\vert A\left( T \right)-A\left( T_{p} \right) \right\vert, FN=\left\vert A\left( T_{p} \right)-A\left( T \right) \right\vert$ | (2) |
| --- | --- | --- |

Then, the Recall, Precision, and F1 scores are calculated using the following formulas:

|  | $Recall=\frac{TP}{TP+FN}=\frac{TP}{\left\vert A\left( T_{p} \right) \right\vert}$ | (3) |
| --- | --- | --- |

|  | $Precision=\frac{TP}{TP+FP}=\frac{TP}{\vert A\left( T \right)\vert}$ | (4) |
| --- | --- | --- |

|  | $F1=2*\frac{Recall* Precision}{Recall +Precision}$ | (5) |
| --- | --- | --- |
|  |  |  |

## Metrics Used to Evaluate Differential Diagnosis Performance

In evaluating the performance of differential diagnosis, we define a best set $G=\left\{ \left( x_{1},y_{1} \right),\ldots,\left( x_{i},y_{i} \right),\ldots,\left( x_{\left| G \right|},y_{\left| G \right|} \right) \right\}$, where $x_{i}$ represents a multi-hot encoding vector for the phenotypes of the $i$-th case, $y_{i}\in\{1,2,\ldots,N\}$represents the label of the corresponding disease$,$and $N$ is the number of diseases in the knowledgebase. A diagnostic model takes $x_{i}$as input and generates a ranked list of diseases, with the most likely disease at the top. We define $r_{i}=rank(y_{i})$ as the rank of the true disease in this list. We consider the true disease to be among the top-$k$ predictions if $r_{i}\leq k$.We used $TP^{k}$ and $FN^{k}$to represent the total number of true positives and false negatives, respectively, based on the top $k$ predictions in the test set:

|  | $TP^{k}=\sum_{i=1}^{\left\vert G \right\vert} \boldsymbol{1}\left( r_{i}\leq k \right)$ | (6) |
| --- | --- | --- |

|  | $FN^{k}=\sum_{i=1}^{\left\vert G \right\vert} \boldsymbol{1}\left( r_{i}>k \right)=\left\vert G \right\vert-TP^{k}$ | (7) |
| --- | --- | --- |

Here **1** denotes the indicator function.

**Recall**

Recall@k, also known as top-k recall, calculates the fractions of true positives among all cases, and it is calculated as follows:

|  | $Recall@k= \frac{TP^{k}}{\left\vert G \right\vert}$ | (8) |
| --- | --- | --- |

**Median Rank**

Median Rank calculates the median of the true label’s ranks among all cases, and it is defined as:

|  | $Median Rank=median(r_{1},\ldots,r_{\vert G\vert} ))$ | (9) |
| --- | --- | --- |

# Supplementary Note 2

## PBTagger

PBTagger is a fully automated deep learning-based pipeline for extracting standard HPO terms from medical texts. It consists of two main components. The first component is TopWORDS^1^, an unsupervised word discovery method applied to EHRs to generate a candidate list of entities. The second component is an HPO linker based on deep learning, which maps each entity in the list into standard phenotype terms in HPO. It should be noted that any extracted terms will be discarded if a sentence or an entity contains negative words, such as “not”.

## TopWORDS

TopWORDS^1^ is an efficient unsupervised model for word discovery and text segmentation in domain-specific Chinese or English texts. It has demonstrated superior performance compared to commonly used supervised learning methods, particularly when dealing with target texts that include unknown words and domain-specific phrases that were not encountered in the training corpus.

## Model Structure of HPO Linker

The HPO linker utilizes an embedding network, denoted as $G_{e}$ to transform a medical term or entity, represented by $t,$into a semantic vector $v_{t}$:

|  | $v_{t}=G_{e}\left( t,\boldsymbol{W}_{\boldsymbol{e}} \right)$ | (10) |
| --- | --- | --- |

where $W_{e}$ refers to the parameters of the network.

In this study, we adopted ALBERT^2^ as the embedding network, $G_{e}$. ALBERT is a lightweight version of the widely used NLP model BERT^3^, while still maintaining comparable performance. The architecture of the HPO linker, including the ALBERT model, is shown in **Supplementary Figure 1**.

## Implementation Details

The HPO Linker was implemented using the framework of Tensorflow, utilizing the ALBERT network pretrained on Chinese corpus (https://github.com/brightmart/albert_zh). The TopWORDS was implemented using C++ and Python (https://github.com/yuhuang-cst/topwords). The hyperparameters for the HPO linker were tuned using the EHRs from the PUMCH-S datasets. Specifically, $\tau$ was set to 2.0, $\eta$ = 0.85, the frequency threshold was set to 1.0, and the maximum length of a string was set to 12.

## Training Set of PBTagger

Due to the lack of a synonym database in the Chinese medical field similar to UMLS, the first step we took was to construct a Unified Chinese Medical Thesaurus. In the second step, we used this thesaurus, along with other medical datasets, to generate a large corpus of text for the training dataset.

First, we collected existing Chinese medical terminology resources, including CHPO, Chinese ICD-10, Chinese SNOMED-CT, and Chinese MeSH. We then used three machine translation engines—Google Translate, Baidu Translate, and Kingsoft PowerWord—to translate the English terms in UMLS into Chinese. By leveraging the Concept Unique Identifier (CUI) in UMLS, we linked terms representing the same concept, resulting in a Chinese unified medical vocabulary with a "concept-term" two-level structure. Given the numerous errors in machine translation, we implemented filtering rules during the construction process to improve the quality of the vocabulary.

Next, since we focused solely on the linkage of HPO phenotypes, we extracted a subset from the aforementioned Chinese Unified Medical Vocabulary related to HPO phenotypes, referred to as the "Chinese HPO Vocabulary." The "standard terms" in the Chinese HPO Vocabulary were manually translated from the CHPO, while the "synonyms" were sourced from other terminologies or machine translation. Using this Chinese vocabulary, we employed three methods to construct the training set for the phenotype linkage model.

The method details, along with all authorized raw data, are available on GitHub. Additionally, we have provided explanations for the source of each raw data set on GitHub. For example, AUIToCUI.json represents the mapping from each unique term (AUI) in UMLS to the corresponding concept (CUI), while HPOToSourceSynTerms.json contains Chinese synonyms for each standard HPO description, which were compiled by us. All the authorized data is also hosted on GitHub (https://github.com/xiaohaomao/timgroup_disease_diagnosis).

# Supplementary Note 3

## Implementation Details of Differential Diagnosis

Regarding implementation details, all prediction methods, except for BOQA and GDDP, were implemented using Python 3.6.12. The source codes for BOQA and GDDP were employed directly. The CNB and MLP models were implemented using the scikit-learn python API. The MLP model was trained using the Adam optimizer^4^ with the framework of Tensorflow. A website for PhenoBrain was constructed, using Flask as the back end, Vue.js as the front end, Lucene as the search engine, and MongoDB as the database.(<http://www.phenobrain.cs.tsinghua.edu.cn/pc>)

# Supplementary Data

## Simulated Datasets

Simulated datasets have been extensively used to develop and evaluate rare disease prediction methods to address insufficient datasets on rare diseases. The simulation is based on the observation that a case often presents symptoms that may reflect other underlying conditions unrelated to the rare disease. In addition, physicians may annotate symptoms that do not precisely match the standard HPO phenotypes, resulting in imprecise annotations. These two types of symptoms are referred to as “noise” (N) and “imprecise” phenotypes (I), respectively.

Based on these observations, four simulated datasets, named SIM, SIM (I), SIM (N), and SIM (I;N), were generated using a dataset of 44 complex dysmorphology syndromes. The generation process followed the same strategy as described in a prior work^5^.

Specifically, for a disease annotated with $n$ phenotypes, $t_{1},t_{2},\ldots,t_{n}$, with observed frequencies, $p_{1},p_{2},\ldots{,p}_{n}$, we assume these phenotypes conditionally independent, and use their observed frequencies to generate simulated cases.

For each disease in the 44 complex dysmorphology syndromes dataset, we generated 100 simulated cases, resulting in 4,400 cases in the “SIM” dataset. Then, we introduced 50% “noise” phenotypes to each case in the SIM dataset to establish the “SIM(N)” dataset. Similarly, we substituted 50% of the phenotypes in each case in the SIM dataset with their ancestor phenotypes to establish the “SIM(I)” dataset. Finally, we added 50% “noise” phenotypes to each case in the SIM(I) dataset, resulting in the “SIM (I; N)” dataset.

These simulated datasets provide a valuable resource for evaluating and benchmarking rare disease prediction methods under different scenarios, including the presence of noise and imprecise phenotypes. Four simulated data files have been uploaded to GitHub. (<https://github.com/xiaohaomao/timgroup_disease_diagnosis/data/simulated>)

## Public Test Sets

The Public Test sets include four public datasets mentioned in the main text, Table 1. For detailed information about each dataset, please refer to Table 1. All the Public Test sets have been consolidated into a PDF file named Supplementary Dataset 2 and uploaded to the NPJ website.

## EHR Test Sets

The EHR Test sets include two datasets from PUMCH mentioned in the main text, Table 1. For detailed information about each dataset, please refer to Table 1. All the EHR Test sets have been consolidated into an Excel file named Supplementary Dataset 1 and uploaded to the NPJ website

# Supplementary Discussion 1

## Comparison with Phenomizer and LIRICAL

To compare the performance of PhenoBrain with existing rare disease diagnostic tools, specifically Phenomizer (<http://compbio.charite.de/phenomizer/>) and LIRICAL (<https://github.com/TheJacksonLaboratory/LIRICAL>), we conducted a comparative analysis.

Phenomizer is a rare disease diagnostic tool that requires manual entry of HPO codes and provides prediction results. We utilized Phenomizer in our evaluation to assess its performance in comparison to PhenoBrain. To ensure a fair comparison, we implemented PhenoBrain and Phenomizer (version 2023.12.01) on the entire Public Test set.

LIRICAL, on the other hand, is a recent phenotype-based diagnostic tool for rare diseases. We implemented LIRICAL (version 2.0; 2023.06.02) on the entire Public Test Set to evaluate its performance alongside PhenoBrain and Phenomizer.

## Merged different rare disease knowledgebases

In this study, we merged the phenotype annotations of three disease knowledgebases, OMIM, Orphanet, and CCRD, to create an integrated knowledgebase. By mappings diseases between Orphanet and OMIM and between Orphanet and CCRD, we consolidated the disease codes that expressed the same concept and merged their phenotype annotations, including frequency information. Specifically, we directly utilized the phenotype annotations for diseases provided by the HPO team (the latest version released on October 9, 2023, available at <https://hpo>.jax.org/app/data/ontology). HPO annotations are provided in a qualitative manner for most phenotype frequencies. These frequency annotations are divided into six categories: obligatory, very frequent, frequent, occasional, very rare, or excluded ^6^. Although these annotations are qualitative, HPO provides reference ranges for each frequent type. In the latest version, “Obligate (100%)” indicates that the phenotype is almost always present in the context of the associated disease or gene mutation, with a frequency close to 100%. “Very frequent (80-99%)” signifies that the phenotype is highly common in the context of the related disease or gene mutation, occurring with a frequency between 80% and 99%. “Frequent (30-79%)” indicates that the phenotype is relatively common in the presence of the related disease or gene mutation, with a frequency ranging from 30% to 79%. “Occasional (5-29%)” suggests that the phenotype occasionally appears in the context of the related disease or gene mutation, occurring with a frequency between 5% and 29%. “Very rare (1-4%)” means that the phenotype is seldom seen in the context of the related disease or gene mutation, with a frequency between 1% and 4%.

To merge knowledgebases, we now use the original qualitative descriptions of phenotype annotations for each disease from different knowledgebases. When the same phenotype for a disease has different definitions or annotations in different knowledgebases, we save both definitions or annotations: for example, “frequent” from OMIM and “occasional” from Orphanet. In fact, this situation is quite rare, as most disease phenotypes are annotated by only one knowledgebase.

The Integrated knowledgebase provides a unique disease code for each rare disease, with the prefix“"RD”". For example, the idiopathic pulmonary fibrosis diseases were present in OMIM, Orphanet, and CCRD knowledgebase. By merging these sources, we assigned a new disease code with the prefix“"RD”" to represent idiopathic pulmonary fibrosis in the integrated knowledgebase.

## Prompt Example

**Instruction:** As a doctor, please provide answers and recommendations. The information within each set of brackets [ ] represents a patien’'s medical record in the xx department. Please provide the ten most probable and self-consistency English diagnoses for rare diseases, including their OMIM and ORPHANET codes.

**In-context:** [ The patient experienced limb pain around 2001, without any apparent triggers. The pain mainly affected the palms and soles and worsened during hot weather, episodes of fever due to colds, and after physical activity. Resting provided relief from the symptoms, which were described as burning and distending, without notable numbness or reduced sensation in the hands and feet. Additionally, the patient had scattered pinpoint-sized, purplish-red, non-raised skin rashes on the lower back, inner thighs, with no tenderness or itching. About one year ago, the patient had two episodes of dizziness characterized by rotational visual sensations, occasionally accompanied by nausea and vomiting, but no tinnitus. They sought medical attention at a Beijing hospital and were diagnosed with“"benign paroxysmal positional vertigo”" improving after repositioning treatment. Over the past six months, the patient occasionally experienced chest tightness, shortness of breath, and intermittent punctate needle-like pain in the anterior chest area. These symptoms were not significantly related to posture or physical activity and typically subsided after resting for approximately 10-20 minutes. The patient had not taken any nitrate medications. The limb pain did not worsen significantly, with occasional electric shock-like sensations, but no obvious numbness. One week ago, the dizziness symptoms recurred, and despite seeking medical attention at an external hospital, a definitive diagnosis was not given. They have been referred to our department for further evaluation. Over the past six months, the patien’'s mental state, appetite, sleep, and urination have been normal. However, over the past two months, there have been changes in bowel movements, with 2-3 times daily of loose, mushy stools of unspecified color. There was no significant weight loss.

Current Diagnosis: vestibular microcirculation disorder not excluded; hearing impairment; myocardial involvement; hypertension (Grade II, moderate-risk group) with narrowing of the right posterior cerebral artery; glaucoma (right eye, not excluded); and facial melanocytic nevus.]

Diagnoses: 1, Fabry Disease, OMIM:301500, ORPHA:324

2, Gitelman Syndrome, OMIM:263800, ORPHA:358

3, …

# Supplementary Figure 1


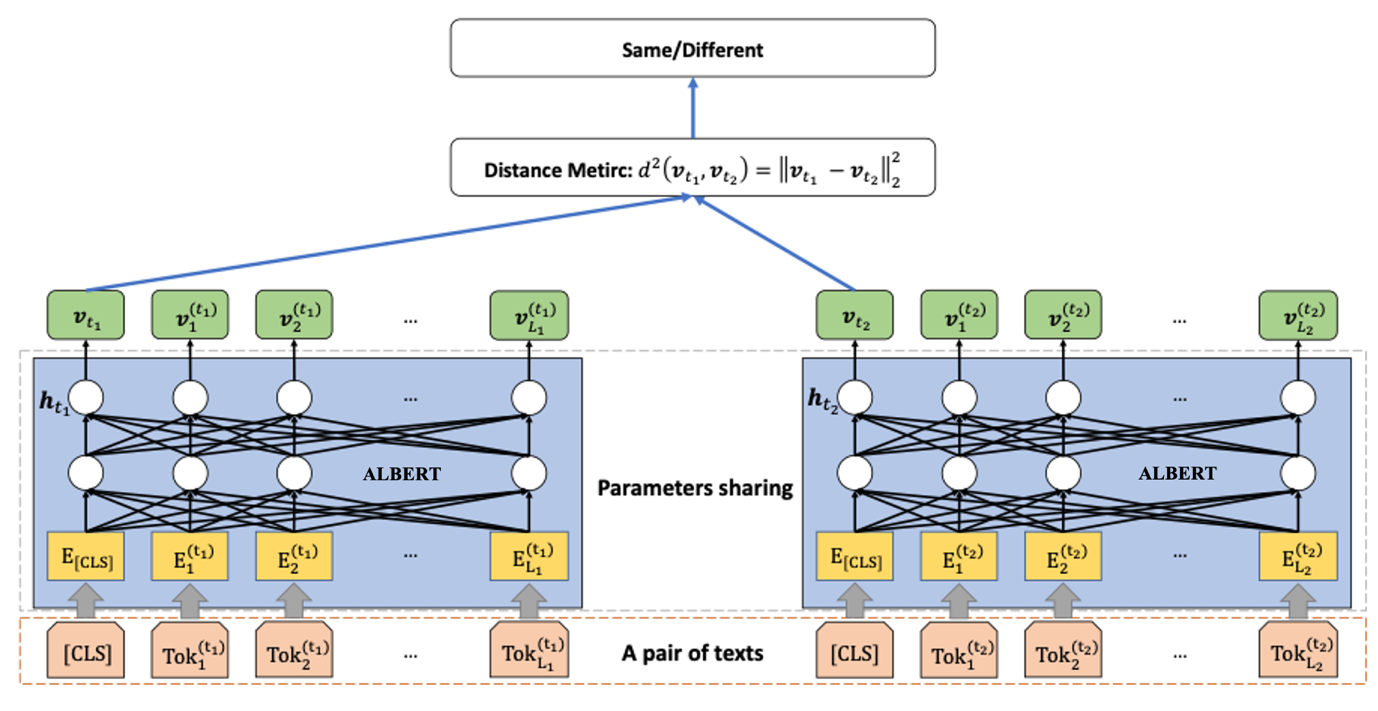


**Supplementary Figure 1. Structure of the HPO linker.** The model receives a pair of medical texts and returns a matching score indicating whether they are the same. Using a pair of texts, the Siamese network with shared parameters based on the ALBERT^2^ model generates two semantic vectors. Training is conducted based on the Euclidean distance between these vectors and the DDML^7^ loss function.

# Supplementary Tables

## Supplementary Table 1. Characteristics of rare disease knowledgebases

| **Knowledgebases** | **Number of Diseases** | **Number of HPO**  **terms (Dedup*)** | **Number of HPO terms per Disease (Min/Median/Max)** | **Number of Annotations with Frequency/**  **Number of Annotations** |
| --- | --- | --- | --- | --- |
| OMIM | 7623 | 7256 | 1 /9.0/ 127 | 10402 / 101127 |
| ORPHA | 3771 | 6595 | 1 / 18.0 / 172 | 79567 / 82388 |
| CCRD | 144 | 1625 | 3 / 23.5 / 66 | 0 / 3663 |
| Integrated (OMIM+ORPHA) | 9225 | 8856 | 1 / 12.0 / 216 | 88083 / 166065 |
| Integrated (OMIM+ORPHA+CCRD) | 9260 | 8930 | 1 / 13.0 / 250 | 88083 / 168780 |

*Dedup: Deduplication. Only the most specific HPO terms for each disease are counted.

###

## Supplementary Table 2. Ratios of case phenotypes matched with disease phenotypes in rare disease datasets

| **Datasets** | **Exact Match*** | **General*** | **Specific*** | **Unmatched*** |
| --- | --- | --- | --- | --- |
| RAMEDIS | 24.6% | 7.2% | 3.0% | 65.2% |
| MME | 51.0% | 7.1% | 6.5% | 35.4% |
| HMS | 21.8% | 8.5% | 22.3% | 47.4% |
| LIRICAL | 23.2% | 6.3% | 5.1% | 65.4% |
| PUMCH-S | 23.0% | 4.7% | 7.0% | 65.3% |
| PUMCH-L | 16.5% | 9.4% | 11.5% | 62.6% |
| PUMCH-ADM | 18.7% | 4.5% | 13.3% | 63.5% |

* "Exact match": case phenotypes match the disease phenotypes annotated in the knowledgebase. "General" and "Specific": case phenotypes are ancestors and descendants, respectively, of the disease phenotypes in the knowledgebase. "Unmatched": case phenotypes are unrelated to the diagnosed rare diseases in the knowledgebase.

## Supplementary Table 3. Top 5 diseases in rare disease datasets

| **Disease names** | **Disease codes** | **Number of cases** |
| --- | --- | --- |
| **RAMEDIS** | | |
| Phenylketonuria | CCRD:90, OMIM:261600, ORPHA:716 | 148 |
| Glutaric acidemia type 1 | CCRD:34.1, OMIM:231670, ORPHA:25 | 39 |
| Medium chain acyl-CoA dehydrogenase deficiency | CCRD:70, OMIM:201450, ORPHA:42 | 36 |
| Methylmalonic aciduria due to methylmalonyl-CoA mutase deficiency | CCRD:71, OMIM:251000, ORPHA:27 | 32 |
| Biotinidase deficiency | CCRD:13, OMIM:253260, ORPHA:79241 | 26 |
| **MME** | | |
| Cerebrocostomandibular syndrome | OMIM:117650, ORPHA:1393 | 11 |
| Congenital disorder of deglycosylation | OMIM:615273, ORPHA:404454 | 8 |
| Poretti-boltshauser syndrome | OMIM:615960, ORPHA:370022 | 6 |
| Spondylometaphyseal dysplasia, sedaghatian type | OMIM:250220, ORPHA:93317 | 2 |
| Mandibulofacial dysostosis, guion-almeida type | OMIM:610536, ORPHA:79113 | 1 |
| **LIRICAL** | | |
| TBCK-related intellectual disability syndrome | OMIM:616900, ORPHA:488632 | 19 |
| Neurodevelopmental disorder with or without anomalies of the brain, eye or heart | OMIM:616975 | 18 |
| Autosomal recessive spastic paraplegia type 76 | OMIM:616907, ORPHA:488594 | 12 |
| Immunoskeletal dysplasia with neurodevelopmental abnormalities | OMIM:617425 | 10 |
| Ataxia-pancytopenia syndrome | OMIM:159550, ORPHA:2585 | 8 |
| **HMS** | | |
| Granulomatosis with polyangiitis | OMIM:608710, ORPHA:900 | 11 |
| Antisynthetase syndrome | ORPHA:81 | 5 |
| Sarcoidosis | OMIM:181000, OMIM:612387, ORPHA:797 | 4 |
| Takayasu arteritis | OMIM:207600, ORPHA:3287 | 4 |
| Cryoglobulinemic vasculitis | OMIM:123550, ORPHA:91138 | 4 |
| **PUMCH-S** | | |
| Eosinophilic granulomatosis with polyangiitis | ORPHA:183 | 5 |
| Cushing syndrome | OMIM:219080, OMIM:219090, OMIM:610475, OMIM:610489, OMIM:614190, OMIM:615830, OMIM:615954, ORPHA:189427, ORPHA:553, ORPHA:96253, ORPHA:99889, ORPHA:99892, ORPHA:99893 | 4 |
| Primary pulmonary hypertension | CCRD:54, OMIM:178600, ORPHA:422 | 4 |
| POEMS syndrome | CCRD:91, ORPHA:2905 | 3 |
| Cronkhite-Canada syndrome | OMIM:175500, ORPHA:2930 | 3 |
| **PUMCH-L** | | |
| Cushing syndrome | OMIM:219080, OMIM:219090, OMIM:610475, OMIM:610489, OMIM:614190, OMIM:615830, OMIM:615954, ORPHA:189427, ORPHA:553, ORPHA:96253, ORPHA:99889, ORPHA:99892, ORPHA:99893 | 200 |
| POEMS syndrome | CCRD:91, ORPHA:2905 | 164 |
| Carcinoid tumors, intestinal | OMIM:114900, ORPHA:100093 | 60 |
| Eosinophilic granulomatosis with polyangiitis | ORPHA:183 | 53 |
| Generalized myasthenia gravis; GMG | CCRD:32, OMIM:254200, ORPHA:589 | 45 |
| **PUMCH-ADM** | | |
| Alport syndrome | CCRD:3, OMIM:104200, OMIM:203780, OMIM:301050, ORPHA:63 | 8 |
| Prader-Willi syndrome | CCRD:93, OMIM:176270, ORPHA:739 | 5 |
| Hepatolenticular degeneration; Wilson disease | CCRD:37, OMIM:277900, ORPHA:905 | 5 |
| McCune-Albright syndrome | CCRD:69, OMIM:174800, ORPHA:562 | 5 |
| Marfan syndrome; MFS | CCRD:68, OMIM:154700, ORPHA:284979, ORPHA:558 | 5 |

## Supplementary Table 4. Disease Names and Number of Cases for two PUMCH Datasets

| **Disease Name** | **Number of Cases** |
| --- | --- |
| **PUMCH-ADM** | 75 |
| Prader-Willi Syndrome | 5 |
| Hepatolenticular Degeneration; Wilson Disease | 5 |
| McCune-Albright Syndrome | 5 |
| Marfan Syndrome | 5 |
| Arrhythmogenic Right Ventricular Cardiomyopathy | 4 |
| Brugada Syndrome | 3 |
| Restrictive Cardiomyopathy | 3 |
| Amyotrophic Lateral Sclerosis | 5 |
| Generalized Myasthenia Gravis | 5 |
| Multiple System Atrophy | 5 |
| Alport Syndrome | 8 |
| Fabry Disease | 3 |
| Gitelman Syndrome | 4 |
| Paroxysmal Nocturnal Hemoglobinuria | 5 |
| POEMS Syndrome | 5 |
| Niemann-Pick Disease | 5 |
|  |  |
| **MDT cases of PUMCH-L** | 27 |
| Oblique Vaginal Septum Syndrome | 1 |
| Familial Hypercholesterolemia | 2 |
| Crest Syndrome | 1 |
| Homozygous Familial Hypercholesterolemia | 1 |
| Proteasome-Associated Autoinflammatory Syndromes 2 | 1 |
| Primary Angiitis of The Central Nervous System | 1 |
| Mucopolysaccharidosis Type II | 1 |
| Gaucher Disease | 2 |
| Takayasu Arteritis | 1 |
| Aicardi-Goutieres Syndrome | 1 |
| Sideroblastic Anemia with B Cell Immunodeficiency, Periodic Fevers, and Developmental Delay | 1 |
| CLOVES Syndrome | 1 |
| Autosomal Recessive Myogenic Arthrogryposis Multiplex Congenita | 1 |
| McCune-Albright Syndrome | 1 |
| Adrenocortical Carcinoma | 1 |
| Fabry Disease | 1 |
| Porphyria | 1 |
| Waardenburg Syndrome | 1 |
| Craniotelencephalic Dysplasia | 1 |
| Tuberous Sclerosis | 1 |
| Primary Hypertrophic Osteoarthropathy | 1 |
| Spinal Muscular Atrophy | 2 |
| Folliculotropic Mycosis Fungoides | 2 |

## Supplementary Table 5. Performance of 17 diagnostic methods on the Public Test Set (873 cases)

| **Methods** | **Recall@10 (95% CI)** | **Recall@3 (95% CI)** | **Recall@1 (95% CI)** | **Median Rank** |
| --- | --- | --- | --- | --- |
| Res^8^ | 0.472(0.439 to 0.505) | 0.328(0.297 to 0.359) | 0.223(0.196 to 0.251) | 12.0 |
| BOQA^9^ | 0.457(0.425 to 0.490) | 0.347(0.316 to 0.379) | 0.234(0.206 to 0.262) | 15.0 |
| RDD^10^ | 0.312(0.282 to 0.342) | 0.190(0.165 to 0.216) | 0.117(0.096 to 0.140) | 31.0 |
| GDDP^11^ | 0.534(0.499 to 0.566) | 0.315(0.284 to 0.346) | 0.196(0.170 to 0.222) | 9.0 |
| RBP^12^ | 0.591(0.558 to 0.624) | 0.410(0.378 to 0.443) | 0.242(0.214 to 0.270) | 6.0 |
| MinIC^13^ | 0.593(0.561 to 0.625) | 0.424(0.391 to 0.457) | 0.257(0.229 to 0.286) | 6.0 |
| Lin^14^ | 0.432(0.399 to 0.465) | 0.291(0.261 to 0.322) | 0.188(0.163 to 0.214) | 17.0 |
| JC^15^ | 0.513(0.480 to 0.546) | 0.373(0.341 to 0.405) | 0.250(0.221 to 0.279) | 9.0 |
| SimGIC^16^ | 0.294(0.263 to 0.325) | 0.187(0.160 to 0.213) | 0.124(0.102 to 0.145) | 43.0 |
| SimUI^17^ | 0.213(0.186 to 0.241) | 0.147(0.123 to 0.171) | 0.092(0.073 to 0.111) | 144.0 |
| TO^18^ | 0.510(0.477 to 0.542) | 0.338(0.307 to 0.369) | 0.197(0.171 to 0.223) | 10.0 |
| Cosine | 0.259(0.231 to 0.288) | 0.171(0.147 to 0.196) | 0.112(0.092 to 0.134) | 67.0 |
| Best* | 0.593(0.561 to 0.625) | 0.424(0.391 to 0.457) | 0.257(0.229 to 0.286) | 6.0 |
|  |  |  |  |  |
| ICTO | 0.625(0.593 to 0.658) | 0.431(0.397 to 0.463) | 0.275(0.245 to 0.305) | 5.0 |
| PP0 | 0.613(0.581 to 0.645) | 0.467(0.434 to 0.501) | 0.296(0.266 to 0.326) | 4.0 |
| CNB | 0.612(0.578 to 0.644) | 0.432(0.400 to 0.464) | 0.260(0.231 to 0.290) | 5.0 |
| MLP | 0.606(0.574 to 0.638) | 0.384(0.353 to 0.416) | 0.250(0.222 to 0.278) | 6.0 |
| Ensemble | 0.640(0.608 to 0.672) | 0.483(0.450 to 0.517) | 0.304(0.273 to 0.334) | 4.0 |

*Best results among the benchmarks.

## Supplementary Table 6. Performance of Phenomizer and 5 proposed methods on Public Test Set* (753 cases)

| **Methods** | **Recall@10 (95% CI)** | **Recall@3 (95% CI)** | **Recall@1 (95% CI)** | **Median Rank** |
| --- | --- | --- | --- | --- |
| Phenomizer | 0.434(0.398-0.470) | 0.256(0.226-0.288) | 0.114(0.092-0.137) | 15.0 |
|  |  |  |  |  |
| ICTO | 0.632(0.598-0.667) | 0.425(0.389-0.461) | 0.258(0.226-0.290) | 5.0 |
| PP0 | 0.623(0.588-0.657) | 0.470(0.434-0.506) | 0.286(0.254-0.317) | 4.0 |
| CNB | 0.618(0.583-0.652) | 0.429(0.393-0.465) | 0.246(0.215-0.276) | 5.0 |
| MLP | 0.603(0.567-0.637) | 0.373(0.339-0.408) | 0.235(0.205-0.266) | 6.0 |
| Ensemble | 0.651(0.616-0.685) | 0.486(0.450-0.522) | 0.295(0.263-0.328) | 4.0 |

*Phenomizer makes differential diagnosis among 8,012 rare diseases. Among the 873 cases in the Public Test Set, 120 have disease codes outside the 8,012 diseases. Excluding these cases resulted in 753 cases.

## Supplementary Table 7. Performance of LIRICAL tool and 5 proposed methods on Public Test Set* (850 cases)

| **Methods** | **Recall@10 (95% CI)** | **Recall@3 (95% CI)** | **Recall@1 (95% CI)** | **Median Rank** |
| --- | --- | --- | --- | --- |
| LIRICAL | 0.560 (0.526 to 0.593) | 0.407(0.374 to 0.440) | 0.232(0.204 to 0.260) | 6.0 |
|  |  |  |  |  |
| ICTO | 0.633(0.601 to 0.665) | 0.439(0.406 to 0.473) | 0.281(0.252 to 0.312) | 5.0 |
| PPO | 0.621(0.588 to 0.653) | 0.475(0.442 to 0.508) | 0.302(0.271 to 0.334) | 4.0 |
| CNB | 0.624(0.591 to 0.655) | 0.442(0.408 to 0.476) | 0.266(0.235 to 0.296) | 5.0 |
| MLP | 0.615(0.582 to 0.648) | 0.393(0.360 to 0.426) | 0.256(0.228 to 0.286) | 6.0 |
| Ensemble | 0.648(0.616 to 0.680) | 0.493(0.459 to 0.527) | 0.312(0.281 to 0.342) | 4.0 |

*LIRICAL makes differential diagnosis among 8,167 rare diseases. Among the 873 cases in the Public Test Set, 23 have disease codes outside the 8,167 diseases. Excluding these cases resulted in 850 cases.

## Supplementary Table 8. Performance of PhenoBrain and various methods of Phen2Disease on Cohort 1* (384 cases)

|  | **MODELS** | **Num_top1** | **Num_top5** | **Num_top10** | **Num_top20** | **Num_top50** | **Num_top100** |
| --- | --- | --- | --- | --- | --- | --- | --- |
| Our methods | ICTO | 127 | 198 | 233 | 254 | 301 | 323 |
|  | PPO | 128 | 195 | 224 | 244 | 275 | 302 |
|  | CNB | 122 | 193 | 227 | 255 | 292 | 325 |
|  | MLP | 113 | 179 | 230 | 257 | 287 | 317 |
|  | PhenoBrain (Ensemble method) | 131 | 202 | 237 | 256 | 299 | 334 |
|  |  |  |  |  |  |  |  |
|  | Phen2Disease | 111 | 192 | 221 | 249 | 291 | 315 |
|  | BASE_IC | 98 | 176 | 202 | 227 | 266 | 304 |
|  | Phen2Disease_patient | 96 | 162 | 184 | 218 | 260 | 285 |
| Methods in Phen2Disease | LIRICAL | 94 | 153 | 187 | 222 | 264 | 304 |
|  | Phrank | 83 | 153 | 178 | 222 | 261 | 286 |
|  | PhenoApt | 83 | 140 | 167 | 195 | 250 | 277 |
|  | Phen2Disease_double | 81 | 138 | 159 | 198 | 244 | 278 |

* Cohort 1, comprising 384 cases representing 262 rare diseases, was utilized to study Phen2Disease. Phen2Disease was compared with baseline methods, namely BASE_IC, LRICAL, Phrank, and PhenoApt, using cohort 1 as the evaluation dataset. The evaluation metric 'Num_topk' denotes the number of cases in which the correct diagnoses were included among the top k predictions..

## Supplementary Table 9. Median ranks of 17 diagnostic methods on test subsets of PUMCH-S (34 cases)using various medical text processing tools

| **Methods** | **Physicians** | **CHPO** | **UMLS** | **PBTagger** |
| --- | --- | --- | --- | --- |
| Res | 5.0 | 12.0 | 10.5 | 9.0 |
| BOQA | 6.0 | 31.0 | 17.5 | 10.0 |
| RDD | 12.0 | 32.0 | 29.5 | 46.0 |
| GDDP | 4.5 | 17.0 | 9.0 | 9.5 |
| RBP | 4.0 | 21.0 | 7.0 | 6.0 |
| MinIC | 3.5 | 12.5 | 9.0 | 7.5 |
| Lin | 5.5 | 21.5 | 26.0 | 15.0 |
| JC | 4.0 | 13.5 | 10.5 | 7.0 |
| SimGIC | 11.5 | 80.0 | 19.0 | 15.5 |
| SimUI | 17.5 | 105.5 | 35.5 | 35.5 |
| TO | 7.0 | 19.0 | 14.0 | 12.0 |
| Cosine | 12.0 | 67.0 | 19.0 | 25.0 |
| Best* | 3.5 | 12.0 | 7.0 | 6.0 |
|  |  |  |  |  |
| ICTO | 3.0 | 13.5 | 7.0 | 3.5 |
| PPO | 3.0 | 12.5 | 6.5 | 5.5 |
| CNB | 3.0 | 20.0 | 6.5 | 5.5 |
| MLP | 3.0 | 11.5 | 8.0 | 4.0 |
| Ensemble | 2.0 | 10.5 | 6.0 | 3.5 |

*Best results among the 12 benchmarks.

## Supplementary Table 10. Performance of 17 diagnostic methods on test subsets of PUMCH-S (34 cases) using PBTagger

| **Methods** | **Recall@10 (95% CI)** | **Recall@3 (95% CI)** | **Recall@1 (95% CI)** | **Median Rank** |
| --- | --- | --- | --- | --- |
| Res | 0.500(0.365 to 0.635) | 0.365(0.231 to 0.500) | 0.154(0.058 to 0.250) | 9.0 |
| BOQA | 0.519(0.385 to 0.654) | 0.404(0.269 to 0.538) | 0.212(0.115 to 0.327) | 10.0 |
| RDD | 0.250(0.135 to 0.365) | 0.096(0.019 to 0.173) | 0.077(0.019 to 0.154) | 46.0 |
| GDDP | 0.577(0.442 to 0.712) | 0.269(0.154 to 0.404) | 0.135(0.058 to 0.231) | 9.5 |
| RBP | 0.558(0.423 to 0.692) | 0.346(0.212 to 0.481) | 0.154(0.058 to 0.250) | 6.0 |
| MinIC | 0.635(0.500 to 0.769) | 0.365(0.231 to 0.500) | 0.250(0.135 to 0.365) | 7.5 |
| Lin | 0.442(0.308 to 0.577) | 0.288(0.173 to 0.423) | 0.192(0.096 to 0.308) | 15.0 |
| JC | 0.577(0.442 to 0.712) | 0.404(0.269 to 0.538) | 0.173(0.077 to 0.288) | 7.0 |
| SimGIC | 0.442(0.308 to 0.577) | 0.212(0.115 to 0.327) | 0.115(0.038 to 0.212) | 15.5 |
| SimUI | 0.385(0.250 to 0.519) | 0.212(0.115 to 0.327) | 0.135(0.058 to 0.231) | 35.5 |
| TO | 0.462(0.327 to 0.596) | 0.288(0.173 to 0.404) | 0.115(0.038 to 0.212) | 12.0 |
| Cosine | 0.423(0.288 to 0.558) | 0.269(0.154 to 0.385) | 0.135(0.058 to 0.231) | 25.0 |
| Best* | 0.635(0.500 to 0.769) | 0.404(0.269 to 0.538) | 0.250(0.135 to 0.365) | 6.0 |
|  |  |  |  |  |
| ICTO | 0.654(0.519 to 0.788) | 0.500(0.365 to 0.635) | 0.288(0.173 to 0.404) | 3.5 |
| PPO | 0.692(0.558 to 0.808) | 0.423(0.288 to 0.558) | 0.231(0.115 to 0.346) | 5.5 |
| CNB | 0.596(0.462 to 0.731) | 0.365(0.231 to 0.500) | 0.231(0.115 to 0.346) | 5.5 |
| MLP | 0.615(0.481 to 0.750) | 0.404(0.269 to 0.538) | 0.269(0.154 to 0.385) | 4.0 |
| Ensemble | 0.673(0.538 to 0.788) | 0.500(0.365 to 0.635) | 0.308(0.192 to 0.442) | 3.5 |

*Best results among the benchmarks.

## Supplementary Table 11. Median ranks of 17 diagnostic methods on PUMCH-L using various medical text processing tools

| **Methods** | **CHPO** | **UMLS** | **PBTagger** |
| --- | --- | --- | --- |
| Res | 11.0 | 11.0 | 9.0 |
| BOQA | 16.0 | 15.0 | 8.0 |
| RDD | 20.0 | 24.5 | 35.5 |
| GDDP | 28.0 | 15.0 | 9.0 |
| RBP | 20.5 | 18.0 | 12.5 |
| MinIC | 10.0 | 9.0 | 6.0 |
| Lin | 13.5 | 14.0 | 15.0 |
| JC | 13.5 | 13.0 | 8.0 |
| SimGIC | 34.5 | 16.0 | 6.0 |
| SimUI | 53.5 | 24.0 | 8.0 |
| TO | 16.0 | 17.0 | 21.0 |
| Cosine | 33.0 | 18.0 | 7.0 |
| Best* | 10.0 | 9.0 | 6.0 |
|  |  |  |  |
| ICTO | 12.0 | 10.0 | 5.0 |
| PPO | 10.0 | 12.0 | 7.5 |
| CNB | 12.0 | 9.0 | 5.0 |
| MLP | 12.0 | 9.0 | 5.0 |
| Ensemble | 10.5 | 8.0 | 4.0 |

*Best results among all benchmarks.

## Supplementary Table 12. Performance of 17 diagnostic methods on PUMCH-L

| **Methods** | **Recall@10 (95% CI)** | **Recall@3 (95% CI)** | **Recall@1 (95% CI)** | **Median Rank** |
| --- | --- | --- | --- | --- |
| Res | 0.524(0.493 to 0.556) | 0.348(0.319 to 0.378) | 0.217(0.191 to 0.242) | 9.0 |
| BOQA | 0.535(0.504 to 0.567) | 0.384(0.353 to 0.415) | 0.256(0.228 to 0.284) | 8.0 |
| RDD | 0.370(0.339 to 0.401) | 0.230(0.203 to 0.256) | 0.155(0.133 to 0.178) | 35.5 |
| GDDP | 0.525(0.494 to 0.557) | 0.342(0.313 to 0.371) | 0.200(0.176 to 0.225) | 9.0 |
| RBP | 0.469(0.437 to 0.500) | 0.308(0.279 to 0.336) | 0.172(0.149 to 0.196) | 12.5 |
| MinIC | 0.582(0.552 to 0.613) | 0.406(0.376 to 0.436) | 0.272(0.244 to 0.301) | 6.0 |
| Lin | 0.435(0.405 to 0.467) | 0.275(0.247 to 0.304) | 0.138(0.116 to 0.160) | 15.0 |
| JC | 0.535(0.504 to 0.566) | 0.381(0.351 to 0.411) | 0.243(0.217 to 0.270) | 8.0 |
| SimGIC | 0.585(0.554 to 0.615) | 0.424(0.394 to 0.454) | 0.294(0.265 to 0.322) | 6.0 |
| SimUI | 0.547(0.515 to 0.576) | 0.402(0.371 to 0.432) | 0.287(0.260 to 0.316) | 8.0 |
| TO | 0.413(0.382 to 0.443) | 0.232(0.205 to 0.258) | 0.128(0.107 to 0.149) | 21.0 |
| Cosine | 0.546(0.515 to 0.577) | 0.401(0.370 to 0.431) | 0.270(0.242 to 0.299) | 7.0 |
| Best* | 0.585(0.554 to 0.615) | 0.424(0.394 to 0.454) | 0.294(0.265 to 0.322) | 6.0 |
|  |  |  |  |  |
| ICTO | 0.613(0.582 to 0.644) | 0.445(0.415 to 0.477) | 0.288(0.261 to 0.317) | 5.0 |
| PPO | 0.565(0.534 to 0.596) | 0.382(0.351 to 0.413) | 0.246(0.220 to 0.273) | 7.5 |
| CNB | 0.601(0.571 to 0.632) | 0.428(0.397 to 0.459) | 0.293(0.264 to 0.321) | 5.0 |
| MLP | 0.594(0.564 to 0.624) | 0.431(0.401 to 0.462) | 0.296(0.268 to 0.324) | 5.0 |
| Ensemble | 0.630(0.599 to 0.660) | 0.468(0.436 to 0.499) | 0.315(0.286 to 0.344) | 4.0 |

*Best results among all benchmarks.

## Supplementary Table 13. Difference of median ranks using various medical text processing methods*

| **Dataset** | **Methods** | **CHPO vs.**  **PBTagger*** | **UMLS vs.**  **PBTagger** | **Human vs.**  **PBTagger** |
| --- | --- | --- | --- | --- |
|  | Res | 3.0 | 1.5 | -4.0 |
|  | BOQA | 21.0 | 7.5 | -4.0 |
|  | RDD | -14.0 | -16.5 | -34.0 |
|  | GDDP | 7.5 | -0.5 | -5.0 |
|  | RBP | 15.0 | 1.0 | -2.0 |
|  | MinIC | 5.0 | 1.5 | -4.0 |
|  | Lin | 6.5 | 11 | -9.5 |
|  | JC | 6.5 | 3.5 | -3.0 |
| **PUMCH-S** | SimGIC | 64.5 | 3.5 | -4.0 |
|  | SimUI | 70.0 | 0.0 | -18.0 |
|  | TO | 7.0 | 2.0 | -5.0 |
|  | Cosine | 42.0 | -6.0 | -13.0 |
|  | ICTO | 10.0 | 3.5 | -0.5 |
|  | PPO | 7.0 | 1.0 | -2.5 |
|  | CNB | 14.5 | 1.0 | -2.5 |
|  | MLP | 7.5 | 4.0 | -1.0 |
|  | Ensemble | 7.0 | 2.5 | -1.5 |
|  | Res | 2.0 | 2.0 |  |
|  | BOQA | 8.0 | 7.0 |  |
|  | RDD | -15.5 | -11.0 |  |
|  | GDDP | 19.0 | 6.0 |  |
|  | RBP | 8.0 | 5.5 |  |
|  | MinIC | 4.0 | 3.0 |  |
|  | Lin | -1.5 | -1.0 |  |
|  | JC | 5.5 | 5.0 |  |
| **PUMCH-L** | SimGIC | 28.5 | 10.0 |  |
|  | SimUI | 45.5 | 16.0 |  |
|  | TO | -5.0 | -4.0 |  |
|  | Cosine | 26.0 | 11.0 |  |
|  | ICTO | 7.0 | 5.0 |  |
|  | PPO | 2.5 | 4.5 |  |
|  | CNB | 7.0 | 4.0 |  |
|  | MLP | 7.0 | 4.0 |  |
|  | Ensemble | 6.5 | 4.0 |  |

*Positive difference represents better performance by PBTagger.

## Supplementary Table 14. Performance of 17 diagnostic methods on PUMCH-ADM and full 9260 rare diseases

| **Methods** | **Recall@10 (95% CI)** | **Recall@3 (95% CI)** | **Recall@1 (95% CI)** | **Median Rank** |
| --- | --- | --- | --- | --- |
| Res | 0.573(0.467 to 0.680) | 0.427(0.320 to 0.533) | 0.307(0.200 to 0.413) | 6.0 |
| BOQA | 0.533(0.413 to 0.653) | 0.373(0.267 to 0.480) | 0.200(0.107 to 0.293) | 10.0 |
| RDD | 0.320(0.227 to 0.427) | 0.173(0.093 to 0.267) | 0.080(0.027 to 0.147) | 27.0 |
| GDDP | 0.627(0.520 to 0.733) | 0.413(0.307 to 0.520) | 0.240(0.147 to 0.333) | 6.0 |
| RBP | 0.627(0.520 to 0.733) | 0.427(0.320 to 0.547) | 0.253(0.160 to 0.360) | 5.0 |
| MinIC | 0.680(0.573 to 0.787) | 0.493(0.387 to 0.613) | 0.253(0.160 to 0.360) | 4.0 |
| Lin | 0.560(0.440 to 0.680) | 0.453(0.347 to 0.573) | 0.267(0.173 to 0.373) | 6.0 |
| JC | 0.653(0.547 to 0.760) | 0.467(0.360 to 0.573) | 0.307(0.200 to 0.413) | 4.0 |
| SimGIC | 0.253(0.160 to 0.360) | 0.147(0.067 to 0.227) | 0.067(0.013 to 0.133) | 29.0 |
| SimUI | 0.213(0.120 to 0.307) | 0.120(0.053 to 0.200) | 0.067(0.013 to 0.120) | 64.0 |
| TO | 0.533(0.413 to 0.640) | 0.400(0.293 to 0.507) | 0.240(0.147 to 0.333) | 7.0 |
| Cosine | 0.253(0.160 to 0.360) | 0.160(0.080 to 0.240) | 0.067(0.013 to 0.133) | 39.0 |
| Best* | 0.680(0.573 to 0.787) | 0.493(0.387 to 0.613) | 0.307(0.200 to 0.413) | 4.0 |
|  |  |  |  |  |
| ICTO | 0.693(0.587 to 0.800) | 0.520(0.413 to 0.640) | 0.347(0.240 to 0.453) | 3.0 |
| PPO | 0.680(0.573 to 0.787) | 0.560(0.440 to 0.667) | 0.427(0.320 to 0.533) | 2.0 |
| CNB | 0.667(0.560 to 0.773) | 0.467(0.360 to 0.587) | 0.320(0.213 to 0.427) | 5.0 |
| MLP | 0.653(0.547 to 0.760) | 0.480(0.360 to 0.600) | 0.307(0.200 to 0.413) | 4.0 |
| Ensemble | 0.693(0.587 to 0.800) | 0.587(0.480 to 0.693) | 0.453(0.347 to 0.573) | 2.0 |

*Best results among all benchmarks.

## Supplementary Table 15. Average Performance of 17 diagnostic methods on Public Test Set, PUMCH-L and PUMCH-ADM

| **Methods** | **Recall@10 (95% CI)** | **Recall@3 (95% CI)** | **Recall@1 (95% CI)** | **Median Rank** |
| --- | --- | --- | --- | --- |
| Res | 0.523(0.482 to 0.564) | 0.367(0.328 to 0.408) | 0.249(0.213 to 0.287) | 9.0 |
| BOQA | 0.509(0.468 to 0.549) | 0.368(0.329 to 0.408) | 0.230(0.198 to 0.264) | 11.0 |
| RDD | 0.334(0.296 to 0.373) | 0.198(0.168 to 0.229) | 0.117(0.096 to 0.141) | 31.2 |
| GDDP | 0.562(0.523 to 0.601) | 0.357(0.319 to 0.396) | 0.212(0.180 to 0.247) | 8.0 |
| RBP | 0.562(0.522 to 0.601) | 0.381(0.342 to 0.421) | 0.222(0.188 to 0.258) | 7.8 |
| MinIC | 0.618(0.580 to 0.656) | 0.441(0.400 to 0.480) | 0.261(0.226 to 0.297) | 5.3 |
| Lin | 0.476(0.436 to 0.516) | 0.340(0.300 to 0.380) | 0.197(0.163 to 0.233) | 12.7 |
| JC | 0.567(0.527 to 0.606) | 0.407(0.366 to 0.447) | 0.266(0.230 to 0.305) | 7.0 |
| SimGIC | 0.378(0.343 to 0.415) | 0.252(0.224 to 0.283) | 0.161(0.141 to 0.186) | 26.0 |
| SimUI | 0.324(0.292 to 0.359) | 0.223(0.196 to 0.252) | 0.149(0.128 to 0.171) | 72.0 |
| TO | 0.485(0.445 to 0.525) | 0.323(0.284 to 0.362) | 0.188(0.155 to 0.223) | 12.7 |
| Cosine | 0.353(0.318 to 0.389) | 0.244(0.215 to 0.276) | 0.150(0.129 to 0.174) | 37.7 |
| Best* | 0.618(0.580 to 0.656) | 0.441(0.400 to 0.480) | 0.266(0.230 to 0.305) | 5.3 |
|  |  |  |  |  |
| ICTO | 0.644(0.605 to 0.680) | 0.465(0.425 to 0.505) | 0.303(0.265 to 0.342) | 4.3 |
| PPO | 0.619(0.580 to 0.657) | 0.470(0.429 to 0.509) | 0.323(0.284 to 0.362) | 4.5 |
| CNB | 0.627(0.588 to 0.664) | 0.442(0.402 to 0.483) | 0.291(0.254 to 0.328) | 5.0 |
| MLP | 0.618(0.578 to 0.655) | 0.432(0.391 to 0.472) | 0.284(0.248 to 0.321) | 5.0 |
| Ensemble | 0.654(0.616 to 0.691) | 0.513(0.471 to 0.552) | 0.357(0.317 to 0.397) | 3.3 |

## Supplementary Table 16. Diagnostic Results of Simulated Human-Computer Collaborations

| **Case ID** | **Disease Name (**abbreviation) | **PhenoBrain** | | **Physicians with assistance** | | **PhenoBrain + Physicians with assistance** | |
| --- | --- | --- | --- | --- | --- | --- | --- |
|  |  | **Top-1 recall** | **Top-3 recall** | **Top-1 recall** | **Top-3 recall** | **Top-1 recall** | **Top-3 recall** |
| 1 | PWS | 1 | 1 | 0.5 | 0.5 | 1 | 1 |
| 2 | PWS | 0 | 1 | 1 | 1 | 1 | 1 |
| 3 | PWS | 1 | 1 | 0.75 | 0.75 | 1 | 1 |
| 4 | PWS | 0 | 0 | 0.5 | 0.5 | 0.5 | 0.5 |
| 5 | PWS | 0 | 1 | 0.75 | 0.75 | 0.75 | 1 |
| 6 | HD | 0 | 1 | 0.75 | 0.75 | 0.75 | 1 |
| 7 | HD | 1 | 1 | 0.75 | 1 | 1 | 1 |
| 8 | HD | 1 | 1 | 1 | 1 | 1 | 1 |
| 9 | HD | 1 | 1 | 0.25 | 0.25 | 1 | 1 |
| 10 | HD | 1 | 1 | 0.25 | 0.5 | 1 | 1 |
| 11 | MAS | 1 | 1 | 0.5 | 0.5 | 1 | 1 |
| 12 | MAS | 1 | 1 | 0.75 | 0.75 | 1 | 1 |
| 13 | MAS | 1 | 1 | 0.5 | 0.5 | 1 | 1 |
| 14 | MAS | 1 | 1 | 0.5 | 0.5 | 1 | 1 |
| 15 | MAS | 1 | 1 | 0.5 | 0.5 | 1 | 1 |
| 16 | MFS | 0 | 1 | 0.75 | 0.75 | 0.75 | 1 |
| 17 | MFS | 0 | 0 | 0.5 | 0.5 | 0.5 | 0.5 |
| 18 | MFS | 1 | 1 | 0.25 | 0.5 | 1 | 1 |
| 19 | MFS | 1 | 1 | 1 | 1 | 1 | 1 |
| 20 | MFS | 0 | 0 | 1 | 1 | 1 | 1 |
| 21 | ARVD/C | 0 | 0 | 0.5 | 0.5 | 0.5 | 0.5 |
| 22 | ARVD/C | 0 | 1 | 0 | 0 | 0 | 1 |
| 23 | ARVD/C | 0 | 0 | 1 | 1 | 1 | 1 |
| 24 | ARVD/C | 0 | 0 | 0.25 | 0.25 | 0.25 | 0.25 |
| 25 | BS | 0 | 1 | 0.75 | 0.75 | 0.75 | 1 |
| 26 | BS | 1 | 1 | 1 | 1 | 1 | 1 |
| 27 | BS | 0 | 0 | 0.75 | 1 | 0.75 | 1 |
| 28 | RCM | 0 | 0 | 0.25 | 0.5 | 0.25 | 0.5 |
| 29 | RCM | 0 | 0 | 0 | 0 | 0 | 0 |
| 30 | RCM | 0 | 0 | 0 | 0 | 0 | 0 |
| 31 | ALS | 0 | 0 | 0 | 0 | 0 | 0 |
| 32 | ALS | 0 | 0 | 0.5 | 0.75 | 0.5 | 0.75 |
| 33 | ALS | 0 | 1 | 0 | 0 | 0 | 1 |
| 34 | ALS | 0 | 0 | 0.25 | 0.5 | 0.25 | 0.5 |
| 35 | ALS | 0 | 0 | 0.25 | 0.25 | 0.25 | 0.25 |
| 36 | GMG | 1 | 1 | 1 | 1 | 1 | 1 |
| 37 | GMG | 1 | 1 | 0.75 | 0.75 | 1 | 1 |
| 38 | GMG | 1 | 1 | 0.25 | 0.25 | 1 | 1 |
| 39 | GMG | 1 | 1 | 0.5 | 0.5 | 1 | 1 |
| 40 | GMG | 1 | 1 | 1 | 1 | 1 | 1 |
| 41 | MSA | 1 | 1 | 0.25 | 0.25 | 1 | 1 |
| 42 | MSA | 1 | 1 | 1 | 1 | 1 | 1 |
| 43 | MSA | 1 | 1 | 1 | 1 | 1 | 1 |
| 44 | MSA | 1 | 1 | 0.5 | 0.75 | 1 | 1 |
| 45 | MSA | 0 | 0 | 1 | 1 | 1 | 1 |
| 46 | AS | 1 | 1 | 0 | 0 | 1 | 1 |
| 47 | AS | 1 | 1 | 1 | 1 | 1 | 1 |
| 48 | AS | 1 | 1 | 0.5 | 0.75 | 1 | 1 |
| 49 | AS | 0 | 0 | 0 | 0 | 0 | 0 |
| 50 | AS | 1 | 1 | 0 | 0 | 1 | 1 |
| 51 | AS | 0 | 0 | 0 | 0 | 0 | 0 |
| 52 | AS | 0 | 0 | 0 | 0.5 | 0 | 0.5 |
| 53 | AS | 0 | 0 | 0 | 0 | 0 | 0 |
| 54 | FD | 0 | 0 | 0 | 0 | 0 | 0 |
| 55 | FD | 0 | 0 | 0 | 0 | 0 | 0 |
| 56 | FD | 1 | 1 | 0 | 0 | 1 | 1 |
| 57 | GS | 1 | 1 | 0 | 0.5 | 1 | 1 |
| 58 | GS | 1 | 1 | 0.25 | 0.25 | 1 | 1 |
| 59 | GS | 1 | 1 | 0 | 0.25 | 1 | 1 |
| 60 | GS | 0 | 0 | 0.5 | 1 | 0.5 | 1 |
| 61 | PNH | 0 | 0 | 0 | 0.33 | 0 | 0.33 |
| 62 | PNH | 0 | 0 | 1 | 1 | 1 | 1 |
| 63 | PNH | 0 | 1 | 0.25 | 0.75 | 0.25 | 1 |
| 64 | PNH | 0 | 0 | 0 | 0 | 0 | 0 |
| 65 | PNH | 0 | 0 | 1 | 1 | 1 | 1 |
| 66 | POS | 0 | 1 | 0.25 | 0.25 | 0.25 | 1 |
| 67 | POS | 1 | 1 | 0.75 | 0.75 | 1 | 1 |
| 68 | POS | 1 | 1 | 0.75 | 0.75 | 1 | 1 |
| 69 | POS | 1 | 1 | 0.5 | 0.5 | 1 | 1 |
| 70 | POS | 1 | 1 | 0.5 | 0.5 | 1 | 1 |
| 71 | NPD | 1 | 1 | 0.75 | 0.75 | 1 | 1 |
| 72 | NPD | 0 | 0 | 0 | 0 | 0 | 0 |
| 73 | NPD | 0 | 0 | 0 | 0 | 0 | 0 |
| 74 | NPD | 1 | 1 | 0 | 0 | 1 | 1 |
| 75 | NPD | 0 | 0 | 0 | 0 | 0 | 0 |

## Supplementary Table 17. Median ranks of 5 ALS cases by PhenoBrain

| **Methods** | **Case 1** | **Case 2** | **Case 3** | **Case 4** | **Case 5** |
| --- | --- | --- | --- | --- | --- |
| ICTO | 66.0 (10.0) | 496.0 (2.0) | 39.0 (5.0) | 147.0 (32.0) | 93.0 (12.0) |
| PPO | 80.0 (3.0) | 178.0 (1.0) | 15.0 (2.0) | 38.0 (18.0) | 120.0 (15.0) |
| CNB | 56.0 (22.0) | 116.0 (2.0) | 38.0 (18.0) | 74.0 (37.0) | 69.0 (9.0) |
| MLP | 47.0 (19.0) | 589.0 (6.0) | 49.0 (31.0) | 101.0 (67.0) | 147.0 (30.0) |
| Ensemble | 73.0 (8.0) | 278.0 (2.0) | 27.0 (3.0) | 74.0 (19.0) | 99.0 (18.0) |

The content in parentheses is the diagnostic result of the case with 'deduced' phenotypes. “With deduced phenotypes”: we manually added two phenotypes into the five ALS cases: abnormal upper motor neuron morphology (HP:0002127) and abnormal lower motor neuron morphology (HP:0002366)).

## Supplementary Table 18. Median ranks of 17 diagnostic methods using various knowledgebases on the Public Test Set

| **Methods** | **OMIM** | **ORPHA** | **OMIM + CCRD** | **ORPHA + CCRD** | **OMIM + ORPHA** | **OMIM + ORPHA + CCRD** |
| --- | --- | --- | --- | --- | --- | --- |
| Res | 12.0 | 96.5 | 11.0 | 24.5 | 13.0 | 12.0 |
| BOQA | 25.0 | 157.5 | 15.5 | 51.0 | 19.0 | 15.0 |
| RDD | 38.0 | 220.0 | 33.0 | 24.0 | 36.0 | 31.0 |
| GDDP | 7.0 | 113.0 | 7.0 | 16.0 | 9.0 | 9.0 |
| RBP | 9.0 | 212.0 | 6.0 | 14.0 | 11.0 | 6.0 |
| MinIC | 6.0 | 120.0 | 4.0 | 14.0 | 7.0 | 6.0 |
| Lin | 27.5 | 126.0 | 18.0 | 30.5 | 23.0 | 17.0 |
| JC | 13.0 | 137.5 | 9.0 | 18.5 | 12.0 | 9.0 |
| SimGIC | 29.0 | 83.5 | 35.0 | 24.5 | 35.0 | 43.0 |
| SimUI | 75.5 | 98.5 | 99.0 | 93.0 | 123.0 | 144.0 |
| TO | 14.0 | 148.5 | 8.0 | 20.0 | 14.0 | 10.0 |
| Cosine | 50.0 | 84.0 | 51.0 | 36.0 | 62.0 | 67.0 |
| Best* | 6.0 | 83.5 | 4.0 | 14.0 | 7.0 | 6.0 |
|  |  |  |  |  |  |  |
| ICTO | 5.0 | 128.0 | 4.0 | 10.0 | 6.0 | 5.0 |
| PPO | 6.0 | 168.5 | 5.0 | 12.0 | 6.0 | 4.0 |
| CNB | 5.0 | 83.0 | 4.5 | 10.0 | 6.0 | 5.0 |
| MLP | 10.0 | 92.0 | 6.0 | 8.0 | 8.0 | 6.0 |
| Ensemble | 6.0 | 101.5 | 4.0 | 7.0 | 6.0 | 4.0 |

*Best results among all benchmarks.

## Supplementary Table 19 Median ranks among 17 diagnostic methods using various knowledgebases on 24 Methylmalonic academia cases

| **Methods** | **OMIM** | **ORPHA** | **OMIM + CCRD** | **ORPHA + CCRD** | **OMIM + ORPHA** | **OMIM + ORPHA + CCRD** |
| --- | --- | --- | --- | --- | --- | --- |
| Res | 13.0 | 97.5 | 10.0 | 8.5 | 15.0 | 15.5 |
| BOQA | 14.0 | 373.5 | 4.0 | 5.5 | 8.5 | 5.5 |
| RDD | 115.5 | 283.5 | 30.5 | 10.5 | 165.5 | 39.0 |
| GDDP | 6.0 | 179.0 | 3.0 | 5.0 | 7.5 | 4.0 |
| RBP | 4.0 | 112.0 | 2.5 | 1.5 | 6.5 | 2.5 |
| MinIC | 11.5 | 122.0 | 3.0 | 3.0 | 12.5 | 3.0 |
| Lin | 23.0 | 49.0 | 9.0 | 9.0 | 19.0 | 19.5 |
| JC | 8.5 | 87.5 | 4.0 | 3.5 | 9.5 | 7.5 |
| SimGIC | 26.0 | 80.0 | 12.0 | 6.0 | 45.0 | 14.5 |
| SimUI | 62.5 | 106.5 | 47.0 | 35.5 | 144.0 | 104.5 |
| TO | 12.0 | 106.5 | 5.0 | 4.0 | 7.5 | 6.5 |
| Cosine | 36.0 | 101.0 | 25.0 | 15.5 | 67.5 | 47.0 |
| Best* | 4.0 | 49.0 | 2.5 | 1.5 | 6.5 | 2.5 |
|  |  |  |  |  |  |  |
| ICTO | 5.0 | 124.0 | 3.0 | 2.5 | 4.0 | 4.5 |
| PPO | 5.0 | 203.5 | 3.0 | 1.5 | 6.0 | 3.0 |
| CNB | 4.0 | 128.0 | 3.0 | 2.5 | 7.0 | 4.0 |
| MLP | 4.0 | 133.0 | 2.0 | 2.5 | 6.5 | 3.0 |
| Ensemble | 4.0 | 114.0 | 3.0 | 1.0 | 4.0 | 3.0 |

*Best results among all benchmarks.

## Supplementary Table 20. P-values for comparing the Ensemble method against the 12 diagnostic methods on rare disease datasets*

| **Methods** | **Public Test Set** | **PUMCH-L** | **Human-Computer Test Set (PUMCH-ADM)** |
| --- | --- | --- | --- |
| Res | 1.58E-45 | 6.29E-35 | 6.25E-04 |
| BOQA | 1.26E-55 | 1.17E-20 | 4.50E-06 |
| RDD | 1.46E-80 | 3.48E-52 | 5.59E-05 |
| GDDP | 4.03E-29 | 2.85E-15 | 4.66E-02 |
| RBP | 4.79E-25 | 4.00E-26 | 7.83E-03 |
| MinIC | 7.97E-17 | 1.22E-17 | 1.04E-02 |
| Lin | 4.57E-58 | 2.04E-72 | 8.80E-05 |
| JC | 2.02E-28 | 1.37E-38 | 2.23E-03 |
| SimGIC | 1.63E-64 | 1.76E-01 | 2.48E-07 |
| SimUI | 7.33E-94 | 5.73E-10 | 1.74E-09 |
| TO | 1.6E-39 | 1.05E-86 | 7.92E-05 |
| Cosine | 6.71E-80 | 4.35E-05 | 1.63E-07 |

*P-values were obtained by Wilcoxon signed rank test.

## Supplementary Table 21. P-values for comparing ensemble method against physicians and large language models on Human-Computer Test Set*

| **Methods** | **Human-Computer Test Set** |
| --- | --- |
| Physicians | 1.21E-06 |
| Physicians_w_assistance | 6.32E-05 |
| ChatGPT (EHR) | 2.01E-04 |
| GPT-4 (EHR) | 4.80E-02 |
| ChatGPT (HPO) | 1.04E-04 |
| GPT-4 (HPO) | 1.48E-01 |

*P-values were obtained by Wilcoxon signed rank test.

## Supplementary Table 22. Performance of ensemble method and physicians and large language models on Human-Computer Test Set

| **Methods** | **Recall@10 (95% CI)** | **Recall@3 (95% CI)** | **Recall@1 (95% CI)** |
| --- | --- | --- | --- |
| Ensemble | 0.813(0.720 to 0.907) | 0.613(0.507 to 0.720) | 0.493(0.387 to 0.600) |
| Physicians | 0.481(0.400 to 0.563) | 0.468(0.386 to 0.551) | 0.407(0.323 to 0.490) |
| Physicians_w_assistance | 0.524(0.441 to 0.607) | 0.511(0.428 to 0.594) | 0.447(0.363 to 0.530) |
| ChatGPT (EHR) | 0.520(0.413 to 0.627) | 0.320(0.213 to 0.427) | 0.240(0.147 to 0.333) |
| GPT-4 (EHR) | 0.667(0.560 to 0.773) | 0.507(0.400 to 0.613) | 0.373(0.267 to 0.480) |
| ChatGPT (HPO) | 0.493(0.387 to 0.600) | 0.373(0.267 to 0.480) | 0.227(0.133 to 0.320) |
| GPT-4 (HPO) | 0.720(0.613 to 0.813) | 0.587(0.480 to 0.693) | 0.373(0.267 to 0.480) |
| Ensemble + Physicians_w_assistance |  | 0.768(0.679 to 0.856) | 0.677(0.580 to 0.770) |

## Supplementary Table 23. Performance of 17 diagnostic methods on simulated datasets

| **Methods** | **Recall@10 (95% CI)** | **Recall@3 (95% CI)** | **Recall@1 (95% CI)** | **Median Rank** |
| --- | --- | --- | --- | --- |
| **SIM** | | | | |
| Res | 0.994(0.992 to 0.996) | 0.980(0.976 to 0.984) | 0.939(0.931 to 0.946) | 1.0 |
| BOQA | 0.983(0.979 to 0.987) | 0.964(0.958 to 0.970) | 0.897(0.888 to 0.907) | 1.0 |
| RDD | 0.857(0.847 to 0.868) | 0.653(0.638 to 0.668) | 0.412(0.397 to 0.426) | 2.0 |
| GDDP | 0.971(0.966 to 0.976) | 0.928(0.920 to 0.935) | 0.883(0.873 to 0.892) | 1.0 |
| RBP | 0.991(0.988 to 0.994) | 0.966(0.960 to 0.971) | 0.923(0.915 to 0.931) | 1.0 |
| MinIC | 0.994(0.992 to 0.997) | 0.984(0.981 to 0.988) | 0.948(0.941 to 0.954) | 1.0 |
| Lin | 0.985(0.981 to 0.988) | 0.970(0.965 to 0.975) | 0.923(0.914 to 0.930) | 1.0 |
| JC | 0.992(0.989 to 0.995) | 0.971(0.966 to 0.976) | 0.930(0.922 to 0.938) | 1.0 |
| SimGIC | 0.579(0.564 to 0.594) | 0.369(0.355 to 0.384) | 0.237(0.224 to 0.250) | 7.0 |
| SimUI | 0.346(0.332 to 0.360) | 0.215(0.203 to 0.228) | 0.140(0.130 to 0.151) | 31.0 |
| TO | 0.984(0.980 to 0.988) | 0.970(0.965 to 0.975) | 0.918(0.910 to 0.927) | 1.0 |
| Cosine | 0.639(0.624 to 0.653) | 0.431(0.417 to 0.446) | 0.265(0.252 to 0.278) | 5.0 |
| Best* | 0.994(0.992 to 0.997) | 0.984(0.981 to 0.988) | 0.948(0.941 to 0.954) | 1.0 |
|  |  |  |  |  |
| ICTO | 0.995(0.993 to 0.997) | 0.975(0.970 to 0.980) | 0.940(0.933 to 0.947) | 1.0 |
| PPO | 0.994(0.991 to 0.996) | 0.975(0.970 to 0.980) | 0.936(0.928 to 0.943) | 1.0 |
| CNB | 0.994(0.992 to 0.997) | 0.976(0.971 to 0.980) | 0.916(0.908 to 0.924) | 1.0 |
| MLP | 0.984(0.980 to 0.988) | 0.963(0.958 to 0.969) | 0.901(0.892 to 0.910) | 1.0 |
| Ensemble | 0.996(0.994 to 0.998) | 0.977(0.973 to 0.982) | 0.938(0.930 to 0.945) | 1.0 |
| **SIM(N)** | | | | |
| Res | 0.955(0.949 to 0.961) | 0.905(0.897 to 0.914) | 0.819(0.807 to 0.830) | 1.0 |
| BOQA | 0.960(0.954 to 0.966) | 0.931(0.923 to 0.938) | 0.844(0.833 to 0.855) | 1.0 |
| RDD | 0.838(0.828 to 0.849) | 0.635(0.621 to 0.649) | 0.427(0.413 to 0.442) | 2.0 |
| GDDP | 0.909(0.900 to 0.917) | 0.827(0.816 to 0.838) | 0.721(0.708 to 0.735) | 1.0 |
| RBP | 0.966(0.960 to 0.971) | 0.930(0.922 to 0.937) | 0.879(0.869 to 0.888) | 1.0 |
| MinIC | 0.983(0.979 to 0.986) | 0.961(0.955 to 0.966) | 0.904(0.895 to 0.913) | 1.0 |
| Lin | 0.961(0.956 to 0.967) | 0.918(0.910 to 0.926) | 0.841(0.830 to 0.851) | 1.0 |
| JC | 0.982(0.978 to 0.986) | 0.950(0.943 to 0.956) | 0.907(0.898 to 0.916) | 1.0 |
| SimGIC | 0.741(0.728 to 0.754) | 0.534(0.519 to 0.548) | 0.376(0.362 to 0.391) | 3.0 |
| SimUI | 0.510(0.495 to 0.525) | 0.346(0.332 to 0.360) | 0.240(0.227 to 0.252) | 10.0 |
| TO | 0.910(0.901 to 0.919) | 0.815(0.804 to 0.827) | 0.673(0.659 to 0.687) | 1.0 |
| Cosine | 0.672(0.658 to 0.686) | 0.479(0.464 to 0.494) | 0.312(0.298 to 0.326) | 4.0 |
| Best* | 0.983(0.979 to 0.986) | 0.961(0.955 to 0.966) | 0.907(0.898 to 0.916) | 1.0 |
|  |  |  |  |  |
| ICTO | 0.987(0.983 to 0.990) | 0.958(0.952 to 0.964) | 0.914(0.905 to 0.922) | 1.0 |
| PPO | 0.984(0.981 to 0.988) | 0.957(0.951 to 0.963) | 0.911(0.902 to 0.919) | 1.0 |
| CNB | 0.974(0.969 to 0.979) | 0.935(0.928 to 0.942) | 0.847(0.836 to 0.858) | 1.0 |
| MLP | 0.963(0.958 to 0.969) | 0.931(0.924 to 0.939) | 0.853(0.843 to 0.864) | 1.0 |
| Ensemble | 0.988(0.985 to 0.991) | 0.960(0.954 to 0.966) | 0.910(0.902 to 0.919) | 1.0 |
| **SIM(I)** | | | | |
| Res | 0.742(0.728 to 0.756) | 0.577(0.562 to 0.592) | 0.387(0.373 to 0.402) | 2.0 |
| BOQA | 0.660(0.646 to 0.675) | 0.516(0.500 to 0.531) | 0.364(0.349 to 0.378) | 3.0 |
| RDD | 0.085(0.077 to 0.094) | 0.033(0.028 to 0.039) | 0.011(0.008 to 0.015) | 168.0 |
| GDDP | 0.318(0.304 to 0.333) | 0.222(0.210 to 0.235) | 0.134(0.123 to 0.144) | 59.0 |
| RBP | 0.738(0.725 to 0.752) | 0.573(0.558 to 0.588) | 0.386(0.371 to 0.401) | 2.0 |
| MinIC | 0.750(0.737 to 0.763) | 0.581(0.565 to 0.596) | 0.389(0.374 to 0.405) | 2.0 |
| Lin | 0.578(0.563 to 0.593) | 0.373(0.358 to 0.388) | 0.210(0.197 to 0.223) | 7.0 |
| JC | 0.360(0.345 to 0.374) | 0.191(0.179 to 0.203) | 0.099(0.090 to 0.108) | 23.0 |
| SimGI | 0.008(0.005 to 0.011) | 0.003(0.001 to 0.004) | 0.001(0.000 to 0.002) | 1131.0 |
| SimUI | 0.002(0.001 to 0.004) | 0.000(0.000 to 0.001) | 0.000(0.000 to 0.000) | 2698.0 |
| TO | 0.740(0.726 to 0.753) | 0.575(0.559 to 0.590) | 0.384(0.369 to 0.399) | 2.0 |
| Cosine | 0.021(0.017 to 0.026) | 0.006(0.004 to 0.008) | 0.001(0.000 to 0.003) | 619.0 |
| Best* | 0.750(0.737 to 0.763) | 0.581(0.565 to 0.596) | 0.389(0.374 to 0.405) | 2.0 |
|  |  |  |  |  |
| ICTO | 0.650(0.635 to 0.665) | 0.412(0.397 to 0.428) | 0.223(0.210 to 0.236) | 5.0 |
| PPO | 0.826(0.814 to 0.838) | 0.691(0.677 to 0.705) | 0.516(0.501 to 0.532) | 1.0 |
| CNB | 0.727(0.713 to 0.741) | 0.587(0.571 to 0.602) | 0.427(0.412 to 0.443) | 2.0 |
| MLP | 0.438(0.422 to 0.453) | 0.291(0.277 to 0.305) | 0.183(0.172 to 0.195) | 17.0 |
| Ensemble | 0.825(0.813 to 0.836) | 0.683(0.668 to 0.697) | 0.508(0.493 to 0.524) | 1.0 |
| **SIM(I;N)** | | | | |
| Res | 0.377(0.363 to 0.392) | 0.212(0.200 to 0.225) | 0.108(0.099 to 0.118) | 21.0 |
| BOQA | 0.509(0.493 to 0.524) | 0.372(0.357 to 0.386) | 0.254(0.242 to 0.267) | 10.0 |
| RDD | 0.110(0.100 to 0.119) | 0.034(0.029 to 0.039) | 0.007(0.004 to 0.009) | 84.0 |
| GDDP | 0.091(0.083 to 0.100) | 0.048(0.042 to 0.055) | 0.022(0.018 to 0.027) | 245.0 |
| RBP | 0.351(0.337 to 0.366) | 0.202(0.190 to 0.214) | 0.125(0.115 to 0.135) | 21.0 |
| MinIC | 0.575(0.560 to 0.590) | 0.381(0.367 to 0.396) | 0.219(0.207 to 0.232) | 7.0 |
| Lin | 0.418(0.403 to 0.433) | 0.223(0.210 to 0.235) | 0.112(0.102 to 0.121) | 16.0 |
| JC | 0.317(0.303 to 0.331) | 0.155(0.145 to 0.166) | 0.077(0.069 to 0.085) | 32.0 |
| SimGIC | 0.012(0.009 to 0.015) | 0.004(0.002 to 0.005) | 0.002(0.001 to 0.003) | 894.0 |
| SimUI | 0.010(0.007 to 0.013) | 0.004(0.002 to 0.006) | 0.001(0.000 to 0.002) | 1772.0 |
| TO | 0.268(0.255 to 0.282) | 0.134(0.124 to 0.144) | 0.060(0.053 to 0.067) | 40.0 |
| Cosine | 0.022(0.018 to 0.027) | 0.006(0.004 to 0.009) | 0.001(0.000 to 0.003) | 617.0 |
| Best* | 0.575(0.560 to 0.590) | 0.381(0.367 to 0.396) | 0.254(0.242 to 0.267) | 7.0 |
|  |  |  |  |  |
| ICTO | 0.410(0.395 to 0.424) | 0.196(0.184 to 0.208) | 0.082(0.074 to 0.090) | 16.0 |
| PPO | 0.749(0.736 to 0.762) | 0.573(0.558 to 0.588) | 0.383(0.369 to 0.398) | 2.0 |
| CNB | 0.282(0.268 to 0.295) | 0.164(0.153 to 0.175) | 0.087(0.079 to 0.096) | 41.0 |
| MLP | 0.295(0.281 to 0.308) | 0.169(0.158 to 0.180) | 0.093(0.084 to 0.102) | 47.0 |
| Ensemble | 0.649(0.634 to 0.663) | 0.428(0.413 to 0.443) | 0.238(0.225 to 0.251) | 5.0 |
| **Average** | | | | |
| Res | 0.767(0.762 to 0.772) | 0.669(0.663 to 0.674) | 0.563(0.558 to 0.569) | 6.3 |
| BOQA | 0.778(0.773 to 0.784) | 0.695(0.690 to 0.701) | 0.590(0.584 to 0.596) | 3.8 |
| RDD | 0.473(0.468 to 0.478) | 0.339(0.333 to 0.344) | 0.214(0.209 to 0.219) | 64.0 |
| GDDP | 0.572(0.567 to 0.577) | 0.506(0.501 to 0.511) | 0.440(0.435 to 0.445) | 76.5 |
| RBP | 0.762(0.757 to 0.767) | 0.668(0.662 to 0.673) | 0.578(0.573 to 0.584) | 6.3 |
| MinIC | 0.826(0.820 to 0.831) | 0.727(0.721 to 0.732) | 0.615(0.609 to 0.621) | 2.8 |
| Lin | 0.736(0.730 to 0.741) | 0.621(0.616 to 0.627) | 0.521(0.516 to 0.526) | 6.3 |
| JC | 0.663(0.657 to 0.668) | 0.567(0.562 to 0.571) | 0.503(0.499 to 0.507) | 14.3 |
| SimGIC | 0.335(0.330 to 0.340) | 0.227(0.222 to 0.233) | 0.154(0.149 to 0.159) | 508.8 |
| SimUI | 0.217(0.212 to 0.222) | 0.141(0.137 to 0.146) | 0.095(0.091 to 0.099) | 1127.8 |
| TO | 0.726(0.720 to 0.731) | 0.623(0.618 to 0.629) | 0.509(0.503 to 0.514) | 11.0 |
| Cosine | 0.339(0.333 to 0.344) | 0.231(0.225 to 0.236) | 0.145(0.140 to 0.150) | 311.3 |
| Best* | 0.826(0.820 to 0.831) | 0.727(0.721 to 0.732) | 0.615(0.609 to 0.621) | 2.8 |
|  |  |  |  |  |
| ICTO | 0.760(0.755 to 0.766) | 0.635(0.630 to 0.641) | 0.540(0.535 to 0.544) | 5.8 |
| PPO | 0.888(0.884 to 0.893) | 0.799(0.793 to 0.805) | 0.687(0.680 to 0.693) | 1.3 |
| CNB | 0.744(0.739 to 0.749) | 0.665(0.660 to 0.670) | 0.569(0.564 to 0.575) | 11.3 |
| MLP | 0.670(0.665 to 0.675) | 0.589(0.584 to 0.594) | 0.508(0.503 to 0.513) | 16.5 |
| Ensemble | 0.865(0.860 to 0.869) | 0.762(0.757 to 0.768) | 0.649(0.643 to 0.654) | 2.0 |

## Supplementary Table 24. Performance of various medical text processing methods on test subsets of PUMCH-S (34 cases)

| **Methods** | **F1** | **Recall** | **Precision** |
| --- | --- | --- | --- |
| PBTagger | 0.732 | 0.824 | 0.658 |
| CHPO | 0.683 | 0.549 | 0.902 |
| UMLS | 0.701 | 0.647 | 0.765 |

The phenotypes annotated by doctors serve as the gold standard.

## Supplementary Table 25. Performance of Phenomizer and 5 proposed methods on Public Test Set* (753 cases) Using Phenomizer's KnowledgeBase

| **Methods** | **Recall@10 (95% CI)** | **Recall@3 (95% CI)** | **Recall@1 (95% CI)** | **Median Rank** |
| --- | --- | --- | --- | --- |
| Phenomizer | 0.434(0.398-0.470) | 0.256(0.226-0.288) | 0.114(0.092-0.137) | 15.0 |
|  |  |  |  |  |
| ICTO | 0.620(0.586-0.653) | 0.398(0.368-0.428) | 0.231(0.202-0.259) | 7.0 |
| PP0 | 0.611(0.578-0.644) | 0.445(0.406-0.483) | 0.258(0.230-0.285) | 5.0 |
| CNB | 0.584(0.553-0.616) | 0.386(0.359-0.412) | 0.205(0.182-0.229) | 8.0 |
| MLP | 0.586(0.552-0.621) | 0.351(0.316-0.385) | 0.207(0.179-0.235) | 8.0 |
| Ensemble | 0.636(0.608-0.665) | 0.465(0.440-0.491) | 0.264(0.233-0.296) | 5.0 |

*Phenomizer makes differential diagnosis among 8,012 rare diseases. our methods are trained using the disease knowledgebases of Phenomizer, without mapping or fusion between diseases.

## Supplementary Table 26. Performance of LIRICAL tool and 5 proposed methods on Public Test Set* (850 cases) Using LIRICAL's KnowledgeBase

| **Methods** | **Recall@10 (95% CI)** | **Recall@3 (95% CI)** | **Recall@1 (95% CI)** | **Median Rank** |
| --- | --- | --- | --- | --- |
| LIRICAL | 0.560 (0.526 to 0.593) | 0.407(0.374 to 0.440) | 0.232(0.204 to 0.260) | 6.0 |
|  |  |  |  |  |
| ICTO | 0.619(0.590 to 0.648) | 0.415(0.386 to 0.445) | 0.269(0.245 to 0.294) | 6.0 |
| PPO | 0.612(0.581 to 0.643) | 0.461(0.430 to 0.492) | 0.286(0.260 to 0.313) | 5.0 |
| CNB | 0.614(0.584 to 0.645) | 0.424(0.396 to 0.452) | 0.248(0.218 to 0.278) | 7.0 |
| MLP | 0.600(0.571 to 0.630) | 0.373(0.342 to 0.403) | 0.235(0.204 to 0.267) | 7.0 |
| Ensemble | 0.632(0.601 to 0.663) | 0.474(0.444 to 0.504) | 0.294(0.267 to 0.321) | 4.0 |

*LIRICAL makes differential diagnosis among 8,167 rare diseases. our methods are trained using the disease knowledgebases of LIRICAL, without mapping or fusion between diseases.

## Supplementary Table 27. Performance of 17 diagnostic methods and 2 tools on the 101 cases (public set) with less than 3 phenotypes

| **Methods** | **Recall@10 (95% CI)** | **Recall@3 (95% CI)** | **Recall@1 (95% CI)** | **Median Rank** |
| --- | --- | --- | --- | --- |
| Res | 0.713(0.624-0.802) | 0.505(0.406-0.604) | 0.069(0.020-0.119) | 3.0 |
| BOQA | 0.505(0.406-0.604) | 0.495(0.396-0.594) | 0.426(0.327-0.525) | 9.0 |
| RDD | 0.396(0.297-0.495) | 0.050(0.010-0.099) | 0.010(0.000-0.030) | 52.0 |
| GDDP | 0.693(0.604-0.782) | 0.465(0.366-0.564) | 0.376(0.287-0.475) | 4.0 |
| RBP | 0.713(0.624-0.802) | 0.525(0.426-0.624) | 0.069(0.020-0.119) | 3.0 |
| MinIC | 0.733(0.644-0.812) | 0.515(0.416-0.614) | 0.069(0.020-0.119) | 3.0 |
| Lin | 0.673(0.584-0.762) | 0.505(0.406-0.604) | 0.059(0.020-0.109) | 3.0 |
| JC | 0.723(0.634-0.812) | 0.495(0.396-0.594) | 0.059(0.020-0.109) | 4.0 |
| SimGIC | 0.069(0.020-0.119) | 0.050(0.010-0.099) | 0.010(0.000-0.030) | 43.0 |
| SimUI | 0.059(0.020-0.109) | 0.050(0.010-0.099) | 0.010(0.000-0.030) | 2116.0 |
| TO | 0.733(0.644-0.812) | 0.495(0.396-0.594) | 0.059(0.020-0.109) | 4.0 |
| Cosine | 0.059(0.020-0.109) | 0.050(0.010-0.099) | 0.010(0.000-0.030) | 286.0 |
| Best* | 0.733(0.644-0.812) | 0.525(0.426-0.624) | 0.426(0.327-0.525) | 3.0 |
|  |  |  |  |  |
| Phenomizer | 0.287(0.202-0.383) | 0.053(0.011-0.106) | 0.032(0.000-0.074) | 15.0 |
| LIRICAL | 0.713(0.624-0.802) | 0.465(0.366-0.564) | 0.337(0.248-0.426) | 5.0 |
|  |  |  |  |  |
| ICTO | 0.604(0.505-0.693) | 0.525(0.426-0.624) | 0.069(0.030-0.119) | 3.0 |
| PP0 | 0.723(0.634-0.812) | 0.663(0.574-0.752) | 0.485(0.386-0.584) | 2.0 |
| CNB | 0.614(0.515-0.703) | 0.525(0.426-0.624) | 0.446(0.347-0.545) | 2.0 |
| MLP | 0.723(0.634-0.812) | 0.554(0.455-0.653) | 0.109(0.050-0.168) | 2.0 |
| Ensemble | 0.723(0.634-0.812) | 0.554(0.455-0.653) | 0.119(0.059-0.188) | 2.0 |

* Among the 101 cases of the public set, 7 have HPO terms outside the knowledgebase of Phenomizer. Excluding these cases resulted in 94 cases for Phenomizer experiment.

# Supplementary References

1 Deng, K., Bol, P. K., Li, K. J. & Liu, J. S. On the unsupervised analysis of domain-specific Chinese texts. *Proceedings of the National Academy of Sciences* **113**, 6154-6159 (2016).

2 Lan, Z. *et al.* Albert: A lite bert for self-supervised learning of language representations. *arXiv preprint arXiv:1909.11942* (2019).

3 Devlin, J., Chang, M.-W., Lee, K. & Toutanova, K. Bert: Pre-training of deep bidirectional transformers for language understanding. *arXiv preprint arXiv:1810.04805* (2018).

4 Kingma, D. P. & Ba, J. Adam: A method for stochastic optimization. *arXiv preprint arXiv:1412.6980* (2014).

5 Köhler, S. *et al.* Clinical diagnostics in human genetics with semantic similarity searches in ontologies. *The American Journal of Human Genetics* **85**, 457-464 (2009).

6 Köhler, S. *et al.* The human phenotype ontology in 2021. *Nucleic acids research* **49**, D1207-D1217 (2021).

7 Hu, J., Lu, J. & Tan, Y.-P. Discriminative deep metric learning for face verification in the wild. in *Proceedings of the IEEE conference on computer vision and pattern recognition.* 1875-1882 (2014).

8 Resnik, P. Using information content to evaluate semantic similarity in a taxonomy. *arXiv preprint cmp-lg/9511007* (1995).

9 Bauer, S., Kohler, S., Schulz, M. H. & Robinson, P. N. Bayesian ontology querying for accurate and noise-tolerant semantic searches. *Bioinformatics* **28**, 2502-2508, doi:10.1093/bioinformatics/bts471 (2012).

10 Pinol, M. *et al.* Rare disease discovery: An optimized disease ranking system. *IEEE Transactions on Industrial Informatics* **13**, 1184-1192 (2017).

11 Chen, J. *et al.* Novel phenotype–disease matching tool for rare genetic diseases. *Genetics in Medicine* **21**, 339-346 (2019).

12 Gong, X., Jiang, J., Duan, Z. & Lu, H. A new method to measure the semantic similarity from query phenotypic abnormalities to diseases based on the human phenotype ontology. *BMC bioinformatics* **19**, 111-119 (2018).

13 Peng, J. *et al.* Measuring phenotype semantic similarity using human phenotype ontology. in *2016 IEEE International Conference on Bioinformatics and Biomedicine (BIBM).* 763-766 (2016).

14 Lin, D. editor An information-theoretic definition of similarity. in *Icml* **98**, 296-304 (1998).

15 Jiang, J. J. & Conrath, D. W. Semantic similarity based on corpus statistics and lexical taxonomy. *arXiv preprint cmp-lg/9709008* (1997).

16 Pesquita, C., Faria, D., Falcao, A. O., Lord, P. & Couto, F. M. Semantic similarity in biomedical ontologies. *PLoS computational biology* **5**, e1000443 (2009).

17 Gentleman, R. Visualizing and distances using GO. *URL* [*http://www*](http://www)*. bioconductor. org/docs/vignettes. html* **38** (2005).

18 Mistry, M. & Pavlidis, P. Gene Ontology term overlap as a measure of gene functional similarity. *BMC bioinformatics* **9**, 1-11 (2008).
