# Supplementary material for: A phenotype-based AI pipeline outperforms human experts in differentially diagnosing rare diseases using EHRs
Source: NPJ Digit Med. 2025 Jan 28;8:68. doi: 10.1038/s41746-025-01452-1 (PMC11775211; doi:10.1038/s41746-025-01452-1)
Supplement: Supplementary file 3 — Supplementary Data 2 [file 41746_2025_1452_MOESM3_ESM.pdf]

```
[
  [
    [
      "HP:0001225",
      "HP:0001382",
      "HP:0001443",
      "HP:0001894",
      "HP:0001974",
      "HP:0003419",
      "HP:0025238",
      "HP:0030833",
      "HP:0030834",
      "HP:0030835",
      "HP:0030836",
      "HP:0030839"
    ],
    [
      "OMIM:106300"
    ]
  ],
  [
    [
      "HP:0000793",
      "HP:0001974",
      "HP:0002205",
      "HP:0002633",
      "HP:0003496",
      "HP:0100778"
    ],
    [
      "OMIM:123550",
      "ORPHA:91138"
    ]
  ],
  [
    [
      "HP:0000155",
      "HP:0000246",
      "HP:0000360",
      "HP:0000365",
      "HP:0000572",
      "HP:0000651",
      "HP:0000787",
      "HP:0000790",
      "HP:0001097",
      "HP:0001155",
      "HP:0001279",
      "HP:0001369",
      "HP:0001880",
      "HP:0001947",
      "HP:0002018",
      "HP:0002027",
      "HP:0002150",
      "HP:0002315",
      "HP:0002321",
```

```
      "HP:0002653",
      "HP:0002875",
      "HP:0003236",
      "HP:0003326",
      "HP:0003401",
      "HP:0003418",
      "HP:0003474",
      "HP:0003537",
      "HP:0004411",
      "HP:0005197",
      "HP:0009763",
      "HP:0011935",
      "HP:0012378",
      "HP:0012595",
      "HP:0030140",
      "HP:0030833",
      "HP:0030836",
      "HP:0030840",
      "HP:0032154",
      "HP:0100749",
      "HP:0100827",
      "HP:0200034",
      "HP:0200039",
      "HP:0410019"
    ],
    [
      "OMIM:109650",
      "ORPHA:117"
    ]
  ],
  [
    [
      "HP:0000421",
      "HP:0002105",
      "HP:0002113",
      "HP:0002829",
      "HP:0002907",
      "HP:0011227",
      "HP:0012213",
      "HP:0032230"
    ],
    [
      "OMIM:608710",
      "ORPHA:900"
    ]
  ],
  [
    [
      "HP:0000093",
      "HP:0001541",
      "HP:0001735",
      "HP:0001744",
      "HP:0001876",
      "HP:0001945",
      "HP:0002014",
      "HP:0002027",
```

```
      "HP:0002586",
      "HP:0002829",
      "HP:0003236",
      "HP:0003324",
      "HP:0003326",
      "HP:0003493",
      "HP:0003546",
      "HP:0011106",
      "HP:0012378",
      "HP:0025435",
      "HP:0030010",
      "HP:0410019"
    ],
    [
      "OMIM:152700",
      "OMIM:606579",
      "OMIM:609939",
      "OMIM:614420",
      "ORPHA:300345",
      "ORPHA:93552"
    ]
  ],
  [
    [
      "HP:0000083",
      "HP:0000093",
      "HP:0000099",
      "HP:0000107",
      "HP:0000554",
      "HP:0001369",
      "HP:0001888",
      "HP:0002204",
      "HP:0002625",
      "HP:0002907",
      "HP:0003225",
      "HP:0003493",
      "HP:0005584",
      "HP:0030839",
      "HP:0032376"
    ],
    [
      "OMIM:144700",
      "OMIM:152700",
      "OMIM:300854",
      "OMIM:605074",
      "OMIM:606579",
      "OMIM:609939",
      "OMIM:614420",
      "ORPHA:300345",
      "ORPHA:47044",
      "ORPHA:93552"
    ]
  ],
  [
    [
      "HP:0000089",
```

"HP:0000107",
"HP:0000217",
"HP:0001407",
"HP:0001785",
"HP:0001962",
"HP:0002015",
"HP:0002018",
"HP:0002020",
"HP:0002027",
"HP:0002036",
"HP:0002110",
"HP:0002321",
"HP:0003138",
"HP:0003165",
"HP:0003236",
"HP:0003259",
"HP:0003418",
"HP:0003565",
"HP:0006682",
"HP:0009098",
"HP:0011227",
"HP:0011897",
"HP:0012213",
"HP:0012378",
"HP:0012387",
"HP:0012514",
"HP:0025179",
"HP:0025392",
"HP:0030057",
"HP:0030830",
"HP:0030877",
"HP:0032017",
"HP:0045051",
"HP:0100643",
"HP:0410019"
],
[
"OMIM:270150",
"ORPHA:289390"
]
],
[
[
"HP:0000271",
"HP:0000975",
"HP:0000980",
"HP:0001279",
"HP:0001824",
"HP:0001974",
"HP:0002013",
"HP:0002018",
"HP:0002315",
"HP:0003546",
"HP:0003565",
"HP:0012378",
"HP:0012531",

```
      "HP:0025143",
      "HP:0030164"
    ],
    [
      "OMIM:187360",
      "ORPHA:397"
    ]
  ],
  [
    [
      "HP:0000540",
      "HP:0000554",
      "HP:0001097",
      "HP:0001443",
      "HP:0002808",
      "HP:0002907",
      "HP:0003418",
      "HP:0005197",
      "HP:0100512"
    ],
    [
      "OMIM:106300",
      "OMIM:183840"
    ]
  ],
  [
    [
      "HP:0000083",
      "HP:0000366",
      "HP:0000989",
      "HP:0001639",
      "HP:0001644",
      "HP:0001698",
      "HP:0001712",
      "HP:0001945",
      "HP:0002094",
      "HP:0010876",
      "HP:0012213",
      "HP:0012378",
      "HP:0012664",
      "HP:0012735",
      "HP:0012819",
      "HP:0032017"
    ],
    [
      "OMIM:207600",
      "ORPHA:3287"
    ]
  ],
  [
    [
      "HP:0000012",
      "HP:0000246",
      "HP:0001649",
      "HP:0001945",
      "HP:0001974",
```

```
      "HP:0002105",
      "HP:0002202",
      "HP:0003115",
      "HP:0005110",
      "HP:0011227",
      "HP:0012735",
      "HP:0020181",
      "HP:0025392",
      "HP:0100534",
      "HP:0100749"
    ],
    [
      "OMIM:608710",
      "ORPHA:900"
    ]
  ],
  [
    [
      "HP:0000217",
      "HP:0000633",
      "HP:0001167",
      "HP:0001225",
      "HP:0001760",
      "HP:0001935",
      "HP:0002076",
      "HP:0002815",
      "HP:0002829",
      "HP:0002923",
      "HP:0003212",
      "HP:0003237",
      "HP:0003326",
      "HP:0003394",
      "HP:0003493",
      "HP:0004386",
      "HP:0005197",
      "HP:0005263",
      "HP:0011911",
      "HP:0030836",
      "HP:0030839",
      "HP:0030840",
      "HP:0032235",
      "HP:0032308"
    ],
    [
      "OMIM:270150",
      "OMIM:604302",
      "ORPHA:289390",
      "ORPHA:29207",
      "ORPHA:85408",
      "ORPHA:85414",
      "ORPHA:85435"
    ]
  ],
  [
    [
      "HP:0000097",
```

"HP:0001065",
"HP:0002149",
"HP:0002155",
"HP:0002907",
"HP:0002910",
"HP:0003073",
"HP:0003124",
"HP:0003138",
"HP:0003149",
"HP:0003565",
"HP:0005413",
"HP:0007390",
"HP:0009741",
"HP:0010783",
"HP:0010931",
"HP:0011227",
"HP:0012213",
"HP:0012595",
"HP:0012611",
"HP:0100515"
],
[
"OMIM:603278",
"OMIM:603965",
"OMIM:607832",
"OMIM:612551",
"OMIM:613237",
"OMIM:614131",
"OMIM:616002",
"OMIM:616032",
"OMIM:616220"
]
],
[
[
"HP:0000155",
"HP:0000246",
"HP:0000433",
"HP:0001894",
"HP:0001945",
"HP:0001974",
"HP:0002094",
"HP:0002815",
"HP:0003155",
"HP:0003565",
"HP:0005413",
"HP:0011227",
"HP:0012219",
"HP:0025289",
"HP:0030948",
"HP:0032118",
"HP:0032154",
"HP:0100721",
"HP:0100749"
],
[

"OMIM:109650",
"ORPHA:117"
]
],
[
[
"HP:0000083",
"HP:0000099",
"HP:0000246",
"HP:0000366",
"HP:0000509",
"HP:0001945",
"HP:0002105",
"HP:0003546",
"HP:0012735",
"HP:0030834",
"HP:0030835",
"HP:0030839",
"HP:0040223",
"HP:0100520"
],
[
"OMIM:608710",
"ORPHA:900"
]
],
[
[
"HP:0000083",
"HP:0000107",
"HP:0000572",
"HP:0000822",
"HP:0000979",
"HP:0001397",
"HP:0001407",
"HP:0001970",
"HP:0002018",
"HP:0002088",
"HP:0002094",
"HP:0002315",
"HP:0002907",
"HP:0009741",
"HP:0012085",
"HP:0012378",
"HP:0012398",
"HP:0012461",
"HP:0012594",
"HP:0012735",
"HP:0031812",
"HP:0100721"
],
[
"OMIM:181000",
"OMIM:612387",
"ORPHA:797"
]

],
[
[
"HP:0000100",
"HP:0000366",
"HP:0000790",
"HP:0000793",
"HP:0000979",
"HP:0001225",
"HP:0002094",
"HP:0002155",
"HP:0003124",
"HP:0004324",
"HP:0010741",
"HP:0012398",
"HP:0012596",
"HP:0012597",
"HP:0031504"
],
[
"ORPHA:761"
]
],
[
[
"HP:0000164",
"HP:0001596",
"HP:0002659",
"HP:0003282",
"HP:0004322",
"HP:0005268",
"HP:0012378",
"HP:0012514",
"HP:0031456"
],
[
"CCRD:50",
"OMIM:146300",
"ORPHA:247676",
"ORPHA:436"
]
],
[
[
"HP:0000265",
"HP:0000365",
"HP:0000666",
"HP:0000858",
"HP:0001649",
"HP:0001785",
"HP:0001824",
"HP:0001962",
"HP:0002321",
"HP:0005421",
"HP:0010628",
"HP:0030057",

```
      "HP:0045042",
      "HP:0200097"
    ],
    [
      "OMIM:181000",
      "OMIM:612387",
      "ORPHA:797"
    ]
  ],
  [
    [
      "HP:0000246",
      "HP:0000520",
      "HP:0000651",
      "HP:0000822",
      "HP:0001144",
      "HP:0001653",
      "HP:0001659",
      "HP:0001712",
      "HP:0002155",
      "HP:0002240",
      "HP:0002896",
      "HP:0003124",
      "HP:0005180",
      "HP:0012213",
      "HP:0012246",
      "HP:0032300",
      "HP:0100540"
    ],
    [
      "CCRD:56",
      "OMIM:228800",
      "ORPHA:284264",
      "ORPHA:49041"
    ]
  ],
  [
    [
      "HP:0000365",
      "HP:0001649",
      "HP:0001744",
      "HP:0001945",
      "HP:0002153",
      "HP:0002321",
      "HP:0002850",
      "HP:0003072",
      "HP:0003115",
      "HP:0003565",
      "HP:0005339",
      "HP:0011227",
      "HP:0011788",
      "HP:0025238",
      "HP:0030835",
      "HP:0030836",
      "HP:0030838",
      "HP:0030839"
```

```
    ],
    [
      "OMIM:120100",
      "OMIM:191900",
      "OMIM:607115",
      "OMIM:611762",
      "OMIM:614468",
      "OMIM:616115",
      "ORPHA:1451",
      "ORPHA:247868",
      "ORPHA:300359",
      "ORPHA:47045",
      "ORPHA:575"
    ]
  ],
  [
    [
      "HP:0000010",
      "HP:0000230",
      "HP:0000282",
      "HP:0000421",
      "HP:0000975",
      "HP:0001600",
      "HP:0001824",
      "HP:0001880",
      "HP:0001935",
      "HP:0002105",
      "HP:0002113",
      "HP:0002153",
      "HP:0002875",
      "HP:0003546",
      "HP:0008221",
      "HP:0010287",
      "HP:0010628",
      "HP:0011227",
      "HP:0012378",
      "HP:0012398",
      "HP:0025289",
      "HP:0025392",
      "HP:0030001",
      "HP:0030835",
      "HP:0030839",
      "HP:0031098",
      "HP:0031292",
      "HP:0032230",
      "HP:0100631"
    ],
    [
      "OMIM:181000",
      "OMIM:612387",
      "ORPHA:797"
    ]
  ],
  [
    [
      "HP:0000036",
```

"HP:0000155",
"HP:0000421",
"HP:0000554",
"HP:0000572",
"HP:0000790",
"HP:0000822",
"HP:0001369",
"HP:0001785",
"HP:0002027",
"HP:0002380",
"HP:0002829",
"HP:0002910",
"HP:0002922",
"HP:0003236",
"HP:0003259",
"HP:0003326",
"HP:0003394",
"HP:0003416",
"HP:0003418",
"HP:0003474",
"HP:0007042",
"HP:0009027",
"HP:0010280",
"HP:0011911",
"HP:0012378",
"HP:0012534",
"HP:0025435",
"HP:0030948",
"HP:0032101",
"HP:0032154",
"HP:0100518",
"HP:0410019"
],
[
"OMIM:109650",
"ORPHA:117"
]
],
[
[
"HP:0000989",
"HP:0001649",
"HP:0001785",
"HP:0001824",
"HP:0001888",
"HP:0001903",
"HP:0001945",
"HP:0001974",
"HP:0002815",
"HP:0003073",
"HP:0003115",
"HP:0003236",
"HP:0003326",
"HP:0003458",
"HP:0003493",
"HP:0003546",

```
      "HP:0003565",
      "HP:0005197",
      "HP:0005413",
      "HP:0006163",
      "HP:0010783",
      "HP:0011227",
      "HP:0011897",
      "HP:0012514",
      "HP:0012735",
      "HP:0025131",
      "HP:0030167",
      "HP:0032232",
      "HP:0100614",
      "HP:0100643",
      "HP:0410174"
    ],
    [
      "ORPHA:81"
    ]
  ],
  [
    [
      "HP:0000961",
      "HP:0001397",
      "HP:0001880",
      "HP:0002113",
      "HP:0002716",
      "HP:0002910",
      "HP:0003124",
      "HP:0003165",
      "HP:0003212",
      "HP:0011799",
      "HP:0030057",
      "HP:0030948"
    ],
    [
      "ORPHA:482"
    ]
  ],
  [
    [
      "HP:0000246",
      "HP:0001880",
      "HP:0002094",
      "HP:0002113",
      "HP:0003212",
      "HP:0025392",
      "HP:0030057",
      "HP:0100582"
    ],
    [
      "ORPHA:183"
    ]
  ],
  [
    [
```

"HP:0000155",
"HP:0000853",
"HP:0001369",
"HP:0001600",
"HP:0001903",
"HP:0001962",
"HP:0002020",
"HP:0002315",
"HP:0002380",
"HP:0002385",
"HP:0002815",
"HP:0002829",
"HP:0003326",
"HP:0003445",
"HP:0003474",
"HP:0003493",
"HP:0005421",
"HP:0006261",
"HP:0010876",
"HP:0011712",
"HP:0011717",
"HP:0011911",
"HP:0012513",
"HP:0012534",
"HP:0030833",
"HP:0030835",
"HP:0030837",
"HP:0030839",
"HP:0031731",
"HP:0032154",
"HP:0100643",
"HP:0410281"
],
[
"ORPHA:81"
]
],
[
[
"HP:0000245",
"HP:0000421",
"HP:0000433",
"HP:0002105",
"HP:0025095",
"HP:0030835",
"HP:0030839",
"HP:0030840"
],
[
"OMIM:608710",
"ORPHA:900"
]
],
[
[
"HP:0001397",

```
        "HP:0001945",
        "HP:0001974",
        "HP:0002020",
        "HP:0002240",
        "HP:0002896",
        "HP:0002910",
        "HP:0003418",
        "HP:0003546",
        "HP:0005165",
        "HP:0005197",
        "HP:0008775",
        "HP:0011227",
        "HP:0012317",
        "HP:0012378",
        "HP:0030830",
        "HP:0030835",
        "HP:0030839",
        "HP:0030948",
        "HP:0031352",
        "HP:0045051",
        "HP:0045073",
        "HP:0100749",
        "HP:0200041"
      ],
      [
        "OMIM:106300",
        "OMIM:183840"
      ]
    ],
    [
      [
        "HP:0001974",
        "HP:0002754",
        "HP:0003565",
        "HP:0011227",
        "HP:0030793",
        "HP:0040264",
        "HP:0100774",
        "HP:0100847"
      ],
      [
        "ORPHA:793"
      ]
    ],
    [
      [
        "HP:0002814",
        "HP:0002907",
        "HP:0003236",
        "HP:0003401",
        "HP:0003565",
        "HP:0008151",
        "HP:0011227",
        "HP:0012733",
        "HP:0045042",
        "HP:0200041"
```

```
      ],
      [
        "OMIM:615688",
        "ORPHA:404553",
        "ORPHA:767"
      ]
    ],
    [
      [
        "HP:0001824",
        "HP:0001882",
        "HP:0002094",
        "HP:0002321",
        "HP:0003236",
        "HP:0003546",
        "HP:0025179",
        "HP:0025238",
        "HP:0030057",
        "HP:0045051"
      ],
      [
        "ORPHA:81"
      ]
    ],
    [
      [
        "HP:0000072",
        "HP:0001744",
        "HP:0003259",
        "HP:0004942",
        "HP:0005200",
        "HP:0008714",
        "HP:0011227",
        "HP:0030150",
        "HP:0030423",
        "HP:0032300",
        "HP:0100581"
      ],
      [
        "ORPHA:49041"
      ]
    ],
    [
      [
        "HP:0000017",
        "HP:0001600",
        "HP:0001824",
        "HP:0001873",
        "HP:0001903",
        "HP:0001945",
        "HP:0001974",
        "HP:0002315",
        "HP:0002923",
        "HP:0003165",
        "HP:0003262",
        "HP:0003326",
```

```
      "HP:0005339",
      "HP:0010931",
      "HP:0011227",
      "HP:0012213",
      "HP:0012514",
      "HP:0012664",
      "HP:0030155",
      "HP:0030166",
      "HP:0030948",
      "HP:0031520",
      "HP:0032300",
      "HP:0100512",
      "HP:0100614"
    ],
    [
      "ORPHA:732"
    ]
  ],
  [
    [
      "HP:0000010",
      "HP:0001155",
      "HP:0001225",
      "HP:0001443",
      "HP:0001882",
      "HP:0001935",
      "HP:0001943",
      "HP:0002315",
      "HP:0002321",
      "HP:0003401",
      "HP:0003416",
      "HP:0003418",
      "HP:0003474",
      "HP:0003565",
      "HP:0005197",
      "HP:0005421",
      "HP:0006979",
      "HP:0011227",
      "HP:0012317",
      "HP:0012513",
      "HP:0025230",
      "HP:0030834",
      "HP:0030836",
      "HP:0030839",
      "HP:0031185",
      "HP:0100686"
    ],
    [
      "OMIM:106300",
      "OMIM:183840"
    ]
  ],
  [
    [
      "HP:0000103",
      "HP:0001061",
```

```
      "HP:0001959",
      "HP:0002315",
      "HP:0002815",
      "HP:0002829",
      "HP:0003236",
      "HP:0005197",
      "HP:0011911",
      "HP:0012378",
      "HP:0025238",
      "HP:0030835",
      "HP:0030836",
      "HP:0030839",
      "HP:0030841",
      "HP:0032300"
    ],
    [
      "CCRD:28",
      "OMIM:134610",
      "OMIM:249100",
      "OMIM:604302",
      "ORPHA:29207",
      "ORPHA:342",
      "ORPHA:85408",
      "ORPHA:85414",
      "ORPHA:85435"
    ]
  ],
  [
    [
      "HP:0000155",
      "HP:0000651",
      "HP:0001167",
      "HP:0001279",
      "HP:0001369",
      "HP:0001945",
      "HP:0001974",
      "HP:0002315",
      "HP:0002907",
      "HP:0003043",
      "HP:0003249",
      "HP:0003326",
      "HP:0003401",
      "HP:0003418",
      "HP:0003474",
      "HP:0003546",
      "HP:0003565",
      "HP:0005413",
      "HP:0011227",
      "HP:0011706",
      "HP:0012378",
      "HP:0012574",
      "HP:0012595",
      "HP:0030837",
      "HP:0032154",
      "HP:0032308"
    ],
```

[
"CCRD:28",
"OMIM:109650",
"OMIM:134610",
"OMIM:249100",
"ORPHA:117",
"ORPHA:342"
]
],
[
[
"HP:0000365",
"HP:0000421",
"HP:0001288",
"HP:0001945",
"HP:0003326",
"HP:0011227",
"HP:0012378",
"HP:0012514",
"HP:0030766",
"HP:0032230"
],
[
"OMIM:608710",
"ORPHA:900"
]
],
[
[
"HP:0000083",
"HP:0000093",
"HP:0000099",
"HP:0000141",
"HP:0000790",
"HP:0001369",
"HP:0001744",
"HP:0001935",
"HP:0002015",
"HP:0002094",
"HP:0002240",
"HP:0003493",
"HP:0003645",
"HP:0004326",
"HP:0012213",
"HP:0012398",
"HP:0031504",
"HP:0032235",
"HP:0045073",
"HP:0100643"
],
[
"OMIM:152700",
"OMIM:606579",
"OMIM:609939",
"OMIM:614420",
"ORPHA:300345",

"ORPHA:809",
"ORPHA:93552"
]
],
[
[
"HP:0000103",
"HP:0000572",
"HP:0000790",
"HP:0000958",
"HP:0001097",
"HP:0001337",
"HP:0002014",
"HP:0002019",
"HP:0002046",
"HP:0002076",
"HP:0002875",
"HP:0003326",
"HP:0003546",
"HP:0011123",
"HP:0012378",
"HP:0012735",
"HP:0030972",
"HP:0032300",
"HP:0410281"
],
[
"CCRD:56",
"OMIM:228800",
"ORPHA:284264",
"ORPHA:49041"
]
],
[
[
"HP:0000246",
"HP:0000366",
"HP:0000388",
"HP:0001945",
"HP:0002315",
"HP:0003546",
"HP:0030835",
"HP:0030839"
],
[
"OMIM:608710",
"ORPHA:900"
]
],
[
[
"HP:0000772",
"HP:0000939",
"HP:0002650",
"HP:0002829",
"HP:0003124",

"HP:0003282",
"HP:0003326",
"HP:0003394",
"HP:0003418",
"HP:0003493",
"HP:0008422",
"HP:0012184",
"HP:0030839",
"HP:0030840"
],
[
"CCRD:50",
"OMIM:146300",
"ORPHA:247676",
"ORPHA:436"
]
],
[
[
"HP:0000089",
"HP:0000097",
"HP:0000121",
"HP:0001397",
"HP:0001407",
"HP:0001744",
"HP:0001873",
"HP:0002014",
"HP:0002149",
"HP:0002153",
"HP:0003043",
"HP:0003138",
"HP:0003149",
"HP:0003165",
"HP:0003259",
"HP:0003281",
"HP:0003493",
"HP:0003565",
"HP:0005339",
"HP:0012185",
"HP:0012213",
"HP:0012513",
"HP:0012514",
"HP:0012611",
"HP:0020181",
"HP:0025020",
"HP:0030833",
"HP:0030834",
"HP:0030873",
"HP:0100529"
],
[
"OMIM:181750",
"ORPHA:90290"
]
],
[

```
    [
      "HP:0000246",
      "HP:0000433",
      "HP:0000822",
      "HP:0000853",
      "HP:0001653",
      "HP:0001785",
      "HP:0001824",
      "HP:0001945",
      "HP:0002875",
      "HP:0003324",
      "HP:0003493",
      "HP:0003546",
      "HP:0004930",
      "HP:0010741",
      "HP:0011227",
      "HP:0012378",
      "HP:0012398",
      "HP:0012735",
      "HP:0025179",
      "HP:0030166",
      "HP:0030839",
      "HP:0030841",
      "HP:0031394",
      "HP:0100827"
    ],
    [
      "ORPHA:81"
    ]
  ],
  [
    [
      "HP:0001025",
      "HP:0001369",
      "HP:0001945",
      "HP:0003115",
      "HP:0005115",
      "HP:0008619",
      "HP:0011227",
      "HP:0025269",
      "HP:0030839"
    ],
    [
      "OMIM:120100",
      "OMIM:142680",
      "OMIM:191900",
      "OMIM:607115",
      "OMIM:611762",
      "OMIM:614468",
      "OMIM:616115",
      "ORPHA:1451",
      "ORPHA:247868",
      "ORPHA:300359",
      "ORPHA:32960",
      "ORPHA:47045",
      "ORPHA:575"
```

```
    ]
  ],
  [
    [
      "HP:0001063",
      "HP:0001780",
      "HP:0001945",
      "HP:0002240",
      "HP:0002625",
      "HP:0002633",
      "HP:0003419",
      "HP:0004326",
      "HP:0004420",
      "HP:0008940",
      "HP:0009098",
      "HP:0010287",
      "HP:0010783",
      "HP:0012378",
      "HP:0012398",
      "HP:0025203",
      "HP:0025238",
      "HP:0030242",
      "HP:0100758"
    ],
    [
      "OMIM:211480",
      "OMIM:615688",
      "ORPHA:36258",
      "ORPHA:404553",
      "ORPHA:767"
    ]
  ],
  [
    [
      "HP:0000421",
      "HP:0001824",
      "HP:0002076",
      "HP:0002321",
      "HP:0002633",
      "HP:0003326",
      "HP:0003546",
      "HP:0003565",
      "HP:0030766",
      "HP:0030833",
      "HP:0100546"
    ],
    [
      "OMIM:207600",
      "ORPHA:3287"
    ]
  ],
  [
    [
      "HP:0000083",
      "HP:0000155",
      "HP:0000366",
```

```
      "HP:0000377",
      "HP:0000766",
      "HP:0001041",
      "HP:0001635",
      "HP:0001649",
      "HP:0001659",
      "HP:0001962",
      "HP:0002149",
      "HP:0002202",
      "HP:0002716",
      "HP:0002907",
      "HP:0003149",
      "HP:0003326",
      "HP:0003418",
      "HP:0003546",
      "HP:0003565",
      "HP:0011227",
      "HP:0011713",
      "HP:0011911",
      "HP:0012317",
      "HP:0012378",
      "HP:0012611",
      "HP:0025238",
      "HP:0030835",
      "HP:0030840",
      "HP:0032154",
      "HP:0100686"
    ],
    [
      "ORPHA:728"
    ]
  ],
  [
    [
      "HP:0001138",
      "HP:0002910",
      "HP:0003124",
      "HP:0003141",
      "HP:0007042",
      "HP:0007663",
      "HP:0012756",
      "HP:0025343",
      "HP:0030833",
      "HP:0030948",
      "HP:0200149"
    ],
    [
      "OMIM:107320"
    ]
  ],
  [
    [
      "HP:0001824",
      "HP:0002829",
      "HP:0003259",
      "HP:0003493",
```

```
      "HP:0003546",
      "HP:0012378",
      "HP:0012452",
      "HP:0100643"
    ],
    [
      "CCRD:112",
      "OMIM:181750",
      "ORPHA:220393",
      "ORPHA:220402",
      "ORPHA:90290",
      "ORPHA:90291"
    ]
  ],
  [
    [
      "HP:0000083",
      "HP:0000093",
      "HP:0000100",
      "HP:0000790",
      "HP:0000793",
      "HP:0000822",
      "HP:0000979",
      "HP:0001744",
      "HP:0001785",
      "HP:0001942",
      "HP:0002018",
      "HP:0002027",
      "HP:0002094",
      "HP:0002101",
      "HP:0002113",
      "HP:0002202",
      "HP:0002315",
      "HP:0003073",
      "HP:0003259",
      "HP:0003546",
      "HP:0004315",
      "HP:0004930",
      "HP:0005421",
      "HP:0008940",
      "HP:0011227",
      "HP:0025346",
      "HP:0030840",
      "HP:0031185",
      "HP:0032177",
      "HP:0045042",
      "HP:0100778"
    ],
    [
      "OMIM:123550",
      "ORPHA:91138"
    ]
  ],
  [
    [
      "HP:0000388",
```

      "HP:0000961",
      "HP:0001698",
      "HP:0001780",
      "HP:0002202",
      "HP:0002829",
      "HP:0009830",
      "HP:0011025",
      "HP:0100758"
    ],
    [
      "OMIM:608710",
      "ORPHA:900"
    ]
  ],
  [
    [
      "HP:0000821",
      "HP:0000989",
      "HP:0001081",
      "HP:0001575",
      "HP:0001644",
      "HP:0001662",
      "HP:0001735",
      "HP:0001824",
      "HP:0001972",
      "HP:0001974",
      "HP:0002018",
      "HP:0002371",
      "HP:0002546",
      "HP:0002633",
      "HP:0002870",
      "HP:0002925",
      "HP:0003115",
      "HP:0003165",
      "HP:0003493",
      "HP:0003565",
      "HP:0011227",
      "HP:0011709",
      "HP:0030948",
      "HP:0031588",
      "HP:0032210",
      "HP:0032229",
      "HP:0032300",
      "HP:0100546",
      "HP:0100646"
    ],
    [
      "CCRD:56",
      "OMIM:228800",
      "ORPHA:284264",
      "ORPHA:49041"
    ]
  ],
  [
    [
      "HP:0001155",

```
      "HP:0001824",
      "HP:0002910",
      "HP:0002923",
      "HP:0003281",
      "HP:0003565",
      "HP:0005339",
      "HP:0005413",
      "HP:0006248",
      "HP:0011227",
      "HP:0012513",
      "HP:0012534",
      "HP:0030833",
      "HP:0100545",
      "HP:0100778",
      "HP:0200041"
    ],
    [
      "OMIM:123550",
      "ORPHA:91138"
    ]
  ],
  [
    [
      "HP:0000967",
      "HP:0001873",
      "HP:0001974",
      "HP:0002315",
      "HP:0002633",
      "HP:0002910",
      "HP:0003262",
      "HP:0003493",
      "HP:0003565",
      "HP:0005421",
      "HP:0006562",
      "HP:0009830",
      "HP:0010741",
      "HP:0011227",
      "HP:0012378",
      "HP:0012514",
      "HP:0025238",
      "HP:0030167",
      "HP:0045042",
      "HP:0100643",
      "HP:0100778"
    ],
    [
      "OMIM:123550",
      "ORPHA:91138"
    ]
  ],
  [
    [
      "HP:0000031",
      "HP:0000509",
      "HP:0001701",
      "HP:0001744",
```

```
      "HP:0001824",
      "HP:0001945",
      "HP:0001974",
      "HP:0002014",
      "HP:0002027",
      "HP:0002815",
      "HP:0003073",
      "HP:0003216",
      "HP:0003259",
      "HP:0003565",
      "HP:0010783",
      "HP:0011227",
      "HP:0011956",
      "HP:0012378",
      "HP:0012596",
      "HP:0012597",
      "HP:0030166",
      "HP:0030838",
      "HP:0100016",
      "HP:0100539"
    ],
    [
      "OMIM:142680",
      "ORPHA:32960"
    ]
  ],
  [
    [
      "HP:0000138",
      "HP:0001397",
      "HP:0001744",
      "HP:0001945",
      "HP:0002829",
      "HP:0002875",
      "HP:0003326",
      "HP:0003546",
      "HP:0011227",
      "HP:0030166"
    ],
    [
      "CCRD:28",
      "OMIM:134610",
      "OMIM:249100",
      "ORPHA:342"
    ]
  ],
  [
    [
      "HP:0000017",
      "HP:0000107",
      "HP:0000138",
      "HP:0000975",
      "HP:0001041",
      "HP:0001386",
      "HP:0001397",
      "HP:0001541",
```

"HP:0001649",
"HP:0001735",
"HP:0001780",
"HP:0002013",
"HP:0002014",
"HP:0002018",
"HP:0002059",
"HP:0002094",
"HP:0002102",
"HP:0002149",
"HP:0002155",
"HP:0002202",
"HP:0002240",
"HP:0002829",
"HP:0002896",
"HP:0002923",
"HP:0003124",
"HP:0003149",
"HP:0003281",
"HP:0003326",
"HP:0003418",
"HP:0003496",
"HP:0003546",
"HP:0003573",
"HP:0005197",
"HP:0005339",
"HP:0006163",
"HP:0009726",
"HP:0009830",
"HP:0011227",
"HP:0012611",
"HP:0025346",
"HP:0030833",
"HP:0030834",
"HP:0030837",
"HP:0030948",
"HP:0031226",
"HP:0031520",
"HP:0032300",
"HP:0100512",
"HP:0100643",
"HP:0100785",
"HP:0410367"
],
[
"ORPHA:81"
]
],
[
[
"HP:0000093",
"HP:0000245",
"HP:0000377",
"HP:0000433",
"HP:0000790",
"HP:0001063",

```
        "HP:0001785",
        "HP:0001903",
        "HP:0002910",
        "HP:0003259",
        "HP:0011227",
        "HP:0012213",
        "HP:0032230"
      ],
      [
        "OMIM:608710",
        "ORPHA:900"
      ]
    ],
    [
      [
        "HP:0000155",
        "HP:0000217",
        "HP:0000360",
        "HP:0000365",
        "HP:0000520",
        "HP:0000572",
        "HP:0000633",
        "HP:0000651",
        "HP:0000787",
        "HP:0001167",
        "HP:0001269",
        "HP:0001288",
        "HP:0001369",
        "HP:0001653",
        "HP:0001947",
        "HP:0001974",
        "HP:0002076",
        "HP:0002321",
        "HP:0002829",
        "HP:0002875",
        "HP:0002925",
        "HP:0003474",
        "HP:0003537",
        "HP:0003546",
        "HP:0011935",
        "HP:0030833",
        "HP:0032154",
        "HP:0100512",
        "HP:0200037",
        "HP:0200039"
      ],
      [
        "OMIM:106300",
        "OMIM:109650",
        "OMIM:183840",
        "ORPHA:117"
      ]
    ],
    [
      [
        "HP:0000246",
```

```
      "HP:0000822",
      "HP:0000958",
      "HP:0000980",
      "HP:0001894",
      "HP:0001903",
      "HP:0001920",
      "HP:0001945",
      "HP:0001974",
      "HP:0002013",
      "HP:0002027",
      "HP:0002094",
      "HP:0002754",
      "HP:0002910",
      "HP:0003138",
      "HP:0003237",
      "HP:0003259",
      "HP:0003418",
      "HP:0003493",
      "HP:0003546",
      "HP:0003565",
      "HP:0004396",
      "HP:0011097",
      "HP:0011227",
      "HP:0030157",
      "HP:0030948",
      "HP:0031030",
      "HP:0032483",
      "HP:0100016",
      "HP:0200034",
      "HP:0410019"
    ],
    [
      "OMIM:207600",
      "ORPHA:3287"
    ]
  ],
  [
    [
      "HP:0001397",
      "HP:0002385",
      "HP:0002817",
      "HP:0002910",
      "HP:0003115",
      "HP:0003236",
      "HP:0003259",
      "HP:0003326",
      "HP:0003493",
      "HP:0005110",
      "HP:0005145",
      "HP:0008978",
      "HP:0011713",
      "HP:0012213",
      "HP:0012596",
      "HP:0012597",
      "HP:0030167",
      "HP:0030948",
```

```
      "HP:0031185",
      "HP:0100545"
    ],
    [
      "OMIM:613806",
      "ORPHA:171",
      "ORPHA:732"
    ]
  ],
  [
    [
      "HP:0000364",
      "HP:0000572",
      "HP:0000822",
      "HP:0000975",
      "HP:0001288",
      "HP:0001397",
      "HP:0001575",
      "HP:0001659",
      "HP:0001744",
      "HP:0002014",
      "HP:0002018",
      "HP:0002019",
      "HP:0002046",
      "HP:0002242",
      "HP:0002315",
      "HP:0002321",
      "HP:0002814",
      "HP:0002829",
      "HP:0002875",
      "HP:0002910",
      "HP:0003115",
      "HP:0003141",
      "HP:0003236",
      "HP:0003270",
      "HP:0003281",
      "HP:0003418",
      "HP:0003474",
      "HP:0003546",
      "HP:0004386",
      "HP:0004756",
      "HP:0005263",
      "HP:0006682",
      "HP:0011227",
      "HP:0011712",
      "HP:0011911",
      "HP:0012185",
      "HP:0012317",
      "HP:0012513",
      "HP:0012514",
      "HP:0012534",
      "HP:0025269",
      "HP:0030833",
      "HP:0030838",
      "HP:0031520",
      "HP:0031588",
```

```
      "HP:0100512",
      "HP:0100749",
      "HP:0100785"
    ],
    [
      "OMIM:106300",
      "OMIM:183840"
    ]
  ],
  [
    [
      "HP:0000853",
      "HP:0001081",
      "HP:0001369",
      "HP:0001600",
      "HP:0001679",
      "HP:0001894",
      "HP:0001903",
      "HP:0001945",
      "HP:0001974",
      "HP:0002240",
      "HP:0002242",
      "HP:0002829",
      "HP:0002910",
      "HP:0003072",
      "HP:0003155",
      "HP:0003326",
      "HP:0003546",
      "HP:0005413",
      "HP:0011227",
      "HP:0025022",
      "HP:0025289",
      "HP:0030166",
      "HP:0030839",
      "HP:0030948"
    ],
    [
      "OMIM:120100",
      "OMIM:191900",
      "OMIM:607115",
      "OMIM:611762",
      "OMIM:614468",
      "OMIM:616115",
      "ORPHA:1451",
      "ORPHA:247868",
      "ORPHA:300359",
      "ORPHA:47045",
      "ORPHA:575"
    ]
  ],
  [
    [
      "HP:0000083",
      "HP:0000089",
      "HP:0000097",
      "HP:0000821",
```

```
      "HP:0001903",
      "HP:0002014",
      "HP:0002153",
      "HP:0003073",
      "HP:0003138",
      "HP:0003165",
      "HP:0003259",
      "HP:0012213",
      "HP:0012595",
      "HP:0100512",
      "HP:0200034"
    ],
    [
      "CCRD:27",
      "OMIM:301500",
      "ORPHA:324"
    ]
  ],
  [
    [
      "HP:0000736",
      "HP:0001873",
      "HP:0001981",
      "HP:0002172",
      "HP:0002321",
      "HP:0002910",
      "HP:0003138",
      "HP:0003259",
      "HP:0003546",
      "HP:0007185",
      "HP:0008330",
      "HP:0012213",
      "HP:0020181",
      "HP:0025435",
      "HP:0032308",
      "HP:0100661"
    ],
    [
      "OMIM:274150",
      "ORPHA:54057",
      "ORPHA:93583"
    ]
  ],
  [
    [
      "HP:0000869",
      "HP:0001629",
      "HP:0001659",
      "HP:0001744",
      "HP:0001824",
      "HP:0001894",
      "HP:0001935",
      "HP:0001945",
      "HP:0001970",
      "HP:0002094",
      "HP:0002829",
```

```
      "HP:0003138",
      "HP:0003546",
      "HP:0003565",
      "HP:0005259",
      "HP:0011712",
      "HP:0012122",
      "HP:0012213",
      "HP:0012513",
      "HP:0012595",
      "HP:0012735",
      "HP:0030157",
      "HP:0030833",
      "HP:0031664",
      "HP:0100515",
      "HP:0100584"
    ],
    [
      "OMIM:607665",
      "ORPHA:91500"
    ]
  ],
  [
    [
      "HP:0000545",
      "HP:0000651",
      "HP:0000958",
      "HP:0001155",
      "HP:0001265",
      "HP:0001760",
      "HP:0001875",
      "HP:0002570",
      "HP:0002781",
      "HP:0002829",
      "HP:0002883",
      "HP:0003236",
      "HP:0006536",
      "HP:0007210",
      "HP:0012513",
      "HP:0012534",
      "HP:0025238",
      "HP:0040126",
      "HP:0100827",
      "HP:0410281"
    ],
    [
      "OMIM:133020",
      "ORPHA:90026"
    ]
  ],
  [
    [
      "HP:0000093",
      "HP:0000142",
      "HP:0000164",
      "HP:0000246",
      "HP:0000433",
```

"HP:0000713",
"HP:0000822",
"HP:0001041",
"HP:0001288",
"HP:0001384",
"HP:0001596",
"HP:0001600",
"HP:0001785",
"HP:0001945",
"HP:0001959",
"HP:0001962",
"HP:0002019",
"HP:0002020",
"HP:0002027",
"HP:0002315",
"HP:0002321",
"HP:0002829",
"HP:0002875",
"HP:0002923",
"HP:0003419",
"HP:0003474",
"HP:0003493",
"HP:0005197",
"HP:0006253",
"HP:0006256",
"HP:0007042",
"HP:0010741",
"HP:0011911",
"HP:0012085",
"HP:0012213",
"HP:0012219",
"HP:0012378",
"HP:0012479",
"HP:0012514",
"HP:0012534",
"HP:0030157",
"HP:0030166",
"HP:0030269",
"HP:0030837",
"HP:0030838",
"HP:0030899",
"HP:0031284",
"HP:0031352",
"HP:0031520",
"HP:0040264",
"HP:0100515",
"HP:0100540",
"HP:0100749",
"HP:0410019"
],
[
"CCRD:112",
"OMIM:181750",
"ORPHA:220393",
"ORPHA:220402",
"ORPHA:732",

"ORPHA:801",
"ORPHA:90289",
"ORPHA:90290",
"ORPHA:90291"
]
],
[
[
"HP:0000217",
"HP:0000505",
"HP:0001824",
"HP:0001945",
"HP:0002907",
"HP:0002910",
"HP:0003418",
"HP:0003546",
"HP:0003565",
"HP:0004396",
"HP:0010783",
"HP:0011227",
"HP:0011801",
"HP:0012122",
"HP:0012577",
"HP:0012595",
"HP:0025289",
"HP:0030166",
"HP:0200026"
],
[
"OMIM:181000",
"OMIM:612387",
"ORPHA:797"
]
],
[
[
"HP:0000155",
"HP:0002315",
"HP:0002354",
"HP:0002754",
"HP:0002758",
"HP:0002808",
"HP:0002870",
"HP:0003028",
"HP:0003418",
"HP:0008419",
"HP:0030840",
"HP:0032154",
"HP:0200039"
],
[
"ORPHA:793"
]
],
[
[

```
      "HP:0000098",
      "HP:0000107",
      "HP:0000138",
      "HP:0000501",
      "HP:0000545",
      "HP:0001167",
      "HP:0001761",
      "HP:0001824",
      "HP:0002615",
      "HP:0002619",
      "HP:0002763",
      "HP:0002829",
      "HP:0002857",
      "HP:0002875",
      "HP:0002907",
      "HP:0004386",
      "HP:0005086",
      "HP:0005108",
      "HP:0005263",
      "HP:0007663",
      "HP:0010741",
      "HP:0030835",
      "HP:0030838",
      "HP:0030839",
      "HP:0031520",
      "HP:0032191"
    ],
    [
      "OMIM:108300",
      "OMIM:184840",
      "OMIM:604841",
      "OMIM:614134",
      "OMIM:614284",
      "ORPHA:166100",
      "ORPHA:250984",
      "ORPHA:828",
      "ORPHA:90653",
      "ORPHA:90654"
    ]
  ],
  [
    [
      "HP:0000083",
      "HP:0000100",
      "HP:0001698",
      "HP:0001701",
      "HP:0001903",
      "HP:0003259",
      "HP:0003493",
      "HP:0003546",
      "HP:0004431",
      "HP:0012378",
      "HP:0012596",
      "HP:0012597"
    ],
    [
```

```
      "OMIM:152700",
      "OMIM:606579",
      "OMIM:609939",
      "OMIM:614420",
      "ORPHA:300345",
      "ORPHA:93552"
    ]
  ],
  [
    [
      "HP:0000142",
      "HP:0000155",
      "HP:0000217",
      "HP:0001097",
      "HP:0001167",
      "HP:0002014",
      "HP:0002027",
      "HP:0002240",
      "HP:0002315",
      "HP:0002814",
      "HP:0002829",
      "HP:0002896",
      "HP:0003124",
      "HP:0003282",
      "HP:0003474",
      "HP:0003546",
      "HP:0004326",
      "HP:0005263",
      "HP:0007042",
      "HP:0010784",
      "HP:0010876",
      "HP:0012514",
      "HP:0012534",
      "HP:0030057",
      "HP:0030140",
      "HP:0030166",
      "HP:0032483",
      "HP:0100633",
      "HP:0100643"
    ],
    [
      "OMIM:270150",
      "ORPHA:289390"
    ]
  ],
  [
    [
      "HP:0000821",
      "HP:0001744",
      "HP:0001888",
      "HP:0001935",
      "HP:0001945",
      "HP:0002018",
      "HP:0002094",
      "HP:0002102",
      "HP:0002202",
```

```
      "HP:0002901",
      "HP:0004396",
      "HP:0010931",
      "HP:0011227",
      "HP:0011703",
      "HP:0012378",
      "HP:0025143",
      "HP:0025435",
      "HP:0030160",
      "HP:0100749"
    ],
    [
      "CCRD:28",
      "OMIM:134610",
      "OMIM:249100",
      "ORPHA:342"
    ]
  ],
  [
    [
      "HP:0000365",
      "HP:0001288",
      "HP:0001397",
      "HP:0001407",
      "HP:0001698",
      "HP:0001744",
      "HP:0002088",
      "HP:0002240",
      "HP:0002385",
      "HP:0003474",
      "HP:0012384",
      "HP:0012514",
      "HP:0030423",
      "HP:0032230",
      "HP:0100785"
    ],
    [
      "OMIM:608710",
      "ORPHA:900"
    ]
  ],
  [
    [
      "HP:0000107",
      "HP:0000121",
      "HP:0001288",
      "HP:0001369",
      "HP:0001744",
      "HP:0001875",
      "HP:0001882",
      "HP:0001913",
      "HP:0001935",
      "HP:0002148",
      "HP:0002240",
      "HP:0002315",
      "HP:0002321",
```

"HP:0002716",
"HP:0002814",
"HP:0002875",
"HP:0003418",
"HP:0003546",
"HP:0003565",
"HP:0004418",
"HP:0005108",
"HP:0005268",
"HP:0006256",
"HP:0006261",
"HP:0007210",
"HP:0010287",
"HP:0012513",
"HP:0012514",
"HP:0012534",
"HP:0025238",
"HP:0030833",
"HP:0030836",
"HP:0030837",
"HP:0030838",
"HP:0030839",
"HP:0030840"
],
[
"OMIM:134750",
"ORPHA:47612"
]
],
[
[
"HP:0000365",
"HP:0000822",
"HP:0002105",
"HP:0002315",
"HP:0002829",
"HP:0012384",
"HP:0030836",
"HP:0030839",
"HP:0030840"
],
[
"OMIM:608710",
"ORPHA:900"
]
],
[
[
"HP:0000083",
"HP:0000366",
"HP:0000377",
"HP:0000572",
"HP:0000938",
"HP:0001041",
"HP:0001167",
"HP:0001225",

```
      "HP:0001780",
      "HP:0001880",
      "HP:0001935",
      "HP:0003546",
      "HP:0009129",
      "HP:0010741",
      "HP:0011911",
      "HP:0025238",
      "HP:0030834",
      "HP:0100534",
      "HP:0100758"
    ],
    [
      "ORPHA:728"
    ]
  ],
  [
    [
      "HP:0000766",
      "HP:0001974",
      "HP:0002094",
      "HP:0002754",
      "HP:0002758",
      "HP:0003565",
      "HP:0011227",
      "HP:0100749",
      "HP:0100774"
    ],
    [
      "ORPHA:793"
    ]
  ],
  [
    [
      "HP:0000223",
      "HP:0000793",
      "HP:0000967",
      "HP:0002315",
      "HP:0012378"
    ],
    [
      "ORPHA:761"
    ]
  ],
  [
    [
      "HP:0000217",
      "HP:0000858",
      "HP:0001097",
      "HP:0001882",
      "HP:0002014",
      "HP:0020181",
      "HP:0030057"
    ],
    [
      "OMIM:152700",
```

"OMIM:270150",
"OMIM:606579",
"OMIM:609939",
"OMIM:614420",
"ORPHA:289390",
"ORPHA:300345",
"ORPHA:93552"
]
],
[
[
"HP:0000246",
"HP:0001880",
"HP:0002094",
"HP:0002113",
"HP:0002583",
"HP:0002910",
"HP:0003212",
"HP:0003236",
"HP:0009098",
"HP:0011227",
"HP:0011897",
"HP:0012213",
"HP:0012387",
"HP:0025066",
"HP:0025392",
"HP:0030057",
"HP:0030877",
"HP:0032300",
"HP:0100512",
"HP:0100582"
],
[
"ORPHA:183"
]
],
[
[
"HP:0002633",
"HP:0003565",
"HP:0011227",
"HP:0012273",
"HP:0012378",
"HP:0025289",
"HP:0030833"
],
[
"OMIM:207600",
"ORPHA:3287"
]
],
[
[
"HP:0000953",
"HP:0001894",
"HP:0001897",

```
        "HP:0001945",
        "HP:0002659",
        "HP:0002910",
        "HP:0003155",
        "HP:0003261",
        "HP:0003326",
        "HP:0003565",
        "HP:0005180",
        "HP:0011120",
        "HP:0011227",
        "HP:0012735",
        "HP:0025392",
        "HP:0025547",
        "HP:0030313",
        "HP:0030840",
        "HP:0030948",
        "HP:0032230",
        "HP:0100827"
      ],
      [
        "OMIM:608710",
        "ORPHA:900"
      ]
    ],
    [
      [
        "HP:0001061",
        "HP:0001649",
        "HP:0001712",
        "HP:0001962",
        "HP:0002829",
        "HP:0003115",
        "HP:0004342",
        "HP:0007514",
        "HP:0012513",
        "HP:0025131",
        "HP:0030837",
        "HP:0030839",
        "HP:0030840",
        "HP:0200034"
      ],
      [
        "CCRD:27",
        "OMIM:301500",
        "ORPHA:324"
      ]
    ],
    [
      [
        "HP:0001167",
        "HP:0001225",
        "HP:0001369",
        "HP:0001386",
        "HP:0001397",
        "HP:0001600",
        "HP:0001707",
```

```
        "HP:0001780",
        "HP:0001785",
        "HP:0001824",
        "HP:0001945",
        "HP:0002027",
        "HP:0002092",
        "HP:0002149",
        "HP:0002592",
        "HP:0002815",
        "HP:0002829",
        "HP:0003149",
        "HP:0003326",
        "HP:0003401",
        "HP:0003418",
        "HP:0003493",
        "HP:0003546",
        "HP:0003565",
        "HP:0004396",
        "HP:0005197",
        "HP:0005202",
        "HP:0010741",
        "HP:0011227",
        "HP:0011956",
        "HP:0012185",
        "HP:0012398",
        "HP:0012611",
        "HP:0025230",
        "HP:0030157",
        "HP:0030166",
        "HP:0030836",
        "HP:0030838",
        "HP:0030839",
        "HP:0030840",
        "HP:0030841",
        "HP:0030948",
        "HP:0100016",
        "HP:0410019"
      ],
      [
        "ORPHA:3452"
      ]
    ],
    [
      [
        "HP:0000010",
        "HP:0000979",
        "HP:0001945",
        "HP:0001974",
        "HP:0002633",
        "HP:0010741",
        "HP:0012514",
        "HP:0031520"
      ],
      [
        "ORPHA:761"
      ]
```

```
    ],
    [
      [
        "HP:0000272",
        "HP:0000347",
        "HP:0000384",
        "HP:0000405",
        "HP:0000413",
        "HP:0000453",
        "HP:0000545",
        "HP:0008551",
        "HP:0008773",
        "HP:0009623",
        "HP:0010880",
        "HP:0011342",
        "HP:0011451",
        "HP:0011471"
      ],
      [
        "OMIM:610536",
        "ORPHA:79113"
      ]
    ],
    [
      [
        "HP:0000253",
        "HP:0000648",
        "HP:0001263",
        "HP:0001285",
        "HP:0001336",
        "HP:0001518",
        "HP:0001857",
        "HP:0003561",
        "HP:0009879",
        "HP:0009882",
        "HP:0011451",
        "HP:0012469",
        "HP:0100026"
      ],
      [
        "OMIM:614261",
        "ORPHA:294016"
      ]
    ],
    [
      [
        "HP:0000407",
        "HP:0000510",
        "HP:0001272",
        "HP:0001284",
        "HP:0001761",
        "HP:0001765",
        "HP:0002474",
        "HP:0003447",
        "HP:0003474",
        "HP:0007141",
```

```
      "HP:0007240",
      "HP:0009088",
      "HP:0011096",
      "HP:0200070"
    ],
    [
      "OMIM:233400",
      "ORPHA:2855"
    ]
  ],
  [
    [
      "HP:0002194",
      "HP:0002282",
      "HP:0007033",
      "HP:0008058"
    ],
    [
      "OMIM:615960",
      "ORPHA:370022"
    ]
  ],
  [
    [
      "HP:0000160",
      "HP:0000518",
      "HP:0000574",
      "HP:0000824",
      "HP:0002194",
      "HP:0002857",
      "HP:0002943",
      "HP:0003510",
      "HP:0005775",
      "HP:0007067",
      "HP:0008619",
      "HP:0011220"
    ],
    [
      "OMIM:616007",
      "ORPHA:436174"
    ]
  ],
  [
    [
      "HP:0000486",
      "HP:0000639",
      "HP:0000750",
      "HP:0001090",
      "HP:0001270",
      "HP:0001274",
      "HP:0001320",
      "HP:0002198",
      "HP:0002350",
      "HP:0002418",
      "HP:0007033",
      "HP:0011003",
```

"HP:0011932",
"HP:0030283"
],
[
"OMIM:615960",
"ORPHA:370022"
]
],
[
[
"HP:0000486",
"HP:0000545",
"HP:0000639",
"HP:0001090",
"HP:0001270",
"HP:0001320",
"HP:0002198",
"HP:0002350",
"HP:0007033",
"HP:0007980"
],
[
"OMIM:615960",
"ORPHA:370022"
]
],
[
[
"HP:0000486",
"HP:0000518",
"HP:0000646",
"HP:0000729",
"HP:0000750",
"HP:0001090",
"HP:0001105",
"HP:0001270",
"HP:0001320",
"HP:0002198",
"HP:0002350",
"HP:0002418",
"HP:0004691",
"HP:0007033",
"HP:0007980",
"HP:0011003",
"HP:0030285"
],
[
"OMIM:615960",
"ORPHA:370022"
]
],
[
[
"HP:0000479",
"HP:0000545",
"HP:0000657",

```
      "HP:0000750",
      "HP:0001270",
      "HP:0001290",
      "HP:0002198",
      "HP:0007033",
      "HP:0007068"
    ],
    [
      "OMIM:615960",
      "ORPHA:370022"
    ]
  ],
  [
    [
      "HP:0000486",
      "HP:0000533",
      "HP:0000639",
      "HP:0000657",
      "HP:0000750",
      "HP:0001090",
      "HP:0001270",
      "HP:0001290",
      "HP:0007033",
      "HP:0007068",
      "HP:0011003"
    ],
    [
      "OMIM:615960",
      "ORPHA:370022"
    ]
  ],
  [
    [
      "HP:0000175",
      "HP:0000201",
      "HP:0001328",
      "HP:0011968",
      "HP:0012418",
      "HP:0030282"
    ],
    [
      "OMIM:117650",
      "ORPHA:1393"
    ]
  ],
  [
    [
      "HP:0000201",
      "HP:0000218",
      "HP:0000494",
      "HP:0000670",
      "HP:0000878",
      "HP:0001328",
      "HP:0011968",
      "HP:0030279",
      "HP:0030282"
```

```
    ],
    [
      "OMIM:117650",
      "ORPHA:1393"
    ]
  ],
  [
    [
      "HP:0000175",
      "HP:0000201",
      "HP:0000316",
      "HP:0000494",
      "HP:0001257",
      "HP:0002011",
      "HP:0002072",
      "HP:0002091",
      "HP:0030282"
    ],
    [
      "OMIM:117650",
      "ORPHA:1393"
    ]
  ],
  [
    [
      "HP:0000175",
      "HP:0000201",
      "HP:0000453",
      "HP:0000465",
      "HP:0000476",
      "HP:0001040",
      "HP:0001631",
      "HP:0002650",
      "HP:0012306"
    ],
    [
      "OMIM:117650",
      "ORPHA:1393"
    ]
  ],
  [
    [
      "HP:0000201",
      "HP:0000218",
      "HP:0000765"
    ],
    [
      "OMIM:117650",
      "ORPHA:1393"
    ]
  ],
  [
    [
      "HP:0000175",
      "HP:0000201",
      "HP:0000405",
```

```
      "HP:0000824",
      "HP:0002650",
      "HP:0030282"
    ],
    [
      "OMIM:117650",
      "ORPHA:1393"
    ]
  ],
  [
    [
      "HP:0000175",
      "HP:0000201",
      "HP:0000883",
      "HP:0002025",
      "HP:0002650",
      "HP:0100543"
    ],
    [
      "OMIM:117650",
      "ORPHA:1393"
    ]
  ],
  [
    [
      "HP:0000175",
      "HP:0000201",
      "HP:0001631",
      "HP:0002093",
      "HP:0004719",
      "HP:0011968",
      "HP:0030282",
      "HP:0030300"
    ],
    [
      "OMIM:117650",
      "ORPHA:1393"
    ]
  ],
  [
    [
      "HP:0000175",
      "HP:0000201",
      "HP:0000204",
      "HP:0002098",
      "HP:0002650",
      "HP:0030282",
      "HP:0030300"
    ],
    [
      "OMIM:117650",
      "ORPHA:1393"
    ]
  ],
  [
    [
```

```
      "HP:0000201",
      "HP:0000405",
      "HP:0000486",
      "HP:0000540",
      "HP:0001631",
      "HP:0002098",
      "HP:0002650",
      "HP:0011968",
      "HP:0030282"
    ],
    [
      "OMIM:117650",
      "ORPHA:1393"
    ]
  ],
  [
    [
      "HP:0000175",
      "HP:0000201",
      "HP:0000405",
      "HP:0000878",
      "HP:0000883",
      "HP:0002650",
      "HP:0030282"
    ],
    [
      "OMIM:117650",
      "ORPHA:1393"
    ]
  ],
  [
    [
      "HP:0000160",
      "HP:0000272",
      "HP:0000286",
      "HP:0000337",
      "HP:0000639",
      "HP:0001250",
      "HP:0003196",
      "HP:0009765",
      "HP:0011344",
      "HP:0011347"
    ],
    [
      "OMIM:245570"
    ]
  ],
  [
    [
      "HP:0000926",
      "HP:0001230",
      "HP:0001999",
      "HP:0002104",
      "HP:0002866",
      "HP:0003025",
      "HP:0003180",
```

```
      "HP:0005257",
      "HP:0006009",
      "HP:0008416",
      "HP:0008905",
      "HP:0010049",
      "HP:0011675",
      "HP:0030306"
    ],
    [
      "OMIM:250220",
      "ORPHA:93317"
    ]
  ],
  [
    [
      "HP:0000926",
      "HP:0000946",
      "HP:0001248",
      "HP:0001290",
      "HP:0001321",
      "HP:0001678",
      "HP:0002079",
      "HP:0002098",
      "HP:0002123",
      "HP:0003180",
      "HP:0003375",
      "HP:0004273",
      "HP:0005108",
      "HP:0009879",
      "HP:0100255"
    ],
    [
      "OMIM:250220",
      "ORPHA:93317"
    ]
  ],
  [
    [
      "HP:0000495",
      "HP:0000522",
      "HP:0001250",
      "HP:0001252",
      "HP:0001263",
      "HP:0001315",
      "HP:0001395",
      "HP:0001773",
      "HP:0002019",
      "HP:0002171",
      "HP:0002353",
      "HP:0002910",
      "HP:0006254",
      "HP:0006579",
      "HP:0009830",
      "HP:0010605",
      "HP:0012520",
      "HP:0200055"
```

],
[
"OMIM:615273",
"ORPHA:404454"
]
],
[
[
"HP:0000252",
"HP:0000495",
"HP:0000522",
"HP:0001252",
"HP:0001263",
"HP:0001315",
"HP:0001511",
"HP:0002019",
"HP:0002151",
"HP:0002353",
"HP:0002650",
"HP:0002910",
"HP:0009830"
],
[
"OMIM:615273",
"ORPHA:404454"
]
],
[
[
"HP:0000486",
"HP:0000495",
"HP:0000522",
"HP:0001250",
"HP:0001252",
"HP:0001263",
"HP:0001315",
"HP:0001395",
"HP:0001511",
"HP:0001773",
"HP:0002019",
"HP:0002151",
"HP:0002171",
"HP:0002353",
"HP:0002650",
"HP:0002910",
"HP:0006254",
"HP:0006579",
"HP:0006958",
"HP:0010605",
"HP:0012448",
"HP:0012520",
"HP:0200055"
],
[
"OMIM:615273",
"ORPHA:404454"

```
      ]
    ],
    [
      [
        "HP:0000218",
        "HP:0000252",
        "HP:0000286",
        "HP:0000347",
        "HP:0000508",
        "HP:0000522",
        "HP:0001250",
        "HP:0001252",
        "HP:0001263",
        "HP:0001315",
        "HP:0001511",
        "HP:0001773",
        "HP:0002019",
        "HP:0002119",
        "HP:0002151",
        "HP:0002353",
        "HP:0002418",
        "HP:0002650",
        "HP:0003196",
        "HP:0006254",
        "HP:0006579",
        "HP:0009085",
        "HP:0009830",
        "HP:0009891",
        "HP:0200055"
      ],
      [
        "OMIM:615273",
        "ORPHA:404454"
      ]
    ],
    [
      [
        "HP:0000252",
        "HP:0000286",
        "HP:0001252",
        "HP:0001263",
        "HP:0001395",
        "HP:0001511",
        "HP:0002353",
        "HP:0003196",
        "HP:0006579",
        "HP:0006956",
        "HP:0100022"
      ],
      [
        "OMIM:615273",
        "ORPHA:404454"
      ]
    ],
    [
      [
```

```
      "HP:0000252",
      "HP:0000286",
      "HP:0000486",
      "HP:0000522",
      "HP:0000657",
      "HP:0001182",
      "HP:0001252",
      "HP:0001263",
      "HP:0001315",
      "HP:0001864",
      "HP:0002007",
      "HP:0002019",
      "HP:0002557",
      "HP:0002910",
      "HP:0005487",
      "HP:0006579",
      "HP:0010605",
      "HP:0010804",
      "HP:0011918"
    ],
    [
      "OMIM:615273",
      "ORPHA:404454"
    ]
  ],
  [
    [
      "HP:0000252",
      "HP:0000431",
      "HP:0000486",
      "HP:0000495",
      "HP:0000520",
      "HP:0000522",
      "HP:0000657",
      "HP:0000678",
      "HP:0001250",
      "HP:0001252",
      "HP:0001263",
      "HP:0001315",
      "HP:0001511",
      "HP:0001773",
      "HP:0002019",
      "HP:0002353",
      "HP:0002553",
      "HP:0002650",
      "HP:0005320",
      "HP:0200055"
    ],
    [
      "OMIM:615273",
      "ORPHA:404454"
    ]
  ],
  [
    [
      "HP:0000252",
```

```
      "HP:0000718",
      "HP:0001250",
      "HP:0010864"
    ],
    [
      "OMIM:251200",
      "OMIM:604317",
      "ORPHA:2512"
    ]
  ],
  [
    [
      "HP:0000639",
      "HP:0000648",
      "HP:0001252",
      "HP:0001254",
      "HP:0001260",
      "HP:0002066",
      "HP:0002370",
      "HP:0007965",
      "HP:0008619"
    ],
    [
      "OMIM:601338",
      "ORPHA:1171"
    ]
  ],
  [
    [
      "HP:0000218",
      "HP:0000278",
      "HP:0000316",
      "HP:0000319",
      "HP:0000347",
      "HP:0000369",
      "HP:0000494",
      "HP:0000768",
      "HP:0001166",
      "HP:0001265",
      "HP:0001363",
      "HP:0001388",
      "HP:0001537",
      "HP:0001655",
      "HP:0001762",
      "HP:0001763",
      "HP:0002151",
      "HP:0002410",
      "HP:0002474",
      "HP:0002868",
      "HP:0003298",
      "HP:0003396",
      "HP:0006801",
      "HP:0006889",
      "HP:0010665",
      "HP:0011220"
    ],
```

```
    [
      "OMIM:182212",
      "ORPHA:2462"
    ]
  ],
  [
    [
      "HP:0000218",
      "HP:0000286",
      "HP:0000316",
      "HP:0000322",
      "HP:0000348",
      "HP:0000463",
      "HP:0000954",
      "HP:0001252",
      "HP:0001263",
      "HP:0001344",
      "HP:0002007",
      "HP:0002079",
      "HP:0002282",
      "HP:0002912",
      "HP:0003196",
      "HP:0006956",
      "HP:0007793",
      "HP:0008070",
      "HP:0010055",
      "HP:0010804",
      "HP:0012120",
      "HP:0012448",
      "HP:0040155",
      "HP:0045034"
    ],
    [
      "OMIM:614105",
      "ORPHA:289307"
    ]
  ],
  [
    [
      "HP:0000325",
      "HP:0000430",
      "HP:0000490",
      "HP:0000558",
      "HP:0000684",
      "HP:0004322",
      "HP:0007495",
      "HP:0009125"
    ],
    [
      "OMIM:269880",
      "ORPHA:3163"
    ]
  ],
  [
    [
      "HP:0000085",
```

```
      "HP:0000179",
      "HP:0000215",
      "HP:0000252",
      "HP:0000348",
      "HP:0000490",
      "HP:0000545",
      "HP:0000582",
      "HP:0000670",
      "HP:0000689",
      "HP:0001643",
      "HP:0002342",
      "HP:0003189",
      "HP:0009765",
      "HP:0009890",
      "HP:0012745"
    ],
    [
      "OMIM:613680",
      "ORPHA:363444"
    ]
  ],
  [
    [
      "HP:0000010",
      "HP:0000230",
      "HP:0000403",
      "HP:0001744",
      "HP:0001875",
      "HP:0001888",
      "HP:0004854",
      "HP:0004866",
      "HP:0005401",
      "HP:0012138"
    ],
    [
      "CCRD:104",
      "OMIM:612541",
      "ORPHA:331176",
      "ORPHA:42738"
    ]
  ],
  [
    [
      "HP:0001539",
      "HP:0005214",
      "HP:0011100"
    ],
    [
      "OMIM:243150",
      "ORPHA:436252"
    ]
  ],
  [
    [
      "HP:0000252",
      "HP:0000486",
```

```
      "HP:0000522",
      "HP:0000657",
      "HP:0001252",
      "HP:0001263",
      "HP:0001395",
      "HP:0001773",
      "HP:0002019",
      "HP:0002059",
      "HP:0002079",
      "HP:0002151",
      "HP:0002353",
      "HP:0002472",
      "HP:0002910",
      "HP:0006579",
      "HP:0006958",
      "HP:0010605",
      "HP:0030303",
      "HP:0100022",
      "HP:0200055"
    ],
    [
      "OMIM:615273",
      "ORPHA:404454"
    ]
  ],
  [
    [
      "HP:0000509",
      "HP:0001025",
      "HP:0001085",
      "HP:0002315",
      "HP:0002516",
      "HP:0002633",
      "HP:0003565",
      "HP:0011227",
      "HP:0011897"
    ],
    [
      "OMIM:191900",
      "ORPHA:575"
    ]
  ],
  [
    [
      "HP:0000256",
      "HP:0001332",
      "HP:0002059",
      "HP:0003150",
      "HP:0100309"
    ],
    [
      "CCRD:34.1",
      "OMIM:231670",
      "ORPHA:25"
    ]
  ],
```

[
  [
    "HP:0000280",
    "HP:0000976",
    "HP:0001880",
    "HP:0002726",
    "HP:0003203",
    "HP:0003212",
    "HP:0006532",
    "HP:0011220",
    "HP:0025419",
    "HP:0031292",
    "HP:0031392",
    "HP:0031393"
  ],
  [
    "OMIM:147060",
    "ORPHA:2314"
  ]
],
[
  [
    "HP:0000272",
    "HP:0000405",
    "HP:0000470",
    "HP:0001014",
    "HP:0001251",
    "HP:0001256",
    "HP:0001433",
    "HP:0001650",
    "HP:0002071",
    "HP:0002779",
    "HP:0003355",
    "HP:0004322",
    "HP:0010729"
  ],
  [
    "OMIM:256540",
    "ORPHA:351"
  ]
],
[
  [
    "HP:0000252",
    "HP:0000269",
    "HP:0000276",
    "HP:0000319",
    "HP:0000341",
    "HP:0000414",
    "HP:0000426",
    "HP:0000490",
    "HP:0000687",
    "HP:0000750",
    "HP:0000767",
    "HP:0001182",
    "HP:0001251",

```
      "HP:0002069",
      "HP:0002373",
      "HP:0010722",
      "HP:0011968",
      "HP:0012171",
      "HP:0100703"
    ],
    [
      "OMIM:614104",
      "ORPHA:464306"
    ]
  ],
  [
    [
      "HP:0000076",
      "HP:0000308",
      "HP:0000733",
      "HP:0001252",
      "HP:0001263",
      "HP:0001266",
      "HP:0002136",
      "HP:0004209",
      "HP:0005709",
      "HP:0011968"
    ],
    [
      "OMIM:616973"
    ]
  ],
  [
    [
      "HP:0000347",
      "HP:0000365",
      "HP:0000400",
      "HP:0000574",
      "HP:0000939",
      "HP:0002354",
      "HP:0002953",
      "HP:0004322",
      "HP:0004349",
      "HP:0009771",
      "HP:0011927",
      "HP:0012368"
    ],
    [
      "OMIM:102500",
      "ORPHA:955"
    ]
  ],
  [
    [
      "HP:0000527",
      "HP:0000533",
      "HP:0000639",
      "HP:0000821",
      "HP:0000824",
```

```
      "HP:0001260",
      "HP:0001265",
      "HP:0001643",
      "HP:0001761",
      "HP:0002066",
      "HP:0002080",
      "HP:0002317",
      "HP:0003477",
      "HP:0004322",
      "HP:0006855",
      "HP:0008070",
      "HP:0011003"
    ],
    [
      "OMIM:275400",
      "ORPHA:3363"
    ]
  ],
  [
    [
      "HP:0000331",
      "HP:0000377",
      "HP:0000498",
      "HP:0000577",
      "HP:0000582",
      "HP:0000767",
      "HP:0001263",
      "HP:0001363",
      "HP:0001385",
      "HP:0001508",
      "HP:0001880",
      "HP:0002155",
      "HP:0002652",
      "HP:0002812",
      "HP:0002938",
      "HP:0002943",
      "HP:0003416",
      "HP:0004430",
      "HP:0004565",
      "HP:0004566",
      "HP:0005352",
      "HP:0006610",
      "HP:0008445",
      "HP:0008947",
      "HP:0009826",
      "HP:0010605",
      "HP:0030043",
      "HP:0030320"
    ],
    [
      "OMIM:617425"
    ]
  ],
  [
    [
      "HP:0001397",
```

```
      "HP:0001976",
      "HP:0003124",
      "HP:0003141",
      "HP:0003155",
      "HP:0010836",
      "HP:0010837",
      "HP:0012347",
      "HP:0012358",
      "HP:0031956",
      "HP:0031964"
    ],
    [
      "OMIM:616829",
      "ORPHA:466703"
    ]
  ],
  [
    [
      "HP:0000218",
      "HP:0001324",
      "HP:0001371",
      "HP:0001385",
      "HP:0001611",
      "HP:0002136",
      "HP:0002650",
      "HP:0003390",
      "HP:0003557",
      "HP:0003803",
      "HP:0010831",
      "HP:0011968"
    ],
    [
      "OMIM:617146"
    ]
  ],
  [
    [
      "HP:0000974",
      "HP:0000977",
      "HP:0001290",
      "HP:0001324",
      "HP:0001382",
      "HP:0002194",
      "HP:0002751",
      "HP:0031158"
    ],
    [
      "OMIM:130010"
    ]
  ],
  [
    [
      "HP:0000175",
      "HP:0000347",
      "HP:0000520",
      "HP:0000592",
```

```
      "HP:0000926",
      "HP:0000973",
      "HP:0001027",
      "HP:0001239",
      "HP:0001252",
      "HP:0001382",
      "HP:0001388",
      "HP:0001883",
      "HP:0002656",
      "HP:0002822",
      "HP:0002827",
      "HP:0002996",
      "HP:0003015",
      "HP:0004233",
      "HP:0004568",
      "HP:0006149",
      "HP:0006187",
      "HP:0006391",
      "HP:0008070",
      "HP:0009811",
      "HP:0011220",
      "HP:0011300",
      "HP:0011341",
      "HP:0012368",
      "HP:0100866"
    ],
    [
      "OMIM:271640",
      "ORPHA:93359"
    ]
  ],
  [
    [
      "HP:0000059",
      "HP:0000248",
      "HP:0000252",
      "HP:0000322",
      "HP:0000341",
      "HP:0000378",
      "HP:0000455",
      "HP:0000463",
      "HP:0000482",
      "HP:0000490",
      "HP:0000527",
      "HP:0000565",
      "HP:0000574",
      "HP:0000664",
      "HP:0000878",
      "HP:0000938",
      "HP:0001263",
      "HP:0001511",
      "HP:0001643",
      "HP:0002000",
      "HP:0002020",
      "HP:0002205",
      "HP:0002553",
```

"HP:0003196",
"HP:0004209",
"HP:0005709",
"HP:0009907",
"HP:0011800"
],
[
"OMIM:615502",
"ORPHA:363611"
]
],
[
[
"HP:0000280",
"HP:0000286",
"HP:0000316",
"HP:0000639",
"HP:0001249",
"HP:0001250",
"HP:0001263",
"HP:0001272",
"HP:0001290",
"HP:0001684",
"HP:0002540",
"HP:0002780",
"HP:0002783",
"HP:0003355",
"HP:0005280",
"HP:0008155",
"HP:0010471",
"HP:0040129"
],
[
"OMIM:616354",
"ORPHA:397709"
]
],
[
[
"HP:0000083",
"HP:0000243",
"HP:0000369",
"HP:0000568",
"HP:0000601",
"HP:0000656",
"HP:0000695",
"HP:0001433",
"HP:0001508",
"HP:0001511",
"HP:0001763",
"HP:0001788",
"HP:0001873",
"HP:0001875",
"HP:0001888",
"HP:0002013",
"HP:0002021",

```
        "HP:0002090",
        "HP:0002148",
        "HP:0002901",
        "HP:0003073",
        "HP:0003075",
        "HP:0007431",
        "HP:0007479",
        "HP:0008064",
        "HP:0008689",
        "HP:0012472"
      ],
      [
        "OMIM:616395"
      ]
    ],
    [
      [
        "HP:0000821",
        "HP:0001249",
        "HP:0001250",
        "HP:0001260",
        "HP:0001289",
        "HP:0001987",
        "HP:0002058",
        "HP:0002066",
        "HP:0002169",
        "HP:0002925",
        "HP:0003201",
        "HP:0003236",
        "HP:0005184",
        "HP:0006801",
        "HP:0007340",
        "HP:0009053"
      ],
      [
        "OMIM:616878",
        "ORPHA:480864"
      ]
    ],
    [
      [
        "HP:0001132",
        "HP:0001519",
        "HP:0002647",
        "HP:0011003"
      ],
      [
        "OMIM:154700",
        "ORPHA:284963"
      ]
    ],
    [
      [
        "HP:0000508",
        "HP:0000520",
        "HP:0002515",
```

```
      "HP:0002653",
      "HP:0002829",
      "HP:0003115",
      "HP:0003155",
      "HP:0003202",
      "HP:0003868",
      "HP:0011220",
      "HP:0012100",
      "HP:0100543",
      "HP:0100774"
    ],
    [
      "OMIM:131300",
      "ORPHA:1328"
    ]
  ],
  [
    [
      "HP:0000340",
      "HP:0000414",
      "HP:0000496",
      "HP:0000582",
      "HP:0000750",
      "HP:0000964",
      "HP:0001249",
      "HP:0001250",
      "HP:0001290",
      "HP:0001344",
      "HP:0001558",
      "HP:0001562",
      "HP:0002099",
      "HP:0002283",
      "HP:0002376",
      "HP:0005946",
      "HP:0006829",
      "HP:0010804",
      "HP:0011344",
      "HP:0012444"
    ],
    [
      "OMIM:616900",
      "ORPHA:488632"
    ]
  ],
  [
    [
      "HP:0000194",
      "HP:0000248",
      "HP:0000340",
      "HP:0000343",
      "HP:0000490",
      "HP:0000565",
      "HP:0000586",
      "HP:0000817",
      "HP:0001270",
      "HP:0001344",
```

```
      "HP:0001347",
      "HP:0002421",
      "HP:0002878",
      "HP:0006829",
      "HP:0010804",
      "HP:0100716"
    ],
    [
      "OMIM:616900",
      "ORPHA:488632"
    ]
  ],
  [
    [
      "HP:0000212",
      "HP:0000737",
      "HP:0001371",
      "HP:0001824",
      "HP:0001941",
      "HP:0002153",
      "HP:0002243",
      "HP:0002902",
      "HP:0003073",
      "HP:0004395",
      "HP:0012531",
      "HP:0030350",
      "HP:0031359"
    ],
    [
      "OMIM:228600"
    ]
  ],
  [
    [
      "HP:0000218",
      "HP:0000704",
      "HP:0000974",
      "HP:0000978",
      "HP:0000993",
      "HP:0001027",
      "HP:0001030",
      "HP:0001058",
      "HP:0001249",
      "HP:0001270",
      "HP:0001319",
      "HP:0001537",
      "HP:0001582",
      "HP:0001763",
      "HP:0002619",
      "HP:0002643",
      "HP:0002650",
      "HP:0003419",
      "HP:0004417",
      "HP:0004419",
      "HP:0004976",
      "HP:0012432",
```

```
      "HP:0031158"
    ],
    [
      "OMIM:618000",
      "ORPHA:536532"
    ]
  ],
  [
    [
      "HP:0001249",
      "HP:0001252",
      "HP:0001263",
      "HP:0001315"
    ],
    [
      "OMIM:616900",
      "ORPHA:488632"
    ]
  ],
  [
    [
      "HP:0000498",
      "HP:0000613",
      "HP:0000653",
      "HP:0002231",
      "HP:0007431",
      "HP:0008070",
      "HP:0045075"
    ],
    [
      "OMIM:602400",
      "ORPHA:91132"
    ]
  ],
  [
    [
      "HP:0000044",
      "HP:0001265",
      "HP:0001272",
      "HP:0002075",
      "HP:0007722",
      "HP:0030341",
      "HP:0030344",
      "HP:0040171"
    ],
    [
      "OMIM:215470",
      "ORPHA:1180"
    ]
  ],
  [
    [
      "HP:0001276",
      "HP:0002011",
      "HP:0002107",
      "HP:0010946",
```

```
        "HP:0100660"
      ],
      [
        "OMIM:149400",
        "ORPHA:3197"
      ]
    ],
    [
      [
        "HP:0000276",
        "HP:0000403",
        "HP:0000426",
        "HP:0000627",
        "HP:0001561",
        "HP:0001629",
        "HP:0002032",
        "HP:0002575",
        "HP:0002780",
        "HP:0003298",
        "HP:0011382",
        "HP:0031936",
        "HP:0100336",
        "HP:0100337"
      ],
      [
        "OMIM:214800",
        "ORPHA:138"
      ]
    ],
    [
      [
        "HP:0000733",
        "HP:0001250",
        "HP:0001252",
        "HP:0002079",
        "HP:0002360",
        "HP:0003763",
        "HP:0005484",
        "HP:0007333",
        "HP:0008872"
      ],
      [
        "OMIM:613454"
      ]
    ],
    [
      [
        "HP:0000518",
        "HP:0001300",
        "HP:0001761",
        "HP:0001765",
        "HP:0002174",
        "HP:0002355",
        "HP:0002460",
        "HP:0003200",
        "HP:0003688",
```

```
        "HP:0003693",
        "HP:0008619",
        "HP:0012049",
        "HP:0100660"
      ],
      [
        "OMIM:606324"
      ]
    ],
    [
      [
        "HP:0000028",
        "HP:0000032",
        "HP:0000049",
        "HP:0000054",
        "HP:0000271",
        "HP:0000316",
        "HP:0000324",
        "HP:0000356",
        "HP:0000358",
        "HP:0000369",
        "HP:0000448",
        "HP:0000478",
        "HP:0000708",
        "HP:0000729",
        "HP:0000965",
        "HP:0001249",
        "HP:0001250",
        "HP:0001252",
        "HP:0007772",
        "HP:0012683",
        "HP:0100764"
      ],
      [
        "OMIM:617516"
      ]
    ],
    [
      [
        "HP:0000166",
        "HP:0000972",
        "HP:0006323",
        "HP:0011132",
        "HP:0025084",
        "HP:0410027"
      ],
      [
        "OMIM:245000",
        "ORPHA:678"
      ]
    ],
    [
      [
        "HP:0000563",
        "HP:0001260",
        "HP:0001310",
```

      "HP:0002061",
      "HP:0002066",
      "HP:0002070",
      "HP:0006986"
    ],
    [
      "OMIM:616907",
      "ORPHA:488594"
    ]
  ],
  [
    [
      "HP:0000020",
      "HP:0000716",
      "HP:0000738",
      "HP:0000741",
      "HP:0001268",
      "HP:0001289",
      "HP:0001332",
      "HP:0001350",
      "HP:0002015",
      "HP:0002059",
      "HP:0002167",
      "HP:0002172",
      "HP:0002527",
      "HP:0004326",
      "HP:0006801",
      "HP:0007256",
      "HP:0031825",
      "HP:0100660"
    ],
    [
      "OMIM:616840"
    ]
  ],
  [
    [
      "HP:0001083",
      "HP:0001166",
      "HP:0001263",
      "HP:0001519"
    ],
    [
      "OMIM:236200",
      "ORPHA:394"
    ]
  ],
  [
    [
      "HP:0000193",
      "HP:0000294",
      "HP:0000421",
      "HP:0000448",
      "HP:0000486",
      "HP:0000490",
      "HP:0000527",

```
      "HP:0000574",
      "HP:0000664",
      "HP:0000958",
      "HP:0000982",
      "HP:0001007",
      "HP:0001257",
      "HP:0001263",
      "HP:0001344",
      "HP:0002015",
      "HP:0002751",
      "HP:0006532",
      "HP:0006989"
    ],
    [
      "OMIM:609528",
      "ORPHA:66631"
    ]
  ],
  [
    [
      "HP:0001257",
      "HP:0002011",
      "HP:0002359",
      "HP:0002754",
      "HP:0003487",
      "HP:0006801",
      "HP:0007021",
      "HP:0200042"
    ],
    [
      "OMIM:613115"
    ]
  ],
  [
    [
      "HP:0001629",
      "HP:0001643",
      "HP:0001669",
      "HP:0001674",
      "HP:0001746",
      "HP:0001750",
      "HP:0002139",
      "HP:0003363",
      "HP:0004935",
      "HP:0004971",
      "HP:0012262",
      "HP:0012890"
    ],
    [
      "OMIM:306955"
    ]
  ],
  [
    [
      "HP:0000958",
      "HP:0000980",
```

```
      "HP:0001155",
      "HP:0001760",
      "HP:0002209",
      "HP:0003777",
      "HP:0006482",
      "HP:0007401",
      "HP:0007502",
      "HP:0007663",
      "HP:0011509",
      "HP:0045075",
      "HP:0200040",
      "HP:0410400"
    ],
    [
      "OMIM:601553",
      "ORPHA:1573"
    ]
  ],
  [
    [
      "HP:0000218",
      "HP:0000252",
      "HP:0000286",
      "HP:0000347",
      "HP:0001249",
      "HP:0001270",
      "HP:0001276",
      "HP:0002360",
      "HP:0006956",
      "HP:0009879"
    ],
    [
      "OMIM:608716"
    ]
  ],
  [
    [
      "HP:0000974",
      "HP:0000978",
      "HP:0001065",
      "HP:0001075",
      "HP:0001373",
      "HP:0001382",
      "HP:0001763",
      "HP:0002829",
      "HP:0025509"
    ],
    [
      "OMIM:130000",
      "ORPHA:287"
    ]
  ],
  [
    [
      "HP:0000649",
      "HP:0000762",
```

```
      "HP:0001761",
      "HP:0002066",
      "HP:0002070",
      "HP:0002073",
      "HP:0002403",
      "HP:0003401",
      "HP:0003474",
      "HP:0007078",
      "HP:0007141",
      "HP:0009053",
      "HP:0031910",
      "HP:0100543"
    ],
    [
      "OMIM:601098",
      "ORPHA:101083"
    ]
  ],
  [
    [
      "HP:0000028",
      "HP:0000041",
      "HP:0000047",
      "HP:0000076",
      "HP:0000098",
      "HP:0000107",
      "HP:0000160",
      "HP:0000164",
      "HP:0000187",
      "HP:0000218",
      "HP:0000221",
      "HP:0000252",
      "HP:0000256",
      "HP:0000286",
      "HP:0000316",
      "HP:0000319",
      "HP:0000325",
      "HP:0000347",
      "HP:0000349",
      "HP:0000369",
      "HP:0000414",
      "HP:0000453",
      "HP:0000463",
      "HP:0000490",
      "HP:0000494",
      "HP:0000525",
      "HP:0000534",
      "HP:0000581",
      "HP:0000582",
      "HP:0000589",
      "HP:0000598",
      "HP:0000601",
      "HP:0000609",
      "HP:0000659",
      "HP:0000708",
      "HP:0000752",
```

```
    "HP:0000957",
    "HP:0000998",
    "HP:0001159",
    "HP:0001250",
    "HP:0001252",
    "HP:0001260",
    "HP:0001263",
    "HP:0001285",
    "HP:0001290",
    "HP:0001385",
    "HP:0001511",
    "HP:0001518",
    "HP:0001622",
    "HP:0001629",
    "HP:0001643",
    "HP:0001655",
    "HP:0001734",
    "HP:0001792",
    "HP:0002007",
    "HP:0002015",
    "HP:0002020",
    "HP:0002021",
    "HP:0002079",
    "HP:0002119",
    "HP:0002247",
    "HP:0002557",
    "HP:0002650",
    "HP:0002938",
    "HP:0003186",
    "HP:0003307",
    "HP:0004209",
    "HP:0004322",
    "HP:0004467",
    "HP:0004532",
    "HP:0006808",
    "HP:0007018",
    "HP:0008587",
    "HP:0010490",
    "HP:0010772",
    "HP:0011328",
    "HP:0011416",
    "HP:0011480",
    "HP:0012110",
    "HP:0012448",
    "HP:0012471",
    "HP:0012803",
    "HP:0025100",
    "HP:0030084",
    "HP:0030301",
    "HP:0045025",
    "HP:0200055",
    "HP:0410030"
  ],
  [
    "OMIM:616975"
  ]
```

],
[
[
"HP:0003281",
"HP:0010836",
"HP:0025435",
"HP:0030890",
"HP:0031956",
"HP:0031964"
],
[
"OMIM:603553"
]
],
[
[
"HP:0000034",
"HP:0000160",
"HP:0000189",
"HP:0000252",
"HP:0000358",
"HP:0000369",
"HP:0000448",
"HP:0000486",
"HP:0000494",
"HP:0000527",
"HP:0000574",
"HP:0000589",
"HP:0000678",
"HP:0000689",
"HP:0000733",
"HP:0000739",
"HP:0000750",
"HP:0001007",
"HP:0001263",
"HP:0002342",
"HP:0002553",
"HP:0002650",
"HP:0009765",
"HP:0010055",
"HP:0011087",
"HP:0011304"
],
[
"OMIM:613684",
"ORPHA:353284"
]
],
[
[
"HP:0001268",
"HP:0001350",
"HP:0002510",
"HP:0100660"
],
[

"OMIM:616840"
]
],
[
[
"HP:0001279",
"HP:0001657",
"HP:0001663",
"HP:0001692",
"HP:0001695",
"HP:0004308",
"HP:0011675",
"HP:0031547"
],
[
"OMIM:614021"
]
],
[
[
"HP:0000750",
"HP:0001256",
"HP:0001263",
"HP:0001348",
"HP:0001762",
"HP:0002079",
"HP:0002191",
"HP:0002317",
"HP:0006913",
"HP:0008936",
"HP:0011448",
"HP:0012795",
"HP:0030890"
],
[
"OMIM:613162",
"ORPHA:320396"
]
],
[
[
"HP:0000010",
"HP:0000467",
"HP:0000969",
"HP:0000988",
"HP:0001263",
"HP:0001270",
"HP:0001284",
"HP:0001324",
"HP:0001558",
"HP:0002829",
"HP:0002907",
"HP:0003115",
"HP:0003458",
"HP:0003687",
"HP:0003724",

"HP:0008944",
"HP:0012587",
"HP:0031910",
"HP:0040083",
"HP:0040129",
"HP:0040180"
],
[
"OMIM:158810",
"ORPHA:610"
]
],
[
[
"HP:0000248",
"HP:0000486",
"HP:0000520",
"HP:0000586",
"HP:0000826",
"HP:0000954",
"HP:0001250",
"HP:0001263",
"HP:0001845",
"HP:0002069",
"HP:0002353",
"HP:0003186",
"HP:0006191",
"HP:0006543",
"HP:0006829",
"HP:0008947"
],
[
"OMIM:616900",
"ORPHA:488632"
]
],
[
[
"HP:0001347",
"HP:0003487",
"HP:0006895",
"HP:0040083"
],
[
"OMIM:616907",
"ORPHA:488594"
]
],
[
[
"HP:0002980",
"HP:0002984",
"HP:0005736",
"HP:0005792"
],
[

```
      "OMIM:610915"
    ]
  ],
  [
    [
      "HP:0000252",
      "HP:0000577",
      "HP:0001249",
      "HP:0001250",
      "HP:0001260",
      "HP:0001943",
      "HP:0001987",
      "HP:0002058",
      "HP:0002104",
      "HP:0002373",
      "HP:0002376",
      "HP:0002913",
      "HP:0003201",
      "HP:0003236",
      "HP:0003487",
      "HP:0006801",
      "HP:0006957",
      "HP:0007359",
      "HP:0011675",
      "HP:0011675"
    ],
    [
      "OMIM:616878",
      "ORPHA:480864"
    ]
  ],
  [
    [
      "HP:0000253",
      "HP:0000768",
      "HP:0000998",
      "HP:0001250",
      "HP:0001371",
      "HP:0001508",
      "HP:0001838",
      "HP:0002808",
      "HP:0003487",
      "HP:0005692",
      "HP:0007377",
      "HP:0007410",
      "HP:0009051",
      "HP:0011922",
      "HP:0012444",
      "HP:0012448",
      "HP:0012713"
    ],
    [
      "OMIM:617527",
      "ORPHA:521426"
    ]
  ],
```

```
[
  [
    "HP:0000365",
    "HP:0001249",
    "HP:0001250",
    "HP:0001263",
    "HP:0011318"
  ],
  [
    "OMIM:602849",
    "ORPHA:53271"
  ]
],
[
  [
    "HP:0000098",
    "HP:0000621",
    "HP:0000973",
    "HP:0001290",
    "HP:0001371",
    "HP:0001385",
    "HP:0001519",
    "HP:0001627",
    "HP:0001639",
    "HP:0001999",
    "HP:0002069",
    "HP:0002119",
    "HP:0002751",
    "HP:0004942"
  ],
  [
    "OMIM:617403"
  ]
],
[
  [
    "HP:0001663",
    "HP:0005184",
    "HP:0012819",
    "HP:0031677"
  ],
  [
    "OMIM:616247"
  ]
],
[
  [
    "HP:0001250",
    "HP:0001371",
    "HP:0002380",
    "HP:0002515",
    "HP:0002650",
    "HP:0002875",
    "HP:0003202",
    "HP:0003323",
    "HP:0003327",
```

```
      "HP:0003391",
      "HP:0003473",
      "HP:0007911",
      "HP:0008944",
      "HP:0010535",
      "HP:0012473",
      "HP:0025331",
      "HP:0030208"
    ],
    [
      "OMIM:616325"
    ]
  ],
  [
    [
      "HP:0000154",
      "HP:0000316",
      "HP:0000369",
      "HP:0000965",
      "HP:0001057",
      "HP:0001250",
      "HP:0001252",
      "HP:0001263",
      "HP:0001558",
      "HP:0002007",
      "HP:0002827",
      "HP:0005180",
      "HP:0005280",
      "HP:0006610",
      "HP:0011304",
      "HP:0200134"
    ],
    [
      "OMIM:608776",
      "ORPHA:79328"
    ]
  ],
  [
    [
      "HP:0000238",
      "HP:0002119",
      "HP:0002507"
    ],
    [
      "OMIM:609637"
    ]
  ],
  [
    [
      "HP:0000486",
      "HP:0001249",
      "HP:0001298",
      "HP:0002650",
      "HP:0003429"
    ],
    [
```

```
      "OMIM:617560",
      "ORPHA:527497"
    ]
  ],
  [
    [
      "HP:0000926",
      "HP:0002515",
      "HP:0003015",
      "HP:0003183",
      "HP:0004568",
      "HP:0008802",
      "HP:0009381",
      "HP:0009826",
      "HP:0200055"
    ],
    [
      "OMIM:177170",
      "ORPHA:750"
    ]
  ],
  [
    [
      "HP:0001268",
      "HP:0001761",
      "HP:0003128",
      "HP:0003259",
      "HP:0003326",
      "HP:0003393",
      "HP:0003394",
      "HP:0003546",
      "HP:0003760",
      "HP:0004305",
      "HP:0008954",
      "HP:0008959",
      "HP:0008981",
      "HP:0009130",
      "HP:0012378",
      "HP:0031177"
    ],
    [
      "OMIM:614321",
      "ORPHA:488650"
    ]
  ],
  [
    [
      "HP:0000278",
      "HP:0000338",
      "HP:0000369",
      "HP:0001260",
      "HP:0001288",
      "HP:0001347",
      "HP:0002063",
      "HP:0002064",
      "HP:0002067",
```

"HP:0004322",
"HP:0025331",
"HP:0100660"
],
[
"OMIM:606693",
"ORPHA:306674"
]
],
[
[
"HP:0001230",
"HP:0001249",
"HP:0001831",
"HP:0003038",
"HP:0004322",
"HP:0005736",
"HP:0006009",
"HP:0009381",
"HP:0010049",
"HP:0010055"
],
[
"OMIM:602875",
"ORPHA:40"
]
],
[
[
"HP:0000518",
"HP:0001251",
"HP:0001263",
"HP:0001272",
"HP:0003417",
"HP:0008820",
"HP:0009901",
"HP:0012368"
],
[
"OMIM:600373",
"ORPHA:1458"
]
],
[
[
"HP:0000093",
"HP:0001903",
"HP:0100502"
],
[
"OMIM:261100",
"ORPHA:35858"
]
],
[
[

```
      "HP:0001433",
      "HP:0002716",
      "HP:0010701",
      "HP:0032455"
    ],
    [
      "OMIM:612840",
      "ORPHA:99844"
    ]
  ],
  [
    [
      "HP:0000501",
      "HP:0000545",
      "HP:0001252",
      "HP:0001382",
      "HP:0002757",
      "HP:0003508",
      "HP:0007973",
      "HP:0030839",
      "HP:0030840"
    ],
    [
      "OMIM:604841",
      "ORPHA:90654"
    ]
  ],
  [
    [
      "HP:0000787",
      "HP:0000869",
      "HP:0002315",
      "HP:0002588",
      "HP:0002893",
      "HP:0003165",
      "HP:0003418",
      "HP:0008200",
      "HP:0011748",
      "HP:0012587",
      "HP:0100522",
      "HP:0100570",
      "HP:0100634",
      "HP:0100829",
      "HP:0500167"
    ],
    [
      "OMIM:131100",
      "ORPHA:652"
    ]
  ],
  [
    [
      "HP:0000083",
      "HP:0000107",
      "HP:0000252",
      "HP:0000961",
```

"HP:0001338",
"HP:0001522",
"HP:0001629",
"HP:0001660",
"HP:0001671",
"HP:0001845",
"HP:0004935",
"HP:0005301",
"HP:0007430",
"HP:0010442",
"HP:0010773",
"HP:0011611",
"HP:0011662",
"HP:0012020",
"HP:0012050",
"HP:0012516",
"HP:0030148",
"HP:0031664",
"HP:0100520"
],
[
"OMIM:617478"
]
],
[
[
"HP:0001260",
"HP:0001272",
"HP:0002015",
"HP:0002066",
"HP:0002385",
"HP:0002650",
"HP:0011448"
],
[
"OMIM:616907",
"ORPHA:488594"
]
],
[
[
"HP:0000514",
"HP:0001260",
"HP:0001276",
"HP:0001285",
"HP:0001776",
"HP:0002070",
"HP:0002317"
],
[
"OMIM:616907",
"ORPHA:488594"
]
],
[
[

```
      "HP:0000316",
      "HP:0000347",
      "HP:0000369",
      "HP:0000677",
      "HP:0000691",
      "HP:0000767",
      "HP:0001234",
      "HP:0001263",
      "HP:0001449",
      "HP:0002308",
      "HP:0002751",
      "HP:0003196",
      "HP:0010109",
      "HP:0011087",
      "HP:0012725",
      "HP:0030084"
    ],
    [
      "OMIM:605282",
      "ORPHA:363417"
    ]
  ],
  [
    [
      "HP:0000098",
      "HP:0000119",
      "HP:0000621",
      "HP:0001290",
      "HP:0001371",
      "HP:0001385",
      "HP:0001519",
      "HP:0001522",
      "HP:0001627",
      "HP:0001639",
      "HP:0001999",
      "HP:0002751",
      "HP:0004942"
    ],
    [
      "OMIM:617402"
    ]
  ],
  [
    [
      "HP:0000252",
      "HP:0000490",
      "HP:0000519",
      "HP:0000958",
      "HP:0000992"
    ],
    [
      "OMIM:133540"
    ]
  ],
  [
    [
```

```
      "HP:0000742",
      "HP:0001252",
      "HP:0001260",
      "HP:0001265",
      "HP:0001927",
      "HP:0002015",
      "HP:0002072",
      "HP:0003763",
      "HP:0006956"
    ],
    [
      "OMIM:200150",
      "ORPHA:2388"
    ]
  ],
  [
    [
      "HP:0000582",
      "HP:0000601",
      "HP:0001263",
      "HP:0001387",
      "HP:0001642",
      "HP:0002938",
      "HP:0004322",
      "HP:0005164",
      "HP:0005280",
      "HP:0009803",
      "HP:0010579",
      "HP:0010884",
      "HP:0011800",
      "HP:0031027"
    ],
    [
      "OMIM:614185"
    ]
  ],
  [
    [
      "HP:0002645",
      "HP:0002753",
      "HP:0002757",
      "HP:0002953",
      "HP:0003100",
      "HP:0003155",
      "HP:0031425",
      "HP:0031846",
      "HP:0040160"
    ],
    [
      "OMIM:615066"
    ]
  ],
  [
    [
      "HP:0000733",
      "HP:0000750",
```

"HP:0000817",
"HP:0001250",
"HP:0001252",
"HP:0002079",
"HP:0002353",
"HP:0002360",
"HP:0003763",
"HP:0007333",
"HP:0011968"
],
[
"OMIM:613454"
]
],
[
[
"HP:0000485",
"HP:0000505",
"HP:0007765",
"HP:0012632"
],
[
"OMIM:309300",
"ORPHA:91489"
]
],
[
[
"HP:0000750",
"HP:0000952",
"HP:0001290",
"HP:0002013",
"HP:0002069",
"HP:0011968",
"HP:0012024"
],
[
"OMIM:230400",
"ORPHA:79239"
]
],
[
[
"HP:0000952",
"HP:0001250",
"HP:0001263",
"HP:0001405",
"HP:0001531",
"HP:0001892",
"HP:0001945",
"HP:0002013",
"HP:0002155",
"HP:0002240",
"HP:0002910",
"HP:0003124",
"HP:0003270",

```
      "HP:0004322"
    ],
    [
      "OMIM:232700",
      "ORPHA:369"
    ]
  ],
  [
    [
      "HP:0000155",
      "HP:0000965",
      "HP:0001269",
      "HP:0001945",
      "HP:0002027",
      "HP:0002140",
      "HP:0002315",
      "HP:0002321",
      "HP:0002829",
      "HP:0003493",
      "HP:0010702",
      "HP:0011227",
      "HP:0012246"
    ],
    [
      "OMIM:615688",
      "ORPHA:404553"
    ]
  ],
  [
    [
      "HP:0000126",
      "HP:0000260",
      "HP:0000280",
      "HP:0000316",
      "HP:0000341",
      "HP:0000369",
      "HP:0000885",
      "HP:0001098",
      "HP:0001141",
      "HP:0001250",
      "HP:0001263",
      "HP:0001510",
      "HP:0001540",
      "HP:0001561",
      "HP:0001643",
      "HP:0001655",
      "HP:0001762",
      "HP:0002007",
      "HP:0003270",
      "HP:0005280",
      "HP:0006487",
      "HP:0006956",
      "HP:0007082",
      "HP:0011129",
      "HP:0011800",
      "HP:0012714"
```

```
      ],
      [
        "OMIM:269150",
        "ORPHA:798"
      ]
    ],
    [
      [
        "HP:0000537",
        "HP:0000635",
        "HP:0002226",
        "HP:0002227",
        "HP:0010862",
        "HP:0012453",
        "HP:0012745",
        "HP:0012785"
      ],
      [
        "OMIM:148820",
        "ORPHA:896"
      ]
    ],
    [
      [
        "HP:0000054",
        "HP:0000212",
        "HP:0000343",
        "HP:0000347",
        "HP:0000463",
        "HP:0000520",
        "HP:0000892",
        "HP:0001647",
        "HP:0002007",
        "HP:0002937",
        "HP:0003042",
        "HP:0003086",
        "HP:0004322",
        "HP:0005280",
        "HP:0007655",
        "HP:0008551",
        "HP:0009381",
        "HP:0009826",
        "HP:0010281"
      ],
      [
        "OMIM:268310",
        "ORPHA:1507"
      ]
    ],
    [
      [
        "HP:0000252",
        "HP:0001250",
        "HP:0001285",
        "HP:0001332",
        "HP:0001344",
```

```
      "HP:0002015",
      "HP:0002119",
      "HP:0002120",
      "HP:0002154",
      "HP:0002194",
      "HP:0002421",
      "HP:0002500",
      "HP:0003348",
      "HP:0004325",
      "HP:0012448",
      "HP:0031518"
    ],
    [
      "OMIM:617668"
    ]
  ],
  [
    [
      "HP:0001822",
      "HP:0001847",
      "HP:0003155",
      "HP:0004363",
      "HP:0010064",
      "HP:0011987",
      "HP:0012531",
      "HP:0025021",
      "HP:0032436",
      "HP:0100529"
    ],
    [
      "OMIM:135100",
      "ORPHA:337"
    ]
  ],
  [
    [
      "HP:0000277",
      "HP:0000508",
      "HP:0000544",
      "HP:0001771",
      "HP:0002486",
      "HP:0003693",
      "HP:0003722",
      "HP:0003749",
      "HP:0003749",
      "HP:0007209",
      "HP:0009053",
      "HP:0040129"
    ],
    [
      "OMIM:160150",
      "ORPHA:169189"
    ]
  ],
  [
    [
```

"HP:0000158",
"HP:0000212",
"HP:0000280",
"HP:0000341",
"HP:0000426",
"HP:0000463",
"HP:0000490",
"HP:0000939",
"HP:0001284",
"HP:0001308",
"HP:0001320",
"HP:0001338",
"HP:0002079",
"HP:0002093",
"HP:0002119",
"HP:0002155",
"HP:0002197",
"HP:0002263",
"HP:0002376",
"HP:0002553",
"HP:0002902",
"HP:0006829",
"HP:0007359",
"HP:0011968",
"HP:0012697",
"HP:0012736",
"HP:0100704"
],
[
"OMIM:616900",
"ORPHA:488632"
]
],
[
[
"HP:0000303",
"HP:0000405",
"HP:0000527",
"HP:0001156",
"HP:0001276",
"HP:0001284",
"HP:0001387",
"HP:0001627",
"HP:0002312",
"HP:0002553",
"HP:0002684",
"HP:0002938",
"HP:0002942",
"HP:0004621",
"HP:0008513",
"HP:0012210",
"HP:0012443",
"HP:0032152"
],
[
"OMIM:139210",

"ORPHA:2588"
]
],
[
[
"HP:0000325",
"HP:0001629",
"HP:0001631",
"HP:0001643",
"HP:0001875",
"HP:0002308",
"HP:0009778",
"HP:0012506",
"HP:0040012"
],
[
"OMIM:609053"
]
],
[
[
"HP:0000049",
"HP:0000218",
"HP:0000316",
"HP:0000347",
"HP:0000463",
"HP:0000508",
"HP:0001249",
"HP:0001388",
"HP:0001840",
"HP:0004322"
],
[
"OMIM:305400",
"ORPHA:915"
]
],
[
[
"HP:0000457",
"HP:0000463",
"HP:0000527",
"HP:0000946",
"HP:0001216",
"HP:0001249",
"HP:0001510",
"HP:0002002",
"HP:0002673",
"HP:0002750",
"HP:0002987",
"HP:0003180",
"HP:0003182",
"HP:0004322",
"HP:0008807",
"HP:0008850",
"HP:0012471"

    ],
    [
      "OMIM:102370",
      "ORPHA:969"
    ]
  ],
  [
    [
      "HP:0001258",
      "HP:0001260",
      "HP:0002064",
      "HP:0002355",
      "HP:0002460",
      "HP:0002600",
      "HP:0007067"
    ],
    [
      "OMIM:616907",
      "ORPHA:488594"
    ]
  ],
  [
    [
      "HP:0000252",
      "HP:0000365",
      "HP:0000518",
      "HP:0000520",
      "HP:0001510"
    ],
    [
      "OMIM:216400"
    ]
  ],
  [
    [
      "HP:0003341",
      "HP:0008066",
      "HP:0031446",
      "HP:0100806",
      "HP:0200041"
    ],
    [
      "OMIM:226700",
      "ORPHA:79404"
    ]
  ],
  [
    [
      "HP:0000750",
      "HP:0001249",
      "HP:0001250",
      "HP:0001270",
      "HP:0001324",
      "HP:0001344",
      "HP:0001649",
      "HP:0001664",

```
      "HP:0001695",
      "HP:0001943",
      "HP:0002151",
      "HP:0002913",
      "HP:0003201",
      "HP:0003236",
      "HP:0003324",
      "HP:0004756",
      "HP:0005184",
      "HP:0006543",
      "HP:0006956",
      "HP:0008942",
      "HP:0011675"
    ],
    [
      "OMIM:616878",
      "ORPHA:480864"
    ]
  ],
  [
    [
      "HP:0001873",
      "HP:0011877",
      "HP:0012143",
      "HP:0030402",
      "HP:0032438"
    ],
    [
      "OMIM:615193"
    ]
  ],
  [
    [
      "HP:0000268",
      "HP:0000483",
      "HP:0000486",
      "HP:0000508",
      "HP:0001065",
      "HP:0001166",
      "HP:0001519",
      "HP:0001653",
      "HP:0002647",
      "HP:0005180",
      "HP:0011003"
    ],
    [
      "OMIM:154700",
      "ORPHA:284963"
    ]
  ],
  [
    [
      "HP:0000543",
      "HP:0000662",
      "HP:0007663",
      "HP:0007737",
```

```
      "HP:0007843"
    ],
    [
      "OMIM:600138"
    ]
  ],
  [
    [
      "HP:0000635",
      "HP:0000639",
      "HP:0001010",
      "HP:0005599"
    ],
    [
      "OMIM:203200",
      "ORPHA:79432"
    ]
  ],
  [
    [
      "HP:0001249",
      "HP:0001263",
      "HP:0001290",
      "HP:0001999",
      "HP:0002750"
    ],
    [
      "OMIM:616900",
      "ORPHA:488632"
    ]
  ],
  [
    [
      "HP:0000639",
      "HP:0001252",
      "HP:0001257",
      "HP:0012736"
    ],
    [
      "OMIM:617560",
      "ORPHA:527497"
    ]
  ],
  [
    [
      "HP:0000639",
      "HP:0002023",
      "HP:0002169",
      "HP:0002266",
      "HP:0002676",
      "HP:0004430",
      "HP:0007281",
      "HP:0025116"
    ],
    [
      "OMIM:617425"
```

```
    ]
  ],
  [
    [
      "HP:0000252",
      "HP:0000470",
      "HP:0000914",
      "HP:0001263",
      "HP:0001276",
      "HP:0001508",
      "HP:0001518",
      "HP:0001999",
      "HP:0002655",
      "HP:0002750",
      "HP:0002788",
      "HP:0002987",
      "HP:0003799",
      "HP:0004209",
      "HP:0004313",
      "HP:0004322",
      "HP:0005407",
      "HP:0005415",
      "HP:0005930",
      "HP:0009237",
      "HP:0009826",
      "HP:0011097",
      "HP:0011344",
      "HP:0011849",
      "HP:0025502",
      "HP:0031141"
    ],
    [
      "OMIM:617425"
    ]
  ],
  [
    [
      "HP:0000218",
      "HP:0000286",
      "HP:0000431",
      "HP:0000490",
      "HP:0000717",
      "HP:0000750",
      "HP:0001249",
      "HP:0001250",
      "HP:0001252",
      "HP:0001500",
      "HP:0001837",
      "HP:0002376",
      "HP:0002650",
      "HP:0007302",
      "HP:0011342"
    ],
    [
      "OMIM:616900",
      "ORPHA:488632"
```

```
    ]
  ],
  [
    [
      "HP:0003557",
      "HP:0003701",
      "HP:0008180",
      "HP:0030319",
      "HP:0031237"
    ],
    [
      "OMIM:609200",
      "ORPHA:98911"
    ]
  ],
  [
    [
      "HP:0000218",
      "HP:0000252",
      "HP:0000278",
      "HP:0000286",
      "HP:0000331",
      "HP:0000341",
      "HP:0000463",
      "HP:0000508",
      "HP:0000574",
      "HP:0000664",
      "HP:0001007",
      "HP:0001508",
      "HP:0002194",
      "HP:0002553",
      "HP:0004209",
      "HP:0004691",
      "HP:0005280",
      "HP:0007665",
      "HP:0008872",
      "HP:0009623",
      "HP:0010044",
      "HP:0010047",
      "HP:0012210"
    ],
    [
      "OMIM:610759"
    ]
  ],
  [
    [
      "HP:0000041",
      "HP:0000047",
      "HP:0000160",
      "HP:0000316",
      "HP:0000346",
      "HP:0000347",
      "HP:0000369",
      "HP:0000431",
      "HP:0001371",
```

```
      "HP:0001762",
      "HP:0005280",
      "HP:0008872",
      "HP:0100490"
    ],
    [
      "OMIM:193700",
      "ORPHA:2053"
    ]
  ],
  [
    [
      "HP:0000175",
      "HP:0000219",
      "HP:0000252",
      "HP:0000294",
      "HP:0000343",
      "HP:0000369",
      "HP:0000664",
      "HP:0000750",
      "HP:0000954",
      "HP:0001007",
      "HP:0005280",
      "HP:0007665",
      "HP:0008897",
      "HP:0010864"
    ],
    [
      "OMIM:122470",
      "ORPHA:199"
    ]
  ],
  [
    [
      "HP:0002666",
      "HP:0003528",
      "HP:0025388"
    ],
    [
      "OMIM:171400",
      "ORPHA:247698"
    ]
  ],
  [
    [
      "HP:0000023",
      "HP:0000243",
      "HP:0000252",
      "HP:0000280",
      "HP:0000520",
      "HP:0000768",
      "HP:0001357",
      "HP:0001371",
      "HP:0001508",
      "HP:0001518",
      "HP:0002194",
```

```
      "HP:0003307",
      "HP:0005280",
      "HP:0006801"
    ],
    [
      "OMIM:252500",
      "ORPHA:576"
    ]
  ],
  [
    [
      "HP:0000248",
      "HP:0000316",
      "HP:0000341",
      "HP:0000520",
      "HP:0000750",
      "HP:0000768",
      "HP:0001156",
      "HP:0001561",
      "HP:0001561",
      "HP:0001822",
      "HP:0002020",
      "HP:0002098",
      "HP:0004209",
      "HP:0005280",
      "HP:0005469",
      "HP:0011951",
      "HP:0012471"
    ],
    [
      "OMIM:617180"
    ]
  ],
  [
    [
      "HP:0000016",
      "HP:0000032",
      "HP:0000054",
      "HP:0000073",
      "HP:0000077",
      "HP:0000308",
      "HP:0000356",
      "HP:0000365",
      "HP:0000369",
      "HP:0000478",
      "HP:0000505",
      "HP:0000729",
      "HP:0000750",
      "HP:0000960",
      "HP:0001249",
      "HP:0001250",
      "HP:0001252",
      "HP:0001270",
      "HP:0001344",
      "HP:0001626",
      "HP:0001643",
```

```
      "HP:0002779",
      "HP:0002817",
      "HP:0003561",
      "HP:0008751",
      "HP:0009601",
      "HP:0010945",
      "HP:0011968",
      "HP:0012795",
      "HP:0100704"
    ],
    [
      "OMIM:617516"
    ]
  ],
  [
    [
      "HP:0000470",
      "HP:0001249",
      "HP:0002751",
      "HP:0003418",
      "HP:0003422",
      "HP:0003521"
    ],
    [
      "OMIM:277300"
    ]
  ],
  [
    [
      "HP:0000316",
      "HP:0001019",
      "HP:0001034",
      "HP:0001508",
      "HP:0002090",
      "HP:0002240",
      "HP:0003073",
      "HP:0004315",
      "HP:0005585",
      "HP:0006515",
      "HP:0006579",
      "HP:0011220",
      "HP:0030948",
      "HP:0032247",
      "HP:0040218",
      "HP:0410240",
      "HP:0410243"
    ],
    [
      "OMIM:222470",
      "ORPHA:84064"
    ]
  ],
  [
    [
      "HP:0001873",
      "HP:0001892",
```

```
      "HP:0001902",
      "HP:0011871",
      "HP:0100309"
    ],
    [
      "OMIM:231200",
      "ORPHA:274"
    ]
  ],
  [
    [
      "HP:0000270",
      "HP:0000689",
      "HP:0000696",
      "HP:0001388",
      "HP:0002645",
      "HP:0002866",
      "HP:0005280",
      "HP:0006660",
      "HP:0008788",
      "HP:0011069"
    ],
    [
      "OMIM:119600",
      "ORPHA:1452"
    ]
  ],
  [
    [
      "HP:0000160",
      "HP:0000252",
      "HP:0000639",
      "HP:0000998",
      "HP:0001019",
      "HP:0001156",
      "HP:0001177",
      "HP:0001250",
      "HP:0001276",
      "HP:0001631",
      "HP:0001671",
      "HP:0001830",
      "HP:0001880",
      "HP:0001999",
      "HP:0002194",
      "HP:0002509",
      "HP:0002521",
      "HP:0002652",
      "HP:0002655",
      "HP:0002987",
      "HP:0004430",
      "HP:0004554",
      "HP:0005387",
      "HP:0005422",
      "HP:0008936",
      "HP:0012736",
      "HP:0040089",
```

```
      "HP:0100878"
    ],
    [
      "OMIM:617425"
    ]
  ],
  [
    [
      "HP:0001433",
      "HP:0001903",
      "HP:0001945",
      "HP:0002113",
      "HP:0002716",
      "HP:0002788",
      "HP:0002851",
      "HP:0003237",
      "HP:0003261",
      "HP:0003270",
      "HP:0003496",
      "HP:0011897",
      "HP:0012311",
      "HP:0100827"
    ],
    [
      "OMIM:601859",
      "ORPHA:3261"
    ]
  ],
  [
    [
      "HP:0000320",
      "HP:0000518",
      "HP:0000819",
      "HP:0000821",
      "HP:0000822",
      "HP:0000939",
      "HP:0000962",
      "HP:0001620",
      "HP:0001763",
      "HP:0001956",
      "HP:0002155",
      "HP:0002216",
      "HP:0003241",
      "HP:0003758",
      "HP:0004322",
      "HP:0005978",
      "HP:0012804",
      "HP:0025441"
    ],
    [
      "OMIM:277700",
      "ORPHA:902"
    ]
  ],
  [
    [
```

```
      "HP:0000602",
      "HP:0001249",
      "HP:0001252",
      "HP:0001283",
      "HP:0001284",
      "HP:0002093",
      "HP:0002307",
      "HP:0002650",
      "HP:0003701",
      "HP:0006466"
    ],
    [
      "OMIM:161800"
    ]
  ],
  [
    [
      "HP:0001252",
      "HP:0001263",
      "HP:0001290",
      "HP:0003124",
      "HP:0003141",
      "HP:0003155",
      "HP:0008947",
      "HP:0010837",
      "HP:0012347",
      "HP:0012358",
      "HP:0031956",
      "HP:0031964"
    ],
    [
      "OMIM:616829",
      "ORPHA:466703"
    ]
  ],
  [
    [
      "HP:0000505",
      "HP:0000510",
      "HP:0000648",
      "HP:0000750",
      "HP:0001249",
      "HP:0001956",
      "HP:0007814",
      "HP:0007843",
      "HP:0010442",
      "HP:0030329"
    ],
    [
      "OMIM:615982"
    ]
  ],
  [
    [
      "HP:0000252",
      "HP:0000961",
```

```
      "HP:0000969",
      "HP:0001319",
      "HP:0001522",
      "HP:0001660",
      "HP:0001999",
      "HP:0003259",
      "HP:0004935",
      "HP:0005301",
      "HP:0007430",
      "HP:0010773",
      "HP:0011611",
      "HP:0011662",
      "HP:0012020",
      "HP:0012516",
      "HP:0030148",
      "HP:0031664",
      "HP:0031834"
    ],
    [
      "OMIM:617478"
    ]
  ],
  [
    [
      "HP:0001635",
      "HP:0001644",
      "HP:0001678",
      "HP:0006699"
    ],
    [
      "OMIM:604145"
    ]
  ],
  [
    [
      "HP:0000303",
      "HP:0000325",
      "HP:0000337",
      "HP:0000411",
      "HP:0000490",
      "HP:0000627",
      "HP:0000952",
      "HP:0000958",
      "HP:0001114",
      "HP:0001263",
      "HP:0001396",
      "HP:0002089",
      "HP:0002240",
      "HP:0003316",
      "HP:0011985",
      "HP:0012704",
      "HP:0030148",
      "HP:0045075"
    ],
    [
      "OMIM:118450",
```

"ORPHA:52"
]
],
[
[
"HP:0000496",
"HP:0001250",
"HP:0001263",
"HP:0007360",
"HP:0007663"
],
[
"OMIM:617622"
]
],
[
[
"HP:0000028",
"HP:0000047",
"HP:0000076",
"HP:0000098",
"HP:0000107",
"HP:0000160",
"HP:0000164",
"HP:0000187",
"HP:0000218",
"HP:0000221",
"HP:0000252",
"HP:0000256",
"HP:0000286",
"HP:0000316",
"HP:0000319",
"HP:0000325",
"HP:0000347",
"HP:0000349",
"HP:0000365",
"HP:0000407",
"HP:0000414",
"HP:0000453",
"HP:0000463",
"HP:0000490",
"HP:0000494",
"HP:0000525",
"HP:0000534",
"HP:0000568",
"HP:0000581",
"HP:0000582",
"HP:0000589",
"HP:0000598",
"HP:0000601",
"HP:0000648",
"HP:0000659",
"HP:0000708",
"HP:0000752",
"HP:0000954",
"HP:0000957",

```
      "HP:0000998",
      "HP:0001159",
      "HP:0001250",
      "HP:0001252",
      "HP:0001256",
      "HP:0001263",
      "HP:0001273",
      "HP:0001285",
      "HP:0001290",
      "HP:0001317",
      "HP:0001385",
      "HP:0001510",
      "HP:0001511",
      "HP:0001629",
      "HP:0001643",
      "HP:0001655",
      "HP:0001671",
      "HP:0001734",
      "HP:0001792",
      "HP:0002007",
      "HP:0002020",
      "HP:0002021",
      "HP:0002119",
      "HP:0002247",
      "HP:0002273",
      "HP:0002334",
      "HP:0002557",
      "HP:0002650",
      "HP:0002938",
      "HP:0003186",
      "HP:0003307",
      "HP:0003429",
      "HP:0004209",
      "HP:0004322",
      "HP:0004467",
      "HP:0004532",
      "HP:0006808",
      "HP:0007018",
      "HP:0007361",
      "HP:0010772",
      "HP:0011328",
      "HP:0011968",
      "HP:0012448",
      "HP:0012471",
      "HP:0025100",
      "HP:0030301",
      "HP:0045025",
      "HP:0200055",
      "HP:0410030"
    ],
    [
      "OMIM:616975"
    ]
  ],
  [
    [
```

"HP:0000218",
"HP:0000232",
"HP:0000325",
"HP:0000327",
"HP:0000460",
"HP:0000581",
"HP:0000883",
"HP:0000895",
"HP:0001166",
"HP:0001249",
"HP:0001763",
"HP:0002761",
"HP:0002857",
"HP:0002987",
"HP:0003083",
"HP:0005280",
"HP:0012385"
],
[
"OMIM:600920",
"ORPHA:2460"
]
],
[
[
"HP:0000252",
"HP:0000316",
"HP:0000320",
"HP:0000403",
"HP:0000582",
"HP:0001010",
"HP:0001511",
"HP:0002110",
"HP:0002265",
"HP:0002720",
"HP:0002850",
"HP:0003347",
"HP:0003565",
"HP:0004315",
"HP:0006532",
"HP:0011227",
"HP:0030084",
"HP:0032170"
],
[
"OMIM:251260",
"ORPHA:647"
]
],
[
[
"HP:0000097",
"HP:0000252",
"HP:0000316",
"HP:0000369",
"HP:0000776",

```
      "HP:0002036",
      "HP:0002059",
      "HP:0002580",
      "HP:0008677",
      "HP:0009879"
    ],
    [
      "OMIM:617730"
    ]
  ],
  [
    [
      "HP:0000175",
      "HP:0000453",
      "HP:0002925",
      "HP:0008191",
      "HP:0011968",
      "HP:0031507",
      "HP:0100786"
    ],
    [
      "OMIM:241850",
      "ORPHA:1226"
    ]
  ],
  [
    [
      "HP:0000274",
      "HP:0000336",
      "HP:0000486",
      "HP:0000586",
      "HP:0000706",
      "HP:0001006",
      "HP:0001596",
      "HP:0002007",
      "HP:0003510",
      "HP:0011120",
      "HP:0012471"
    ],
    [
      "OMIM:230740",
      "ORPHA:2067"
    ]
  ],
  [
    [
      "HP:0000639",
      "HP:0001876",
      "HP:0002503",
      "HP:0002720",
      "HP:0002850",
      "HP:0004315",
      "HP:0005374",
      "HP:0005528",
      "HP:0007340",
      "HP:0008969"
```

```
    ],
    [
      "OMIM:159550",
      "ORPHA:2585"
    ]
  ],
  [
    [
      "HP:0000164",
      "HP:0000561",
      "HP:0000971",
      "HP:0002223",
      "HP:0002298",
      "HP:0008404",
      "HP:0040039"
    ],
    [
      "OMIM:614931"
    ]
  ],
  [
    [
      "HP:0000325",
      "HP:0000358",
      "HP:0000407",
      "HP:0000750",
      "HP:0001263",
      "HP:0001338",
      "HP:0001533",
      "HP:0002099",
      "HP:0004691",
      "HP:0010485",
      "HP:0045075",
      "HP:0045086",
      "HP:0100702"
    ],
    [
      "OMIM:604213",
      "ORPHA:314597"
    ]
  ],
  [
    [
      "HP:0000193",
      "HP:0000272",
      "HP:0000347",
      "HP:0000768",
      "HP:0001166",
      "HP:0001631",
      "HP:0001655",
      "HP:0001659",
      "HP:0001704",
      "HP:0002616",
      "HP:0002650",
      "HP:0004927",
      "HP:0005180",
```

      "HP:0011645",
      "HP:0012385"
    ],
    [
      "OMIM:610168"
    ]
  ],
  [
    [
      "HP:0000083",
      "HP:0000107",
      "HP:0001997",
      "HP:0002149",
      "HP:0003259",
      "HP:0003774"
    ],
    [
      "OMIM:162000",
      "ORPHA:88950"
    ]
  ],
  [
    [
      "HP:0000239",
      "HP:0000242",
      "HP:0000680",
      "HP:0000774",
      "HP:0000894",
      "HP:0002007",
      "HP:0002645",
      "HP:0002650",
      "HP:0002866",
      "HP:0003396",
      "HP:0004322",
      "HP:0005259",
      "HP:0005280",
      "HP:0006297",
      "HP:0008788",
      "HP:0008804",
      "HP:0011223",
      "HP:0011800",
      "HP:0100864"
    ],
    [
      "OMIM:119600",
      "ORPHA:1452"
    ]
  ],
  [
    [
      "HP:0000325",
      "HP:0000378",
      "HP:0000411",
      "HP:0000545",
      "HP:0000577",
      "HP:0000729",

```
      "HP:0001249",
      "HP:0001263",
      "HP:0004209",
      "HP:0005824",
      "HP:0008619",
      "HP:0009904",
      "HP:0100021"
    ],
    [
      "OMIM:616975"
    ]
  ],
  [
    [
      "HP:0001288",
      "HP:0003044",
      "HP:0003273",
      "HP:0003798",
      "HP:0008994"
    ],
    [
      "OMIM:609285"
    ]
  ],
  [
    [
      "HP:0000605",
      "HP:0000639",
      "HP:0001251",
      "HP:0001260",
      "HP:0001272",
      "HP:0002061",
      "HP:0002353",
      "HP:0002355",
      "HP:0002395",
      "HP:0003487",
      "HP:0007377",
      "HP:0012896",
      "HP:0100543"
    ],
    [
      "OMIM:607259",
      "ORPHA:99013"
    ]
  ],
  [
    [
      "HP:0001257",
      "HP:0001260",
      "HP:0001272",
      "HP:0002015",
      "HP:0002066"
    ],
    [
      "OMIM:616907",
      "ORPHA:488594"
```

```
    ]
  ],
  [
    [
      "HP:0000028",
      "HP:0000054",
      "HP:0000308",
      "HP:0000376",
      "HP:0000480",
      "HP:0001290",
      "HP:0001635",
      "HP:0002015",
      "HP:0002020",
      "HP:0002033",
      "HP:0002643",
      "HP:0002878",
      "HP:0010628",
      "HP:0011381"
    ],
    [
      "OMIM:214800",
      "ORPHA:138"
    ]
  ],
  [
    [
      "HP:0001347",
      "HP:0002061",
      "HP:0002497",
      "HP:0002506",
      "HP:0011448"
    ],
    [
      "OMIM:616907",
      "ORPHA:488594"
    ]
  ],
  [
    [
      "HP:0001250",
      "HP:0001324",
      "HP:0001433",
      "HP:0002376",
      "HP:0003236",
      "HP:0012759",
      "HP:0025435",
      "HP:0031956"
    ],
    [
      "OMIM:610217"
    ]
  ],
  [
    [
      "HP:0000926",
      "HP:0001256",
```

```
      "HP:0001433",
      "HP:0002071",
      "HP:0008166",
      "HP:0010729"
    ],
    [
      "OMIM:230650",
      "ORPHA:79257"
    ]
  ],
  [
    [
      "HP:0001595",
      "HP:0001597",
      "HP:0006482",
      "HP:0007530"
    ],
    [
      "OMIM:148600"
    ]
  ],
  [
    [
      "HP:0000767",
      "HP:0000957",
      "HP:0001010",
      "HP:0001067",
      "HP:0001480",
      "HP:0002751",
      "HP:0007565",
      "HP:0009732"
    ],
    [
      "OMIM:162200",
      "ORPHA:363700"
    ]
  ],
  [
    [
      "HP:0001397",
      "HP:0001405",
      "HP:0003124",
      "HP:0003155",
      "HP:0003236",
      "HP:0010837",
      "HP:0011967"
    ],
    [
      "OMIM:616829",
      "ORPHA:466703"
    ]
  ],
  [
    [
      "HP:0000510",
      "HP:0000580",
```

```
      "HP:0000662",
      "HP:0001123",
      "HP:0007663",
      "HP:0007843",
      "HP:0012512",
      "HP:0025159"
    ],
    [
      "OMIM:617433"
    ]
  ],
  [
    [
      "HP:0000543",
      "HP:0001257",
      "HP:0002015",
      "HP:0002194",
      "HP:0002300",
      "HP:0002421",
      "HP:0007663",
      "HP:0008947",
      "HP:0011471",
      "HP:0012043"
    ],
    [
      "OMIM:617560",
      "ORPHA:527497"
    ]
  ],
  [
    [
      "HP:0000119",
      "HP:0000238",
      "HP:0001256",
      "HP:0001321",
      "HP:0001876",
      "HP:0002099",
      "HP:0002317",
      "HP:0002500",
      "HP:0002673",
      "HP:0004315",
      "HP:0031413",
      "HP:0100702"
    ],
    [
      "OMIM:159550",
      "ORPHA:2585"
    ]
  ],
  [
    [
      "HP:0001250",
      "HP:0001308",
      "HP:0001336",
      "HP:0002069",
      "HP:0002322",
```

```
      "HP:0002515",
      "HP:0003391",
      "HP:0003690",
      "HP:0003701",
      "HP:0010819",
      "HP:0012473",
      "HP:0040081",
      "HP:0045040"
    ],
    [
      "OMIM:159950",
      "ORPHA:2590"
    ]
  ],
  [
    [
      "HP:0000280",
      "HP:0000486",
      "HP:0000767",
      "HP:0000886",
      "HP:0001252",
      "HP:0001270",
      "HP:0001407",
      "HP:0001539",
      "HP:0001582",
      "HP:0001653",
      "HP:0001999",
      "HP:0002176",
      "HP:0002651",
      "HP:0002673",
      "HP:0002751",
      "HP:0003048",
      "HP:0003090",
      "HP:0003265",
      "HP:0003319",
      "HP:0004322",
      "HP:0004565",
      "HP:0008462",
      "HP:0040163"
    ],
    [
      "OMIM:617425"
    ]
  ],
  [
    [
      "HP:0004808",
      "HP:0012484",
      "HP:0030402"
    ],
    [
      "OMIM:601399"
    ]
  ],
  [
    [
```

```
      "HP:0000347",
      "HP:0000508",
      "HP:0000822",
      "HP:0001284",
      "HP:0002938",
      "HP:0002987",
      "HP:0003077",
      "HP:0007485",
      "HP:0007495",
      "HP:0008112",
      "HP:0008994",
      "HP:0009046"
    ],
    [
      "OMIM:616516"
    ]
  ],
  [
    [
      "HP:0000729",
      "HP:0000750",
      "HP:0001249",
      "HP:0001344",
      "HP:0010535",
      "HP:0010677"
    ],
    [
      "OMIM:616975"
    ]
  ],
  [
    [
      "HP:0001249",
      "HP:0001250",
      "HP:0001251",
      "HP:0001254",
      "HP:0001260",
      "HP:0001288",
      "HP:0001324",
      "HP:0001649",
      "HP:0001941",
      "HP:0001942",
      "HP:0001943",
      "HP:0001987",
      "HP:0002013",
      "HP:0002058",
      "HP:0002094",
      "HP:0002151",
      "HP:0002307",
      "HP:0002910",
      "HP:0002913",
      "HP:0002919",
      "HP:0003128",
      "HP:0003198",
      "HP:0003201",
      "HP:0003236",
```

```
      "HP:0003690",
      "HP:0004756",
      "HP:0005184",
      "HP:0006682",
      "HP:0006956",
      "HP:0007340",
      "HP:0012544",
      "HP:0045045",
      "HP:0100960"
    ],
    [
      "OMIM:616878",
      "ORPHA:480864"
    ]
  ],
  [
    [
      "HP:0000028",
      "HP:0000126",
      "HP:0000218",
      "HP:0000219",
      "HP:0000316",
      "HP:0000369",
      "HP:0000494",
      "HP:0000664",
      "HP:0000974",
      "HP:0001181",
      "HP:0001182",
      "HP:0001290",
      "HP:0001762",
      "HP:0002804",
      "HP:0006094",
      "HP:0010489"
    ],
    [
      "OMIM:601776",
      "ORPHA:2953"
    ]
  ],
  [
    [
      "HP:0001258",
      "HP:0002064",
      "HP:0002355"
    ],
    [
      "OMIM:616907",
      "ORPHA:488594"
    ]
  ],
  [
    [
      "HP:0001251",
      "HP:0001257",
      "HP:0002064",
      "HP:0002169",
```

```
      "HP:0002312",
      "HP:0003401",
      "HP:0003487",
      "HP:0006886",
      "HP:0009830",
      "HP:0012534",
      "HP:0031910",
      "HP:0100543"
    ],
    [
      "OMIM:604187",
      "ORPHA:100991"
    ]
  ],
  [
    [
      "HP:0000093",
      "HP:0000969",
      "HP:0001518",
      "HP:0002090",
      "HP:0002202",
      "HP:0003073",
      "HP:0003124",
      "HP:0008360",
      "HP:0008677"
    ],
    [
      "OMIM:256300",
      "ORPHA:839"
    ]
  ],
  [
    [
      "HP:0000369",
      "HP:0000510",
      "HP:0000653",
      "HP:0000750",
      "HP:0000957",
      "HP:0001156",
      "HP:0004322",
      "HP:0008070",
      "HP:0009890",
      "HP:0012471",
      "HP:0031936",
      "HP:0045075"
    ],
    [
      "OMIM:250410",
      "ORPHA:166035"
    ]
  ],
  [
    [
      "HP:0000158",
      "HP:0000262",
      "HP:0000280",
```

"HP:0000341",
"HP:0000426",
"HP:0000463",
"HP:0000490",
"HP:0000565",
"HP:0000648",
"HP:0000878",
"HP:0000998",
"HP:0001250",
"HP:0001284",
"HP:0001320",
"HP:0001338",
"HP:0002079",
"HP:0002119",
"HP:0002263",
"HP:0002353",
"HP:0002376",
"HP:0002553",
"HP:0006829",
"HP:0011968",
"HP:0012020",
"HP:0012697",
"HP:0012736"
],
[
"OMIM:616900",
"ORPHA:488632"
]
],
[
[
"HP:0000256",
"HP:0000316",
"HP:0000337",
"HP:0000369",
"HP:0000470",
"HP:0000475",
"HP:0000494",
"HP:0000957",
"HP:0001480",
"HP:0001488",
"HP:0001510",
"HP:0001638",
"HP:0002162",
"HP:0007018",
"HP:0011968"
],
[
"OMIM:613224"
]
],
[
[
"HP:0000545",
"HP:0000577",
"HP:0001083",

```
        "HP:0001166",
        "HP:0001249",
        "HP:0001251",
        "HP:0001260",
        "HP:0001288",
        "HP:0007256",
        "HP:0012444",
        "HP:0030854",
        "HP:0031284"
      ],
      [
        "OMIM:236200",
        "ORPHA:394"
      ]
    ],
    [
      [
        "HP:0000212",
        "HP:0000252",
        "HP:0000253",
        "HP:0000347",
        "HP:0000463",
        "HP:0000520",
        "HP:0000670",
        "HP:0000924",
        "HP:0000960",
        "HP:0001028",
        "HP:0001156",
        "HP:0001252",
        "HP:0001265",
        "HP:0001270",
        "HP:0001290",
        "HP:0001324",
        "HP:0001407",
        "HP:0001508",
        "HP:0001548",
        "HP:0001561",
        "HP:0002007",
        "HP:0002063",
        "HP:0002090",
        "HP:0002176",
        "HP:0002240",
        "HP:0002808",
        "HP:0002850",
        "HP:0002867",
        "HP:0002904",
        "HP:0002938",
        "HP:0003016",
        "HP:0003196",
        "HP:0003265",
        "HP:0003307",
        "HP:0003416",
        "HP:0003690",
        "HP:0004060",
        "HP:0004315",
        "HP:0004565",
```

```
      "HP:0005193",
      "HP:0005280",
      "HP:0005306",
      "HP:0005619",
      "HP:0006532",
      "HP:0007340",
      "HP:0008936",
      "HP:0009826",
      "HP:0100543",
      "HP:0100625",
      "HP:0100865"
    ],
    [
      "OMIM:617425"
    ]
  ],
  [
    [
      "HP:0001761",
      "HP:0002527",
      "HP:0002921",
      "HP:0003448",
      "HP:0003487",
      "HP:0006970",
      "HP:0007340",
      "HP:0009072",
      "HP:0011808",
      "HP:0030180",
      "HP:0030181"
    ],
    [
      "OMIM:609260",
      "ORPHA:99947"
    ]
  ],
  [
    [
      "HP:0000272",
      "HP:0000308",
      "HP:0000365",
      "HP:0000494"
    ],
    [
      "OMIM:613717"
    ]
  ],
  [
    [
      "HP:0000161",
      "HP:0000252",
      "HP:0000437",
      "HP:0000873",
      "HP:0005273",
      "HP:0006870",
      "HP:0009099"
    ],
```

```
    [
      "OMIM:142946"
    ]
  ],
  [
    [
      "HP:0000733",
      "HP:0000750",
      "HP:0001250",
      "HP:0001252",
      "HP:0002188",
      "HP:0002353",
      "HP:0002360",
      "HP:0005484",
      "HP:0008872"
    ],
    [
      "OMIM:613454"
    ]
  ],
  [
    [
      "HP:0000252",
      "HP:0000508",
      "HP:0000545",
      "HP:0000717",
      "HP:0000750",
      "HP:0000752",
      "HP:0001249",
      "HP:0004322",
      "HP:0005617",
      "HP:0010631",
      "HP:0031936"
    ],
    [
      "OMIM:617333"
    ]
  ],
  [
    [
      "HP:0000023",
      "HP:0000076",
      "HP:0000579",
      "HP:0000670",
      "HP:0000822",
      "HP:0000973",
      "HP:0001653",
      "HP:0002110",
      "HP:0002616",
      "HP:0005180",
      "HP:0009926",
      "HP:0100857"
    ],
    [
      "OMIM:219100"
    ]
```

],
[
[
"HP:0000407",
"HP:0000741",
"HP:0002515",
"HP:0002653",
"HP:0003691",
"HP:0003701",
"HP:0003805",
"HP:0007126",
"HP:0008180",
"HP:0010639",
"HP:0100543"
],
[
"OMIM:167320",
"ORPHA:52430"
]
],
[
[
"HP:0000394",
"HP:0000506",
"HP:0000556",
"HP:0000813",
"HP:0001153",
"HP:0001642",
"HP:0001647",
"HP:0001770",
"HP:0002023",
"HP:0004209",
"HP:0004322",
"HP:0004442",
"HP:0031624",
"HP:0410049"
],
[
"OMIM:300707",
"ORPHA:140952"
]
],
[
[
"HP:0000621",
"HP:0000973",
"HP:0001290",
"HP:0001371",
"HP:0001385",
"HP:0001519",
"HP:0001627",
"HP:0001671",
"HP:0001999",
"HP:0002751",
"HP:0007552"
],

    [
      "OMIM:617403"
    ]
  ],
  [
    [
      "HP:0000158",
      "HP:0000252",
      "HP:0000280",
      "HP:0000527",
      "HP:0000664",
      "HP:0000824",
      "HP:0001007",
      "HP:0001250",
      "HP:0001265",
      "HP:0001270",
      "HP:0001344",
      "HP:0002120",
      "HP:0002376",
      "HP:0002650",
      "HP:0006829",
      "HP:0007957",
      "HP:0011344",
      "HP:0011734"
    ],
    [
      "OMIM:616900",
      "ORPHA:488632"
    ]
  ],
  [
    [
      "HP:0000319",
      "HP:0000343",
      "HP:0000347",
      "HP:0000365",
      "HP:0000518",
      "HP:0000527",
      "HP:0000670",
      "HP:0001252",
      "HP:0001270",
      "HP:0001488",
      "HP:0002007",
      "HP:0002205",
      "HP:0008872",
      "HP:0008897",
      "HP:0012646"
    ],
    [
      "OMIM:242840",
      "ORPHA:1493"
    ]
  ],
  [
    [
      "HP:0001265",

```
      "HP:0001324",
      "HP:0002486",
      "HP:0003236",
      "HP:0003552",
      "HP:0003688",
      "HP:0003720"
    ],
    [
      "OMIM:160800"
    ]
  ],
  [
    [
      "HP:0000952",
      "HP:0001744",
      "HP:0001903",
      "HP:0001923",
      "HP:0002240",
      "HP:0004445",
      "HP:0004844",
      "HP:0005560",
      "HP:0032106",
      "HP:0410177"
    ],
    [
      "OMIM:130600"
    ]
  ],
  [
    [
      "HP:0001397",
      "HP:0001976",
      "HP:0002240",
      "HP:0003155",
      "HP:0010837",
      "HP:0011967"
    ],
    [
      "OMIM:616829",
      "ORPHA:466703"
    ]
  ],
  [
    [
      "HP:0001260",
      "HP:0001283",
      "HP:0002015",
      "HP:0002273",
      "HP:0003487",
      "HP:0011448",
      "HP:0012473"
    ],
    [
      "OMIM:105400"
    ]
  ],
```

```
[
  [
    "HP:0000407",
    "HP:0001319",
    "HP:0001324",
    "HP:0001363",
    "HP:0001531",
    "HP:0002020",
    "HP:0002098",
    "HP:0002151",
    "HP:0002490",
    "HP:0002910",
    "HP:0003198",
    "HP:0003200",
    "HP:0003324",
    "HP:0003348",
    "HP:0003688",
    "HP:0008872",
    "HP:0011950",
    "HP:0045045"
  ],
  [
    "OMIM:616974",
    "ORPHA:478042"
  ]
],
[
  [
    "HP:0000952",
    "HP:0000989",
    "HP:0001396",
    "HP:0001433",
    "HP:0001510",
    "HP:0001627",
    "HP:0001928",
    "HP:0002240",
    "HP:0002650",
    "HP:0002908",
    "HP:0003112",
    "HP:0003573",
    "HP:0010701",
    "HP:0012202",
    "HP:0031956",
    "HP:0031964",
    "HP:0100810"
  ],
  [
    "OMIM:615878",
    "ORPHA:480483"
  ]
],
[
  [
    "HP:0000952",
    "HP:0001744",
    "HP:0001878",
```

```
      "HP:0001923",
      "HP:0005502"
    ],
    [
      "OMIM:612690"
    ]
  ],
  [
    [
      "HP:0000047",
      "HP:0000369",
      "HP:0000508",
      "HP:0000589",
      "HP:0000609",
      "HP:0000612",
      "HP:0000819",
      "HP:0000822",
      "HP:0000961",
      "HP:0000969",
      "HP:0001290",
      "HP:0001488",
      "HP:0001561",
      "HP:0001629",
      "HP:0001631",
      "HP:0001635",
      "HP:0001655",
      "HP:0001671",
      "HP:0002003",
      "HP:0002094",
      "HP:0002104",
      "HP:0002119",
      "HP:0002282",
      "HP:0004502",
      "HP:0005133",
      "HP:0005280",
      "HP:0005989",
      "HP:0006610",
      "HP:0007700",
      "HP:0007957",
      "HP:0008551",
      "HP:0008936",
      "HP:0009062",
      "HP:0009800",
      "HP:0009879",
      "HP:0011220",
      "HP:0012785",
      "HP:0100598"
    ],
    [
      "OMIM:616975"
    ]
  ],
  [
    [
      "HP:0000341",
      "HP:0000426",
```

"HP:0000463",
"HP:0000490",
"HP:0000505",
"HP:0000939",
"HP:0001250",
"HP:0001284",
"HP:0001320",
"HP:0001347",
"HP:0002079",
"HP:0002093",
"HP:0002119",
"HP:0002263",
"HP:0002353",
"HP:0002376",
"HP:0002380",
"HP:0002553",
"HP:0003477",
"HP:0006829",
"HP:0011968",
"HP:0012697",
"HP:0012736",
"HP:0030236"
],
[
"OMIM:616900",
"ORPHA:488632"
]
],
[
[
"HP:0000365",
"HP:0000592",
"HP:0001371",
"HP:0001433",
"HP:0002757",
"HP:0003116",
"HP:0003155",
"HP:0004322",
"HP:0004349"
],
[
"OMIM:610968"
]
],
[
[
"HP:0000510",
"HP:0000750",
"HP:0000957",
"HP:0001156",
"HP:0001344",
"HP:0001363",
"HP:0001763",
"HP:0001822",
"HP:0002194",
"HP:0004322",

```
      "HP:0007565"
    ],
    [
      "OMIM:250410",
      "ORPHA:166035"
    ]
  ],
  [
    [
      "HP:0000280",
      "HP:0000349",
      "HP:0000403",
      "HP:0000414",
      "HP:0000729",
      "HP:0000739",
      "HP:0000750",
      "HP:0001156",
      "HP:0001212",
      "HP:0001263",
      "HP:0001992",
      "HP:0003112",
      "HP:0003355",
      "HP:0004322",
      "HP:0008155",
      "HP:0010471",
      "HP:0010529",
      "HP:0011893",
      "HP:0030352",
      "HP:0200055"
    ],
    [
      "OMIM:266265",
      "ORPHA:99843"
    ]
  ],
  [
    [
      "HP:0001250",
      "HP:0001987",
      "HP:0002181",
      "HP:0003218",
      "HP:0005961",
      "HP:0008358",
      "HP:0011966",
      "HP:0032397"
    ],
    [
      "OMIM:215700",
      "ORPHA:247525"
    ]
  ],
  [
    [
      "HP:0000248",
      "HP:0000384",
      "HP:0000486",
```

"HP:0000520",
"HP:0000639",
"HP:0000826",
"HP:0000954",
"HP:0001239",
"HP:0001263",
"HP:0001290",
"HP:0001845",
"HP:0002069",
"HP:0002353",
"HP:0002421",
"HP:0002987",
"HP:0003739",
"HP:0006191",
"HP:0006380",
"HP:0006829",
"HP:0007359",
"HP:0008947"
],
[
"OMIM:616900",
"ORPHA:488632"
]
],
[
[
"HP:0000076",
"HP:0000098",
"HP:0000107",
"HP:0000160",
"HP:0000164",
"HP:0000187",
"HP:0000218",
"HP:0000221",
"HP:0000252",
"HP:0000256",
"HP:0000286",
"HP:0000316",
"HP:0000319",
"HP:0000347",
"HP:0000349",
"HP:0000358",
"HP:0000365",
"HP:0000368",
"HP:0000407",
"HP:0000414",
"HP:0000463",
"HP:0000490",
"HP:0000494",
"HP:0000525",
"HP:0000568",
"HP:0000581",
"HP:0000589",
"HP:0000601",
"HP:0000648",
"HP:0000659",

```
      "HP:0000708",
      "HP:0000750",
      "HP:0000752",
      "HP:0000957",
      "HP:0000998",
      "HP:0001159",
      "HP:0001250",
      "HP:0001252",
      "HP:0001263",
      "HP:0001270",
      "HP:0001285",
      "HP:0001290",
      "HP:0001344",
      "HP:0001385",
      "HP:0001510",
      "HP:0001511",
      "HP:0001629",
      "HP:0001643",
      "HP:0001655",
      "HP:0001671",
      "HP:0001734",
      "HP:0001792",
      "HP:0002007",
      "HP:0002020",
      "HP:0002021",
      "HP:0002247",
      "HP:0002273",
      "HP:0002311",
      "HP:0002557",
      "HP:0002650",
      "HP:0002938",
      "HP:0003186",
      "HP:0003307",
      "HP:0004209",
      "HP:0004322",
      "HP:0004467",
      "HP:0004532",
      "HP:0007018",
      "HP:0011229",
      "HP:0011328",
      "HP:0011968",
      "HP:0012168",
      "HP:0012170",
      "HP:0012471",
      "HP:0045025",
      "HP:0200055",
      "HP:0200055",
      "HP:0410030"
    ],
    [
      "OMIM:616975"
    ]
  ],
  [
    [
      "HP:0000252",
```

```
      "HP:0001250",
      "HP:0001259",
      "HP:0001263",
      "HP:0001344",
      "HP:0001664",
      "HP:0001943",
      "HP:0001946",
      "HP:0002061",
      "HP:0002133",
      "HP:0002151",
      "HP:0002913",
      "HP:0002919",
      "HP:0002925",
      "HP:0003128",
      "HP:0003201",
      "HP:0003236",
      "HP:0006846",
      "HP:0008936",
      "HP:0011675",
      "HP:0012444",
      "HP:0040145",
      "HP:0045045"
    ],
    [
      "OMIM:616878",
      "ORPHA:480864"
    ]
  ],
  [
    [
      "HP:0001300",
      "HP:0002063",
      "HP:0002067",
      "HP:0002141",
      "HP:0002172",
      "HP:0002353",
      "HP:0002527"
    ],
    [
      "OMIM:260300",
      "ORPHA:171695"
    ]
  ],
  [
    [
      "HP:0000044",
      "HP:0001029",
      "HP:0001875",
      "HP:0002110",
      "HP:0004322",
      "HP:0006530",
      "HP:0032252"
    ],
    [
      "OMIM:604173",
      "ORPHA:221046"
```

```
    ]
  ],
  [
    [
      "HP:0000107",
      "HP:0000256",
      "HP:0000774",
      "HP:0001328",
      "HP:0002119",
      "HP:0002573",
      "HP:0009882",
      "HP:0011220",
      "HP:0030255"
    ],
    [
      "OMIM:616638",
      "ORPHA:457485"
    ]
  ],
  [
    [
      "HP:0001627",
      "HP:0001972",
      "HP:0002011",
      "HP:0011873",
      "HP:0011893"
    ],
    [
      "CCRD:25",
      "OMIM:105650",
      "ORPHA:124"
    ]
  ],
  [
    [
      "HP:0000518",
      "HP:0000790",
      "HP:0000829",
      "HP:0001281",
      "HP:0002024",
      "HP:0002321",
      "HP:0002728",
      "HP:0002901",
      "HP:0006297",
      "HP:0008207"
    ],
    [
      "OMIM:240300",
      "ORPHA:3453"
    ]
  ],
  [
    [
      "HP:0000028",
      "HP:0000218",
      "HP:0000239",
```

```
      "HP:0000248",
      "HP:0000368",
      "HP:0000494",
      "HP:0000520",
      "HP:0001561",
      "HP:0001631",
      "HP:0006610",
      "HP:0010709",
      "HP:0010713",
      "HP:0011220",
      "HP:0011800"
    ],
    [
      "OMIM:101200",
      "ORPHA:87"
    ]
  ],
  [
    [
      "HP:0003074",
      "HP:0004904",
      "HP:0005562",
      "HP:0012090",
      "HP:0025329",
      "HP:0040217"
    ],
    [
      "OMIM:137920",
      "ORPHA:93111"
    ]
  ],
  [
    [
      "HP:0000218",
      "HP:0000467",
      "HP:0001249",
      "HP:0001324",
      "HP:0001623",
      "HP:0002747",
      "HP:0002751",
      "HP:0003391",
      "HP:0006380",
      "HP:0010546",
      "HP:0031936",
      "HP:0040129"
    ],
    [
      "OMIM:610687"
    ]
  ],
  [
    [
      "HP:0001265",
      "HP:0001265",
      "HP:0001271",
      "HP:0003380",
```

```
      "HP:0003400",
      "HP:0005406",
      "HP:0007021",
      "HP:0011096",
      "HP:0012332",
      "HP:0030972",
      "HP:0031860",
      "HP:0031917",
      "HP:0040132"
    ],
    [
      "OMIM:201300"
    ]
  ],
  [
    [
      "HP:0011897",
      "HP:0032434",
      "HP:0032435",
      "HP:0200042"
    ],
    [
      "OMIM:116920",
      "ORPHA:99842"
    ]
  ],
  [
    [
      "HP:0000032",
      "HP:0000365",
      "HP:0000708",
      "HP:0000729",
      "HP:0000750",
      "HP:0001249",
      "HP:0001250",
      "HP:0001270",
      "HP:0001344",
      "HP:0001626",
      "HP:0011968",
      "HP:0030148"
    ],
    [
      "OMIM:617516"
    ]
  ],
  [
    [
      "HP:0000218",
      "HP:0000343",
      "HP:0000369",
      "HP:0000851",
      "HP:0001007",
      "HP:0001522",
      "HP:0001561",
      "HP:0002015",
      "HP:0002033",
```

```
      "HP:0002509",
      "HP:0002871",
      "HP:0003196",
      "HP:0007598",
      "HP:0007633",
      "HP:0008872",
      "HP:0008936",
      "HP:0010576",
      "HP:0011968",
      "HP:0025116",
      "HP:0031605",
      "HP:0100022",
      "HP:0100259"
    ],
    [
      "OMIM:617527",
      "ORPHA:521426"
    ]
  ],
  [
    [
      "HP:0000790",
      "HP:0001385",
      "HP:0001903",
      "HP:0001942",
      "HP:0001974",
      "HP:0003159",
      "HP:0008672",
      "HP:0011227",
      "HP:0011280",
      "HP:0012085",
      "HP:0012100"
    ],
    [
      "OMIM:260000",
      "ORPHA:93599"
    ]
  ],
  [
    [
      "HP:0000077",
      "HP:0000365",
      "HP:0000453",
      "HP:0000478",
      "HP:0000750",
      "HP:0000826",
      "HP:0001252",
      "HP:0001263",
      "HP:0001290",
      "HP:0001344",
      "HP:0001627",
      "HP:0002650",
      "HP:0007033",
      "HP:0010864",
      "HP:0011968",
      "HP:0025161"
```

```
    ],
    [
      "OMIM:616975"
    ]
  ],
  [
    [
      "HP:0001252",
      "HP:0001290",
      "HP:0001382",
      "HP:0006443",
      "HP:0008404",
      "HP:0009781"
    ],
    [
      "OMIM:161200",
      "ORPHA:2614"
    ]
  ],
  [
    [
      "HP:0000221",
      "HP:0000966",
      "HP:0000973",
      "HP:0003401",
      "HP:0003403",
      "HP:0007089"
    ],
    [
      "OMIM:105120",
      "ORPHA:85448"
    ]
  ],
  [
    [
      "HP:0000343",
      "HP:0000400",
      "HP:0000414",
      "HP:0001649",
      "HP:0002069",
      "HP:0002104",
      "HP:0002536",
      "HP:0002719",
      "HP:0005957",
      "HP:0005972",
      "HP:0007204",
      "HP:0011294",
      "HP:0030917",
      "HP:0100259"
    ],
    [
      "OMIM:617527",
      "ORPHA:521426"
    ]
  ],
  [
```

[
"HP:0000486",
"HP:0000510",
"HP:0000608",
"HP:0001263",
"HP:0001328",
"HP:0001513",
"HP:0007843"
],
[
"OMIM:209900"
]
],
[
[
"HP:0000098",
"HP:0000545",
"HP:0000767",
"HP:0000963",
"HP:0001166",
"HP:0001653",
"HP:0002616",
"HP:0002650",
"HP:0002751",
"HP:0004325",
"HP:0012019"
],
[
"OMIM:154700",
"ORPHA:284963"
]
],
[
[
"HP:0000233",
"HP:0000260",
"HP:0000316",
"HP:0000347",
"HP:0000369",
"HP:0000463",
"HP:0000470",
"HP:0000474",
"HP:0000582",
"HP:0001239",
"HP:0001274",
"HP:0001305",
"HP:0001511",
"HP:0001627",
"HP:0001942",
"HP:0002002",
"HP:0002119",
"HP:0002335",
"HP:0003196",
"HP:0005280",
"HP:0005684",
"HP:0006380",

```
      "HP:0006466",
      "HP:0010880",
      "HP:0012210",
      "HP:0012385",
      "HP:0100806"
    ],
    [
      "OMIM:611182",
      "ORPHA:95428"
    ]
  ],
  [
    [
      "HP:0000486",
      "HP:0000545",
      "HP:0000582",
      "HP:0000664",
      "HP:0000739",
      "HP:0000750",
      "HP:0001156",
      "HP:0001249",
      "HP:0001250",
      "HP:0001252",
      "HP:0001508",
      "HP:0001763",
      "HP:0001956",
      "HP:0002020",
      "HP:0002099",
      "HP:0002108",
      "HP:0002121",
      "HP:0002360",
      "HP:0002714",
      "HP:0004209",
      "HP:0004220",
      "HP:0004322",
      "HP:0011342",
      "HP:0012168",
      "HP:0100716"
    ],
    [
      "OMIM:182290",
      "ORPHA:819"
    ]
  ],
  [
    [
      "HP:0000225",
      "HP:0000421",
      "HP:0000967",
      "HP:0001873",
      "HP:0004866",
      "HP:0008148",
      "HP:0011870",
      "HP:0011871",
      "HP:0011877",
      "HP:0012343"
```

```
    ],
    [
      "OMIM:231200",
      "ORPHA:274"
    ]
  ],
  [
    [
      "HP:0001290",
      "HP:0001433",
      "HP:0001522",
      "HP:0001873",
      "HP:0003656",
      "HP:0004325",
      "HP:0007479"
    ],
    [
      "OMIM:608013",
      "ORPHA:85212"
    ]
  ],
  [
    [
      "HP:0000028",
      "HP:0000076",
      "HP:0000098",
      "HP:0000107",
      "HP:0000160",
      "HP:0000187",
      "HP:0000221",
      "HP:0000252",
      "HP:0000256",
      "HP:0000286",
      "HP:0000316",
      "HP:0000319",
      "HP:0000325",
      "HP:0000347",
      "HP:0000349",
      "HP:0000365",
      "HP:0000407",
      "HP:0000414",
      "HP:0000453",
      "HP:0000463",
      "HP:0000486",
      "HP:0000490",
      "HP:0000494",
      "HP:0000505",
      "HP:0000525",
      "HP:0000534",
      "HP:0000568",
      "HP:0000581",
      "HP:0000582",
      "HP:0000589",
      "HP:0000601",
      "HP:0000648",
      "HP:0000659",
```

"HP:0000708",
"HP:0000750",
"HP:0000752",
"HP:0000957",
"HP:0000998",
"HP:0001159",
"HP:0001252",
"HP:0001263",
"HP:0001273",
"HP:0001285",
"HP:0001290",
"HP:0001320",
"HP:0001344",
"HP:0001385",
"HP:0001510",
"HP:0001511",
"HP:0001572",
"HP:0001629",
"HP:0001643",
"HP:0001655",
"HP:0001671",
"HP:0001734",
"HP:0001792",
"HP:0001999",
"HP:0002007",
"HP:0002020",
"HP:0002021",
"HP:0002119",
"HP:0002194",
"HP:0002247",
"HP:0002273",
"HP:0002384",
"HP:0002557",
"HP:0002650",
"HP:0002938",
"HP:0003186",
"HP:0003307",
"HP:0003429",
"HP:0004209",
"HP:0004322",
"HP:0004467",
"HP:0004532",
"HP:0006808",
"HP:0007018",
"HP:0007361",
"HP:0010490",
"HP:0010772",
"HP:0011081",
"HP:0011328",
"HP:0011968",
"HP:0012448",
"HP:0012471",
"HP:0025100",
"HP:0030026",
"HP:0030301",
"HP:0045025",

```
        "HP:0100704",
        "HP:0200055",
        "HP:0410030"
      ],
      [
        "OMIM:616975"
      ]
    ],
    [
      [
        "HP:0000750",
        "HP:0002099",
        "HP:0002750",
        "HP:0004322",
        "HP:0010535",
        "HP:0010862"
      ],
      [
        "OMIM:604804"
      ]
    ],
    [
      [
        "HP:0000543",
        "HP:0001250",
        "HP:0001276",
        "HP:0001290",
        "HP:0001336",
        "HP:0001992",
        "HP:0002093",
        "HP:0002151",
        "HP:0002353",
        "HP:0002421",
        "HP:0003323",
        "HP:0006801",
        "HP:0006892",
        "HP:0007305",
        "HP:0011470",
        "HP:0025517",
        "HP:0031358",
        "HP:0100952"
      ],
      [
        "OMIM:616211"
      ]
    ],
    [
      [
        "HP:0000233",
        "HP:0000325",
        "HP:0000369",
        "HP:0000460",
        "HP:0000592",
        "HP:0000963",
        "HP:0000973",
        "HP:0001075",
```

```
      "HP:0001263",
      "HP:0001382",
      "HP:0001385",
      "HP:0001763",
      "HP:0002194",
      "HP:0007392",
      "HP:0008887",
      "HP:0010648"
    ],
    [
      "OMIM:612940",
      "ORPHA:357064"
    ]
  ],
  [
    [
      "HP:0000233",
      "HP:0000240",
      "HP:0000343",
      "HP:0001156",
      "HP:0001263",
      "HP:0001377",
      "HP:0001488",
      "HP:0001561",
      "HP:0002205",
      "HP:0002750",
      "HP:0003022",
      "HP:0003066",
      "HP:0003097",
      "HP:0004322",
      "HP:0009803",
      "HP:0010049"
    ],
    [
      "OMIM:231050"
    ]
  ],
  [
    [
      "HP:0000020",
      "HP:0001258",
      "HP:0001260",
      "HP:0001348",
      "HP:0002064",
      "HP:0002355",
      "HP:0003487",
      "HP:0008081"
    ],
    [
      "OMIM:616907",
      "ORPHA:488594"
    ]
  ],
  [
    [
      "HP:0000121",
```

      "HP:0000973",
      "HP:0001627",
      "HP:0001999"
    ],
    [
      "OMIM:617402"
    ]
  ],
  [
    [
      "HP:0000218",
      "HP:0000219",
      "HP:0000237",
      "HP:0000316",
      "HP:0000400",
      "HP:0000463",
      "HP:0000508",
      "HP:0001522",
      "HP:0002007",
      "HP:0005280",
      "HP:0010109",
      "HP:0010708",
      "HP:0010713",
      "HP:0011318",
      "HP:0011330",
      "HP:0011800"
    ],
    [
      "OMIM:101200",
      "ORPHA:87"
    ]
  ],
  [
    [
      "HP:0000083",
      "HP:0000085",
      "HP:0000926",
      "HP:0001156",
      "HP:0001407",
      "HP:0001999",
      "HP:0002079",
      "HP:0002119",
      "HP:0002652",
      "HP:0002789",
      "HP:0005562",
      "HP:0009826",
      "HP:0011839",
      "HP:0030747"
    ],
    [
      "OMIM:617425"
    ]
  ],
  [
    [
      "HP:0000238",

```
      "HP:0000256",
      "HP:0000316",
      "HP:0000369",
      "HP:0000470",
      "HP:0000486",
      "HP:0000508",
      "HP:0000767",
      "HP:0001250",
      "HP:0002967",
      "HP:0004322",
      "HP:0004482",
      "HP:0008070",
      "HP:0011342"
    ],
    [
      "OMIM:609942"
    ]
  ],
  [
    [
      "HP:0000023",
      "HP:0000028",
      "HP:0000098",
      "HP:0000119",
      "HP:0000621",
      "HP:0000973",
      "HP:0001250",
      "HP:0001290",
      "HP:0001371",
      "HP:0001385",
      "HP:0001519",
      "HP:0001522",
      "HP:0001627",
      "HP:0001635",
      "HP:0001671",
      "HP:0001999",
      "HP:0002751",
      "HP:0004942",
      "HP:0007552"
    ],
    [
      "OMIM:617403"
    ]
  ],
  [
    [
      "HP:0000077",
      "HP:0000316",
      "HP:0000357",
      "HP:0000365",
      "HP:0000385",
      "HP:0000395",
      "HP:0000408",
      "HP:0000431",
      "HP:0000453",
      "HP:0000567",
```

```
      "HP:0000589",
      "HP:0000612",
      "HP:0000708",
      "HP:0001249",
      "HP:0001250",
      "HP:0001252",
      "HP:0001263",
      "HP:0001290",
      "HP:0001344",
      "HP:0001371",
      "HP:0001385",
      "HP:0001510",
      "HP:0001511",
      "HP:0001649",
      "HP:0002098",
      "HP:0002355",
      "HP:0002376",
      "HP:0002650",
      "HP:0002944",
      "HP:0003698",
      "HP:0004322",
      "HP:0004384",
      "HP:0004502",
      "HP:0004755",
      "HP:0011849",
      "HP:0012368",
      "HP:0012803"
    ],
    [
      "OMIM:616975"
    ]
  ],
  [
    [
      "HP:0004390",
      "HP:0032451",
      "HP:0032454"
    ],
    [
      "CCRD:89",
      "OMIM:175200",
      "ORPHA:2869"
    ]
  ],
  [
    [
      "HP:0007556",
      "HP:0007559",
      "HP:0010765"
    ],
    [
      "OMIM:144200",
      "ORPHA:2199"
    ]
  ],
  [
```

```
    [
      "HP:0000750",
      "HP:0001263",
      "HP:0001276",
      "HP:0002188",
      "HP:0010851",
      "HP:0011167"
    ],
    [
      "OMIM:612164"
    ]
  ],
  [
    [
      "HP:0001678",
      "HP:0001688",
      "HP:0005184",
      "HP:0030682"
    ],
    [
      "OMIM:616249"
    ]
  ],
  [
    [
      "HP:0000218",
      "HP:0000410",
      "HP:0000482",
      "HP:0000567",
      "HP:0000568",
      "HP:0000612",
      "HP:0000677",
      "HP:0000750",
      "HP:0001597",
      "HP:0004476",
      "HP:0007678",
      "HP:0008494",
      "HP:0012210"
    ],
    [
      "OMIM:113620",
      "ORPHA:1297"
    ]
  ],
  [
    [
      "HP:0000252",
      "HP:0001263",
      "HP:0001270",
      "HP:0001344",
      "HP:0002353",
      "HP:0010864",
      "HP:0012443"
    ],
    [
      "OMIM:608393"
```

```
      ]
    ],
    [
      [
        "HP:0000160",
        "HP:0000218",
        "HP:0000336",
        "HP:0000407",
        "HP:0000411",
        "HP:0000684",
        "HP:0000703",
        "HP:0002650",
        "HP:0002757",
        "HP:0004322",
        "HP:0004349",
        "HP:0004592",
        "HP:0005280",
        "HP:0011220",
        "HP:0011800",
        "HP:0045086"
      ],
      [
        "OMIM:613849"
      ]
    ],
    [
      [
        "HP:0001284",
        "HP:0001761",
        "HP:0003431",
        "HP:0003448",
        "HP:0006937",
        "HP:0008959",
        "HP:0031810"
      ],
      [
        "OMIM:162500",
        "ORPHA:640"
      ]
    ],
    [
      [
        "HP:0000635",
        "HP:0001100",
        "HP:0002226",
        "HP:0002227",
        "HP:0007894",
        "HP:0008527"
      ],
      [
        "OMIM:103500",
        "ORPHA:42665"
      ]
    ],
    [
      [
```

"HP:0000028",
"HP:0000047",
"HP:0000076",
"HP:0000098",
"HP:0000107",
"HP:0000160",
"HP:0000164",
"HP:0000187",
"HP:0000218",
"HP:0000221",
"HP:0000252",
"HP:0000256",
"HP:0000286",
"HP:0000316",
"HP:0000319",
"HP:0000325",
"HP:0000347",
"HP:0000349",
"HP:0000365",
"HP:0000407",
"HP:0000414",
"HP:0000453",
"HP:0000463",
"HP:0000490",
"HP:0000494",
"HP:0000525",
"HP:0000534",
"HP:0000568",
"HP:0000581",
"HP:0000582",
"HP:0000589",
"HP:0000598",
"HP:0000601",
"HP:0000648",
"HP:0000659",
"HP:0000708",
"HP:0000717",
"HP:0000750",
"HP:0000752",
"HP:0000957",
"HP:0000998",
"HP:0001159",
"HP:0001249",
"HP:0001250",
"HP:0001252",
"HP:0001273",
"HP:0001285",
"HP:0001290",
"HP:0001344",
"HP:0001385",
"HP:0001510",
"HP:0001511",
"HP:0001629",
"HP:0001643",
"HP:0001655",
"HP:0001671",

"HP:0001734",
"HP:0001792",
"HP:0002007",
"HP:0002015",
"HP:0002020",
"HP:0002021",
"HP:0002247",
"HP:0002273",
"HP:0002557",
"HP:0002650",
"HP:0002938",
"HP:0003186",
"HP:0003307",
"HP:0003429",
"HP:0004209",
"HP:0004322",
"HP:0004467",
"HP:0004532",
"HP:0006808",
"HP:0007018",
"HP:0010490",
"HP:0010772",
"HP:0011328",
"HP:0012448",
"HP:0012471",
"HP:0045025",
"HP:0200055",
"HP:0410030"
],
[
"OMIM:616975"
]
],
[
[
"HP:0000107",
"HP:0000126",
"HP:0000154",
"HP:0000252",
"HP:0000331",
"HP:0000400",
"HP:0000414",
"HP:0000486",
"HP:0000582",
"HP:0000952",
"HP:0001252",
"HP:0001263",
"HP:0001558",
"HP:0001591",
"HP:0001792",
"HP:0001883",
"HP:0002000",
"HP:0002098",
"HP:0002236",
"HP:0002944",
"HP:0007099",

```
      "HP:0008689",
      "HP:0009890",
      "HP:0011819",
      "HP:0012385",
      "HP:0012450",
      "HP:0012523",
      "HP:0100730",
      "HP:0410018"
    ],
    [
      "OMIM:602342",
      "ORPHA:487825"
    ]
  ],
  [
    [
      "HP:0000169",
      "HP:0009719",
      "HP:0009722",
      "HP:0009730"
    ],
    [
      "OMIM:613254"
    ]
  ],
  [
    [
      "HP:0000239",
      "HP:0000242",
      "HP:0000316",
      "HP:0000680",
      "HP:0000774",
      "HP:0000894",
      "HP:0002007",
      "HP:0002645",
      "HP:0002650",
      "HP:0002866",
      "HP:0004322",
      "HP:0005259",
      "HP:0005280",
      "HP:0006297",
      "HP:0008788",
      "HP:0008804",
      "HP:0011223",
      "HP:0011800",
      "HP:0100864"
    ],
    [
      "OMIM:119600",
      "ORPHA:1452"
    ]
  ],
  [
    [
      "HP:0000505",
      "HP:0000613",
```

```
      "HP:0000662",
      "HP:0001123"
    ],
    [
      "OMIM:120970",
      "ORPHA:1872"
    ]
  ],
  [
    [
      "HP:0000365",
      "HP:0001319",
      "HP:0001410",
      "HP:0001531",
      "HP:0001712",
      "HP:0001941",
      "HP:0002033",
      "HP:0002098",
      "HP:0002126",
      "HP:0002151",
      "HP:0002490",
      "HP:0002910",
      "HP:0003128",
      "HP:0008872",
      "HP:0040288"
    ],
    [
      "OMIM:616974",
      "ORPHA:478042"
    ]
  ],
  [
    [
      "HP:0001250",
      "HP:0001263",
      "HP:0001290",
      "HP:0001344",
      "HP:0011182"
    ],
    [
      "OMIM:614959"
    ]
  ],
  [
    [
      "HP:0001663",
      "HP:0001688",
      "HP:0030682"
    ],
    [
      "OMIM:163800"
    ]
  ],
  [
    [
      "HP:0000964",
```

"HP:0001010",
"HP:0001263",
"HP:0001386",
"HP:0001433",
"HP:0001892",
"HP:0001945",
"HP:0002013",
"HP:0002014",
"HP:0002090",
"HP:0003212",
"HP:0004396",
"HP:0008070",
"HP:0012735"
],
[
"OMIM:214500",
"ORPHA:167"
]
],
[
[
"HP:0000563",
"HP:0001260",
"HP:0001310",
"HP:0002061",
"HP:0002066",
"HP:0002070",
"HP:0006986"
],
[
"OMIM:616907",
"ORPHA:488594"
]
],
[
[
"HP:0001762",
"HP:0005792",
"HP:0005855",
"HP:0006385",
"HP:0006487"
],
[
"OMIM:259440"
]
],
[
[
"HP:0000252",
"HP:0000365",
"HP:0000648",
"HP:0000750",
"HP:0001263",
"HP:0001332",
"HP:0001508",
"HP:0001943",

```
      "HP:0002120",
      "HP:0002307",
      "HP:0003128",
      "HP:0003200",
      "HP:0003535",
      "HP:0003688",
      "HP:0003781",
      "HP:0008936",
      "HP:0008947",
      "HP:0011968",
      "HP:0012157",
      "HP:0012707",
      "HP:0012708",
      "HP:0012751",
      "HP:0200134"
    ],
    [
      "OMIM:614739",
      "ORPHA:352328"
    ]
  ],
  [
    [
      "HP:0000218",
      "HP:0000574",
      "HP:0000689",
      "HP:0001162",
      "HP:0001212",
      "HP:0001250",
      "HP:0001263",
      "HP:0001511",
      "HP:0004209",
      "HP:0007236",
      "HP:0010554"
    ],
    [
      "OMIM:174300",
      "ORPHA:2919"
    ]
  ],
  [
    [
      "HP:0001397",
      "HP:0001405",
      "HP:0003124",
      "HP:0003155",
      "HP:0003236",
      "HP:0010837",
      "HP:0011967"
    ],
    [
      "OMIM:616829",
      "ORPHA:466703"
    ]
  ],
  [
```

[
  "HP:0000028",
  "HP:0000047",
  "HP:0000076",
  "HP:0000098",
  "HP:0000160",
  "HP:0000187",
  "HP:0000218",
  "HP:0000221",
  "HP:0000252",
  "HP:0000256",
  "HP:0000286",
  "HP:0000316",
  "HP:0000319",
  "HP:0000325",
  "HP:0000347",
  "HP:0000349",
  "HP:0000365",
  "HP:0000407",
  "HP:0000414",
  "HP:0000453",
  "HP:0000463",
  "HP:0000483",
  "HP:0000490",
  "HP:0000494",
  "HP:0000525",
  "HP:0000568",
  "HP:0000581",
  "HP:0000582",
  "HP:0000589",
  "HP:0000598",
  "HP:0000601",
  "HP:0000648",
  "HP:0000659",
  "HP:0000691",
  "HP:0000708",
  "HP:0000729",
  "HP:0000750",
  "HP:0000752",
  "HP:0000957",
  "HP:0000998",
  "HP:0001159",
  "HP:0001250",
  "HP:0001252",
  "HP:0001273",
  "HP:0001285",
  "HP:0001290",
  "HP:0001317",
  "HP:0001344",
  "HP:0001385",
  "HP:0001510",
  "HP:0001511",
  "HP:0001629",
  "HP:0001643",
  "HP:0001655",
  "HP:0001671",

"HP:0001734",
"HP:0001792",
"HP:0002007",
"HP:0002020",
"HP:0002021",
"HP:0002033",
"HP:0002119",
"HP:0002247",
"HP:0002273",
"HP:0002334",
"HP:0002360",
"HP:0002557",
"HP:0002650",
"HP:0002938",
"HP:0003186",
"HP:0003307",
"HP:0003429",
"HP:0004209",
"HP:0004322",
"HP:0004467",
"HP:0004532",
"HP:0006808",
"HP:0007018",
"HP:0007361",
"HP:0010490",
"HP:0010772",
"HP:0010862",
"HP:0011230",
"HP:0011328",
"HP:0011848",
"HP:0011968",
"HP:0012448",
"HP:0012471",
"HP:0025100",
"HP:0030301",
"HP:0045025",
"HP:0200055",
"HP:0410030"
],
[
"OMIM:616975"
]
],
[
[
"HP:0000510",
"HP:0000580",
"HP:0000662",
"HP:0001123",
"HP:0007663",
"HP:0007843",
"HP:0012512",
"HP:0025159"
],
[
"OMIM:617433"

```
    ]
  ],
  [
    [
      "HP:0000256",
      "HP:0000486",
      "HP:0000639",
      "HP:0001249",
      "HP:0001250",
      "HP:0001344",
      "HP:0002079",
      "HP:0002376",
      "HP:0002522",
      "HP:0006829",
      "HP:0006970",
      "HP:0011344"
    ],
    [
      "OMIM:616900",
      "ORPHA:488632"
    ]
  ],
  [
    [
      "HP:0000286",
      "HP:0000325",
      "HP:0000331",
      "HP:0000343",
      "HP:0000358",
      "HP:0000369",
      "HP:0000455",
      "HP:0000473",
      "HP:0000494",
      "HP:0000506",
      "HP:0000939",
      "HP:0001371",
      "HP:0001572",
      "HP:0002463",
      "HP:0002808",
      "HP:0005280",
      "HP:0006191",
      "HP:0011344",
      "HP:0100543",
      "HP:0200055"
    ],
    [
      "OMIM:617694"
    ]
  ],
  [
    [
      "HP:0000252",
      "HP:0000750",
      "HP:0001249",
      "HP:0001252",
      "HP:0001257",
```

```
      "HP:0001263",
      "HP:0002355"
    ],
    [
      "OMIM:614249"
    ]
  ],
  [
    [
      "HP:0001371",
      "HP:0002104",
      "HP:0002540",
      "HP:0002792",
      "HP:0002793",
      "HP:0002878",
      "HP:0003202",
      "HP:0003388",
      "HP:0003691",
      "HP:0008959",
      "HP:0009027",
      "HP:0009053",
      "HP:0010307",
      "HP:0010535",
      "HP:0030210"
    ],
    [
      "OMIM:615120"
    ]
  ],
  [
    [
      "HP:0000238",
      "HP:0001249",
      "HP:0002370",
      "HP:0002454",
      "HP:0003155",
      "HP:0003236",
      "HP:0012503"
    ],
    [
      "OMIM:234200",
      "ORPHA:157850"
    ]
  ],
  [
    [
      "HP:0000077",
      "HP:0000154",
      "HP:0000316",
      "HP:0000453",
      "HP:0000463",
      "HP:0000664",
      "HP:0000708",
      "HP:0000722",
      "HP:0000729",
      "HP:0000733",
```

```
      "HP:0000739",
      "HP:0000750",
      "HP:0001263",
      "HP:0001627",
      "HP:0002360",
      "HP:0002474",
      "HP:0002650",
      "HP:0007015",
      "HP:0009889",
      "HP:0012372",
      "HP:0031468"
    ],
    [
      "OMIM:616975"
    ]
  ],
  [
    [
      "HP:0000752",
      "HP:0001249",
      "HP:0002750",
      "HP:0005338"
    ],
    [
      "OMIM:190350"
    ]
  ],
  [
    [
      "HP:0000032",
      "HP:0000047",
      "HP:0000077",
      "HP:0000085",
      "HP:0000086",
      "HP:0000277",
      "HP:0000308",
      "HP:0000356",
      "HP:0000358",
      "HP:0000365",
      "HP:0000369",
      "HP:0000478",
      "HP:0000486",
      "HP:0000639",
      "HP:0000666",
      "HP:0000708",
      "HP:0000750",
      "HP:0001249",
      "HP:0001252",
      "HP:0001270",
      "HP:0001290",
      "HP:0001344",
      "HP:0001626",
      "HP:0001629",
      "HP:0001631",
      "HP:0001643",
      "HP:0001660",
```

```
      "HP:0002090",
      "HP:0002817",
      "HP:0004736",
      "HP:0006532",
      "HP:0009601",
      "HP:0011968",
      "HP:0012304"
    ],
    [
      "OMIM:617516"
    ]
  ],
  [
    [
      "HP:0000543",
      "HP:0000649",
      "HP:0000666",
      "HP:0001257",
      "HP:0001332",
      "HP:0001583",
      "HP:0002300",
      "HP:0002421",
      "HP:0006958",
      "HP:0007015",
      "HP:0008947",
      "HP:0011471",
      "HP:0100543"
    ],
    [
      "OMIM:617560",
      "ORPHA:527497"
    ]
  ],
  [
    [
      "HP:0001250",
      "HP:0009719",
      "HP:0009721",
      "HP:0010614",
      "HP:0012733",
      "HP:0012736",
      "HP:0100804"
    ],
    [
      "OMIM:191100"
    ]
  ],
  [
    [
      "HP:0000365",
      "HP:0001662",
      "HP:0002789",
      "HP:0005184"
    ],
    [
      "OMIM:220400",
```

```
      "ORPHA:90647"
    ]
  ],
  [
    [
      "HP:0000028",
      "HP:0000054",
      "HP:0000252",
      "HP:0000280",
      "HP:0000316",
      "HP:0001935",
      "HP:0002669",
      "HP:0002714",
      "HP:0010864"
    ],
    [
      "OMIM:301040",
      "ORPHA:847"
    ]
  ],
  [
    [
      "HP:0000160",
      "HP:0000252",
      "HP:0000358",
      "HP:0000448",
      "HP:0000486",
      "HP:0000494",
      "HP:0000527",
      "HP:0000574",
      "HP:0000589",
      "HP:0000678",
      "HP:0000689",
      "HP:0000729",
      "HP:0000739",
      "HP:0000750",
      "HP:0001007",
      "HP:0001263",
      "HP:0002342",
      "HP:0002553",
      "HP:0002650",
      "HP:0002750",
      "HP:0008872",
      "HP:0009765",
      "HP:0010055",
      "HP:0010562",
      "HP:0011087",
      "HP:0011304"
    ],
    [
      "OMIM:613684",
      "ORPHA:353284"
    ]
  ],
  [
    [
```

```
      "HP:0000639",
      "HP:0000752",
      "HP:0001310",
      "HP:0001387",
      "HP:0001873",
      "HP:0001875",
      "HP:0002141",
      "HP:0002172",
      "HP:0002395",
      "HP:0002721",
      "HP:0002863",
      "HP:0007018",
      "HP:0011950"
    ],
    [
      "OMIM:159550",
      "ORPHA:2585"
    ]
  ],
  [
    [
      "HP:0000023",
      "HP:0000952",
      "HP:0001263",
      "HP:0001397",
      "HP:0001507",
      "HP:0002904",
      "HP:0003124",
      "HP:0003141",
      "HP:0003155",
      "HP:0003236",
      "HP:0006579",
      "HP:0010837",
      "HP:0012347",
      "HP:0025321",
      "HP:0031956",
      "HP:0031964",
      "HP:0100790"
    ],
    [
      "OMIM:616829",
      "ORPHA:466703"
    ]
  ],
  [
    [
      "HP:0000733",
      "HP:0000750",
      "HP:0001159",
      "HP:0001249",
      "HP:0001252",
      "HP:0001634",
      "HP:0001773",
      "HP:0002317",
      "HP:0031936"
    ],
```

```
    [
      "OMIM:615516",
      "ORPHA:329195"
    ]
  ],
  [
    [
      "HP:0000164",
      "HP:0000316",
      "HP:0000369",
      "HP:0000456",
      "HP:0000494",
      "HP:0000508",
      "HP:0000912",
      "HP:0001156",
      "HP:0001233",
      "HP:0001274",
      "HP:0001328",
      "HP:0001363",
      "HP:0001807",
      "HP:0004209",
      "HP:0004440",
      "HP:0012741",
      "HP:0100874"
    ],
    [
      "OMIM:304110",
      "ORPHA:1520"
    ]
  ],
  [
    [
      "HP:0000076",
      "HP:0000098",
      "HP:0000107",
      "HP:0000160",
      "HP:0000164",
      "HP:0000187",
      "HP:0000218",
      "HP:0000221",
      "HP:0000252",
      "HP:0000256",
      "HP:0000286",
      "HP:0000316",
      "HP:0000319",
      "HP:0000325",
      "HP:0000347",
      "HP:0000349",
      "HP:0000356",
      "HP:0000365",
      "HP:0000407",
      "HP:0000414",
      "HP:0000453",
      "HP:0000463",
      "HP:0000490",
      "HP:0000494",
```

"HP:0000525",
"HP:0000568",
"HP:0000581",
"HP:0000582",
"HP:0000589",
"HP:0000601",
"HP:0000648",
"HP:0000659",
"HP:0000708",
"HP:0000752",
"HP:0000954",
"HP:0000957",
"HP:0000998",
"HP:0001156",
"HP:0001159",
"HP:0001250",
"HP:0001252",
"HP:0001285",
"HP:0001290",
"HP:0001320",
"HP:0001385",
"HP:0001510",
"HP:0001511",
"HP:0001629",
"HP:0001643",
"HP:0001655",
"HP:0001734",
"HP:0001792",
"HP:0001943",
"HP:0001999",
"HP:0002007",
"HP:0002020",
"HP:0002021",
"HP:0002079",
"HP:0002119",
"HP:0002247",
"HP:0002273",
"HP:0002342",
"HP:0002557",
"HP:0002650",
"HP:0002938",
"HP:0003186",
"HP:0003307",
"HP:0003429",
"HP:0004209",
"HP:0004322",
"HP:0004467",
"HP:0004532",
"HP:0005968",
"HP:0006808",
"HP:0007018",
"HP:0007361",
"HP:0010490",
"HP:0010772",
"HP:0011304",
"HP:0011328",

      "HP:0011968",
      "HP:0012448",
      "HP:0012471",
      "HP:0030301",
      "HP:0045025",
      "HP:0200055",
      "HP:0410030"
    ],
    [
      "OMIM:616975"
    ]
  ],
  [
    [
      "HP:0000154",
      "HP:0000168",
      "HP:0000219",
      "HP:0000280",
      "HP:0000293",
      "HP:0000316",
      "HP:0000322",
      "HP:0000336",
      "HP:0000341",
      "HP:0000343",
      "HP:0000347",
      "HP:0000388",
      "HP:0000455",
      "HP:0000463",
      "HP:0000473",
      "HP:0000486",
      "HP:0000490",
      "HP:0000520",
      "HP:0000540",
      "HP:0000545",
      "HP:0000687",
      "HP:0000768",
      "HP:0001007",
      "HP:0001090",
      "HP:0001250",
      "HP:0001263",
      "HP:0001276",
      "HP:0001357",
      "HP:0001508",
      "HP:0001773",
      "HP:0002007",
      "HP:0002019",
      "HP:0002020",
      "HP:0002064",
      "HP:0002373",
      "HP:0002500",
      "HP:0002779",
      "HP:0003194",
      "HP:0003202",
      "HP:0003763",
      "HP:0004209",
      "HP:0004322",

"HP:0005280",
"HP:0005338",
"HP:0006959",
"HP:0007269",
"HP:0009623",
"HP:0009933",
"HP:0010800",
"HP:0012171",
"HP:0012471",
"HP:0200055",
"HP:0430028"
],
[
"OMIM:617616",
"ORPHA:513456"
]
],
[
[
"HP:0000218",
"HP:0000463",
"HP:0000465",
"HP:0000470",
"HP:0000494",
"HP:0000508",
"HP:0000962",
"HP:0001250",
"HP:0001631",
"HP:0001642",
"HP:0004482",
"HP:0005280",
"HP:0008064",
"HP:0008070",
"HP:0009891",
"HP:0010864"
],
[
"OMIM:115150",
"ORPHA:1340"
]
],
[
[
"HP:0000252",
"HP:0000712",
"HP:0000750",
"HP:0001320",
"HP:0001845",
"HP:0002079",
"HP:0002126",
"HP:0004322",
"HP:0004325",
"HP:0012758"
],
[
"OMIM:614833",

```
      "ORPHA:468631"
    ]
  ],
  [
    [
      "HP:0000750",
      "HP:0001263",
      "HP:0002069",
      "HP:0006821"
    ],
    [
      "OMIM:606854",
      "ORPHA:101070"
    ]
  ],
  [
    [
      "HP:0000119",
      "HP:0000707",
      "HP:0001510",
      "HP:0001876",
      "HP:0002721",
      "HP:0005528",
      "HP:0031413"
    ],
    [
      "OMIM:159550",
      "ORPHA:2585"
    ]
  ],
  [
    [
      "HP:0000662",
      "HP:0001123",
      "HP:0007663",
      "HP:0007737"
    ],
    [
      "OMIM:613750"
    ]
  ],
  [
    [
      "HP:0000107",
      "HP:0000510",
      "HP:0000512",
      "HP:0000561",
      "HP:0000649",
      "HP:0001156",
      "HP:0001249",
      "HP:0001363",
      "HP:0001596",
      "HP:0002223",
      "HP:0004322",
      "HP:0008064",
      "HP:0011344",
```

```
      "HP:0011968"
    ],
    [
      "OMIM:250410",
      "ORPHA:166035"
    ]
  ],
  [
    [
      "HP:0002322",
      "HP:0002451",
      "HP:0002527",
      "HP:0012332",
      "HP:0100543"
    ],
    [
      "OMIM:616840"
    ]
  ],
  [
    [
      "HP:0000718",
      "HP:0000726",
      "HP:0002354"
    ],
    [
      "OMIM:606889"
    ]
  ],
  [
    [
      "HP:0000218",
      "HP:0000343",
      "HP:0000592",
      "HP:0000974",
      "HP:0000978",
      "HP:0001065",
      "HP:0001083",
      "HP:0001382",
      "HP:0001634",
      "HP:0002650",
      "HP:0005116",
      "HP:0010812",
      "HP:0012532",
      "HP:0100775",
      "HP:0100790"
    ],
    [
      "OMIM:614816"
    ]
  ],
  [
    [
      "HP:0000639",
      "HP:0001251",
      "HP:0001260",
```

```
      "HP:0001903",
      "HP:0002066",
      "HP:0005528"
    ],
    [
      "OMIM:159550",
      "ORPHA:2585"
    ]
  ],
  [
    [
      "HP:0000341",
      "HP:0000426",
      "HP:0000463",
      "HP:0000505",
      "HP:0001250",
      "HP:0001320",
      "HP:0002079",
      "HP:0002093",
      "HP:0002119",
      "HP:0002263",
      "HP:0002353",
      "HP:0002553",
      "HP:0003477",
      "HP:0011968",
      "HP:0012697",
      "HP:0012736"
    ],
    [
      "OMIM:616900",
      "ORPHA:488632"
    ]
  ],
  [
    [
      "HP:0000077",
      "HP:0000396",
      "HP:0000400",
      "HP:0000453",
      "HP:0000478",
      "HP:0000486",
      "HP:0000505",
      "HP:0000540",
      "HP:0000582",
      "HP:0000750",
      "HP:0000878",
      "HP:0001156",
      "HP:0001252",
      "HP:0001263",
      "HP:0001290",
      "HP:0001513",
      "HP:0001631",
      "HP:0001773",
      "HP:0002099",
      "HP:0002650",
      "HP:0002779",
```

```
      "HP:0002791",
      "HP:0004322",
      "HP:0004467",
      "HP:0007110",
      "HP:0012372",
      "HP:0012443",
      "HP:0030055",
      "HP:0100704",
      "HP:0200055",
      "HP:0400008"
    ],
    [
      "OMIM:616975"
    ]
  ],
  [
    [
      "HP:0000218",
      "HP:0000252",
      "HP:0000400",
      "HP:0000407",
      "HP:0000444",
      "HP:0000490",
      "HP:0000568",
      "HP:0001176",
      "HP:0001249",
      "HP:0001321",
      "HP:0002650",
      "HP:0002684",
      "HP:0002808",
      "HP:0003121",
      "HP:0004322",
      "HP:0005671",
      "HP:0008897",
      "HP:0011470"
    ],
    [
      "OMIM:133540"
    ]
  ],
  [
    [
      "HP:0000733",
      "HP:0000750",
      "HP:0001250",
      "HP:0001252",
      "HP:0002079",
      "HP:0002188",
      "HP:0002353",
      "HP:0002376",
      "HP:0002540",
      "HP:0003763",
      "HP:0005484",
      "HP:0007333"
    ],
    [
```

```
      "OMIM:613454"
    ]
  ],
  [
    [
      "HP:0000054",
      "HP:0000154",
      "HP:0000212",
      "HP:0000256",
      "HP:0000316",
      "HP:0000369",
      "HP:0000463",
      "HP:0000520",
      "HP:0000921",
      "HP:0002007",
      "HP:0002944",
      "HP:0002984",
      "HP:0003022",
      "HP:0003086",
      "HP:0004322",
      "HP:0005280",
      "HP:0008439",
      "HP:0010804",
      "HP:0012646",
      "HP:0200055"
    ],
    [
      "OMIM:268310",
      "ORPHA:1507"
    ]
  ],
  [
    [
      "HP:0000047",
      "HP:0000048",
      "HP:0000054",
      "HP:0000062",
      "HP:0000369",
      "HP:0001047",
      "HP:0003146",
      "HP:0003196",
      "HP:0003462",
      "HP:0004691",
      "HP:0008734",
      "HP:0010569",
      "HP:0030087"
    ],
    [
      "OMIM:270400",
      "ORPHA:818"
    ]
  ],
  [
    [
      "HP:0000135",
      "HP:0000486",
```

"HP:0000543",
"HP:0000662",
"HP:0001513",
"HP:0007663"
],
[
"OMIM:615983"
]
],
[
[
"HP:0000194",
"HP:0000218",
"HP:0000268",
"HP:0000411",
"HP:0000508",
"HP:0000637",
"HP:0000639",
"HP:0001263",
"HP:0001270",
"HP:0001319",
"HP:0001324",
"HP:0001525",
"HP:0001611",
"HP:0002591",
"HP:0002650",
"HP:0003391",
"HP:0010804",
"HP:0030319"
],
[
"OMIM:616224"
]
],
[
[
"HP:0000119",
"HP:0000621",
"HP:0000973",
"HP:0001290",
"HP:0001371",
"HP:0001385",
"HP:0001519",
"HP:0001627",
"HP:0001999",
"HP:0002107",
"HP:0002617",
"HP:0002751",
"HP:0004942"
],
[
"OMIM:617402"
]
],
[
[

```
      "HP:0001888",
      "HP:0002090",
      "HP:0002720",
      "HP:0030273",
      "HP:0031381",
      "HP:0032218"
    ],
    [
      "OMIM:102700",
      "ORPHA:277"
    ]
  ],
  [
    [
      "HP:0000316",
      "HP:0000347",
      "HP:0000369",
      "HP:0000388",
      "HP:0000518",
      "HP:0000520",
      "HP:0000555",
      "HP:0000586",
      "HP:0000750",
      "HP:0001382",
      "HP:0001634",
      "HP:0002684",
      "HP:0002829",
      "HP:0003311",
      "HP:0004322",
      "HP:0006376",
      "HP:0008829",
      "HP:0011800",
      "HP:0410031"
    ],
    [
      "OMIM:108300",
      "ORPHA:90653"
    ]
  ],
  [
    [
      "HP:0001880",
      "HP:0001888",
      "HP:0003212",
      "HP:0003237",
      "HP:0008404",
      "HP:0009098",
      "HP:0031392",
      "HP:0031393"
    ],
    [
      "OMIM:212050",
      "ORPHA:457088"
    ]
  ],
  [
```

```
      [
        "HP:0000098",
        "HP:0001166",
        "HP:0002751",
        "HP:0009901",
        "HP:0012372",
        "HP:0012385"
      ],
      [
        "OMIM:121050",
        "ORPHA:115"
      ]
    ],
    [
      [
        "HP:0000083",
        "HP:0000093",
        "HP:0000510",
        "HP:0000678",
        "HP:0000819",
        "HP:0001162",
        "HP:0001328",
        "HP:0001513",
        "HP:0100259"
      ],
      [
        "OMIM:615981"
      ]
    ],
    [
      [
        "HP:0001519",
        "HP:0002650",
        "HP:0004970",
        "HP:0011003"
      ],
      [
        "OMIM:154700",
        "ORPHA:284963"
      ]
    ],
    [
      [
        "HP:0001510",
        "HP:0001518",
        "HP:0001561",
        "HP:0003270",
        "HP:0005208",
        "HP:0012604",
        "HP:0032368",
        "HP:0032484",
        "HP:0032487"
      ],
      [
        "OMIM:616868"
      ]
```

],
[
[
"HP:0000065",
"HP:0000105",
"HP:0000138",
"HP:0000388",
"HP:0000842",
"HP:0001263",
"HP:0001712",
"HP:0001943",
"HP:0002035",
"HP:0002090",
"HP:0002719",
"HP:0003270",
"HP:0008665",
"HP:0030796"
],
[
"OMIM:246200",
"ORPHA:508"
]
],
[
[
"HP:0001249",
"HP:0001250",
"HP:0001263",
"HP:0001290",
"HP:0001508",
"HP:0001942",
"HP:0001943",
"HP:0001946",
"HP:0001987",
"HP:0002013",
"HP:0002098",
"HP:0002151",
"HP:0003201",
"HP:0003648",
"HP:0011675",
"HP:0040145",
"HP:0045045"
],
[
"OMIM:616878",
"ORPHA:480864"
]
],
[
[
"HP:0000307",
"HP:0000674",
"HP:0000966",
"HP:0001006",
"HP:0001954",
"HP:0005280",

"HP:0009931",
"HP:0011220",
"HP:0012471"
],
[
"OMIM:305100",
"ORPHA:181"
]
],
[
[
"HP:0000119",
"HP:0000238",
"HP:0001510",
"HP:0001876",
"HP:0002721",
"HP:0005528",
"HP:0100702"
],
[
"OMIM:159550",
"ORPHA:2585"
]
],
[
[
"HP:0000175",
"HP:0000316",
"HP:0000325",
"HP:0000337",
"HP:0000486",
"HP:0000520",
"HP:0000527",
"HP:0000729",
"HP:0000735",
"HP:0000817",
"HP:0000998",
"HP:0001212",
"HP:0001250",
"HP:0001252",
"HP:0001290",
"HP:0001344",
"HP:0001388",
"HP:0001572",
"HP:0002019",
"HP:0002360",
"HP:0002719",
"HP:0004780",
"HP:0008763",
"HP:0011081",
"HP:0011344",
"HP:0100716"
],
[
"OMIM:618089"
]

```
    ],
    [
      [
        "HP:0000194",
        "HP:0000303",
        "HP:0000337",
        "HP:0000365",
        "HP:0000414",
        "HP:0000708",
        "HP:0001290",
        "HP:0001344",
        "HP:0001999",
        "HP:0002376",
        "HP:0002750",
        "HP:0011344",
        "HP:0012471"
      ],
      [
        "OMIM:616900",
        "ORPHA:488632"
      ]
    ],
    [
      [
        "HP:0001279",
        "HP:0001962",
        "HP:0012251"
      ],
      [
        "CCRD:14.3",
        "OMIM:601144",
        "ORPHA:130"
      ]
    ],
    [
      [
        "HP:0000179",
        "HP:0000215",
        "HP:0000252",
        "HP:0000293",
        "HP:0000309",
        "HP:0000457",
        "HP:0000470",
        "HP:0000750",
        "HP:0000960",
        "HP:0001263",
        "HP:0001270",
        "HP:0003508",
        "HP:0007441",
        "HP:0008676",
        "HP:0010230",
        "HP:0011968"
      ],
      [
        "OMIM:605130",
        "ORPHA:319182"
```

```
      ]
    ],
    [
      [
        "HP:0001019",
        "HP:0001263",
        "HP:0001347",
        "HP:0002179",
        "HP:0002197",
        "HP:0004322",
        "HP:0008873",
        "HP:0025116",
        "HP:0031430",
        "HP:0100806"
      ],
      [
        "OMIM:617425"
      ]
    ],
    [
      [
        "HP:0000501",
        "HP:0000518",
        "HP:0001249",
        "HP:0001252",
        "HP:0003076",
        "HP:0003126",
        "HP:0003355",
        "HP:0012592"
      ],
      [
        "OMIM:309000",
        "ORPHA:534"
      ]
    ],
    [
      [
        "HP:0000098",
        "HP:0000193",
        "HP:0000268",
        "HP:0000272",
        "HP:0000316",
        "HP:0000494",
        "HP:0000767",
        "HP:0001065",
        "HP:0001166",
        "HP:0001634",
        "HP:0002616",
        "HP:0002650",
        "HP:0003179",
        "HP:0004938",
        "HP:0010646",
        "HP:0011003",
        "HP:0012180",
        "HP:0100775"
      ],
```

```
    [
      "OMIM:609192",
      "ORPHA:60030"
    ]
  ],
  [
    [
      "HP:0001156",
      "HP:0002970",
      "HP:0004060",
      "HP:0004379",
      "HP:0008450",
      "HP:0030352",
      "HP:0031508",
      "HP:0100864",
      "HP:0100866"
    ],
    [
      "OMIM:146000",
      "ORPHA:429"
    ]
  ],
  [
    [
      "HP:0000726",
      "HP:0000738",
      "HP:0000746",
      "HP:0001260",
      "HP:0001288",
      "HP:0001300",
      "HP:0001332",
      "HP:0001337",
      "HP:0002063",
      "HP:0031908",
      "HP:0100660"
    ],
    [
      "OMIM:616840"
    ]
  ],
  [
    [
      "HP:0000219",
      "HP:0000252",
      "HP:0000294",
      "HP:0000343",
      "HP:0000664",
      "HP:0000750",
      "HP:0002342",
      "HP:0005280",
      "HP:0007665",
      "HP:0008897"
    ],
    [
      "OMIM:122470",
      "ORPHA:199"
```

```
    ]
  ],
  [
    [
      "HP:0000175",
      "HP:0000269",
      "HP:0000567",
      "HP:0001531",
      "HP:0001944",
      "HP:0002013",
      "HP:0002014",
      "HP:0002902",
      "HP:0004325",
      "HP:0004467",
      "HP:0011106",
      "HP:0011470",
      "HP:0410030"
    ],
    [
      "OMIM:270420"
    ]
  ],
  [
    [
      "HP:0000639",
      "HP:0001010",
      "HP:0005599"
    ],
    [
      "OMIM:203290",
      "ORPHA:79433"
    ]
  ],
  [
    [
      "HP:0001251",
      "HP:0001260",
      "HP:0001272",
      "HP:0001310",
      "HP:0003487"
    ],
    [
      "OMIM:159550",
      "ORPHA:2585"
    ]
  ],
  [
    [
      "HP:0000662",
      "HP:0001133",
      "HP:0007737",
      "HP:0008043"
    ],
    [
      "OMIM:601414"
    ]
```

```
  ],
  [
    [
      "HP:0000010",
      "HP:0000077",
      "HP:0000286",
      "HP:0000294",
      "HP:0000316",
      "HP:0000341",
      "HP:0000453",
      "HP:0000476",
      "HP:0000664",
      "HP:0001252",
      "HP:0001257",
      "HP:0001270",
      "HP:0001276",
      "HP:0001290",
      "HP:0001627",
      "HP:0002194",
      "HP:0002650",
      "HP:0005989",
      "HP:0008589",
      "HP:0012372",
      "HP:0012443",
      "HP:0030680"
    ],
    [
      "OMIM:616975"
    ]
  ],
  [
    [
      "HP:0000508",
      "HP:0000592",
      "HP:0000703",
      "HP:0000926",
      "HP:0000938",
      "HP:0001249",
      "HP:0001270",
      "HP:0001510",
      "HP:0001788",
      "HP:0002757",
      "HP:0003023"
    ],
    [
      "OMIM:615220"
    ]
  ],
  [
    [
      "HP:0000011",
      "HP:0000158",
      "HP:0000212",
      "HP:0000280",
      "HP:0000341",
      "HP:0000426",
```

```
      "HP:0000463",
      "HP:0000508",
      "HP:0000518",
      "HP:0000939",
      "HP:0001284",
      "HP:0001297",
      "HP:0001320",
      "HP:0001338",
      "HP:0002093",
      "HP:0002155",
      "HP:0002263",
      "HP:0002376",
      "HP:0002553",
      "HP:0006829",
      "HP:0007359",
      "HP:0010841",
      "HP:0011968",
      "HP:0012697",
      "HP:0012736",
      "HP:0100277",
      "HP:0100309",
      "HP:0100704"
    ],
    [
      "OMIM:616900",
      "ORPHA:488632"
    ]
  ],
  [
    [
      "HP:0001257",
      "HP:0001260",
      "HP:0002066"
    ],
    [
      "OMIM:616907",
      "ORPHA:488594"
    ]
  ],
  [
    [
      "HP:0000275",
      "HP:0000448",
      "HP:0000689",
      "HP:0000708",
      "HP:0001249",
      "HP:0006346",
      "HP:0011092",
      "HP:0040080",
      "HP:0100018"
    ],
    [
      "OMIM:302350",
      "ORPHA:627"
    ]
  ],
```

[
  [
    "HP:0000175",
    "HP:0001222",
    "HP:0001374",
    "HP:0001762",
    "HP:0002948",
    "HP:0003042",
    "HP:0003883",
    "HP:0004322",
    "HP:0005191",
    "HP:0008438",
    "HP:0011800",
    "HP:0011849"
  ],
  [
    "OMIM:150250",
    "ORPHA:503"
  ]
],
[
  [
    "HP:0000028",
    "HP:0000047",
    "HP:0000076",
    "HP:0000089",
    "HP:0000098",
    "HP:0000107",
    "HP:0000126",
    "HP:0000160",
    "HP:0000164",
    "HP:0000187",
    "HP:0000218",
    "HP:0000221",
    "HP:0000252",
    "HP:0000256",
    "HP:0000286",
    "HP:0000316",
    "HP:0000319",
    "HP:0000325",
    "HP:0000347",
    "HP:0000349",
    "HP:0000365",
    "HP:0000377",
    "HP:0000395",
    "HP:0000407",
    "HP:0000414",
    "HP:0000453",
    "HP:0000463",
    "HP:0000490",
    "HP:0000494",
    "HP:0000525",
    "HP:0000534",
    "HP:0000568",
    "HP:0000581",
    "HP:0000582",

"HP:0000589",
"HP:0000610",
"HP:0000648",
"HP:0000659",
"HP:0000708",
"HP:0000752",
"HP:0000957",
"HP:0001172",
"HP:0001252",
"HP:0001263",
"HP:0001285",
"HP:0001290",
"HP:0001320",
"HP:0001385",
"HP:0001510",
"HP:0001510",
"HP:0001511",
"HP:0001561",
"HP:0001629",
"HP:0001643",
"HP:0001655",
"HP:0001671",
"HP:0001734",
"HP:0001792",
"HP:0001999",
"HP:0002007",
"HP:0002020",
"HP:0002021",
"HP:0002079",
"HP:0002119",
"HP:0002121",
"HP:0002247",
"HP:0002273",
"HP:0002557",
"HP:0002650",
"HP:0002896",
"HP:0002938",
"HP:0003186",
"HP:0003307",
"HP:0003429",
"HP:0004209",
"HP:0004322",
"HP:0004467",
"HP:0006097",
"HP:0006808",
"HP:0007018",
"HP:0007361",
"HP:0007376",
"HP:0008897",
"HP:0009908",
"HP:0010322",
"HP:0010704",
"HP:0010772",
"HP:0011259",
"HP:0011968",
"HP:0012448",

```
      "HP:0012471",
      "HP:0025100",
      "HP:0030301",
      "HP:0045025",
      "HP:0200055",
      "HP:0410030"
    ],
    [
      "OMIM:616975"
    ]
  ],
  [
    [
      "HP:0001156",
      "HP:0001387",
      "HP:0001642",
      "HP:0002650",
      "HP:0002938",
      "HP:0004322",
      "HP:0011003",
      "HP:0011623",
      "HP:0030961"
    ],
    [
      "OMIM:277600"
    ]
  ],
  [
    [
      "HP:0000047",
      "HP:0000076",
      "HP:0000083",
      "HP:0000286",
      "HP:0000294",
      "HP:0000369",
      "HP:0000384",
      "HP:0000470",
      "HP:0000527",
      "HP:0000718",
      "HP:0000750",
      "HP:0000752",
      "HP:0001263",
      "HP:0001631",
      "HP:0001845",
      "HP:0002023",
      "HP:0002353",
      "HP:0004322",
      "HP:0007018",
      "HP:0008551",
      "HP:0009944",
      "HP:0012443",
      "HP:0100258"
    ],
    [
      "OMIM:107480"
    ]
```

```
  ],
  [
    [
      "HP:0000175",
      "HP:0000218",
      "HP:0000238",
      "HP:0000347",
      "HP:0000369",
      "HP:0000411",
      "HP:0000437",
      "HP:0000637",
      "HP:0001290",
      "HP:0001385",
      "HP:0001392",
      "HP:0001510",
      "HP:0001612",
      "HP:0001623",
      "HP:0001626",
      "HP:0001743",
      "HP:0006956",
      "HP:0007655",
      "HP:0008897",
      "HP:0031508",
      "HP:0045075"
    ],
    [
      "OMIM:300867"
    ]
  ],
  [
    [
      "HP:0000212",
      "HP:0000218",
      "HP:0000276",
      "HP:0000365",
      "HP:0000486",
      "HP:0000666",
      "HP:0000678",
      "HP:0001238",
      "HP:0001263",
      "HP:0001285",
      "HP:0001290",
      "HP:0001321",
      "HP:0001332",
      "HP:0001347",
      "HP:0001548",
      "HP:0002079",
      "HP:0002415",
      "HP:0002500",
      "HP:0002650",
      "HP:0002828",
      "HP:0003429",
      "HP:0006808",
      "HP:0007034",
      "HP:0012444"
    ],
```

```
    [
      "OMIM:617560",
      "ORPHA:527497"
    ]
  ],
  [
    [
      "HP:0000110",
      "HP:0000252",
      "HP:0000821",
      "HP:0000961",
      "HP:0000976",
      "HP:0001336",
      "HP:0001999",
      "HP:0002104",
      "HP:0002721",
      "HP:0002878",
      "HP:0002925",
      "HP:0003121",
      "HP:0005352",
      "HP:0005484",
      "HP:0005943",
      "HP:0008936",
      "HP:0010881",
      "HP:0011123",
      "HP:0012211",
      "HP:0031430"
    ],
    [
      "OMIM:617425"
    ]
  ],
  [
    [
      "HP:0001878",
      "HP:0004444",
      "HP:0005502"
    ],
    [
      "OMIM:612653"
    ]
  ],
  [
    [
      "HP:0000962",
      "HP:0001010",
      "HP:0001510",
      "HP:0002091",
      "HP:0002110",
      "HP:0002664",
      "HP:0002721",
      "HP:0002745",
      "HP:0007898",
      "HP:0008404",
      "HP:0031631",
      "HP:0100792"
```

```
    ],
    [
      "OMIM:613990"
    ]
  ],
  [
    [
      "HP:0000729",
      "HP:0000750",
      "HP:0001250",
      "HP:0001263",
      "HP:0002018",
      "HP:0002019",
      "HP:0025051"
    ],
    [
      "OMIM:300352",
      "ORPHA:52503"
    ]
  ],
  [
    [
      "HP:0000275",
      "HP:0000347",
      "HP:0000508",
      "HP:0000651",
      "HP:0001260",
      "HP:0001265",
      "HP:0002495",
      "HP:0002515",
      "HP:0002527",
      "HP:0002650",
      "HP:0002705",
      "HP:0002913",
      "HP:0003202",
      "HP:0003326",
      "HP:0003391",
      "HP:0003691",
      "HP:0003701",
      "HP:0009046",
      "HP:0010830",
      "HP:0040129"
    ],
    [
      "OMIM:609284"
    ]
  ],
  [
    [
      "HP:0001258",
      "HP:0001260",
      "HP:0002066",
      "HP:0002070"
    ],
    [
      "OMIM:616907",
```

```
      "ORPHA:488594"
    ]
  ],
  [
    [
      "HP:0000003",
      "HP:0000028",
      "HP:0000047",
      "HP:0000076",
      "HP:0000098",
      "HP:0000160",
      "HP:0000164",
      "HP:0000187",
      "HP:0000218",
      "HP:0000221",
      "HP:0000252",
      "HP:0000256",
      "HP:0000286",
      "HP:0000316",
      "HP:0000319",
      "HP:0000325",
      "HP:0000347",
      "HP:0000349",
      "HP:0000365",
      "HP:0000377",
      "HP:0000407",
      "HP:0000414",
      "HP:0000453",
      "HP:0000463",
      "HP:0000490",
      "HP:0000494",
      "HP:0000525",
      "HP:0000534",
      "HP:0000568",
      "HP:0000581",
      "HP:0000582",
      "HP:0000601",
      "HP:0000612",
      "HP:0000648",
      "HP:0000659",
      "HP:0000708",
      "HP:0000752",
      "HP:0000957",
      "HP:0000998",
      "HP:0001159",
      "HP:0001250",
      "HP:0001252",
      "HP:0001263",
      "HP:0001285",
      "HP:0001290",
      "HP:0001317",
      "HP:0001385",
      "HP:0001511",
      "HP:0001629",
      "HP:0001643",
      "HP:0001655",
```

```
      "HP:0001734",
      "HP:0001792",
      "HP:0002007",
      "HP:0002020",
      "HP:0002021",
      "HP:0002079",
      "HP:0002119",
      "HP:0002247",
      "HP:0002273",
      "HP:0002334",
      "HP:0002557",
      "HP:0002650",
      "HP:0002938",
      "HP:0003186",
      "HP:0003307",
      "HP:0004209",
      "HP:0004467",
      "HP:0004532",
      "HP:0006808",
      "HP:0007018",
      "HP:0007361",
      "HP:0008897",
      "HP:0010490",
      "HP:0010772",
      "HP:0011328",
      "HP:0011968",
      "HP:0012448",
      "HP:0012471",
      "HP:0025100",
      "HP:0030301",
      "HP:0045025",
      "HP:0200055",
      "HP:0410030"
    ],
    [
      "OMIM:616975"
    ]
  ],
  [
    [
      "HP:0001873",
      "HP:0001892",
      "HP:0005513",
      "HP:0011974",
      "HP:0012528"
    ],
    [
      "OMIM:187900"
    ]
  ],
  [
    [
      "HP:0001256",
      "HP:0002035",
      "HP:0003183",
      "HP:0003796",
```

```
      "HP:0008905",
      "HP:0010049",
      "HP:0010743"
    ],
    [
      "OMIM:223800",
      "ORPHA:239"
    ]
  ],
  [
    [
      "HP:0000175",
      "HP:0000316",
      "HP:0001623",
      "HP:0002650",
      "HP:0002827",
      "HP:0002947",
      "HP:0002987",
      "HP:0004976",
      "HP:0005280",
      "HP:0010049",
      "HP:0010301",
      "HP:0011432"
    ],
    [
      "OMIM:150250",
      "ORPHA:503"
    ]
  ],
  [
    [
      "HP:0000253",
      "HP:0000322",
      "HP:0000486",
      "HP:0000574",
      "HP:0000580",
      "HP:0001182",
      "HP:0001263",
      "HP:0001290",
      "HP:0001317",
      "HP:0001382",
      "HP:0001510",
      "HP:0001956",
      "HP:0002033",
      "HP:0002363",
      "HP:0004322",
      "HP:0007074",
      "HP:0012471",
      "HP:0100874"
    ],
    [
      "OMIM:216550",
      "ORPHA:193"
    ]
  ],
  [
```

```
    [
      "HP:0000518",
      "HP:0001263",
      "HP:0001266",
      "HP:0001270",
      "HP:0001290",
      "HP:0002023",
      "HP:0002059",
      "HP:0002061",
      "HP:0003112",
      "HP:0003542",
      "HP:0004322",
      "HP:0010818",
      "HP:0040081"
    ],
    [
      "OMIM:600373",
      "ORPHA:1458"
    ]
  ],
  [
    [
      "HP:0000729",
      "HP:0001249",
      "HP:0001250",
      "HP:0001328",
      "HP:0009717",
      "HP:0009719",
      "HP:0010615"
    ],
    [
      "OMIM:191100"
    ]
  ],
  [
    [
      "HP:0000218",
      "HP:0000219",
      "HP:0000276",
      "HP:0000319",
      "HP:0000337",
      "HP:0000400",
      "HP:0000490",
      "HP:0000508",
      "HP:0000601",
      "HP:0001511",
      "HP:0001636",
      "HP:0002020",
      "HP:0011968",
      "HP:0012758"
    ],
    [
      "OMIM:617452",
      "ORPHA:505237"
    ]
  ],
```

```
[
  [
    "HP:0000077",
    "HP:0000218",
    "HP:0000365",
    "HP:0000407",
    "HP:0000453",
    "HP:0000478",
    "HP:0000490",
    "HP:0000508",
    "HP:0000565",
    "HP:0000722",
    "HP:0000733",
    "HP:0000750",
    "HP:0000961",
    "HP:0001222",
    "HP:0001249",
    "HP:0001251",
    "HP:0001252",
    "HP:0001263",
    "HP:0001290",
    "HP:0001488",
    "HP:0001631",
    "HP:0001671",
    "HP:0001795",
    "HP:0002058",
    "HP:0002079",
    "HP:0002104",
    "HP:0002363",
    "HP:0002463",
    "HP:0002474",
    "HP:0002616",
    "HP:0002650",
    "HP:0003198",
    "HP:0010535",
    "HP:0012372",
    "HP:0030842"
  ],
  [
    "OMIM:616975"
  ]
],
[
  [
    "HP:0000248",
    "HP:0000365",
    "HP:0000403",
    "HP:0000486",
    "HP:0000750",
    "HP:0001249",
    "HP:0001263",
    "HP:0001270",
    "HP:0001290",
    "HP:0001956",
    "HP:0002360",
    "HP:0002650",
```

```
        "HP:0004322",
        "HP:0011800",
        "HP:0100716"
      ],
      [
        "OMIM:182290",
        "ORPHA:819"
      ]
    ],
    [
      [
        "HP:0000473",
        "HP:0001270",
        "HP:0001302",
        "HP:0001336",
        "HP:0001339",
        "HP:0001347",
        "HP:0001385",
        "HP:0002069",
        "HP:0002174",
        "HP:0002857",
        "HP:0003307",
        "HP:0003487",
        "HP:0003808",
        "HP:0006956",
        "HP:0007018",
        "HP:0007033",
        "HP:0100021",
        "HP:0100543"
      ],
      [
        "OMIM:610031"
      ]
    ],
    [
      [
        "HP:0001156",
        "HP:0001216",
        "HP:0001999",
        "HP:0002515",
        "HP:0002834",
        "HP:0002970",
        "HP:0003025",
        "HP:0003026",
        "HP:0003090",
        "HP:0003180",
        "HP:0004322",
        "HP:0004568",
        "HP:0008155",
        "HP:0008833",
        "HP:0008873",
        "HP:0010582",
        "HP:0012514"
      ],
      [
        "OMIM:177170",
```

```
      "ORPHA:750"
    ]
  ],
  [
    [
      "HP:0000639",
      "HP:0001876",
      "HP:0005528"
    ],
    [
      "OMIM:159550",
      "ORPHA:2585"
    ]
  ],
  [
    [
      "HP:0001279",
      "HP:0001638",
      "HP:0001663",
      "HP:0001695",
      "HP:0011712",
      "HP:0012819"
    ],
    [
      "CCRD:14.4",
      "OMIM:604772",
      "ORPHA:3286"
    ]
  ],
  [
    [
      "HP:0000737",
      "HP:0001276",
      "HP:0001290",
      "HP:0001347",
      "HP:0001945",
      "HP:0006532"
    ],
    [
      "OMIM:245200",
      "ORPHA:487"
    ]
  ],
  [
    [
      "HP:0000605",
      "HP:0001260",
      "HP:0001265",
      "HP:0002305"
    ],
    [
      "OMIM:208920",
      "ORPHA:1168"
    ]
  ],
  [
```

```
    [
      "HP:0000407",
      "HP:0000848",
      "HP:0001252",
      "HP:0001561",
      "HP:0002149",
      "HP:0002150",
      "HP:0002902",
      "HP:0003113",
      "HP:0200114"
    ],
    [
      "OMIM:602522"
    ]
  ],
  [
    [
      "HP:0000158",
      "HP:0000193",
      "HP:0001363",
      "HP:0001655",
      "HP:0001888",
      "HP:0001943",
      "HP:0001999",
      "HP:0002205",
      "HP:0002652",
      "HP:0002904",
      "HP:0008454",
      "HP:0011569"
    ],
    [
      "OMIM:617425"
    ]
  ],
  [
    [
      "HP:0000967",
      "HP:0001873",
      "HP:0005537",
      "HP:0005548"
    ],
    [
      "OMIM:273900"
    ]
  ],
  [
    [
      "HP:0000212",
      "HP:0000639",
      "HP:0001332",
      "HP:0002878",
      "HP:0006855",
      "HP:0007015",
      "HP:0007663",
      "HP:0008947",
      "HP:0100543"
```

```
    ],
    [
      "OMIM:617560",
      "ORPHA:527497"
    ]
  ],
  [
    [
      "HP:0001433",
      "HP:0002180",
      "HP:0002354",
      "HP:0002361",
      "HP:0002448",
      "HP:0002527",
      "HP:0002529",
      "HP:0007009",
      "HP:0007017",
      "HP:0007064",
      "HP:0007086",
      "HP:0007164",
      "HP:0007272",
      "HP:0007369"
    ],
    [
      "CCRD:82.3",
      "OMIM:257220",
      "ORPHA:646"
    ]
  ],
  [
    [
      "HP:0000252",
      "HP:0000407",
      "HP:0000490",
      "HP:0000670",
      "HP:0000992",
      "HP:0001290",
      "HP:0004322"
    ],
    [
      "OMIM:216400"
    ]
  ],
  [
    [
      "HP:0000340",
      "HP:0000414",
      "HP:0001249",
      "HP:0001250",
      "HP:0002283",
      "HP:0006829",
      "HP:0010804",
      "HP:0011344"
    ],
    [
      "OMIM:616900",
```

```
      "ORPHA:488632"
    ]
  ],
  [
    [
      "HP:0000011",
      "HP:0000158",
      "HP:0000256",
      "HP:0000280",
      "HP:0000574",
      "HP:0000836",
      "HP:0001249",
      "HP:0001250",
      "HP:0001315",
      "HP:0001344",
      "HP:0002376",
      "HP:0002650",
      "HP:0006829",
      "HP:0011344"
    ],
    [
      "OMIM:616900",
      "ORPHA:488632"
    ]
  ],
  [
    [
      "HP:0000301",
      "HP:0001283",
      "HP:0001371",
      "HP:0001388",
      "HP:0002505",
      "HP:0003236",
      "HP:0003691",
      "HP:0003720",
      "HP:0003749",
      "HP:0006520",
      "HP:0008994",
      "HP:0032178",
      "HP:0100851"
    ],
    [
      "OMIM:617232",
      "ORPHA:480682"
    ]
  ],
  [
    [
      "HP:0000175",
      "HP:0000238",
      "HP:0000252",
      "HP:0000341",
      "HP:0000347",
      "HP:0000358",
      "HP:0000369",
      "HP:0000403",
```

```
      "HP:0000426",
      "HP:0000470",
      "HP:0000475",
      "HP:0000494",
      "HP:0000527",
      "HP:0000577",
      "HP:0000750",
      "HP:0000767",
      "HP:0000914",
      "HP:0001181",
      "HP:0001263",
      "HP:0001510",
      "HP:0001558",
      "HP:0001762",
      "HP:0001845",
      "HP:0002007",
      "HP:0002019",
      "HP:0002099",
      "HP:0002208",
      "HP:0002307",
      "HP:0003487",
      "HP:0005487",
      "HP:0006610",
      "HP:0007665",
      "HP:0031910",
      "HP:0100807"
    ],
    [
      "OMIM:602398",
      "ORPHA:35107"
    ]
  ],
  [
    [
      "HP:0000325",
      "HP:0000957",
      "HP:0001371",
      "HP:0001511",
      "HP:0001629",
      "HP:0004322",
      "HP:0005528"
    ],
    [
      "OMIM:227645"
    ]
  ],
  [
    [
      "HP:0000256",
      "HP:0000750",
      "HP:0001249",
      "HP:0001250",
      "HP:0001252",
      "HP:0001315",
      "HP:0001999",
      "HP:0002119",
```

```
      "HP:0002376",
      "HP:0011344"
    ],
    [
      "OMIM:616900",
      "ORPHA:488632"
    ]
  ],
  [
    [
      "HP:0000194",
      "HP:0000248",
      "HP:0000340",
      "HP:0000343",
      "HP:0000490",
      "HP:0000586",
      "HP:0000817",
      "HP:0001270",
      "HP:0001272",
      "HP:0001284",
      "HP:0001331",
      "HP:0001344",
      "HP:0001347",
      "HP:0001935",
      "HP:0002069",
      "HP:0002079",
      "HP:0002119",
      "HP:0002133",
      "HP:0002280",
      "HP:0002283",
      "HP:0002375",
      "HP:0002878",
      "HP:0005667",
      "HP:0006829",
      "HP:0007305",
      "HP:0010804",
      "HP:0011344",
      "HP:0012032",
      "HP:0012444",
      "HP:0100288"
    ],
    [
      "OMIM:616900",
      "ORPHA:488632"
    ]
  ],
  [
    [
      "HP:0000473",
      "HP:0000511",
      "HP:0000571",
      "HP:0000605",
      "HP:0000639",
      "HP:0001251",
      "HP:0001260",
      "HP:0001270",
```

```
      "HP:0001272",
      "HP:0001332",
      "HP:0002062",
      "HP:0002497",
      "HP:0002540",
      "HP:0003429",
      "HP:0003474",
      "HP:0007108",
      "HP:0009830",
      "HP:0012547",
      "HP:0030187"
    ],
    [
      "OMIM:617560",
      "ORPHA:527497"
    ]
  ],
  [
    [
      "HP:0000239",
      "HP:0000242",
      "HP:0000316",
      "HP:0000347",
      "HP:0000680",
      "HP:0000894",
      "HP:0002007",
      "HP:0002645",
      "HP:0002650",
      "HP:0002866",
      "HP:0004322",
      "HP:0005259",
      "HP:0005280",
      "HP:0006297",
      "HP:0008788",
      "HP:0008804",
      "HP:0011223",
      "HP:0011800",
      "HP:0100864"
    ],
    [
      "OMIM:119600",
      "ORPHA:1452"
    ]
  ],
  [
    [
      "HP:0000252",
      "HP:0000485",
      "HP:0000543",
      "HP:0000639",
      "HP:0001263",
      "HP:0001272",
      "HP:0002079",
      "HP:0030890"
    ],
    [
```

```
      "OMIM:252650",
      "ORPHA:578"
    ]
  ],
  [
    [
      "HP:0001522",
      "HP:0002013",
      "HP:0040319"
    ],
    [
      "OMIM:203500",
      "ORPHA:56"
    ]
  ],
  [
    [
      "HP:0000518",
      "HP:0000750",
      "HP:0001081",
      "HP:0001249",
      "HP:0001257",
      "HP:0001508",
      "HP:0001522",
      "HP:0001760",
      "HP:0002013",
      "HP:0002240",
      "HP:0002910",
      "HP:0003155",
      "HP:0003811",
      "HP:0003819",
      "HP:0004342",
      "HP:0010899",
      "HP:0010916",
      "HP:0011421",
      "HP:0011968",
      "HP:0012024"
    ],
    [
      "CCRD:30",
      "OMIM:230400",
      "ORPHA:352",
      "ORPHA:79239"
    ]
  ],
  [
    [
      "HP:0000752",
      "HP:0001254",
      "HP:0001259",
      "HP:0001298",
      "HP:0001317",
      "HP:0001332",
      "HP:0001649",
      "HP:0001919",
      "HP:0001945",
```

```
      "HP:0001974",
      "HP:0001987",
      "HP:0002060",
      "HP:0002071",
      "HP:0002153",
      "HP:0002329",
      "HP:0002360",
      "HP:0002910",
      "HP:0003228",
      "HP:0003819",
      "HP:0012378",
      "HP:0012622",
      "HP:0100660"
    ],
    [
      "CCRD:18",
      "OMIM:215700",
      "ORPHA:187",
      "ORPHA:247525"
    ]
  ],
  [
    [
      "HP:0001265",
      "HP:0001290",
      "HP:0001397",
      "HP:0001522",
      "HP:0001649",
      "HP:0001944",
      "HP:0001945",
      "HP:0002013",
      "HP:0002027",
      "HP:0002094",
      "HP:0002151",
      "HP:0002155",
      "HP:0002240",
      "HP:0002789",
      "HP:0003202",
      "HP:0003348",
      "HP:0003355",
      "HP:0003811",
      "HP:0009128",
      "HP:0011968",
      "HP:0012447",
      "HP:0031500"
    ],
    [
      "CCRD:40",
      "OMIM:229600",
      "ORPHA:469"
    ]
  ],
  [
    [
      "HP:0001250",
      "HP:0001270",
```

```
      "HP:0001522",
      "HP:0010899"
    ],
    [
      "OMIM:271900",
      "ORPHA:141"
    ]
  ],
  [
    [
      "HP:0001051",
      "HP:0001250",
      "HP:0001290",
      "HP:0001522",
      "HP:0001942",
      "HP:0002151",
      "HP:0040156"
    ],
    [
      "CCRD:13",
      "OMIM:253260",
      "ORPHA:79241"
    ]
  ],
  [
    [
      "HP:0001197",
      "HP:0001249",
      "HP:0001522",
      "HP:0003231",
      "HP:0003811",
      "HP:0004923",
      "HP:0011421"
    ],
    [
      "CCRD:90",
      "OMIM:261600",
      "ORPHA:716"
    ]
  ],
  [
    [
      "HP:0001522",
      "HP:0001629",
      "HP:0001638",
      "HP:0001974",
      "HP:0002013",
      "HP:0002098",
      "HP:0003075",
      "HP:0010472",
      "HP:0010899",
      "HP:0011968",
      "HP:0025435"
    ],
    [
      "OMIM:261750",
```

```
      "ORPHA:79240"
    ]
  ],
  [
    [
      "HP:0000252",
      "HP:0000486",
      "HP:0000821",
      "HP:0001249",
      "HP:0001250",
      "HP:0001270",
      "HP:0001522",
      "HP:0002019",
      "HP:0002925",
      "HP:0003231",
      "HP:0003819",
      "HP:0004923"
    ],
    [
      "CCRD:90",
      "OMIM:261600",
      "ORPHA:716"
    ]
  ],
  [
    [
      "HP:0000158",
      "HP:0000431",
      "HP:0001249",
      "HP:0001251",
      "HP:0001270",
      "HP:0001284",
      "HP:0001290",
      "HP:0001522",
      "HP:0001760",
      "HP:0002090",
      "HP:0002355",
      "HP:0002751",
      "HP:0003202",
      "HP:0003270",
      "HP:0003551",
      "HP:0003819",
      "HP:0008064",
      "HP:0009046",
      "HP:0009128",
      "HP:0010571",
      "HP:0011421",
      "HP:0012714"
    ],
    [
      "OMIM:266500",
      "ORPHA:773"
    ]
  ],
  [
    [
```

"HP:0001250",
"HP:0001270",
"HP:0001290",
"HP:0001522",
"HP:0003215"
],
[
"OMIM:220110",
"ORPHA:254905"
]
],
[
[
"HP:0001197",
"HP:0001522",
"HP:0003231",
"HP:0003811",
"HP:0004923"
],
[
"CCRD:90",
"OMIM:261600",
"ORPHA:716"
]
],
[
[
"HP:0001249",
"HP:0001259",
"HP:0001270",
"HP:0001290",
"HP:0001332",
"HP:0001522",
"HP:0001942",
"HP:0001943",
"HP:0001987",
"HP:0002098",
"HP:0003819",
"HP:0010895",
"HP:0011968",
"HP:0040156"
],
[
"CCRD:12",
"OMIM:203750",
"ORPHA:134"
]
],
[
[
"HP:0000750",
"HP:0001265",
"HP:0001290",
"HP:0003819",
"HP:0004353",
"HP:0012127"

```
    ],
    [
      "OMIM:222748",
      "ORPHA:38874"
    ]
  ],
  [
    [
      "HP:0001385",
      "HP:0001522",
      "HP:0001622",
      "HP:0003231",
      "HP:0003510",
      "HP:0003811",
      "HP:0004923",
      "HP:0011421",
      "HP:0040156"
    ],
    [
      "CCRD:90",
      "OMIM:261600",
      "ORPHA:716"
    ]
  ],
  [
    [
      "HP:0000741",
      "HP:0001290",
      "HP:0001522",
      "HP:0001942",
      "HP:0001987",
      "HP:0002151",
      "HP:0002789",
      "HP:0003209",
      "HP:0003353",
      "HP:0003355",
      "HP:0008315",
      "HP:0010895",
      "HP:0040156"
    ],
    [
      "CCRD:44",
      "OMIM:253270",
      "ORPHA:79242"
    ]
  ],
  [
    [
      "HP:0001197",
      "HP:0001522",
      "HP:0003811",
      "HP:0003819",
      "HP:0010967",
      "HP:0100950"
    ],
    [
```

"CCRD:70",
"OMIM:201450",
"ORPHA:42"
]
],
[
[
"HP:0000252",
"HP:0000648",
"HP:0000750",
"HP:0001249",
"HP:0001250",
"HP:0001251",
"HP:0001264",
"HP:0001907",
"HP:0002160",
"HP:0002510",
"HP:0003658",
"HP:0003819",
"HP:0011421"
],
[
"OMIM:236250",
"ORPHA:395"
]
],
[
[
"HP:0000486",
"HP:0000639",
"HP:0001251",
"HP:0001265",
"HP:0001270",
"HP:0001272",
"HP:0001290",
"HP:0001298",
"HP:0001510",
"HP:0001522",
"HP:0012642"
],
[
"OMIM:212065",
"ORPHA:79318"
]
],
[
[
"HP:0001051",
"HP:0001250",
"HP:0001265",
"HP:0001290",
"HP:0001522",
"HP:0003355",
"HP:0010895",
"HP:0040156",
"HP:0500001"

```
    ],
    [
      "CCRD:13",
      "OMIM:253260",
      "ORPHA:79241"
    ]
  ],
  [
    [
      "HP:0000750",
      "HP:0001197",
      "HP:0001522",
      "HP:0003811",
      "HP:0003819",
      "HP:0008315",
      "HP:0100950"
    ],
    [
      "CCRD:70",
      "OMIM:201450",
      "ORPHA:42"
    ]
  ],
  [
    [
      "HP:0000961",
      "HP:0000976",
      "HP:0000988",
      "HP:0001290",
      "HP:0001522",
      "HP:0001903",
      "HP:0001942",
      "HP:0001987",
      "HP:0002014",
      "HP:0002789",
      "HP:0003209",
      "HP:0003353",
      "HP:0003355",
      "HP:0003811",
      "HP:0010895",
      "HP:0010996",
      "HP:0040156"
    ],
    [
      "CCRD:44",
      "OMIM:253270",
      "ORPHA:79242"
    ]
  ],
  [
    [
      "HP:0000570",
      "HP:0000961",
      "HP:0001250",
      "HP:0001522",
      "HP:0002013",
```

```
      "HP:0002060",
      "HP:0002094",
      "HP:0003150",
      "HP:0011968"
    ],
    [
      "CCRD:34.1",
      "OMIM:231670",
      "ORPHA:25"
    ]
  ],
  [
    [
      "HP:0001197",
      "HP:0001522",
      "HP:0002013",
      "HP:0003811",
      "HP:0003819",
      "HP:0004386",
      "HP:0004429",
      "HP:0008315",
      "HP:0100950"
    ],
    [
      "CCRD:70",
      "OMIM:201450",
      "ORPHA:42"
    ]
  ],
  [
    [
      "HP:0001254",
      "HP:0001257",
      "HP:0001290",
      "HP:0001347",
      "HP:0001522",
      "HP:0001987",
      "HP:0002329",
      "HP:0002360",
      "HP:0003811",
      "HP:0011968"
    ],
    [
      "OMIM:237300",
      "ORPHA:147"
    ]
  ],
  [
    [
      "HP:0001051",
      "HP:0001197",
      "HP:0001522",
      "HP:0003231",
      "HP:0003811",
      "HP:0003819",
      "HP:0004923"
```

```
    ],
    [
      "CCRD:90",
      "OMIM:261600",
      "ORPHA:716"
    ]
  ],
  [
    [
      "HP:0000509",
      "HP:0000750",
      "HP:0001510",
      "HP:0001513",
      "HP:0001518",
      "HP:0001522",
      "HP:0001622",
      "HP:0001877",
      "HP:0003231",
      "HP:0003811",
      "HP:0003819",
      "HP:0004923",
      "HP:0010472",
      "HP:0012418"
    ],
    [
      "CCRD:90",
      "OMIM:261600",
      "ORPHA:716"
    ]
  ],
  [
    [
      "HP:0000252",
      "HP:0000486",
      "HP:0000639",
      "HP:0001249",
      "HP:0001251",
      "HP:0001265",
      "HP:0001270",
      "HP:0001272",
      "HP:0001290",
      "HP:0001298",
      "HP:0001510",
      "HP:0001522",
      "HP:0012642"
    ],
    [
      "OMIM:212065",
      "ORPHA:79318"
    ]
  ],
  [
    [
      "HP:0000486",
      "HP:0001249",
      "HP:0001251",
```

```
      "HP:0001270",
      "HP:0001272",
      "HP:0001513",
      "HP:0001522",
      "HP:0001907",
      "HP:0003819",
      "HP:0012642"
    ],
    [
      "OMIM:212065",
      "ORPHA:79318"
    ]
  ],
  [
    [
      "HP:0000962",
      "HP:0000976",
      "HP:0000988",
      "HP:0001197",
      "HP:0001522",
      "HP:0001642",
      "HP:0002239",
      "HP:0003231",
      "HP:0003811",
      "HP:0003819",
      "HP:0004386",
      "HP:0004923"
    ],
    [
      "CCRD:90",
      "OMIM:261600",
      "ORPHA:716"
    ]
  ],
  [
    [
      "HP:0000238",
      "HP:0001250",
      "HP:0001251",
      "HP:0001290",
      "HP:0001522",
      "HP:0003355",
      "HP:0003811",
      "HP:0010895",
      "HP:0040156"
    ],
    [
      "OMIM:210200",
      "ORPHA:6"
    ]
  ],
  [
    [
      "HP:0000776",
      "HP:0001270",
      "HP:0001272",
```

```
      "HP:0001508",
      "HP:0001522",
      "HP:0002036",
      "HP:0009125",
      "HP:0011804",
      "HP:0012642"
    ],
    [
      "OMIM:212065",
      "ORPHA:79318"
    ]
  ],
  [
    [
      "HP:0000486",
      "HP:0001265",
      "HP:0001290",
      "HP:0001513",
      "HP:0001522",
      "HP:0001945",
      "HP:0002059",
      "HP:0010899"
    ],
    [
      "OMIM:271900",
      "ORPHA:141"
    ]
  ],
  [
    [
      "HP:0001197",
      "HP:0001522",
      "HP:0003231",
      "HP:0003811",
      "HP:0004923"
    ],
    [
      "CCRD:90",
      "OMIM:261600",
      "ORPHA:716"
    ]
  ],
  [
    [
      "HP:0001197",
      "HP:0001522",
      "HP:0003811",
      "HP:0010967"
    ],
    [
      "CCRD:70",
      "OMIM:201450",
      "ORPHA:42"
    ]
  ],
  [
```

```
      [
        "HP:0003231",
        "HP:0004923",
        "HP:0100613"
      ],
      [
        "CCRD:90",
        "OMIM:261600",
        "ORPHA:716"
      ]
    ],
    [
      [
        "HP:0001197",
        "HP:0001522",
        "HP:0003231",
        "HP:0003811",
        "HP:0004923"
      ],
      [
        "CCRD:90",
        "OMIM:261600",
        "ORPHA:716"
      ]
    ],
    [
      [
        "HP:0000369",
        "HP:0000639",
        "HP:0001257",
        "HP:0001522",
        "HP:0002094",
        "HP:0002286",
        "HP:0002705",
        "HP:0003573",
        "HP:0003811",
        "HP:0010307",
        "HP:0011364",
        "HP:0011968",
        "HP:0012378"
      ],
      [
        "OMIM:266130",
        "ORPHA:289846"
      ]
    ],
    [
      [
        "HP:0001290",
        "HP:0001522",
        "HP:0010899"
      ],
      [
        "OMIM:271900",
        "ORPHA:141"
      ]
```

```
  ],
  [
    [
      "HP:0000238",
      "HP:0000256",
      "HP:0000975",
      "HP:0001250",
      "HP:0001254",
      "HP:0001332",
      "HP:0001347",
      "HP:0001522",
      "HP:0001903",
      "HP:0001945",
      "HP:0002013",
      "HP:0002059",
      "HP:0002179",
      "HP:0002329",
      "HP:0002360",
      "HP:0002380",
      "HP:0002781",
      "HP:0002922",
      "HP:0003150",
      "HP:0003819",
      "HP:0004429",
      "HP:0006541",
      "HP:0011421"
    ],
    [
      "CCRD:34.1",
      "OMIM:231670",
      "ORPHA:25"
    ]
  ],
  [
    [
      "HP:0000618",
      "HP:0001903",
      "HP:0001987",
      "HP:0002024",
      "HP:0002154",
      "HP:0002315",
      "HP:0003510",
      "HP:0003819",
      "HP:0005961",
      "HP:0011421",
      "HP:0011968",
      "HP:0012026",
      "HP:0012278",
      "HP:0100613"
    ],
    [
      "OMIM:258870",
      "ORPHA:414"
    ]
  ],
  [
```

```
    [
      "HP:0001522",
      "HP:0002093",
      "HP:0003811",
      "HP:0004353",
      "HP:0012127"
    ],
    [
      "OMIM:274270",
      "ORPHA:1675"
    ]
  ],
  [
    [
      "HP:0001197",
      "HP:0001522",
      "HP:0003231",
      "HP:0003811",
      "HP:0003819",
      "HP:0004923"
    ],
    [
      "OMIM:276710",
      "ORPHA:69723"
    ]
  ],
  [
    [
      "HP:0001197",
      "HP:0001508",
      "HP:0001522",
      "HP:0003811",
      "HP:0003819",
      "HP:0010967"
    ],
    [
      "CCRD:70",
      "OMIM:201450",
      "ORPHA:42"
    ]
  ],
  [
    [
      "HP:0000741",
      "HP:0000976",
      "HP:0000988",
      "HP:0001290",
      "HP:0001522",
      "HP:0001942",
      "HP:0001987",
      "HP:0002013",
      "HP:0002151",
      "HP:0002240",
      "HP:0003209",
      "HP:0003353",
      "HP:0003355",
```

```
      "HP:0010895",
      "HP:0011968",
      "HP:0040156"
    ],
    [
      "CCRD:44",
      "OMIM:253270",
      "ORPHA:79242"
    ]
  ],
  [
    [
      "HP:0001522",
      "HP:0003231",
      "HP:0003811",
      "HP:0003819",
      "HP:0004923",
      "HP:0011421",
      "HP:0040156"
    ],
    [
      "CCRD:90",
      "OMIM:261600",
      "ORPHA:716"
    ]
  ],
  [
    [
      "HP:0001249",
      "HP:0001250",
      "HP:0001264",
      "HP:0001270",
      "HP:0001513",
      "HP:0001903",
      "HP:0001987",
      "HP:0002344",
      "HP:0002355",
      "HP:0002510",
      "HP:0003218",
      "HP:0003355",
      "HP:0003551",
      "HP:0003645",
      "HP:0003819",
      "HP:0009046",
      "HP:0010472",
      "HP:0010899",
      "HP:0010907",
      "HP:0010909",
      "HP:0010916",
      "HP:0011421",
      "HP:0012026",
      "HP:0012127",
      "HP:0025435",
      "HP:0100613",
      "HP:0200119",
      "HP:0200123"
```

```
    ],
    [
      "CCRD:6",
      "OMIM:207800",
      "ORPHA:90"
    ]
  ],
  [
    [
      "HP:0001197",
      "HP:0001254",
      "HP:0001522",
      "HP:0001987",
      "HP:0002329",
      "HP:0002360",
      "HP:0003811",
      "HP:0003819",
      "HP:0010895",
      "HP:0010967",
      "HP:0011968"
    ],
    [
      "CCRD:58",
      "OMIM:243500",
      "ORPHA:33"
    ]
  ],
  [
    [
      "HP:0001197",
      "HP:0001522",
      "HP:0001943",
      "HP:0003355",
      "HP:0003811",
      "HP:0010895",
      "HP:0010967",
      "HP:0012638",
      "HP:0040156"
    ],
    [
      "OMIM:210200",
      "ORPHA:6"
    ]
  ],
  [
    [
      "HP:0000508",
      "HP:0000529",
      "HP:0000602",
      "HP:0001315",
      "HP:0003200",
      "HP:0003324",
      "HP:0003700",
      "HP:0200119",
      "HP:0200123"
    ],
```

```
    [
      "OMIM:251880",
      "ORPHA:279934"
    ]
  ],
  [
    [
      "HP:0001332",
      "HP:0001760",
      "HP:0002355",
      "HP:0003202",
      "HP:0003231",
      "HP:0003551",
      "HP:0003819",
      "HP:0004923",
      "HP:0005781",
      "HP:0009046",
      "HP:0009128",
      "HP:0010472",
      "HP:0011421"
    ],
    [
      "OMIM:128230",
      "ORPHA:98808"
    ]
  ],
  [
    [
      "HP:0003131",
      "HP:0003268",
      "HP:0003532",
      "HP:0010908",
      "HP:0011421"
    ],
    [
      "OMIM:220100",
      "ORPHA:214"
    ]
  ],
  [
    [
      "HP:0000047",
      "HP:0000054",
      "HP:0000509",
      "HP:0001520",
      "HP:0001522",
      "HP:0001944",
      "HP:0001945",
      "HP:0002013",
      "HP:0002014",
      "HP:0002153",
      "HP:0002902",
      "HP:0003072",
      "HP:0003811",
      "HP:0003819",
      "HP:0004429",
```

```
      "HP:0011968",
      "HP:0012318",
      "HP:0030088",
      "HP:0031213"
    ],
    [
      "OMIM:201810",
      "ORPHA:90791"
    ]
  ],
  [
    [
      "HP:0001513",
      "HP:0001522",
      "HP:0001680",
      "HP:0001945",
      "HP:0002090",
      "HP:0003231",
      "HP:0003811",
      "HP:0003819",
      "HP:0004923",
      "HP:0011421",
      "HP:0100613"
    ],
    [
      "CCRD:90",
      "OMIM:261600",
      "ORPHA:716"
    ]
  ],
  [
    [
      "HP:0000752",
      "HP:0001290",
      "HP:0001522",
      "HP:0001622",
      "HP:0001987",
      "HP:0003811",
      "HP:0003819",
      "HP:0008315",
      "HP:0010899",
      "HP:0010916",
      "HP:0040081"
    ],
    [
      "CCRD:116",
      "OMIM:201475",
      "ORPHA:26793"
    ]
  ],
  [
    [
      "HP:0003231",
      "HP:0004923",
      "HP:0100613"
    ],
```

[
"CCRD:90",
"OMIM:261600",
"ORPHA:716"
]
],
[
[
"HP:0000752",
"HP:0001257",
"HP:0001522",
"HP:0001878",
"HP:0001942",
"HP:0003573",
"HP:0003811",
"HP:0004429",
"HP:0010472",
"HP:0025435"
],
[
"OMIM:266130",
"ORPHA:289846"
]
],
[
[
"HP:0000256",
"HP:0000486",
"HP:0000750",
"HP:0001250",
"HP:0001251",
"HP:0001254",
"HP:0001522",
"HP:0002060",
"HP:0002151",
"HP:0002329",
"HP:0002344",
"HP:0002360",
"HP:0003819",
"HP:0010899"
],
[
"OMIM:271900",
"ORPHA:141"
]
],
[
[
"HP:0000670",
"HP:0001249",
"HP:0001281",
"HP:0001290",
"HP:0001508",
"HP:0001522",
"HP:0002240",
"HP:0002653",

```
      "HP:0002900",
      "HP:0003124",
      "HP:0003152",
      "HP:0003155",
      "HP:0003510",
      "HP:0003819",
      "HP:0008315",
      "HP:0011421"
    ],
    [
      "OMIM:227810",
      "ORPHA:2088"
    ]
  ],
  [
    [
      "HP:0001251",
      "HP:0001337",
      "HP:0002305",
      "HP:0011421",
      "HP:0040144",
      "HP:0100613"
    ],
    [
      "OMIM:236792",
      "ORPHA:79314"
    ]
  ],
  [
    [
      "HP:0001250",
      "HP:0001522",
      "HP:0001987",
      "HP:0002181",
      "HP:0003218",
      "HP:0003811"
    ],
    [
      "CCRD:85",
      "OMIM:311250",
      "ORPHA:664"
    ]
  ],
  [
    [
      "HP:0001522",
      "HP:0001944",
      "HP:0003210",
      "HP:0003811",
      "HP:0011968"
    ],
    [
      "CCRD:71",
      "OMIM:251000",
      "ORPHA:27"
    ]
```

],
[
[
"HP:0001249",
"HP:0003510",
"HP:0003819",
"HP:0004342"
],
[
"CCRD:30",
"OMIM:230400",
"ORPHA:352",
"ORPHA:79239"
]
],
[
[
"HP:0002013",
"HP:0002027",
"HP:0003819",
"HP:0004429"
],
[
"CCRD:92",
"OMIM:176000",
"ORPHA:738",
"ORPHA:79276"
]
],
[
[
"HP:0001250",
"HP:0001251",
"HP:0001254",
"HP:0001347",
"HP:0001942",
"HP:0001945",
"HP:0001987",
"HP:0002014",
"HP:0002329",
"HP:0002360",
"HP:0002380",
"HP:0003218",
"HP:0003819",
"HP:0011421"
],
[
"CCRD:85",
"OMIM:311250",
"ORPHA:664"
]
],
[
[
"HP:0000047",
"HP:0001522",

"HP:0001622",
"HP:0003231",
"HP:0003510",
"HP:0003811",
"HP:0004923",
"HP:0100790"
],
[
"CCRD:90",
"OMIM:261600",
"ORPHA:716"
]
],
[
[
"HP:0000975",
"HP:0000988",
"HP:0001197",
"HP:0001290",
"HP:0001522",
"HP:0003155",
"HP:0003219",
"HP:0003231",
"HP:0003348",
"HP:0003811",
"HP:0010899",
"HP:0010967",
"HP:0025474",
"HP:0025475",
"HP:0030350",
"HP:0040081"
],
[
"OMIM:201470",
"ORPHA:26792"
]
],
[
[
"HP:0001250",
"HP:0001257",
"HP:0001522",
"HP:0001987",
"HP:0002151",
"HP:0002789",
"HP:0003218",
"HP:0003811",
"HP:0045045"
],
[
"CCRD:85",
"OMIM:311250",
"ORPHA:664"
]
],
[

```
    [
      "HP:0000741",
      "HP:0000750",
      "HP:0001250",
      "HP:0001270",
      "HP:0001290",
      "HP:0001399",
      "HP:0001510",
      "HP:0001522",
      "HP:0001635",
      "HP:0001733",
      "HP:0001738",
      "HP:0001873",
      "HP:0001882",
      "HP:0001903",
      "HP:0001919",
      "HP:0001944",
      "HP:0001987",
      "HP:0002090",
      "HP:0002149",
      "HP:0002151",
      "HP:0002154",
      "HP:0002181",
      "HP:0002209",
      "HP:0002240",
      "HP:0002910",
      "HP:0003108",
      "HP:0003138",
      "HP:0003259",
      "HP:0003348",
      "HP:0003645",
      "HP:0003819",
      "HP:0004429",
      "HP:0010899",
      "HP:0010903",
      "HP:0010912",
      "HP:0010914",
      "HP:0010918",
      "HP:0010967",
      "HP:0010996",
      "HP:0012622",
      "HP:0025435",
      "HP:0040156"
    ],
    [
      "CCRD:99",
      "OMIM:606054",
      "ORPHA:35"
    ]
  ],
  [
    [
      "HP:0000750",
      "HP:0000821",
      "HP:0001260",
      "HP:0001264",
```

```
      "HP:0001298",
      "HP:0001347",
      "HP:0002510",
      "HP:0003219",
      "HP:0003688",
      "HP:0004429",
      "HP:0010895",
      "HP:0011421",
      "HP:0100613"
    ],
    [
      "OMIM:220110",
      "ORPHA:254905"
    ]
  ],
  [
    [
      "HP:0002013",
      "HP:0002027",
      "HP:0003819"
    ],
    [
      "CCRD:92",
      "OMIM:176000",
      "ORPHA:738",
      "ORPHA:79276"
    ]
  ],
  [
    [
      "HP:0001522",
      "HP:0001877",
      "HP:0001894",
      "HP:0003072",
      "HP:0003073",
      "HP:0003075",
      "HP:0003155",
      "HP:0003231",
      "HP:0003573",
      "HP:0004923",
      "HP:0006254",
      "HP:0010472",
      "HP:0012024"
    ],
    [
      "CCRD:115.1",
      "OMIM:276700",
      "ORPHA:882"
    ]
  ],
  [
    [
      "HP:0000147",
      "HP:0000976",
      "HP:0000988",
      "HP:0001290",
```

```
      "HP:0001513",
      "HP:0001522",
      "HP:0001945",
      "HP:0002013",
      "HP:0002014",
      "HP:0003231",
      "HP:0003819",
      "HP:0004386",
      "HP:0004429",
      "HP:0004923",
      "HP:0011421",
      "HP:0100613",
      "HP:0200039"
    ],
    [
      "CCRD:90",
      "OMIM:261600",
      "ORPHA:716"
    ]
  ],
  [
    [
      "HP:0001250",
      "HP:0001522",
      "HP:0001744",
      "HP:0002093"
    ],
    [
      "OMIM:220100",
      "ORPHA:214"
    ]
  ],
  [
    [
      "HP:0000750",
      "HP:0001513",
      "HP:0001522",
      "HP:0003348",
      "HP:0003355",
      "HP:0003573",
      "HP:0004342",
      "HP:0010899",
      "HP:0010917",
      "HP:0011421",
      "HP:0012024"
    ],
    [
      "CCRD:30",
      "OMIM:230400",
      "ORPHA:352",
      "ORPHA:79239"
    ]
  ],
  [
    [
      "HP:0000741",
```

```
      "HP:0000961",
      "HP:0001251",
      "HP:0001347",
      "HP:0001522",
      "HP:0001942",
      "HP:0001943",
      "HP:0002013",
      "HP:0002045",
      "HP:0002154",
      "HP:0002912",
      "HP:0003210",
      "HP:0003348",
      "HP:0003819",
      "HP:0004429",
      "HP:0010909",
      "HP:0010910",
      "HP:0010967",
      "HP:0011966",
      "HP:0012026",
      "HP:0012120",
      "HP:0012343",
      "HP:0100660"
    ],
    [
      "CCRD:71",
      "OMIM:251000",
      "ORPHA:27"
    ]
  ],
  [
    [
      "HP:0001197",
      "HP:0001522",
      "HP:0003231",
      "HP:0003811",
      "HP:0004923"
    ],
    [
      "CCRD:90",
      "OMIM:261600",
      "ORPHA:716"
    ]
  ],
  [
    [
      "HP:0001290",
      "HP:0001522",
      "HP:0001824",
      "HP:0001945",
      "HP:0002094",
      "HP:0003218",
      "HP:0003811",
      "HP:0030215"
    ],
    [
      "CCRD:85",
```

```
      "OMIM:311250",
      "ORPHA:664"
    ]
  ],
  [
    [
      "HP:0000737",
      "HP:0001257",
      "HP:0001347",
      "HP:0001522",
      "HP:0003231",
      "HP:0003811",
      "HP:0003819",
      "HP:0004429",
      "HP:0004923",
      "HP:0011421"
    ],
    [
      "CCRD:90",
      "OMIM:261600",
      "ORPHA:716"
    ]
  ],
  [
    [
      "HP:0001251",
      "HP:0001254",
      "HP:0001290",
      "HP:0001522",
      "HP:0001942",
      "HP:0002329",
      "HP:0002360",
      "HP:0003150"
    ],
    [
      "CCRD:34.1",
      "OMIM:231670",
      "ORPHA:25"
    ]
  ],
  [
    [
      "HP:0001290",
      "HP:0001522",
      "HP:0001649",
      "HP:0001945",
      "HP:0001987",
      "HP:0003110",
      "HP:0003811",
      "HP:0011968"
    ],
    [
      "OMIM:237300",
      "ORPHA:147"
    ]
  ],
```

```
[
  [
    "HP:0000256",
    "HP:0000750",
    "HP:0001197",
    "HP:0001249",
    "HP:0001251",
    "HP:0001270",
    "HP:0001522",
    "HP:0001987",
    "HP:0002179",
    "HP:0002240",
    "HP:0003355",
    "HP:0003811",
    "HP:0003819",
    "HP:0010907",
    "HP:0010909",
    "HP:0011966",
    "HP:0012127"
  ],
  [
    "OMIM:207900",
    "ORPHA:23"
  ]
],
[
  [
    "HP:0000486",
    "HP:0001197",
    "HP:0001249",
    "HP:0001513",
    "HP:0001522",
    "HP:0003231",
    "HP:0003811",
    "HP:0004923",
    "HP:0011421",
    "HP:0100613"
  ],
  [
    "CCRD:90",
    "OMIM:261600",
    "ORPHA:716"
  ]
],
[
  [
    "HP:0000256",
    "HP:0000476",
    "HP:0001522",
    "HP:0003150",
    "HP:0003530"
  ],
  [
    "CCRD:34.1",
    "OMIM:231670",
    "ORPHA:25"
```

```
    ]
  ],
  [
    [
      "HP:0000741",
      "HP:0001290",
      "HP:0001522",
      "HP:0001987",
      "HP:0002181",
      "HP:0003811",
      "HP:0011968"
    ],
    [
      "OMIM:237300",
      "ORPHA:147"
    ]
  ],
  [
    [
      "HP:0001270",
      "HP:0001522",
      "HP:0010899"
    ],
    [
      "OMIM:271900",
      "ORPHA:141"
    ]
  ],
  [
    [
      "HP:0001249",
      "HP:0001513",
      "HP:0003231",
      "HP:0004923",
      "HP:0040126",
      "HP:0040156"
    ],
    [
      "CCRD:90",
      "OMIM:261600",
      "ORPHA:716"
    ]
  ],
  [
    [
      "HP:0000975",
      "HP:0001522",
      "HP:0001638",
      "HP:0001640",
      "HP:0002615",
      "HP:0002789",
      "HP:0003355",
      "HP:0003819",
      "HP:0010895",
      "HP:0011968",
      "HP:0040156"
```

```
    ],
    [
      "OMIM:210200",
      "ORPHA:6"
    ]
  ],
  [
    [
      "HP:0001987",
      "HP:0002154",
      "HP:0003348",
      "HP:0008315"
    ],
    [
      "CCRD:99",
      "OMIM:606054",
      "ORPHA:35"
    ]
  ],
  [
    [
      "HP:0001259",
      "HP:0001290",
      "HP:0001522",
      "HP:0001942",
      "HP:0001945",
      "HP:0001987",
      "HP:0002013",
      "HP:0002014",
      "HP:0002045",
      "HP:0002094",
      "HP:0002179",
      "HP:0002181",
      "HP:0002789",
      "HP:0003076",
      "HP:0003645",
      "HP:0003811",
      "HP:0004429",
      "HP:0100806"
    ],
    [
      "OMIM:237300",
      "ORPHA:147"
    ]
  ],
  [
    [
      "HP:0001197",
      "HP:0001513",
      "HP:0001522",
      "HP:0003811",
      "HP:0003819",
      "HP:0010967",
      "HP:0100950"
    ],
    [
```

```
      "CCRD:70",
      "OMIM:201450",
      "ORPHA:42"
    ]
  ],
  [
    [
      "HP:0001522",
      "HP:0003355",
      "HP:0003811",
      "HP:0010895",
      "HP:0010967",
      "HP:0040156"
    ],
    [
      "OMIM:210200",
      "ORPHA:6"
    ]
  ],
  [
    [
      "HP:0001272",
      "HP:0003819",
      "HP:0012642"
    ],
    [
      "OMIM:212065",
      "ORPHA:79318"
    ]
  ],
  [
    [
      "HP:0001254",
      "HP:0001257",
      "HP:0001290",
      "HP:0001522",
      "HP:0001903",
      "HP:0001992",
      "HP:0002329",
      "HP:0002360"
    ],
    [
      "CCRD:13",
      "OMIM:253260",
      "ORPHA:79241"
    ]
  ],
  [
    [
      "HP:0000278",
      "HP:0001197",
      "HP:0001250",
      "HP:0001290",
      "HP:0001522",
      "HP:0002104",
      "HP:0002179",
```

```
      "HP:0003231",
      "HP:0003811",
      "HP:0003819",
      "HP:0004923"
    ],
    [
      "CCRD:90",
      "OMIM:261600",
      "ORPHA:716"
    ]
  ],
  [
    [
      "HP:0001250",
      "HP:0001259",
      "HP:0001290",
      "HP:0001522",
      "HP:0001987",
      "HP:0002013",
      "HP:0002151",
      "HP:0002240",
      "HP:0002789",
      "HP:0003811",
      "HP:0011968",
      "HP:0012719",
      "HP:0200114"
    ],
    [
      "OMIM:207900",
      "ORPHA:23"
    ]
  ],
  [
    [
      "HP:0001197",
      "HP:0001522",
      "HP:0003231",
      "HP:0003811",
      "HP:0004923"
    ],
    [
      "CCRD:90",
      "OMIM:261600",
      "ORPHA:716"
    ]
  ],
  [
    [
      "HP:0000256",
      "HP:0000476",
      "HP:0000975",
      "HP:0001254",
      "HP:0001290",
      "HP:0001332",
      "HP:0001522",
      "HP:0001942",
```

```
      "HP:0001945",
      "HP:0002013",
      "HP:0002014",
      "HP:0002094",
      "HP:0002329",
      "HP:0002360",
      "HP:0003150",
      "HP:0003530",
      "HP:0003811",
      "HP:0003819",
      "HP:0008315",
      "HP:0100660"
    ],
    [
      "CCRD:34.1",
      "OMIM:231670",
      "ORPHA:25"
    ]
  ],
  [
    [
      "HP:0001197",
      "HP:0001522",
      "HP:0001760",
      "HP:0001945",
      "HP:0002789",
      "HP:0003811",
      "HP:0003819",
      "HP:0008315"
    ],
    [
      "CCRD:70",
      "OMIM:201450",
      "ORPHA:42"
    ]
  ],
  [
    [
      "HP:0001290",
      "HP:0001522",
      "HP:0001662",
      "HP:0002045",
      "HP:0002104",
      "HP:0003571",
      "HP:0003811",
      "HP:0011968",
      "HP:0012378"
    ],
    [
      "CCRD:99",
      "OMIM:606054",
      "ORPHA:35"
    ]
  ],
  [
    [
```

```
      "HP:0000822",
      "HP:0001249",
      "HP:0001513",
      "HP:0003231",
      "HP:0004923",
      "HP:0011421",
      "HP:0040156",
      "HP:0100613"
    ],
    [
      "CCRD:90",
      "OMIM:261600",
      "ORPHA:716"
    ]
  ],
  [
    [
      "HP:0001257",
      "HP:0002090",
      "HP:0003344",
      "HP:0003819",
      "HP:0004360"
    ],
    [
      "OMIM:250951",
      "ORPHA:67048"
    ]
  ],
  [
    [
      "HP:0001522",
      "HP:0001943",
      "HP:0002013",
      "HP:0002014",
      "HP:0002910",
      "HP:0010967"
    ],
    [
      "CCRD:70",
      "OMIM:201450",
      "ORPHA:42"
    ]
  ],
  [
    [
      "HP:0000486",
      "HP:0001249",
      "HP:0001251",
      "HP:0001270",
      "HP:0001272",
      "HP:0001513",
      "HP:0001522",
      "HP:0001907",
      "HP:0003819",
      "HP:0012642"
    ],
```

```
    [
      "OMIM:212065",
      "ORPHA:79318"
    ]
  ],
  [
    [
      "HP:0000776",
      "HP:0001249",
      "HP:0001270",
      "HP:0001508",
      "HP:0001522",
      "HP:0002036",
      "HP:0002059",
      "HP:0009125",
      "HP:0011804"
    ],
    [
      "OMIM:212065",
      "ORPHA:79318"
    ]
  ],
  [
    [
      "HP:0001197",
      "HP:0001522",
      "HP:0001987",
      "HP:0003811",
      "HP:0003819",
      "HP:0010895",
      "HP:0010967"
    ],
    [
      "CCRD:58",
      "OMIM:243500",
      "ORPHA:33"
    ]
  ],
  [
    [
      "HP:0000486",
      "HP:0001249",
      "HP:0001251",
      "HP:0001272",
      "HP:0001290",
      "HP:0001522",
      "HP:0001999",
      "HP:0002648",
      "HP:0012642"
    ],
    [
      "OMIM:212065",
      "ORPHA:79318"
    ]
  ],
  [
```

```
    [
      "HP:0003468",
      "HP:0003819",
      "HP:0040319"
    ],
    [
      "OMIM:203500",
      "ORPHA:56"
    ]
  ],
  [
    [
      "HP:0001522",
      "HP:0001903",
      "HP:0002160",
      "HP:0010472",
      "HP:0040087",
      "HP:0040126"
    ],
    [
      "CCRD:45",
      "OMIM:236200",
      "ORPHA:394"
    ]
  ],
  [
    [
      "HP:0001943",
      "HP:0003076",
      "HP:0010472"
    ],
    [
      "OMIM:125850",
      "ORPHA:552"
    ]
  ],
  [
    [
      "HP:0000976",
      "HP:0000988",
      "HP:0001250",
      "HP:0001254",
      "HP:0001522",
      "HP:0002019",
      "HP:0002093",
      "HP:0002329",
      "HP:0002360",
      "HP:0003219",
      "HP:0003688",
      "HP:0010895"
    ],
    [
      "OMIM:220110",
      "ORPHA:254905"
    ]
  ],
```

```
[
  [
    "HP:0000988",
    "HP:0001522",
    "HP:0003076",
    "HP:0003219",
    "HP:0003688",
    "HP:0010895",
    "HP:0011968",
    "HP:0012719",
    "HP:0025474",
    "HP:0025475",
    "HP:0030350"
  ],
  [
    "OMIM:220110",
    "ORPHA:254905"
  ]
],
[
  [
    "HP:0001250",
    "HP:0001254",
    "HP:0001290",
    "HP:0001522",
    "HP:0002329",
    "HP:0002360",
    "HP:0003811",
    "HP:0011968",
    "HP:0012120"
  ],
  [
    "CCRD:71",
    "OMIM:251000",
    "ORPHA:27"
  ]
],
[
  [
    "HP:0001197",
    "HP:0001522",
    "HP:0003231",
    "HP:0003811",
    "HP:0004923",
    "HP:0040156",
    "HP:0100613"
  ],
  [
    "CCRD:90",
    "OMIM:261600",
    "ORPHA:716"
  ]
],
[
  [
    "HP:0001259",
```

```
      "HP:0001522",
      "HP:0002153",
      "HP:0002902",
      "HP:0003811",
      "HP:0005972",
      "HP:0040156"
    ],
    [
      "OMIM:271980",
      "ORPHA:22"
    ]
  ],
  [
    [
      "HP:0000639",
      "HP:0001251",
      "HP:0001315",
      "HP:0002151",
      "HP:0002490",
      "HP:0003150",
      "HP:0003542",
      "HP:0008315",
      "HP:0010701",
      "HP:0012117",
      "HP:0012318",
      "HP:0100613"
    ],
    [
      "CCRD:34.1",
      "OMIM:231670",
      "ORPHA:25"
    ]
  ],
  [
    [
      "HP:0001197",
      "HP:0001522",
      "HP:0003811",
      "HP:0003819",
      "HP:0010967"
    ],
    [
      "CCRD:70",
      "OMIM:201450",
      "ORPHA:42"
    ]
  ],
  [
    [
      "HP:0001197",
      "HP:0001522",
      "HP:0003231",
      "HP:0003811",
      "HP:0004923"
    ],
    [
```

```
      "CCRD:90",
      "OMIM:261600",
      "ORPHA:716"
    ]
  ],
  [
    [
      "HP:0000670",
      "HP:0001249",
      "HP:0003819",
      "HP:0004923",
      "HP:0008064",
      "HP:0010917",
      "HP:0011421"
    ],
    [
      "CCRD:90",
      "OMIM:261600",
      "ORPHA:716"
    ]
  ],
  [
    [
      "HP:0001522",
      "HP:0001987",
      "HP:0003573",
      "HP:0003811",
      "HP:0011966"
    ],
    [
      "CCRD:18",
      "OMIM:215700",
      "ORPHA:187",
      "ORPHA:247525"
    ]
  ],
  [
    [
      "HP:0001197",
      "HP:0001522",
      "HP:0003231",
      "HP:0003811",
      "HP:0003819",
      "HP:0004923"
    ],
    [
      "CCRD:90",
      "OMIM:261600",
      "ORPHA:716"
    ]
  ],
  [
    [
      "HP:0001290",
      "HP:0001522",
      "HP:0001622",
```

```
      "HP:0001903",
      "HP:0003231",
      "HP:0003811",
      "HP:0003819",
      "HP:0004923",
      "HP:0012343",
      "HP:0031437",
      "HP:0040303",
      "HP:0100511"
    ],
    [
      "CCRD:90",
      "OMIM:261600",
      "ORPHA:716"
    ]
  ],
  [
    [
      "HP:0001197",
      "HP:0001522",
      "HP:0003811",
      "HP:0004923",
      "HP:0010917"
    ],
    [
      "CCRD:90",
      "OMIM:261600",
      "ORPHA:716"
    ]
  ],
  [
    [
      "HP:0000486",
      "HP:0001249",
      "HP:0001250",
      "HP:0001270",
      "HP:0001272",
      "HP:0001522",
      "HP:0002240",
      "HP:0003075",
      "HP:0003146",
      "HP:0009125",
      "HP:0012642",
      "HP:0200119",
      "HP:0200123"
    ],
    [
      "OMIM:212065",
      "ORPHA:79318"
    ]
  ],
  [
    [
      "HP:0001197",
      "HP:0001522",
      "HP:0003231",
```

```
      "HP:0003811",
      "HP:0004386",
      "HP:0004923"
    ],
    [
      "CCRD:90",
      "OMIM:261600",
      "ORPHA:716"
    ]
  ],
  [
    [
      "HP:0000976",
      "HP:0000988",
      "HP:0001197",
      "HP:0001522",
      "HP:0002910",
      "HP:0003811",
      "HP:0010899",
      "HP:0010916",
      "HP:0010967",
      "HP:0100950"
    ],
    [
      "CCRD:70",
      "OMIM:201450",
      "ORPHA:42"
    ]
  ],
  [
    [
      "HP:0000252",
      "HP:0000670",
      "HP:0000750",
      "HP:0001197",
      "HP:0001522",
      "HP:0003231",
      "HP:0003811",
      "HP:0003819",
      "HP:0004923",
      "HP:0040156"
    ],
    [
      "CCRD:90",
      "OMIM:261600",
      "ORPHA:716"
    ]
  ],
  [
    [
      "HP:0000752",
      "HP:0001061",
      "HP:0001249",
      "HP:0001513",
      "HP:0003231",
      "HP:0003510",
```

```
        "HP:0003819",
        "HP:0004923",
        "HP:0011421",
        "HP:0100613"
      ],
      [
        "CCRD:90",
        "OMIM:261600",
        "ORPHA:716"
      ]
    ],
    [
      [
        "HP:0001522",
        "HP:0001892",
        "HP:0001987",
        "HP:0002013",
        "HP:0003218",
        "HP:0003256"
      ],
      [
        "CCRD:85",
        "OMIM:311250",
        "ORPHA:664"
      ]
    ],
    [
      [
        "HP:0001290",
        "HP:0001510",
        "HP:0001522",
        "HP:0001649",
        "HP:0001903",
        "HP:0002789",
        "HP:0003811",
        "HP:0012638",
        "HP:0040156"
      ],
      [
        "CCRD:67",
        "OMIM:248600",
        "ORPHA:511"
      ]
    ],
    [
      [
        "HP:0001919",
        "HP:0002912",
        "HP:0008315",
        "HP:0010472",
        "HP:0011421",
        "HP:0012100",
        "HP:0012120",
        "HP:0012622",
        "HP:0100613"
      ],
```

    [
      "CCRD:71",
      "OMIM:251000",
      "ORPHA:27"
    ]
  ],
  [
    [
      "HP:0000750",
      "HP:0001197",
      "HP:0001522",
      "HP:0003231",
      "HP:0003811",
      "HP:0003819",
      "HP:0004923"
    ],
    [
      "CCRD:90",
      "OMIM:261600",
      "ORPHA:716"
    ]
  ],
  [
    [
      "HP:0001290",
      "HP:0001522",
      "HP:0002240",
      "HP:0002789",
      "HP:0003535",
      "HP:0003811",
      "HP:0011968"
    ],
    [
      "OMIM:237300",
      "ORPHA:147"
    ]
  ],
  [
    [
      "HP:0001197",
      "HP:0001522",
      "HP:0001877",
      "HP:0001894",
      "HP:0003811",
      "HP:0003819",
      "HP:0004342",
      "HP:0010472",
      "HP:0100511"
    ],
    [
      "CCRD:30",
      "OMIM:230400",
      "ORPHA:352",
      "ORPHA:79239"
    ]
  ],

```
[
  [
    "HP:0001197",
    "HP:0001251",
    "HP:0001513",
    "HP:0001522",
    "HP:0002104",
    "HP:0003231",
    "HP:0003401",
    "HP:0003811",
    "HP:0004923",
    "HP:0100613"
  ],
  [
    "CCRD:90",
    "OMIM:261600",
    "ORPHA:716"
  ]
],
[
  [
    "HP:0001254",
    "HP:0001522",
    "HP:0002329",
    "HP:0002360",
    "HP:0003218",
    "HP:0012025"
  ],
  [
    "CCRD:85",
    "OMIM:311250",
    "ORPHA:664"
  ]
],
[
  [
    "HP:0001522",
    "HP:0001942",
    "HP:0001987",
    "HP:0003108",
    "HP:0003217",
    "HP:0003268",
    "HP:0003811",
    "HP:0010909",
    "HP:0011966"
  ],
  [
    "CCRD:18",
    "OMIM:215700",
    "ORPHA:187",
    "ORPHA:247525"
  ]
],
[
  [
    "HP:0001251",
```

```
      "HP:0001332",
      "HP:0002060",
      "HP:0003150",
      "HP:0003819",
      "HP:0008315",
      "HP:0012447"
    ],
    [
      "CCRD:34.1",
      "OMIM:231670",
      "ORPHA:25"
    ]
  ],
  [
    [
      "HP:0001197",
      "HP:0001522",
      "HP:0001943",
      "HP:0001944",
      "HP:0001945",
      "HP:0002013",
      "HP:0002014",
      "HP:0003811",
      "HP:0003819",
      "HP:0004386",
      "HP:0008315",
      "HP:0012378"
    ],
    [
      "CCRD:70",
      "OMIM:201450",
      "ORPHA:42"
    ]
  ],
  [
    [
      "HP:0000741",
      "HP:0001250",
      "HP:0001290",
      "HP:0001522",
      "HP:0001622",
      "HP:0001943",
      "HP:0001974",
      "HP:0001987",
      "HP:0002155",
      "HP:0003510",
      "HP:0003811",
      "HP:0003819",
      "HP:0004429",
      "HP:0010899",
      "HP:0010916",
      "HP:0010967"
    ],
    [
      "CCRD:70",
      "OMIM:201450",
```

```
      "ORPHA:42"
    ]
  ],
  [
    [
      "HP:0001250",
      "HP:0001254",
      "HP:0001347",
      "HP:0001945",
      "HP:0001987",
      "HP:0002013",
      "HP:0002181",
      "HP:0002329",
      "HP:0002360",
      "HP:0003218",
      "HP:0003819",
      "HP:0012025"
    ],
    [
      "CCRD:85",
      "OMIM:311250",
      "ORPHA:664"
    ]
  ],
  [
    [
      "HP:0000752",
      "HP:0001249",
      "HP:0001250",
      "HP:0001513",
      "HP:0003231",
      "HP:0003510",
      "HP:0003819",
      "HP:0004923",
      "HP:0011421",
      "HP:0040156",
      "HP:0100613"
    ],
    [
      "CCRD:90",
      "OMIM:261600",
      "ORPHA:716"
    ]
  ],
  [
    [
      "HP:0001522",
      "HP:0001987",
      "HP:0003218",
      "HP:0003811"
    ],
    [
      "OMIM:237300",
      "ORPHA:147"
    ]
  ],
```

[
  [
    "HP:0001197",
    "HP:0001522",
    "HP:0002013",
    "HP:0003811",
    "HP:0010967",
    "HP:0100950"
  ],
  [
    "CCRD:70",
    "OMIM:201450",
    "ORPHA:42"
  ]
],
[
  [
    "HP:0000750",
    "HP:0001249",
    "HP:0001522",
    "HP:0003296",
    "HP:0003811",
    "HP:0003819",
    "HP:0004342",
    "HP:0010899",
    "HP:0010916",
    "HP:0010917",
    "HP:0012023",
    "HP:0012024"
  ],
  [
    "CCRD:30",
    "OMIM:230400",
    "ORPHA:352",
    "ORPHA:79239"
  ]
],
[
  [
    "HP:0000750",
    "HP:0001270",
    "HP:0001290",
    "HP:0001518",
    "HP:0001522",
    "HP:0001622",
    "HP:0001945",
    "HP:0001987",
    "HP:0002013",
    "HP:0002151",
    "HP:0003155",
    "HP:0003218",
    "HP:0003355",
    "HP:0003811",
    "HP:0003819",
    "HP:0010895",
    "HP:0010899",

```
      "HP:0010907",
      "HP:0010909",
      "HP:0010911",
      "HP:0010916",
      "HP:0010967",
      "HP:0011966",
      "HP:0011968",
      "HP:0012378"
    ],
    [
      "OMIM:207900",
      "ORPHA:23"
    ]
  ],
  [
    [
      "HP:0001522",
      "HP:0001942",
      "HP:0003210",
      "HP:0003819"
    ],
    [
      "CCRD:71",
      "OMIM:251000",
      "ORPHA:27"
    ]
  ],
  [
    [
      "HP:0001249",
      "HP:0003401",
      "HP:0004923",
      "HP:0100613",
      "HP:0200119",
      "HP:0200123"
    ],
    [
      "CCRD:90",
      "OMIM:261600",
      "ORPHA:716"
    ]
  ],
  [
    [
      "HP:0001522",
      "HP:0001942",
      "HP:0003210",
      "HP:0003819"
    ],
    [
      "CCRD:71",
      "OMIM:251000",
      "ORPHA:27"
    ]
  ],
  [
```

```
    [
      "HP:0001197",
      "HP:0001522",
      "HP:0003231",
      "HP:0003811",
      "HP:0003819",
      "HP:0004923"
    ],
    [
      "CCRD:90",
      "OMIM:261600",
      "ORPHA:716"
    ]
  ],
  [
    [
      "HP:0000256",
      "HP:0000966",
      "HP:0000976",
      "HP:0000988",
      "HP:0001290",
      "HP:0001522",
      "HP:0001596",
      "HP:0001942",
      "HP:0003811",
      "HP:0011675",
      "HP:0040156"
    ],
    [
      "CCRD:13",
      "OMIM:253260",
      "ORPHA:79241"
    ]
  ],
  [
    [
      "HP:0000988",
      "HP:0001250",
      "HP:0001522",
      "HP:0001987",
      "HP:0003355",
      "HP:0003811",
      "HP:0010895",
      "HP:0010899",
      "HP:0025474",
      "HP:0025475",
      "HP:0030350"
    ],
    [
      "OMIM:271900",
      "ORPHA:141"
    ]
  ],
  [
    [
      "HP:0000750",
```

```
      "HP:0001197",
      "HP:0001522",
      "HP:0003110",
      "HP:0003811",
      "HP:0003819"
    ],
    [
      "CCRD:13",
      "OMIM:253260",
      "ORPHA:79241"
    ]
  ],
  [
    [
      "HP:0001942",
      "HP:0002013",
      "HP:0003819",
      "HP:0012120"
    ],
    [
      "CCRD:71",
      "OMIM:251000",
      "ORPHA:27"
    ]
  ],
  [
    [
      "HP:0001290",
      "HP:0001522",
      "HP:0001873",
      "HP:0001882",
      "HP:0001942",
      "HP:0002104",
      "HP:0003210",
      "HP:0003811",
      "HP:0011968",
      "HP:0012120",
      "HP:0012638"
    ],
    [
      "CCRD:71",
      "OMIM:251000",
      "ORPHA:27"
    ]
  ],
  [
    [
      "HP:0000750",
      "HP:0001250",
      "HP:0001290",
      "HP:0001522",
      "HP:0002179",
      "HP:0003811",
      "HP:0003819",
      "HP:0012321"
    ],
```

```
    [
      "OMIM:600721",
      "ORPHA:79315"
    ]
  ],
  [
    [
      "HP:0000976",
      "HP:0000988",
      "HP:0001197",
      "HP:0001522",
      "HP:0003231",
      "HP:0003811",
      "HP:0003819",
      "HP:0004923",
      "HP:0040156"
    ],
    [
      "CCRD:90",
      "OMIM:261600",
      "ORPHA:716"
    ]
  ],
  [
    [
      "HP:0001522",
      "HP:0001987",
      "HP:0002094",
      "HP:0003110",
      "HP:0003811",
      "HP:0012638"
    ],
    [
      "OMIM:237300",
      "ORPHA:147"
    ]
  ],
  [
    [
      "HP:0000238",
      "HP:0000348",
      "HP:0001257",
      "HP:0001290",
      "HP:0001522",
      "HP:0002059",
      "HP:0002305",
      "HP:0003150",
      "HP:0003819"
    ],
    [
      "CCRD:34.1",
      "OMIM:231670",
      "ORPHA:25"
    ]
  ],
  [
```

```
    [
      "HP:0004923",
      "HP:0010917",
      "HP:0100613"
    ],
    [
      "CCRD:90",
      "OMIM:261600",
      "ORPHA:716"
    ]
  ],
  [
    [
      "HP:0001197",
      "HP:0001522",
      "HP:0003231",
      "HP:0003811",
      "HP:0003819",
      "HP:0004923"
    ],
    [
      "CCRD:90",
      "OMIM:261600",
      "ORPHA:716"
    ]
  ],
  [
    [
      "HP:0001254",
      "HP:0001290",
      "HP:0001522",
      "HP:0001942",
      "HP:0001945",
      "HP:0002151",
      "HP:0002329",
      "HP:0002360",
      "HP:0003209",
      "HP:0003353",
      "HP:0003355",
      "HP:0010280",
      "HP:0010307",
      "HP:0010895",
      "HP:0012378",
      "HP:0040156",
      "HP:0100758"
    ],
    [
      "CCRD:13",
      "OMIM:253260",
      "ORPHA:79241"
    ]
  ],
  [
    [
      "HP:0000518",
      "HP:0001197",
```

```
        "HP:0001399",
        "HP:0001522",
        "HP:0001631",
        "HP:0001987",
        "HP:0002908",
        "HP:0003573",
        "HP:0003607",
        "HP:0003645",
        "HP:0003811",
        "HP:0003819",
        "HP:0004342",
        "HP:0010899",
        "HP:0010916",
        "HP:0012024"
      ],
      [
        "CCRD:30",
        "OMIM:230400",
        "ORPHA:352",
        "ORPHA:79239"
      ]
    ],
    [
      [
        "HP:0000980",
        "HP:0001522",
        "HP:0001541",
        "HP:0001894",
        "HP:0001903",
        "HP:0003073",
        "HP:0003075",
        "HP:0003155",
        "HP:0003231",
        "HP:0003355",
        "HP:0006254",
        "HP:0010472",
        "HP:0100790"
      ],
      [
        "CCRD:115.1",
        "OMIM:276700",
        "ORPHA:882"
      ]
    ],
    [
      [
        "HP:0003231",
        "HP:0004923",
        "HP:0100613"
      ],
      [
        "CCRD:90",
        "OMIM:261600",
        "ORPHA:716"
      ]
    ],
```

```
[
  [
    "HP:0001257",
    "HP:0001522",
    "HP:0001622",
    "HP:0002595",
    "HP:0003811",
    "HP:0003819",
    "HP:0008315",
    "HP:0012343",
    "HP:0012418",
    "HP:0030995",
    "HP:0040126",
    "HP:0100950"
  ],
  [
    "CCRD:70",
    "OMIM:201450",
    "ORPHA:42"
  ]
],
[
  [
    "HP:0001197",
    "HP:0001522",
    "HP:0003231",
    "HP:0003811",
    "HP:0003819",
    "HP:0004923"
  ],
  [
    "CCRD:90",
    "OMIM:261600",
    "ORPHA:716"
  ]
],
[
  [
    "HP:0001197",
    "HP:0001522",
    "HP:0003811",
    "HP:0003819",
    "HP:0010967"
  ],
  [
    "CCRD:70",
    "OMIM:201450",
    "ORPHA:42"
  ]
],
[
  [
    "HP:0001197",
    "HP:0001522",
    "HP:0003811",
    "HP:0010967"
```

```
    ],
    [
      "CCRD:70",
      "OMIM:201450",
      "ORPHA:42"
    ]
  ],
  [
    [
      "HP:0001522",
      "HP:0003231",
      "HP:0003819",
      "HP:0004429",
      "HP:0004923",
      "HP:0010472",
      "HP:0011421",
      "HP:0012343",
      "HP:0040156"
    ],
    [
      "CCRD:90",
      "OMIM:261600",
      "ORPHA:716"
    ]
  ],
  [
    [
      "HP:0000509",
      "HP:0000613",
      "HP:0000962",
      "HP:0001249",
      "HP:0001250",
      "HP:0003231",
      "HP:0003819",
      "HP:0009926"
    ],
    [
      "OMIM:276600",
      "ORPHA:28378"
    ]
  ],
  [
    [
      "HP:0000238",
      "HP:0001522",
      "HP:0001903",
      "HP:0003150",
      "HP:0003530",
      "HP:0008315"
    ],
    [
      "CCRD:34.1",
      "OMIM:231670",
      "ORPHA:25"
    ]
  ],
```

```
[
  [
    "HP:0000252",
    "HP:0001197",
    "HP:0001518",
    "HP:0001522",
    "HP:0001698",
    "HP:0002202",
    "HP:0003231",
    "HP:0003510",
    "HP:0003811",
    "HP:0003819",
    "HP:0004923",
    "HP:0011968"
  ],
  [
    "CCRD:90",
    "OMIM:261600",
    "ORPHA:716"
  ]
],
[
  [
    "HP:0001197",
    "HP:0001522",
    "HP:0003231",
    "HP:0003811",
    "HP:0004923"
  ],
  [
    "CCRD:90",
    "OMIM:261600",
    "ORPHA:716"
  ]
],
[
  [
    "HP:0001272",
    "HP:0001290",
    "HP:0001649",
    "HP:0001695",
    "HP:0003202",
    "HP:0003819",
    "HP:0004429",
    "HP:0009128",
    "HP:0012642",
    "HP:0040301"
  ],
  [
    "OMIM:307030",
    "ORPHA:408"
  ]
],
[
  [
    "HP:0000123",
```

```
      "HP:0001290",
      "HP:0001522",
      "HP:0001629",
      "HP:0001638",
      "HP:0001642"
    ],
    [
      "OMIM:261750",
      "ORPHA:79240"
    ]
  ],
  [
    [
      "HP:0001343",
      "HP:0001513",
      "HP:0001522",
      "HP:0001945",
      "HP:0003231",
      "HP:0003811",
      "HP:0004429",
      "HP:0004923",
      "HP:0011421",
      "HP:0040156"
    ],
    [
      "CCRD:90",
      "OMIM:261600",
      "ORPHA:716"
    ]
  ],
  [
    [
      "HP:0001197",
      "HP:0001522",
      "HP:0003231",
      "HP:0003811",
      "HP:0003819",
      "HP:0004923"
    ],
    [
      "CCRD:90",
      "OMIM:261600",
      "ORPHA:716"
    ]
  ],
  [
    [
      "HP:0000570",
      "HP:0000649",
      "HP:0001250",
      "HP:0001257",
      "HP:0001522",
      "HP:0001943",
      "HP:0001945",
      "HP:0001987",
      "HP:0002060",
```

"HP:0003150",
"HP:0003530",
"HP:0008315",
"HP:0010551",
"HP:0045045"
],
[
"CCRD:34.1",
"OMIM:231670",
"ORPHA:25"
]
],
[
[
"HP:0000976",
"HP:0000988",
"HP:0001250",
"HP:0001522",
"HP:0001945",
"HP:0011227",
"HP:0040144",
"HP:0040303"
],
[
"OMIM:236792",
"ORPHA:79314"
]
],
[
[
"HP:0000750",
"HP:0001251",
"HP:0001260",
"HP:0001264",
"HP:0001270",
"HP:0001290",
"HP:0001347",
"HP:0001510",
"HP:0001522",
"HP:0001760",
"HP:0002014",
"HP:0002134",
"HP:0002151",
"HP:0002510",
"HP:0003219",
"HP:0003542",
"HP:0003688",
"HP:0003819",
"HP:0011421"
],
[
"OMIM:220110",
"ORPHA:254905"
]
],
[

```
    [
      "HP:0001254",
      "HP:0001522",
      "HP:0001824",
      "HP:0001944",
      "HP:0001987",
      "HP:0002090",
      "HP:0002094",
      "HP:0002179",
      "HP:0002181",
      "HP:0002329",
      "HP:0002360",
      "HP:0002789",
      "HP:0003217",
      "HP:0003218",
      "HP:0003348",
      "HP:0003355",
      "HP:0003811",
      "HP:0003819",
      "HP:0004353",
      "HP:0004386",
      "HP:0004429",
      "HP:0010899",
      "HP:0010907",
      "HP:0010909",
      "HP:0010967",
      "HP:0011421",
      "HP:0011966",
      "HP:0012127",
      "HP:0040156"
    ],
    [
      "CCRD:85",
      "OMIM:311250",
      "ORPHA:664"
    ]
  ],
  [
    [
      "HP:0001522",
      "HP:0001942",
      "HP:0001987",
      "HP:0002013",
      "HP:0012120"
    ],
    [
      "CCRD:71",
      "OMIM:251000",
      "ORPHA:27"
    ]
  ],
  [
    [
      "HP:0000988",
      "HP:0001197",
      "HP:0001513",
```

```
      "HP:0001522",
      "HP:0002014",
      "HP:0003231",
      "HP:0003811",
      "HP:0003819",
      "HP:0004386",
      "HP:0004429",
      "HP:0004923",
      "HP:0025474",
      "HP:0025475",
      "HP:0030350",
      "HP:0100790"
    ],
    [
      "CCRD:90",
      "OMIM:261600",
      "ORPHA:716"
    ]
  ],
  [
    [
      "HP:0000741",
      "HP:0001254",
      "HP:0001522",
      "HP:0001942",
      "HP:0001943",
      "HP:0002329",
      "HP:0002360",
      "HP:0003076",
      "HP:0003819",
      "HP:0011033"
    ],
    [
      "OMIM:229700",
      "ORPHA:348"
    ]
  ],
  [
    [
      "HP:0000750",
      "HP:0001249",
      "HP:0001347",
      "HP:0001522",
      "HP:0002355",
      "HP:0002500",
      "HP:0003551",
      "HP:0003819",
      "HP:0009046"
    ],
    [
      "OMIM:236792",
      "ORPHA:79314"
    ]
  ],
  [
    [
```

```
      "HP:0000709",
      "HP:0000976",
      "HP:0000988",
      "HP:0001249",
      "HP:0001522",
      "HP:0002027",
      "HP:0002321",
      "HP:0003231",
      "HP:0004923",
      "HP:0012318",
      "HP:0040156",
      "HP:0100613",
      "HP:0200039"
    ],
    [
      "CCRD:90",
      "OMIM:261600",
      "ORPHA:716"
    ]
  ],
  [
    [
      "HP:0001522",
      "HP:0001640",
      "HP:0001943",
      "HP:0002151",
      "HP:0002240",
      "HP:0002789",
      "HP:0003215",
      "HP:0003811",
      "HP:0003819",
      "HP:0004429",
      "HP:0010967"
    ],
    [
      "CCRD:116",
      "OMIM:201475",
      "ORPHA:26793"
    ]
  ],
  [
    [
      "HP:0000252",
      "HP:0000486",
      "HP:0000741",
      "HP:0000752",
      "HP:0001249",
      "HP:0001290",
      "HP:0001522",
      "HP:0001942",
      "HP:0001987",
      "HP:0002013",
      "HP:0002154",
      "HP:0003348",
      "HP:0003510",
      "HP:0003811",
```

```
      "HP:0003819",
      "HP:0004386",
      "HP:0010911",
      "HP:0010967",
      "HP:0011421",
      "HP:0011968"
    ],
    [
      "CCRD:99",
      "OMIM:606054",
      "ORPHA:35"
    ]
  ],
  [
    [
      "HP:0001290",
      "HP:0001513",
      "HP:0001522",
      "HP:0001629",
      "HP:0003231",
      "HP:0003811",
      "HP:0003819",
      "HP:0004429",
      "HP:0004923"
    ],
    [
      "CCRD:90",
      "OMIM:261600",
      "ORPHA:716"
    ]
  ],
  [
    [
      "HP:0002013",
      "HP:0003219",
      "HP:0003819",
      "HP:0010967",
      "HP:0011421",
      "HP:0100950"
    ],
    [
      "OMIM:201470",
      "ORPHA:26792"
    ]
  ],
  [
    [
      "HP:0001197",
      "HP:0001249",
      "HP:0001522",
      "HP:0003231",
      "HP:0003811",
      "HP:0003819",
      "HP:0004923",
      "HP:0040156"
    ],
```

```
      [
        "CCRD:90",
        "OMIM:261600",
        "ORPHA:716"
      ]
    ],
    [
      [
        "HP:0001250",
        "HP:0001290",
        "HP:0001510",
        "HP:0001522",
        "HP:0001629",
        "HP:0003468",
        "HP:0010280",
        "HP:0011356",
        "HP:0100758"
      ],
      [
        "CCRD:13",
        "OMIM:253260",
        "ORPHA:79241"
      ]
    ],
    [
      [
        "HP:0000741",
        "HP:0001522",
        "HP:0003218",
        "HP:0003811",
        "HP:0011968",
        "HP:0100806"
      ],
      [
        "OMIM:207900",
        "ORPHA:23"
      ]
    ],
    [
      [
        "HP:0001051",
        "HP:0001522",
        "HP:0002151",
        "HP:0002286",
        "HP:0002299",
        "HP:0003348",
        "HP:0003811",
        "HP:0003819",
        "HP:0011364",
        "HP:0011421"
      ],
      [
        "CCRD:13",
        "OMIM:253260",
        "ORPHA:79241"
      ]
```

```
  ],
  [
    [
      "HP:0003231",
      "HP:0004923",
      "HP:0100613"
    ],
    [
      "CCRD:90",
      "OMIM:261600",
      "ORPHA:716"
    ]
  ],
  [
    [
      "HP:0000988",
      "HP:0001522",
      "HP:0001596",
      "HP:0001942",
      "HP:0002490",
      "HP:0003355",
      "HP:0010895",
      "HP:0025474",
      "HP:0025475",
      "HP:0030350",
      "HP:0040156"
    ],
    [
      "CCRD:13",
      "OMIM:253260",
      "ORPHA:79241"
    ]
  ],
  [
    [
      "HP:0001522",
      "HP:0001945",
      "HP:0003231",
      "HP:0003819",
      "HP:0004429",
      "HP:0004923",
      "HP:0010472",
      "HP:0011421",
      "HP:0012343",
      "HP:0040156"
    ],
    [
      "CCRD:90",
      "OMIM:261600",
      "ORPHA:716"
    ]
  ],
  [
    [
      "HP:0001250",
      "HP:0001522",
```

```
      "HP:0001942",
      "HP:0002789",
      "HP:0003344",
      "HP:0003811"
    ],
    [
      "OMIM:237300",
      "ORPHA:147"
    ]
  ],
  [
    [
      "HP:0000365",
      "HP:0000750",
      "HP:0001513",
      "HP:0001522",
      "HP:0001945",
      "HP:0003231",
      "HP:0003510",
      "HP:0003811",
      "HP:0003819",
      "HP:0004923",
      "HP:0011421",
      "HP:0040156"
    ],
    [
      "CCRD:90",
      "OMIM:261600",
      "ORPHA:716"
    ]
  ],
  [
    [
      "HP:0000486",
      "HP:0000750",
      "HP:0001249",
      "HP:0001394",
      "HP:0001395",
      "HP:0001522",
      "HP:0002149",
      "HP:0002896",
      "HP:0003155",
      "HP:0003161",
      "HP:0003231",
      "HP:0003811",
      "HP:0003819",
      "HP:0006254",
      "HP:0010472",
      "HP:0010899",
      "HP:0010916",
      "HP:0011421",
      "HP:0012343",
      "HP:0040303"
    ],
    [
      "CCRD:115.1",
```

```
      "OMIM:276700",
      "ORPHA:882"
    ]
  ],
  [
    [
      "HP:0001259",
      "HP:0001522",
      "HP:0001699",
      "HP:0001943",
      "HP:0002013",
      "HP:0003155",
      "HP:0008315",
      "HP:0010899",
      "HP:0010916",
      "HP:0025435"
    ],
    [
      "CCRD:15",
      "OMIM:212140",
      "ORPHA:158"
    ]
  ],
  [
    [
      "HP:0000967",
      "HP:0001250",
      "HP:0001254",
      "HP:0001290",
      "HP:0001315",
      "HP:0001522",
      "HP:0002151",
      "HP:0002329",
      "HP:0002360",
      "HP:0003150",
      "HP:0003202",
      "HP:0003219",
      "HP:0003688",
      "HP:0009128"
    ],
    [
      "OMIM:220110",
      "ORPHA:254905"
    ]
  ],
  [
    [
      "HP:0000741",
      "HP:0001251",
      "HP:0001254",
      "HP:0001987",
      "HP:0002013",
      "HP:0002329",
      "HP:0002360",
      "HP:0003218",
      "HP:0011421"
```

```
    ],
    [
      "CCRD:85",
      "OMIM:311250",
      "ORPHA:664"
    ]
  ],
  [
    [
      "HP:0000028",
      "HP:0000822",
      "HP:0000939",
      "HP:0001254",
      "HP:0001264",
      "HP:0001290",
      "HP:0001347",
      "HP:0001649",
      "HP:0001760",
      "HP:0001903",
      "HP:0001919",
      "HP:0001945",
      "HP:0001987",
      "HP:0002013",
      "HP:0002149",
      "HP:0002153",
      "HP:0002154",
      "HP:0002209",
      "HP:0002329",
      "HP:0002355",
      "HP:0002360",
      "HP:0002510",
      "HP:0003072",
      "HP:0003075",
      "HP:0003138",
      "HP:0003210",
      "HP:0003228",
      "HP:0003259",
      "HP:0003348",
      "HP:0003510",
      "HP:0003551",
      "HP:0003819",
      "HP:0004357",
      "HP:0004429",
      "HP:0009046",
      "HP:0010472",
      "HP:0010912",
      "HP:0010914",
      "HP:0010967",
      "HP:0011421",
      "HP:0012120",
      "HP:0012239",
      "HP:0012622",
      "HP:0012714",
      "HP:0040081",
      "HP:0100613"
    ],
```

```
    [
      "CCRD:71",
      "OMIM:251000",
      "ORPHA:27"
    ]
  ],
  [
    [
      "HP:0000256",
      "HP:0001250",
      "HP:0001522",
      "HP:0001903",
      "HP:0001945",
      "HP:0003150",
      "HP:0003819"
    ],
    [
      "CCRD:34.1",
      "OMIM:231670",
      "ORPHA:25"
    ]
  ],
  [
    [
      "HP:0001197",
      "HP:0001249",
      "HP:0001513",
      "HP:0001522",
      "HP:0002060",
      "HP:0003231",
      "HP:0003811",
      "HP:0004923",
      "HP:0011421",
      "HP:0040156"
    ],
    [
      "CCRD:90",
      "OMIM:261600",
      "ORPHA:716"
    ]
  ],
  [
    [
      "HP:0001197",
      "HP:0001522",
      "HP:0002781",
      "HP:0003231",
      "HP:0003811",
      "HP:0004923",
      "HP:0006541"
    ],
    [
      "CCRD:90",
      "OMIM:261600",
      "ORPHA:716"
    ]
```

```
  ],
  [
    [
      "HP:0000123",
      "HP:0001508",
      "HP:0001510",
      "HP:0001522",
      "HP:0002013",
      "HP:0002014",
      "HP:0010917",
      "HP:0040156"
    ],
    [
      "OMIM:140350",
      "ORPHA:2118"
    ]
  ],
  [
    [
      "HP:0001513",
      "HP:0003124",
      "HP:0003231",
      "HP:0004923",
      "HP:0100613"
    ],
    [
      "CCRD:90",
      "OMIM:261600",
      "ORPHA:716"
    ]
  ],
  [
    [
      "HP:0000608",
      "HP:0000750",
      "HP:0001249",
      "HP:0001250",
      "HP:0001254",
      "HP:0001257",
      "HP:0001332",
      "HP:0001347",
      "HP:0001510",
      "HP:0001522",
      "HP:0001945",
      "HP:0001974",
      "HP:0002059",
      "HP:0002329",
      "HP:0002360",
      "HP:0002500",
      "HP:0002813",
      "HP:0003819",
      "HP:0004353",
      "HP:0010551",
      "HP:0012127"
    ],
    [
```

```
      "OMIM:274270",
      "ORPHA:1675"
    ]
  ],
  [
    [
      "HP:0002027",
      "HP:0003163",
      "HP:0003819"
    ],
    [
      "CCRD:92",
      "OMIM:176000",
      "ORPHA:738",
      "ORPHA:79276"
    ]
  ],
  [
    [
      "HP:0000158",
      "HP:0000256",
      "HP:0000280",
      "HP:0000752",
      "HP:0001744",
      "HP:0002240",
      "HP:0003819",
      "HP:0004429",
      "HP:0011421",
      "HP:0011968"
    ],
    [
      "CCRD:73",
      "OMIM:309900",
      "ORPHA:580",
      "ORPHA:79213"
    ]
  ],
  [
    [
      "HP:0000570",
      "HP:0001254",
      "HP:0001257",
      "HP:0001522",
      "HP:0001662",
      "HP:0001987",
      "HP:0002013",
      "HP:0002104",
      "HP:0002329",
      "HP:0002360",
      "HP:0003217",
      "HP:0003218",
      "HP:0003572",
      "HP:0003811"
    ],
    [
      "OMIM:237300",
```

```
      "ORPHA:147"
    ]
  ],
  [
    [
      "HP:0000980",
      "HP:0001522",
      "HP:0001541",
      "HP:0001894",
      "HP:0001903",
      "HP:0002240",
      "HP:0003073",
      "HP:0003075",
      "HP:0003155",
      "HP:0003231",
      "HP:0003355",
      "HP:0006254",
      "HP:0010472",
      "HP:0200039"
    ],
    [
      "CCRD:115.1",
      "OMIM:276700",
      "ORPHA:882"
    ]
  ],
  [
    [
      "HP:0001197",
      "HP:0001522",
      "HP:0003811",
      "HP:0010967"
    ],
    [
      "CCRD:116",
      "OMIM:201475",
      "ORPHA:26793"
    ]
  ],
  [
    [
      "HP:0001197",
      "HP:0001522",
      "HP:0002781",
      "HP:0003231",
      "HP:0003811",
      "HP:0003819",
      "HP:0004923",
      "HP:0006541",
      "HP:0011421",
      "HP:0100613"
    ],
    [
      "CCRD:90",
      "OMIM:261600",
      "ORPHA:716"
```

```
    ]
  ],
  [
    [
      "HP:0001522",
      "HP:0003811",
      "HP:0040156"
    ],
    [
      "CCRD:13",
      "OMIM:253260",
      "ORPHA:79241"
    ]
  ],
  [
    [
      "HP:0003355",
      "HP:0010895",
      "HP:0040156"
    ],
    [
      "OMIM:210200",
      "ORPHA:6"
    ]
  ],
  [
    [
      "HP:0001197",
      "HP:0001522",
      "HP:0003231",
      "HP:0003811",
      "HP:0004923"
    ],
    [
      "CCRD:90",
      "OMIM:261600",
      "ORPHA:716"
    ]
  ],
  [
    [
      "HP:0001522",
      "HP:0001942",
      "HP:0003210"
    ],
    [
      "CCRD:71",
      "OMIM:251000",
      "ORPHA:27"
    ]
  ],
  [
    [
      "HP:0000750",
      "HP:0001249",
      "HP:0001270",
```

```
      "HP:0001522",
      "HP:0003231",
      "HP:0003510",
      "HP:0003819",
      "HP:0004923"
    ],
    [
      "CCRD:90",
      "OMIM:261600",
      "ORPHA:716"
    ]
  ],
  [
    [
      "HP:0002355",
      "HP:0003326",
      "HP:0003551",
      "HP:0003752",
      "HP:0003819",
      "HP:0009046",
      "HP:0010899",
      "HP:0010967",
      "HP:0040081",
      "HP:0200072"
    ],
    [
      "OMIM:255110",
      "ORPHA:228302"
    ]
  ],
  [
    [
      "HP:0001197",
      "HP:0001522",
      "HP:0003811"
    ],
    [
      "CCRD:13",
      "OMIM:253260",
      "ORPHA:79241"
    ]
  ],
  [
    [
      "HP:0000028",
      "HP:0000047",
      "HP:0001249",
      "HP:0001250",
      "HP:0001270",
      "HP:0001290",
      "HP:0001298",
      "HP:0001508",
      "HP:0001513",
      "HP:0001522",
      "HP:0001882",
      "HP:0001974",
```

```
      "HP:0001987",
      "HP:0002021",
      "HP:0002093",
      "HP:0002154",
      "HP:0003811",
      "HP:0003819",
      "HP:0010899",
      "HP:0010916",
      "HP:0010967",
      "HP:0011421",
      "HP:0011968",
      "HP:0100806"
    ],
    [
      "CCRD:99",
      "OMIM:606054",
      "ORPHA:35"
    ]
  ],
  [
    [
      "HP:0001959",
      "HP:0002910",
      "HP:0010837",
      "HP:0011421",
      "HP:0011967"
    ],
    [
      "CCRD:37",
      "OMIM:277900",
      "ORPHA:905"
    ]
  ],
  [
    [
      "HP:0001513",
      "HP:0001760",
      "HP:0001987",
      "HP:0003819"
    ],
    [
      "CCRD:13",
      "OMIM:253260",
      "ORPHA:79241"
    ]
  ],
  [
    [
      "HP:0000976",
      "HP:0000988",
      "HP:0001197",
      "HP:0001290",
      "HP:0001522",
      "HP:0002151",
      "HP:0003811",
      "HP:0003819"
```

```
    ],
    [
      "CCRD:13",
      "OMIM:253260",
      "ORPHA:79241"
    ]
  ],
  [
    [
      "HP:0000316",
      "HP:0000463",
      "HP:0000742",
      "HP:0000750",
      "HP:0000752",
      "HP:0001159",
      "HP:0001249",
      "HP:0001250",
      "HP:0001270",
      "HP:0001522",
      "HP:0001760",
      "HP:0003146",
      "HP:0003462",
      "HP:0003811",
      "HP:0003819",
      "HP:0010569",
      "HP:0012714"
    ],
    [
      "OMIM:270400",
      "ORPHA:818"
    ]
  ],
  [
    [
      "HP:0000741",
      "HP:0001522",
      "HP:0001942",
      "HP:0001943",
      "HP:0001987",
      "HP:0002013",
      "HP:0002090",
      "HP:0002240",
      "HP:0003215",
      "HP:0003535",
      "HP:0008315",
      "HP:0010895",
      "HP:0012071",
      "HP:0040156"
    ],
    [
      "OMIM:246450",
      "ORPHA:20"
    ]
  ],
  [
    [
```

```
      "HP:0001197",
      "HP:0001522",
      "HP:0003811",
      "HP:0004923"
    ],
    [
      "CCRD:90",
      "OMIM:261600",
      "ORPHA:716"
    ]
  ],
  [
    [
      "HP:0001290",
      "HP:0001744",
      "HP:0002240",
      "HP:0002910",
      "HP:0003819",
      "HP:0010837",
      "HP:0010839",
      "HP:0010899",
      "HP:0010916",
      "HP:0011421",
      "HP:0011967",
      "HP:0025321"
    ],
    [
      "CCRD:37",
      "OMIM:277900",
      "ORPHA:905"
    ]
  ],
  [
    [
      "HP:0001197",
      "HP:0001522",
      "HP:0003811",
      "HP:0004923"
    ],
    [
      "CCRD:90",
      "OMIM:261600",
      "ORPHA:716"
    ]
  ],
  [
    [
      "HP:0001249",
      "HP:0001270",
      "HP:0001290",
      "HP:0001522",
      "HP:0001987",
      "HP:0002013",
      "HP:0003075",
      "HP:0003138",
      "HP:0003354",
```

"HP:0003811",
"HP:0003819",
"HP:0004429",
"HP:0010910",
"HP:0010912",
"HP:0010967",
"HP:0011968",
"HP:0012101",
"HP:0012120"
],
[
"CCRD:71",
"OMIM:251000",
"ORPHA:27"
]
],
[
[
"HP:0000967",
"HP:0001000",
"HP:0001522",
"HP:0001873",
"HP:0001943",
"HP:0001987",
"HP:0003155",
"HP:0003161",
"HP:0003231",
"HP:0003235",
"HP:0003645",
"HP:0003819",
"HP:0004923",
"HP:0006254",
"HP:0007832",
"HP:0008064",
"HP:0010472"
],
[
"CCRD:115.1",
"OMIM:276700",
"ORPHA:882"
]
],
[
[
"HP:0001197",
"HP:0001522",
"HP:0001943",
"HP:0002148",
"HP:0002240",
"HP:0002900",
"HP:0003010",
"HP:0003161",
"HP:0003231",
"HP:0003811",
"HP:0003819",
"HP:0004923",

```
      "HP:0006254"
    ],
    [
      "CCRD:115.1",
      "OMIM:276700",
      "ORPHA:882"
    ]
  ],
  [
    [
      "HP:0000256",
      "HP:0000737",
      "HP:0000975",
      "HP:0001254",
      "HP:0001290",
      "HP:0001332",
      "HP:0001522",
      "HP:0001944",
      "HP:0001945",
      "HP:0002013",
      "HP:0002014",
      "HP:0002329",
      "HP:0002360",
      "HP:0003150",
      "HP:0003530",
      "HP:0003811",
      "HP:0003819",
      "HP:0008315",
      "HP:0011968",
      "HP:0100660"
    ],
    [
      "CCRD:34.1",
      "OMIM:231670",
      "ORPHA:25"
    ]
  ],
  [
    [
      "HP:0001522",
      "HP:0002013",
      "HP:0003573",
      "HP:0003811",
      "HP:0012024"
    ],
    [
      "CCRD:30",
      "OMIM:230400",
      "ORPHA:352",
      "ORPHA:79239"
    ]
  ],
  [
    [
      "HP:0000175",
      "HP:0000252",
```

```
      "HP:0000286",
      "HP:0000369",
      "HP:0000463",
      "HP:0000486",
      "HP:0000508",
      "HP:0000639",
      "HP:0000750",
      "HP:0001052",
      "HP:0001159",
      "HP:0001249",
      "HP:0001254",
      "HP:0001270",
      "HP:0001290",
      "HP:0001508",
      "HP:0001522",
      "HP:0002305",
      "HP:0002329",
      "HP:0002360",
      "HP:0003124",
      "HP:0003146",
      "HP:0003462",
      "HP:0003811",
      "HP:0003819",
      "HP:0010569",
      "HP:0011968"
    ],
    [
      "OMIM:270400",
      "ORPHA:818"
    ]
  ],
  [
    [
      "HP:0002013",
      "HP:0002076",
      "HP:0003217",
      "HP:0003218",
      "HP:0003348",
      "HP:0003572",
      "HP:0003819",
      "HP:0011421",
      "HP:0012127",
      "HP:0012343",
      "HP:0040126"
    ],
    [
      "CCRD:85",
      "OMIM:311250",
      "ORPHA:664"
    ]
  ],
  [
    [
      "HP:0001250",
      "HP:0001259",
      "HP:0001397",
```

```
      "HP:0001522",
      "HP:0001943",
      "HP:0001987",
      "HP:0002013",
      "HP:0002239",
      "HP:0002900",
      "HP:0002910",
      "HP:0003215",
      "HP:0003344",
      "HP:0010472",
      "HP:0010895",
      "HP:0010899",
      "HP:0010916"
    ],
    [
      "OMIM:246450",
      "ORPHA:20"
    ]
  ],
  [
    [
      "HP:0000252",
      "HP:0000742",
      "HP:0000750",
      "HP:0001249",
      "HP:0001250",
      "HP:0001522",
      "HP:0003231",
      "HP:0003510",
      "HP:0004923",
      "HP:0011421"
    ],
    [
      "CCRD:90",
      "OMIM:261600",
      "ORPHA:716"
    ]
  ],
  [
    [
      "HP:0001264",
      "HP:0001522",
      "HP:0002120",
      "HP:0002179",
      "HP:0002305",
      "HP:0002510",
      "HP:0003150",
      "HP:0003530",
      "HP:0008315"
    ],
    [
      "CCRD:34.1",
      "OMIM:231670",
      "ORPHA:25"
    ]
  ],
```

```
[
  [
    "HP:0001197",
    "HP:0001522",
    "HP:0003811",
    "HP:0004923"
  ],
  [
    "CCRD:90",
    "OMIM:261600",
    "ORPHA:716"
  ]
],
[
  [
    "HP:0001250",
    "HP:0001522",
    "HP:0003355",
    "HP:0003811",
    "HP:0004357",
    "HP:0010912",
    "HP:0010914"
  ],
  [
    "CCRD:67",
    "OMIM:248600",
    "ORPHA:511"
  ]
],
[
  [
    "HP:0000256",
    "HP:0001250",
    "HP:0001522",
    "HP:0002179",
    "HP:0003150",
    "HP:0003530",
    "HP:0003811"
  ],
  [
    "CCRD:34.1",
    "OMIM:231670",
    "ORPHA:25"
  ]
],
[
  [
    "HP:0001254",
    "HP:0001259",
    "HP:0001290",
    "HP:0001522",
    "HP:0001873",
    "HP:0001903",
    "HP:0002013",
    "HP:0002094",
    "HP:0002329",
```

"HP:0002360",
"HP:0002901",
"HP:0003811",
"HP:0004360",
"HP:0010472",
"HP:0010895",
"HP:0011968",
"HP:0040156"
],
[
"CCRD:99",
"OMIM:606054",
"ORPHA:35"
]
],
[
[
"HP:0000709",
"HP:0000939",
"HP:0001249",
"HP:0001903",
"HP:0001987",
"HP:0002059",
"HP:0002154",
"HP:0002751",
"HP:0003138",
"HP:0003259",
"HP:0003348",
"HP:0003510",
"HP:0010472",
"HP:0010967",
"HP:0011421",
"HP:0012714",
"HP:0040156",
"HP:0100613",
"HP:0500001"
],
[
"CCRD:99",
"OMIM:606054",
"ORPHA:35"
]
],
[
[
"HP:0001197",
"HP:0001522",
"HP:0003811",
"HP:0004923",
"HP:0011421"
],
[
"CCRD:90",
"OMIM:261600",
"ORPHA:716"
]

```
  ],
  [
    [
      "HP:0000369",
      "HP:0000431",
      "HP:0000463",
      "HP:0000508",
      "HP:0001159",
      "HP:0001249",
      "HP:0001270",
      "HP:0001290",
      "HP:0001522",
      "HP:0003146",
      "HP:0003462",
      "HP:0003811",
      "HP:0010569",
      "HP:0011968"
    ],
    [
      "OMIM:270400",
      "ORPHA:818"
    ]
  ],
  [
    [
      "HP:0000010",
      "HP:0001522",
      "HP:0003131",
      "HP:0003268",
      "HP:0003297",
      "HP:0003532"
    ],
    [
      "OMIM:220100",
      "ORPHA:214"
    ]
  ],
  [
    [
      "HP:0000618",
      "HP:0000639",
      "HP:0001250",
      "HP:0001270",
      "HP:0001290",
      "HP:0001522",
      "HP:0002179",
      "HP:0003811",
      "HP:0010899",
      "HP:0012447",
      "HP:0012714"
    ],
    [
      "OMIM:271900",
      "ORPHA:141"
    ]
  ],
```

```
[
  [
    "HP:0001250",
    "HP:0001254",
    "HP:0001510",
    "HP:0001522",
    "HP:0001942",
    "HP:0001987",
    "HP:0002329",
    "HP:0002360",
    "HP:0010910",
    "HP:0010911",
    "HP:0010913",
    "HP:0011968",
    "HP:0012714",
    "HP:0040156"
  ],
  [
    "CCRD:67",
    "OMIM:248600",
    "ORPHA:511"
  ]
],
[
  [
    "HP:0000750",
    "HP:0000752",
    "HP:0001250",
    "HP:0001251",
    "HP:0001270",
    "HP:0001290",
    "HP:0001522",
    "HP:0003819",
    "HP:0040156"
  ],
  [
    "OMIM:271980",
    "ORPHA:22"
  ]
],
[
  [
    "HP:0001522",
    "HP:0003811",
    "HP:0003819",
    "HP:0004386",
    "HP:0004429",
    "HP:0010910",
    "HP:0010911",
    "HP:0010913",
    "HP:0011421",
    "HP:0100613",
    "HP:0200039"
  ],
  [
    "CCRD:67",
```

```
      "OMIM:248600",
      "ORPHA:511"
    ]
  ],
  [
    [
      "HP:0001254",
      "HP:0001290",
      "HP:0001522",
      "HP:0001987",
      "HP:0002329",
      "HP:0002360",
      "HP:0003811",
      "HP:0010910",
      "HP:0010911",
      "HP:0010918",
      "HP:0100806",
      "HP:0200114"
    ],
    [
      "CCRD:67",
      "OMIM:248600",
      "ORPHA:511"
    ]
  ],
  [
    [
      "HP:0000010",
      "HP:0000123",
      "HP:0000790",
      "HP:0001522",
      "HP:0003131",
      "HP:0003268",
      "HP:0003297",
      "HP:0003532"
    ],
    [
      "OMIM:220100",
      "ORPHA:214"
    ]
  ],
  [
    [
      "HP:0000028",
      "HP:0000047",
      "HP:0000252",
      "HP:0000369",
      "HP:0000486",
      "HP:0000508",
      "HP:0000540",
      "HP:0000976",
      "HP:0000988",
      "HP:0001159",
      "HP:0001249",
      "HP:0001254",
      "HP:0001270",
```

```
      "HP:0001290",
      "HP:0001332",
      "HP:0001522",
      "HP:0002013",
      "HP:0002019",
      "HP:0002329",
      "HP:0002360",
      "HP:0003146",
      "HP:0003462",
      "HP:0003811",
      "HP:0003819",
      "HP:0004429",
      "HP:0010569",
      "HP:0011968",
      "HP:0012418",
      "HP:0012714"
    ],
    [
      "OMIM:270400",
      "ORPHA:818"
    ]
  ],
  [
    [
      "HP:0000670",
      "HP:0000750",
      "HP:0001249",
      "HP:0001250",
      "HP:0001264",
      "HP:0001270",
      "HP:0001290",
      "HP:0001522",
      "HP:0001987",
      "HP:0002013",
      "HP:0002014",
      "HP:0002093",
      "HP:0002355",
      "HP:0002510",
      "HP:0003551",
      "HP:0003781",
      "HP:0003811",
      "HP:0003819",
      "HP:0004429",
      "HP:0009046",
      "HP:0010910",
      "HP:0010911",
      "HP:0010913",
      "HP:0011421",
      "HP:0100613"
    ],
    [
      "CCRD:67",
      "OMIM:248600",
      "ORPHA:511"
    ]
  ],
```

[
  [
    "HP:0000750",
    "HP:0000976",
    "HP:0000988",
    "HP:0001522",
    "HP:0001882",
    "HP:0001987",
    "HP:0002154",
    "HP:0003108",
    "HP:0003155",
    "HP:0003819",
    "HP:0010899",
    "HP:0010916",
    "HP:0010967",
    "HP:0011421",
    "HP:0012278",
    "HP:0040156"
  ],
  [
    "CCRD:99",
    "OMIM:606054",
    "ORPHA:35"
  ]
],
[
  [
    "HP:0001250",
    "HP:0001259",
    "HP:0001945",
    "HP:0002013",
    "HP:0002789",
    "HP:0003819",
    "HP:0010910",
    "HP:0010913",
    "HP:0011968"
  ],
  [
    "CCRD:67",
    "OMIM:248600",
    "ORPHA:511"
  ]
],
[
  [
    "HP:0001197",
    "HP:0001522",
    "HP:0003811",
    "HP:0004923"
  ],
  [
    "CCRD:90",
    "OMIM:261600",
    "ORPHA:716"
  ]
],

```
[
  [
    "HP:0001197",
    "HP:0001249",
    "HP:0001250",
    "HP:0001270",
    "HP:0001522",
    "HP:0001903",
    "HP:0001987",
    "HP:0002154",
    "HP:0002240",
    "HP:0003811",
    "HP:0003819",
    "HP:0010967",
    "HP:0012277"
  ],
  [
    "CCRD:99",
    "OMIM:606054",
    "ORPHA:35"
  ]
],
[
  [
    "HP:0000256",
    "HP:0000975",
    "HP:0001254",
    "HP:0001290",
    "HP:0001332",
    "HP:0001522",
    "HP:0001945",
    "HP:0002013",
    "HP:0002014",
    "HP:0002094",
    "HP:0002329",
    "HP:0002360",
    "HP:0003150",
    "HP:0100660"
  ],
  [
    "CCRD:34.1",
    "OMIM:231670",
    "ORPHA:25"
  ]
],
[
  [
    "HP:0000741",
    "HP:0000961",
    "HP:0001290",
    "HP:0001522",
    "HP:0001662",
    "HP:0001943",
    "HP:0001987",
    "HP:0003215",
    "HP:0003811"
```

```
      ],
      [
        "OMIM:212138",
        "ORPHA:159"
      ]
    ],
    [
      [
        "HP:0000256",
        "HP:0001254",
        "HP:0001290",
        "HP:0001332",
        "HP:0001522",
        "HP:0001945",
        "HP:0002013",
        "HP:0002014",
        "HP:0002094",
        "HP:0002305",
        "HP:0002329",
        "HP:0002360",
        "HP:0003150",
        "HP:0003530",
        "HP:0003811",
        "HP:0003819",
        "HP:0008315",
        "HP:0100660"
      ],
      [
        "CCRD:34.1",
        "OMIM:231670",
        "ORPHA:25"
      ]
    ],
    [
      [
        "HP:0000126",
        "HP:0000750",
        "HP:0001197",
        "HP:0001249",
        "HP:0001270",
        "HP:0001290",
        "HP:0001522",
        "HP:0001945",
        "HP:0002179",
        "HP:0002305",
        "HP:0002751",
        "HP:0003811",
        "HP:0003819",
        "HP:0010910",
        "HP:0010911",
        "HP:0010913",
        "HP:0011421",
        "HP:0011968",
        "HP:0040156"
      ],
      [
```

```
      "CCRD:67",
      "OMIM:248600",
      "ORPHA:511"
    ]
  ],
  [
    [
      "HP:0001254",
      "HP:0001522",
      "HP:0001942",
      "HP:0001943",
      "HP:0001987",
      "HP:0002013",
      "HP:0002014",
      "HP:0002240",
      "HP:0002329",
      "HP:0002360",
      "HP:0003215",
      "HP:0003344",
      "HP:0003819",
      "HP:0004429",
      "HP:0040156"
    ],
    [
      "OMIM:246450",
      "ORPHA:20"
    ]
  ],
  [
    [
      "HP:0001254",
      "HP:0001290",
      "HP:0001337",
      "HP:0001522",
      "HP:0002179",
      "HP:0002329",
      "HP:0002360",
      "HP:0003811",
      "HP:0004357",
      "HP:0040156",
      "HP:0500001"
    ],
    [
      "CCRD:67",
      "OMIM:248600",
      "ORPHA:511"
    ]
  ],
  [
    [
      "HP:0001943",
      "HP:0003819",
      "HP:0008315",
      "HP:0200119",
      "HP:0200123"
    ],
```

```
      [
        "CCRD:15",
        "OMIM:212140",
        "ORPHA:158"
      ]
    ],
    [
      [
        "HP:0000252",
        "HP:0000486",
        "HP:0000639",
        "HP:0000648",
        "HP:0000742",
        "HP:0001141",
        "HP:0001249",
        "HP:0001250",
        "HP:0001254",
        "HP:0001270",
        "HP:0001396",
        "HP:0001508",
        "HP:0001522",
        "HP:0001629",
        "HP:0001873",
        "HP:0001875",
        "HP:0001882",
        "HP:0001903",
        "HP:0001987",
        "HP:0002098",
        "HP:0002160",
        "HP:0002240",
        "HP:0002329",
        "HP:0002360",
        "HP:0002813",
        "HP:0003235",
        "HP:0003658",
        "HP:0003811",
        "HP:0003819",
        "HP:0004360",
        "HP:0008315",
        "HP:0010995",
        "HP:0012120",
        "HP:0100660"
      ],
      [
        "CCRD:71",
        "OMIM:277400",
        "ORPHA:79282"
      ]
    ],
    [
      [
        "HP:0001249",
        "HP:0001270",
        "HP:0001999",
        "HP:0002648",
        "HP:0003819",
```

```
        "HP:0010996",
        "HP:0040156"
      ],
      [
        "OMIM:271980",
        "ORPHA:22"
      ]
    ],
    [
      [
        "HP:0001197",
        "HP:0001249",
        "HP:0001522",
        "HP:0003811",
        "HP:0003819",
        "HP:0004923",
        "HP:0011421"
      ],
      [
        "CCRD:90",
        "OMIM:261600",
        "ORPHA:716"
      ]
    ],
    [
      [
        "HP:0001250",
        "HP:0001290",
        "HP:0001522",
        "HP:0001873",
        "HP:0001882",
        "HP:0001942",
        "HP:0001987",
        "HP:0002013",
        "HP:0002912",
        "HP:0003811",
        "HP:0012120"
      ],
      [
        "CCRD:71",
        "OMIM:251000",
        "ORPHA:27"
      ]
    ],
    [
      [
        "HP:0001290",
        "HP:0001522",
        "HP:0002305",
        "HP:0003150",
        "HP:0003530",
        "HP:0004429",
        "HP:0011968"
      ],
      [
        "CCRD:34.1",
```

```
      "OMIM:231670",
      "ORPHA:25"
    ]
  ],
  [
    [
      "HP:0001197",
      "HP:0001522",
      "HP:0003819",
      "HP:0004923"
    ],
    [
      "CCRD:90",
      "OMIM:261600",
      "ORPHA:716"
    ]
  ],
  [
    [
      "HP:0001259",
      "HP:0001397",
      "HP:0001522",
      "HP:0002013",
      "HP:0002181",
      "HP:0012071"
    ],
    [
      "CCRD:70",
      "OMIM:201450",
      "ORPHA:42"
    ]
  ],
  [
    [
      "HP:0001250",
      "HP:0001522",
      "HP:0001629",
      "HP:0001631",
      "HP:0001903",
      "HP:0001943",
      "HP:0002240",
      "HP:0002789",
      "HP:0003215",
      "HP:0003535",
      "HP:0003811",
      "HP:0011968",
      "HP:0040156"
    ],
    [
      "OMIM:246450",
      "ORPHA:20"
    ]
  ],
  [
    [
      "HP:0000175",
```

"HP:0000289",
"HP:0000343",
"HP:0000431",
"HP:0000463",
"HP:0000501",
"HP:0000518",
"HP:0000811",
"HP:0001159",
"HP:0001290",
"HP:0001522",
"HP:0002002",
"HP:0002013",
"HP:0002021",
"HP:0002705",
"HP:0003146",
"HP:0003270",
"HP:0003462",
"HP:0003811",
"HP:0010569",
"HP:0011968"
],
[
"OMIM:270400",
"ORPHA:818"
]
],
[
[
"HP:0001197",
"HP:0001522",
"HP:0003811",
"HP:0004923"
],
[
"CCRD:90",
"OMIM:261600",
"ORPHA:716"
]
],
[
[
"HP:0001197",
"HP:0001522",
"HP:0003811",
"HP:0004923",
"HP:0100613"
],
[
"CCRD:90",
"OMIM:261600",
"ORPHA:716"
]
],
[
[
"HP:0000252",

```
      "HP:0000369",
      "HP:0000431",
      "HP:0000486",
      "HP:0000750",
      "HP:0001159",
      "HP:0001249",
      "HP:0001270",
      "HP:0001522",
      "HP:0002355",
      "HP:0003462",
      "HP:0003510",
      "HP:0003551",
      "HP:0003811",
      "HP:0003819",
      "HP:0009046",
      "HP:0010569",
      "HP:0012714"
    ],
    [
      "OMIM:270400",
      "ORPHA:818"
    ]
  ],
  [
    [
      "HP:0001290",
      "HP:0001522",
      "HP:0001945",
      "HP:0002060",
      "HP:0003150",
      "HP:0008315",
      "HP:0011220",
      "HP:0011968"
    ],
    [
      "CCRD:34.1",
      "OMIM:231670",
      "ORPHA:25"
    ]
  ],
  [
    [
      "HP:0001257",
      "HP:0001265",
      "HP:0001290",
      "HP:0001522",
      "HP:0003811",
      "HP:0040081",
      "HP:0040301"
    ],
    [
      "OMIM:307030",
      "ORPHA:408"
    ]
  ],
  [
```

[
"HP:0001250",
"HP:0001254",
"HP:0001284",
"HP:0001290",
"HP:0001332",
"HP:0001522",
"HP:0001945",
"HP:0002094",
"HP:0002154",
"HP:0002179",
"HP:0002329",
"HP:0002360",
"HP:0002380",
"HP:0003150",
"HP:0003217",
"HP:0003530",
"HP:0010967",
"HP:0011968"
],
[
"CCRD:34.1",
"OMIM:231670",
"ORPHA:25"
]
],
[
[
"HP:0001249",
"HP:0001250",
"HP:0001251",
"HP:0001337",
"HP:0003819",
"HP:0040144"
],
[
"OMIM:236792",
"ORPHA:79314"
]
],
[
[
"HP:0000238",
"HP:0000252",
"HP:0000486",
"HP:0000639",
"HP:0001249",
"HP:0001522",
"HP:0001622",
"HP:0002093",
"HP:0002160",
"HP:0003811",
"HP:0003819",
"HP:0010967",
"HP:0012120",
"HP:0100660"

```
    ],
    [
      "CCRD:71",
      "OMIM:277400",
      "ORPHA:79282"
    ]
  ],
  [
    [
      "HP:0000252",
      "HP:0000819",
      "HP:0001249",
      "HP:0001250",
      "HP:0001522",
      "HP:0002500",
      "HP:0003231",
      "HP:0004923",
      "HP:0100613",
      "HP:0500001"
    ],
    [
      "CCRD:90",
      "OMIM:261600",
      "ORPHA:716"
    ]
  ],
  [
    [
      "HP:0000252",
      "HP:0000954",
      "HP:0000980",
      "HP:0001197",
      "HP:0001270",
      "HP:0001508",
      "HP:0001518",
      "HP:0001522",
      "HP:0001903",
      "HP:0001942",
      "HP:0001943",
      "HP:0001987",
      "HP:0002013",
      "HP:0002090",
      "HP:0002151",
      "HP:0002490",
      "HP:0003146",
      "HP:0003210",
      "HP:0003811",
      "HP:0010472",
      "HP:0010967",
      "HP:0011968",
      "HP:0012120"
    ],
    [
      "CCRD:71",
      "OMIM:251000",
      "ORPHA:27"
```

    ]
  ],
  [
    [
      "HP:0000737",
      "HP:0001250",
      "HP:0001290",
      "HP:0001522",
      "HP:0001942",
      "HP:0002179",
      "HP:0003215",
      "HP:0003535",
      "HP:0003811",
      "HP:0003819",
      "HP:0040156"
    ],
    [
      "OMIM:246450",
      "ORPHA:20"
    ]
  ],
  [
    [
      "HP:0001397",
      "HP:0002910",
      "HP:0003819",
      "HP:0004923",
      "HP:0010837",
      "HP:0010839",
      "HP:0010899",
      "HP:0010916",
      "HP:0011967"
    ],
    [
      "CCRD:37",
      "OMIM:277900",
      "ORPHA:905"
    ]
  ],
  [
    [
      "HP:0000486",
      "HP:0001249",
      "HP:0001251",
      "HP:0001270",
      "HP:0001272",
      "HP:0001290",
      "HP:0001394",
      "HP:0001395",
      "HP:0001522",
      "HP:0001907",
      "HP:0001976",
      "HP:0003819",
      "HP:0009125",
      "HP:0011421",
      "HP:0012642"

```
    ],
    [
      "OMIM:212065",
      "ORPHA:79318"
    ]
  ],
  [
    [
      "HP:0001270",
      "HP:0001290",
      "HP:0001522",
      "HP:0002090",
      "HP:0040156"
    ],
    [
      "OMIM:271980",
      "ORPHA:22"
    ]
  ],
  [
    [
      "HP:0000750",
      "HP:0000752",
      "HP:0001249",
      "HP:0003819",
      "HP:0011421",
      "HP:0040156"
    ],
    [
      "OMIM:271980",
      "ORPHA:22"
    ]
  ],
  [
    [
      "HP:0000508",
      "HP:0001083",
      "HP:0001176",
      "HP:0001249",
      "HP:0001270",
      "HP:0001833",
      "HP:0002160",
      "HP:0003235",
      "HP:0003819",
      "HP:0011421",
      "HP:0012378"
    ],
    [
      "CCRD:45",
      "OMIM:236200",
      "ORPHA:394"
    ]
  ],
  [
    [
      "HP:0000545",
```

```
      "HP:0000662",
      "HP:0001987",
      "HP:0003819",
      "HP:0011421",
      "HP:0012026"
    ],
    [
      "OMIM:258870",
      "ORPHA:414"
    ]
  ],
  [
    [
      "HP:0001250",
      "HP:0001254",
      "HP:0001522",
      "HP:0001942",
      "HP:0001943",
      "HP:0001945",
      "HP:0002013",
      "HP:0002329",
      "HP:0002344",
      "HP:0002360",
      "HP:0003150",
      "HP:0003344",
      "HP:0003819",
      "HP:0004429",
      "HP:0010895",
      "HP:0040156"
    ],
    [
      "OMIM:246450",
      "ORPHA:20"
    ]
  ],
  [
    [
      "HP:0001522",
      "HP:0001987",
      "HP:0002013",
      "HP:0002154",
      "HP:0003108",
      "HP:0003348",
      "HP:0003819",
      "HP:0008064",
      "HP:0010903",
      "HP:0010967",
      "HP:0040156"
    ],
    [
      "CCRD:99",
      "OMIM:606054",
      "ORPHA:35"
    ]
  ],
  [
```

    [
      "HP:0000369",
      "HP:0000463",
      "HP:0000508",
      "HP:0000750",
      "HP:0000752",
      "HP:0001159",
      "HP:0001249",
      "HP:0001270",
      "HP:0001522",
      "HP:0002705",
      "HP:0003146",
      "HP:0003462",
      "HP:0003811",
      "HP:0003819",
      "HP:0010442",
      "HP:0010569"
    ],
    [
      "OMIM:270400",
      "ORPHA:818"
    ]
  ],
  [
    [
      "HP:0000750",
      "HP:0001270",
      "HP:0001290",
      "HP:0001522",
      "HP:0001944",
      "HP:0001945",
      "HP:0002013",
      "HP:0002098",
      "HP:0002912",
      "HP:0003108",
      "HP:0003124",
      "HP:0003348",
      "HP:0003819",
      "HP:0010472",
      "HP:0010967",
      "HP:0012120"
    ],
    [
      "CCRD:71",
      "OMIM:251100",
      "ORPHA:79310"
    ]
  ],
  [
    [
      "HP:0001197",
      "HP:0001522",
      "HP:0003811",
      "HP:0004923"
    ],
    [

```
      "CCRD:90",
      "OMIM:261600",
      "ORPHA:716"
    ]
  ],
  [
    [
      "HP:0000256",
      "HP:0000750",
      "HP:0001249",
      "HP:0001250",
      "HP:0001522",
      "HP:0001945",
      "HP:0002060",
      "HP:0012714",
      "HP:0040144",
      "HP:0040147"
    ],
    [
      "OMIM:236792",
      "ORPHA:79314"
    ]
  ],
  [
    [
      "HP:0001249",
      "HP:0001250",
      "HP:0001290",
      "HP:0001522",
      "HP:0001662",
      "HP:0002104",
      "HP:0002179",
      "HP:0003811",
      "HP:0004429",
      "HP:0010910",
      "HP:0010911",
      "HP:0010913",
      "HP:0012378",
      "HP:0500001"
    ],
    [
      "CCRD:67",
      "OMIM:248600",
      "ORPHA:511"
    ]
  ],
  [
    [
      "HP:0001249",
      "HP:0001250",
      "HP:0001264",
      "HP:0001270",
      "HP:0001332",
      "HP:0001522",
      "HP:0002305",
      "HP:0002510",
```

"HP:0003150",
"HP:0003510",
"HP:0010551",
"HP:0011421"
],
[
"CCRD:34.1",
"OMIM:231670",
"ORPHA:25"
]
],
[
[
"HP:0001250",
"HP:0001522",
"HP:0001943",
"HP:0002240",
"HP:0002910",
"HP:0003215",
"HP:0003344",
"HP:0004360",
"HP:0008315",
"HP:0040156"
],
[
"OMIM:246450",
"ORPHA:20"
]
],
[
[
"HP:0001250",
"HP:0001264",
"HP:0001270",
"HP:0001290",
"HP:0001522",
"HP:0001942",
"HP:0002094",
"HP:0002104",
"HP:0002179",
"HP:0002510",
"HP:0003811",
"HP:0010910",
"HP:0010911",
"HP:0010913",
"HP:0011968",
"HP:0500001"
],
[
"CCRD:67",
"OMIM:248600",
"ORPHA:511"
]
],
[
[

"HP:0000618",
"HP:0000939",
"HP:0001083",
"HP:0001638",
"HP:0001677",
"HP:0001760",
"HP:0002090",
"HP:0002160",
"HP:0003235",
"HP:0003348",
"HP:0003468",
"HP:0011421",
"HP:0100613"
],
[
"CCRD:45",
"OMIM:236200",
"ORPHA:394"
]
],
[
[
"HP:0000252",
"HP:0000750",
"HP:0001197",
"HP:0001249",
"HP:0001270",
"HP:0001290",
"HP:0001508",
"HP:0001518",
"HP:0001522",
"HP:0001744",
"HP:0001903",
"HP:0001919",
"HP:0001942",
"HP:0001943",
"HP:0001944",
"HP:0001945",
"HP:0001987",
"HP:0002045",
"HP:0002090",
"HP:0002149",
"HP:0002151",
"HP:0002154",
"HP:0002240",
"HP:0002912",
"HP:0003108",
"HP:0003138",
"HP:0003155",
"HP:0003210",
"HP:0003259",
"HP:0003510",
"HP:0003811",
"HP:0003819",
"HP:0004386",
"HP:0008315",

```
      "HP:0010472",
      "HP:0010899",
      "HP:0010916",
      "HP:0010919",
      "HP:0011227",
      "HP:0011968",
      "HP:0012120",
      "HP:0012622",
      "HP:0040156"
    ],
    [
      "CCRD:71",
      "OMIM:251000",
      "ORPHA:27"
    ]
  ],
  [
    [
      "HP:0000750",
      "HP:0003161",
      "HP:0003163",
      "HP:0003231",
      "HP:0003819",
      "HP:0010893"
    ],
    [
      "CCRD:115.1",
      "OMIM:276700",
      "ORPHA:882"
    ]
  ],
  [
    [
      "HP:0001251",
      "HP:0001259",
      "HP:0001315",
      "HP:0001596",
      "HP:0001942",
      "HP:0002181",
      "HP:0002500",
      "HP:0003819",
      "HP:0004429",
      "HP:0010911",
      "HP:0011421"
    ],
    [
      "CCRD:67",
      "OMIM:248600",
      "ORPHA:511"
    ]
  ],
  [
    [
      "HP:0000091",
      "HP:0000105",
      "HP:0000123",
```

```
      "HP:0000790",
      "HP:0000822",
      "HP:0001250",
      "HP:0001394",
      "HP:0001395",
      "HP:0001522",
      "HP:0001959",
      "HP:0002013",
      "HP:0002039",
      "HP:0002240",
      "HP:0003161",
      "HP:0003270",
      "HP:0003355",
      "HP:0003819",
      "HP:0006254",
      "HP:0010917",
      "HP:0031500"
    ],
    [
      "CCRD:115.1",
      "OMIM:276700",
      "ORPHA:882"
    ]
  ],
  [
    [
      "HP:0001197",
      "HP:0001522",
      "HP:0003811",
      "HP:0004923"
    ],
    [
      "CCRD:90",
      "OMIM:261600",
      "ORPHA:716"
    ]
  ],
  [
    [
      "HP:0000750",
      "HP:0000752",
      "HP:0001249",
      "HP:0001250",
      "HP:0001290",
      "HP:0003819",
      "HP:0008315",
      "HP:0010996",
      "HP:0040156"
    ],
    [
      "OMIM:271980",
      "ORPHA:22"
    ]
  ],
  [
    [
```

"HP:0000252",
"HP:0000463",
"HP:0000486",
"HP:0000598",
"HP:0001159",
"HP:0001251",
"HP:0001259",
"HP:0001270",
"HP:0001522",
"HP:0001943",
"HP:0003146",
"HP:0003462",
"HP:0003811",
"HP:0003819",
"HP:0004429",
"HP:0010569"
],
[
"OMIM:270400",
"ORPHA:818"
]
],
[
[
"HP:0001249",
"HP:0001290",
"HP:0001347",
"HP:0001987",
"HP:0002060",
"HP:0003819",
"HP:0012447",
"HP:0040147"
],
[
"OMIM:236792",
"ORPHA:79314"
]
],
[
[
"HP:0000737",
"HP:0001259",
"HP:0001290",
"HP:0001298",
"HP:0001638",
"HP:0001903",
"HP:0001943",
"HP:0002013",
"HP:0002240",
"HP:0002910",
"HP:0003819",
"HP:0008315",
"HP:0010472",
"HP:0010899",
"HP:0010916"
],

[
"CCRD:15",
"OMIM:212140",
"ORPHA:158"
]
],
[
[
"HP:0000726",
"HP:0000988",
"HP:0001257",
"HP:0001284",
"HP:0001522",
"HP:0001945",
"HP:0001987",
"HP:0002014",
"HP:0002181",
"HP:0002240",
"HP:0002344",
"HP:0003297",
"HP:0005961",
"HP:0010901",
"HP:0010903",
"HP:0010916",
"HP:0011966",
"HP:0025474",
"HP:0025475",
"HP:0030350"
],
[
"CCRD:18",
"OMIM:215700",
"ORPHA:187",
"ORPHA:247525"
]
],
[
[
"HP:0000976",
"HP:0000988",
"HP:0001522",
"HP:0003131",
"HP:0003268",
"HP:0003297",
"HP:0003532"
],
[
"OMIM:220100",
"ORPHA:214"
]
],
[
[
"HP:0001522",
"HP:0001695",
"HP:0003150"

```
    ],
    [
      "CCRD:34.1",
      "OMIM:231670",
      "ORPHA:25"
    ]
  ],
  [
    [
      "HP:0000737",
      "HP:0000842",
      "HP:0001197",
      "HP:0001522",
      "HP:0001873",
      "HP:0001892",
      "HP:0001943",
      "HP:0001987",
      "HP:0003155",
      "HP:0003161",
      "HP:0003231",
      "HP:0003235",
      "HP:0003645",
      "HP:0003811",
      "HP:0003819",
      "HP:0004923",
      "HP:0006254",
      "HP:0010472",
      "HP:0012117"
    ],
    [
      "CCRD:115.1",
      "OMIM:276700",
      "ORPHA:882"
    ]
  ],
  [
    [
      "HP:0001197",
      "HP:0001522",
      "HP:0001987",
      "HP:0003811",
      "HP:0003819",
      "HP:0011966"
    ],
    [
      "CCRD:18",
      "OMIM:605814",
      "ORPHA:187",
      "ORPHA:247598"
    ]
  ],
  [
    [
      "HP:0001596",
      "HP:0002013",
      "HP:0002014",
```

"HP:0004923",
"HP:0100613"
],
[
"CCRD:90",
"OMIM:261600",
"ORPHA:716"
]
],
[
[
"HP:0003215",
"HP:0003344",
"HP:0040156"
],
[
"OMIM:246450",
"ORPHA:20"
]
],
[
[
"HP:0000976",
"HP:0000988",
"HP:0001522",
"HP:0001596",
"HP:0002151",
"HP:0002490"
],
[
"CCRD:13",
"OMIM:253260",
"ORPHA:79241"
]
],
[
[
"HP:0001254",
"HP:0001522",
"HP:0001873",
"HP:0001882",
"HP:0001943",
"HP:0001944",
"HP:0001945",
"HP:0001987",
"HP:0002027",
"HP:0002045",
"HP:0002090",
"HP:0002154",
"HP:0002329",
"HP:0002360",
"HP:0003348",
"HP:0003811",
"HP:0003819",
"HP:0004429",
"HP:0005561",

```
        "HP:0010910",
        "HP:0010913",
        "HP:0010967",
        "HP:0011421",
        "HP:0011968",
        "HP:0012378",
        "HP:0500001"
      ],
      [
        "CCRD:58",
        "OMIM:243500",
        "ORPHA:33"
      ]
    ],
    [
      [
        "HP:0000961",
        "HP:0001250",
        "HP:0001522",
        "HP:0001987",
        "HP:0002181",
        "HP:0002380",
        "HP:0003218",
        "HP:0003572",
        "HP:0003811",
        "HP:0005961"
      ],
      [
        "OMIM:237300",
        "ORPHA:147"
      ]
    ],
    [
      [
        "HP:0001061",
        "HP:0001197",
        "HP:0001522",
        "HP:0003231",
        "HP:0003811",
        "HP:0004923",
        "HP:0011421"
      ],
      [
        "CCRD:90",
        "OMIM:261600",
        "ORPHA:716"
      ]
    ],
    [
      [
        "HP:0000486",
        "HP:0001249",
        "HP:0001251",
        "HP:0001270",
        "HP:0001272",
        "HP:0001290",
```

```
      "HP:0003819",
      "HP:0012642"
    ],
    [
      "OMIM:212065",
      "ORPHA:79318"
    ]
  ],
  [
    [
      "HP:0001638",
      "HP:0001945",
      "HP:0003110",
      "HP:0003819",
      "HP:0004386",
      "HP:0008315"
    ],
    [
      "CCRD:15",
      "OMIM:212140",
      "ORPHA:158"
    ]
  ],
  [
    [
      "HP:0000252",
      "HP:0000324",
      "HP:0000473",
      "HP:0000608",
      "HP:0000750",
      "HP:0000752",
      "HP:0001250",
      "HP:0001251",
      "HP:0001264",
      "HP:0001522",
      "HP:0002014",
      "HP:0002059",
      "HP:0002090",
      "HP:0002179",
      "HP:0002305",
      "HP:0002510",
      "HP:0002538",
      "HP:0003811",
      "HP:0003819",
      "HP:0100660"
    ],
    [
      "OMIM:256730",
      "ORPHA:228329"
    ]
  ],
  [
    [
      "HP:0001987",
      "HP:0002013",
      "HP:0003217",
```

```
      "HP:0003218",
      "HP:0003819"
    ],
    [
      "CCRD:85",
      "OMIM:311250",
      "ORPHA:664"
    ]
  ],
  [
    [
      "HP:0000729",
      "HP:0000822",
      "HP:0000969",
      "HP:0001249",
      "HP:0001250",
      "HP:0001259",
      "HP:0001290",
      "HP:0001522",
      "HP:0001733",
      "HP:0001882",
      "HP:0001903",
      "HP:0001919",
      "HP:0001987",
      "HP:0001997",
      "HP:0002013",
      "HP:0002149",
      "HP:0002151",
      "HP:0002154",
      "HP:0002312",
      "HP:0002370",
      "HP:0002751",
      "HP:0002902",
      "HP:0002912",
      "HP:0003075",
      "HP:0003097",
      "HP:0003138",
      "HP:0003155",
      "HP:0003210",
      "HP:0003259",
      "HP:0003281",
      "HP:0003819",
      "HP:0010472",
      "HP:0010899",
      "HP:0010914",
      "HP:0010916",
      "HP:0010967",
      "HP:0011421",
      "HP:0012120",
      "HP:0012239",
      "HP:0012622",
      "HP:0100613"
    ],
    [
      "CCRD:71",
      "OMIM:251110",
```

```
      "ORPHA:79311"
    ]
  ],
  [
    [
      "HP:0001249",
      "HP:0001270",
      "HP:0001290",
      "HP:0002013",
      "HP:0003217",
      "HP:0003819",
      "HP:0004429",
      "HP:0010911",
      "HP:0011968",
      "HP:0012120"
    ],
    [
      "CCRD:71",
      "OMIM:251000",
      "ORPHA:27"
    ]
  ],
  [
    [
      "HP:0001254",
      "HP:0001290",
      "HP:0001522",
      "HP:0001987",
      "HP:0002329",
      "HP:0002360",
      "HP:0003811",
      "HP:0011968",
      "HP:0500001"
    ],
    [
      "CCRD:67",
      "OMIM:248600",
      "ORPHA:511"
    ]
  ],
  [
    [
      "HP:0001259",
      "HP:0001265",
      "HP:0001290",
      "HP:0001522",
      "HP:0001649",
      "HP:0001942",
      "HP:0001987",
      "HP:0002098",
      "HP:0002181",
      "HP:0002380",
      "HP:0003573",
      "HP:0003645",
      "HP:0003811",
      "HP:0010899",
```

```
        "HP:0010903",
        "HP:0011965"
      ],
      [
        "CCRD:18",
        "OMIM:215700",
        "ORPHA:187",
        "ORPHA:247525"
      ]
    ],
    [
      [
        "HP:0000708",
        "HP:0000752",
        "HP:0001249",
        "HP:0001259",
        "HP:0001270",
        "HP:0001399",
        "HP:0001824",
        "HP:0001945",
        "HP:0001987",
        "HP:0002013",
        "HP:0002120",
        "HP:0002355",
        "HP:0003217",
        "HP:0003218",
        "HP:0003551",
        "HP:0003819",
        "HP:0009046",
        "HP:0010899",
        "HP:0010916",
        "HP:0011421"
      ],
      [
        "CCRD:85",
        "OMIM:311250",
        "ORPHA:664"
      ]
    ],
    [
      [
        "HP:0000316",
        "HP:0000369",
        "HP:0000431",
        "HP:0001197",
        "HP:0001249",
        "HP:0001270",
        "HP:0001522",
        "HP:0003781",
        "HP:0003811",
        "HP:0012071",
        "HP:0012714",
        "HP:0040081"
      ],
      [
        "OMIM:255120",
```

```
      "ORPHA:156"
    ]
  ],
  [
    [
      "HP:0003131",
      "HP:0003268",
      "HP:0010909",
      "HP:0011965",
      "HP:0012127"
    ],
    [
      "CCRD:6",
      "OMIM:207800",
      "ORPHA:90"
    ]
  ],
  [
    [
      "HP:0001942",
      "HP:0003210",
      "HP:0003819"
    ],
    [
      "CCRD:71",
      "OMIM:251000",
      "ORPHA:27"
    ]
  ],
  [
    [
      "HP:0000961",
      "HP:0001250",
      "HP:0001522",
      "HP:0001987",
      "HP:0003811",
      "HP:0008160"
    ],
    [
      "CCRD:35.1",
      "OMIM:232200",
      "ORPHA:79258"
    ]
  ],
  [
    [
      "HP:0001290",
      "HP:0001522",
      "HP:0002910",
      "HP:0003155",
      "HP:0003573",
      "HP:0003645",
      "HP:0003811",
      "HP:0004342",
      "HP:0010899",
      "HP:0010916",
```

```
      "HP:0012024",
      "HP:0012343",
      "HP:0200119",
      "HP:0200123"
    ],
    [
      "CCRD:30",
      "OMIM:230400",
      "ORPHA:352",
      "ORPHA:79239"
    ]
  ],
  [
    [
      "HP:0001254",
      "HP:0001290",
      "HP:0001522",
      "HP:0002093",
      "HP:0002151",
      "HP:0002329",
      "HP:0002360",
      "HP:0003215",
      "HP:0003688",
      "HP:0003811",
      "HP:0040156"
    ],
    [
      "OMIM:220110",
      "ORPHA:254905"
    ]
  ],
  [
    [
      "HP:0001254",
      "HP:0001290",
      "HP:0001522",
      "HP:0001943",
      "HP:0001987",
      "HP:0002013",
      "HP:0002329",
      "HP:0002360",
      "HP:0003819",
      "HP:0008315"
    ],
    [
      "CCRD:70",
      "OMIM:201450",
      "ORPHA:42"
    ]
  ],
  [
    [
      "HP:0001290",
      "HP:0001510",
      "HP:0001522",
      "HP:0010628",
```

```
      "HP:0040319"
    ],
    [
      "OMIM:203500",
      "ORPHA:56"
    ]
  ],
  [
    [
      "HP:0001513",
      "HP:0002027",
      "HP:0003819"
    ],
    [
      "CCRD:92",
      "OMIM:176000",
      "ORPHA:738",
      "ORPHA:79276"
    ]
  ],
  [
    [
      "HP:0001522",
      "HP:0003811",
      "HP:0004923"
    ],
    [
      "CCRD:90",
      "OMIM:261600",
      "ORPHA:716"
    ]
  ],
  [
    [
      "HP:0001290",
      "HP:0001522",
      "HP:0002013",
      "HP:0003215",
      "HP:0003355",
      "HP:0003811",
      "HP:0004357",
      "HP:0010912",
      "HP:0040156"
    ],
    [
      "CCRD:67",
      "OMIM:248600",
      "ORPHA:511"
    ]
  ],
  [
    [
      "HP:0001197",
      "HP:0001518",
      "HP:0001522",
      "HP:0003348",
```

```
      "HP:0003811",
      "HP:0003819",
      "HP:0010967",
      "HP:0012714"
    ],
    [
      "CCRD:70",
      "OMIM:201450",
      "ORPHA:42"
    ]
  ],
  [
    [
      "HP:0000256",
      "HP:0000476",
      "HP:0001254",
      "HP:0001290",
      "HP:0001332",
      "HP:0001522",
      "HP:0001945",
      "HP:0002013",
      "HP:0002014",
      "HP:0002094",
      "HP:0002329",
      "HP:0002360",
      "HP:0003150",
      "HP:0003811",
      "HP:0008315",
      "HP:0100660"
    ],
    [
      "CCRD:34.1",
      "OMIM:231670",
      "ORPHA:25"
    ]
  ],
  [
    [
      "HP:0000729",
      "HP:0000742",
      "HP:0000752",
      "HP:0001249",
      "HP:0001250",
      "HP:0001270",
      "HP:0001513",
      "HP:0001522",
      "HP:0003155",
      "HP:0003231",
      "HP:0003811",
      "HP:0003819",
      "HP:0004923",
      "HP:0006254",
      "HP:0010899",
      "HP:0010916",
      "HP:0040303"
    ],
```

    [
      "CCRD:115.1",
      "OMIM:276700",
      "ORPHA:882"
    ]
  ],
  [
    [
      "HP:0001522",
      "HP:0001622",
      "HP:0002155",
      "HP:0003811",
      "HP:0003819",
      "HP:0010967"
    ],
    [
      "CCRD:70",
      "OMIM:201450",
      "ORPHA:42"
    ]
  ],
  [
    [
      "HP:0000752",
      "HP:0001522",
      "HP:0010899",
      "HP:0011968",
      "HP:0012447"
    ],
    [
      "OMIM:271900",
      "ORPHA:141"
    ]
  ],
  [
    [
      "HP:0000508",
      "HP:0000750",
      "HP:0001159",
      "HP:0001249",
      "HP:0001270",
      "HP:0001290",
      "HP:0001522",
      "HP:0001760",
      "HP:0002705",
      "HP:0003462",
      "HP:0003819",
      "HP:0010569",
      "HP:0011421"
    ],
    [
      "OMIM:270400",
      "ORPHA:818"
    ]
  ],
  [

    [
      "HP:0000100",
      "HP:0001337",
      "HP:0003819",
      "HP:0040144"
    ],
    [
      "OMIM:236792",
      "ORPHA:79314"
    ]
  ],
  [
    [
      "HP:0001250",
      "HP:0001290",
      "HP:0001522",
      "HP:0001942",
      "HP:0003811",
      "HP:0012120"
    ],
    [
      "CCRD:71",
      "OMIM:251000",
      "ORPHA:27"
    ]
  ],
  [
    [
      "HP:0000976",
      "HP:0000988",
      "HP:0001259",
      "HP:0001290",
      "HP:0001522",
      "HP:0001943",
      "HP:0001945",
      "HP:0001987",
      "HP:0002013",
      "HP:0002181",
      "HP:0002305",
      "HP:0003217",
      "HP:0003218",
      "HP:0003355",
      "HP:0003811",
      "HP:0003819",
      "HP:0004386",
      "HP:0004429",
      "HP:0010907",
      "HP:0010909",
      "HP:0011421",
      "HP:0011966",
      "HP:0012127",
      "HP:0012819",
      "HP:0040081"
    ],
    [
      "CCRD:85",

```
      "OMIM:311250",
      "ORPHA:664"
    ]
  ],
  [
    [
      "HP:0003355",
      "HP:0010895",
      "HP:0010967",
      "HP:0040156"
    ],
    [
      "OMIM:210200",
      "ORPHA:6"
    ]
  ],
  [
    [
      "HP:0000750",
      "HP:0001522",
      "HP:0002013",
      "HP:0002312",
      "HP:0002370",
      "HP:0003811",
      "HP:0003819",
      "HP:0040301"
    ],
    [
      "OMIM:307030",
      "ORPHA:408"
    ]
  ],
  [
    [
      "HP:0000518",
      "HP:0001385",
      "HP:0001518",
      "HP:0001522",
      "HP:0001622",
      "HP:0001903",
      "HP:0001987",
      "HP:0003259",
      "HP:0003811",
      "HP:0003819",
      "HP:0010967",
      "HP:0012120",
      "HP:0100613"
    ],
    [
      "CCRD:71",
      "OMIM:251000",
      "ORPHA:27"
    ]
  ],
  [
    [
```

```
      "HP:0000750",
      "HP:0001197",
      "HP:0001522",
      "HP:0003231",
      "HP:0003811",
      "HP:0003819",
      "HP:0004923",
      "HP:0100613"
    ],
    [
      "CCRD:90",
      "OMIM:261600",
      "ORPHA:716"
    ]
  ],
  [
    [
      "HP:0001197",
      "HP:0001522",
      "HP:0003811",
      "HP:0003819",
      "HP:0004386",
      "HP:0008315"
    ],
    [
      "CCRD:70",
      "OMIM:201450",
      "ORPHA:42"
    ]
  ],
  [
    [
      "HP:0000750",
      "HP:0001249",
      "HP:0001270",
      "HP:0001522",
      "HP:0003231",
      "HP:0003819",
      "HP:0004923",
      "HP:0010472",
      "HP:0011421"
    ],
    [
      "CCRD:90",
      "OMIM:261600",
      "ORPHA:716"
    ]
  ],
  [
    [
      "HP:0001197",
      "HP:0001522",
      "HP:0002160",
      "HP:0003235",
      "HP:0003355",
      "HP:0003811",
```

```
      "HP:0003819"
    ],
    [
      "CCRD:45",
      "OMIM:236200",
      "ORPHA:394"
    ]
  ],
  [
    [
      "HP:0000939",
      "HP:0001290",
      "HP:0001513",
      "HP:0001760",
      "HP:0001882",
      "HP:0001987",
      "HP:0002154",
      "HP:0002312",
      "HP:0002370",
      "HP:0003348",
      "HP:0003510",
      "HP:0003819",
      "HP:0004386",
      "HP:0010472",
      "HP:0010899",
      "HP:0010911",
      "HP:0010967",
      "HP:0011421",
      "HP:0012714",
      "HP:0100613"
    ],
    [
      "CCRD:99",
      "OMIM:606054",
      "ORPHA:35"
    ]
  ],
  [
    [
      "HP:0003231",
      "HP:0003819",
      "HP:0004923"
    ],
    [
      "CCRD:90",
      "OMIM:261600",
      "ORPHA:716"
    ]
  ],
  [
    [
      "HP:0000077",
      "HP:0000822",
      "HP:0001250",
      "HP:0001259",
      "HP:0001522",
```

```
      "HP:0001638",
      "HP:0001873",
      "HP:0001882",
      "HP:0001903",
      "HP:0001974",
      "HP:0001987",
      "HP:0002013",
      "HP:0002149",
      "HP:0002151",
      "HP:0002152",
      "HP:0002153",
      "HP:0002154",
      "HP:0002155",
      "HP:0002652",
      "HP:0002789",
      "HP:0002912",
      "HP:0003075",
      "HP:0003124",
      "HP:0003138",
      "HP:0003155",
      "HP:0003165",
      "HP:0003210",
      "HP:0003228",
      "HP:0003235",
      "HP:0003259",
      "HP:0003348",
      "HP:0003510",
      "HP:0003811",
      "HP:0003819",
      "HP:0004421",
      "HP:0005117",
      "HP:0010472",
      "HP:0010906",
      "HP:0010910",
      "HP:0010967",
      "HP:0011421",
      "HP:0011966",
      "HP:0011968",
      "HP:0012026",
      "HP:0012120",
      "HP:0100613"
    ],
    [
      "CCRD:71",
      "OMIM:251000",
      "ORPHA:27"
    ]
  ],
  [
    [
      "HP:0000750",
      "HP:0000752",
      "HP:0001250",
      "HP:0001290",
      "HP:0001522",
      "HP:0002355",
```

```
      "HP:0003551",
      "HP:0003819",
      "HP:0009046",
      "HP:0040156"
    ],
    [
      "OMIM:271980",
      "ORPHA:22"
    ]
  ],
  [
    [
      "HP:0001250",
      "HP:0001332",
      "HP:0001522",
      "HP:0003150",
      "HP:0010551",
      "HP:0011968"
    ],
    [
      "CCRD:34.1",
      "OMIM:231670",
      "ORPHA:25"
    ]
  ],
  [
    [
      "HP:0001197",
      "HP:0001518",
      "HP:0001522",
      "HP:0001987",
      "HP:0003355",
      "HP:0003811",
      "HP:0003819",
      "HP:0008315",
      "HP:0010895",
      "HP:0040156"
    ],
    [
      "OMIM:210200",
      "ORPHA:6"
    ]
  ],
  [
    [
      "HP:0000488",
      "HP:0000518",
      "HP:0000822",
      "HP:0001635",
      "HP:0001658",
      "HP:0001919",
      "HP:0002344",
      "HP:0003076",
      "HP:0012622",
      "HP:0040217",
      "HP:0100613",
```

```
    "HP:0100806"
  ],
  [
    "OMIM:125850",
    "ORPHA:552"
  ]
],
[
  [
    "HP:0001197",
    "HP:0001522",
    "HP:0003231",
    "HP:0003811",
    "HP:0004923",
    "HP:0011421",
    "HP:0040156"
  ],
  [
    "CCRD:90",
    "OMIM:261600",
    "ORPHA:716"
  ]
],
[
  [
    "HP:0000750",
    "HP:0001249",
    "HP:0001270",
    "HP:0001399",
    "HP:0001522",
    "HP:0001622",
    "HP:0002014",
    "HP:0003573",
    "HP:0003645",
    "HP:0003811",
    "HP:0003819",
    "HP:0004342",
    "HP:0010899",
    "HP:0010916",
    "HP:0012024"
  ],
  [
    "CCRD:30",
    "OMIM:230400",
    "ORPHA:352",
    "ORPHA:79239"
  ]
],
[
  [
    "HP:0001197",
    "HP:0001522",
    "HP:0003811",
    "HP:0003819",
    "HP:0004386",
    "HP:0004923"
```

```
    ],
    [
      "CCRD:90",
      "OMIM:261600",
      "ORPHA:716"
    ]
  ],
  [
    [
      "HP:0000047",
      "HP:0001197",
      "HP:0001522",
      "HP:0002032",
      "HP:0002781",
      "HP:0003231",
      "HP:0003811",
      "HP:0003819",
      "HP:0004923",
      "HP:0006541",
      "HP:0011421",
      "HP:0100613"
    ],
    [
      "CCRD:90",
      "OMIM:261600",
      "ORPHA:716"
    ]
  ],
  [
    [
      "HP:0001197",
      "HP:0001522",
      "HP:0003231",
      "HP:0003811",
      "HP:0004923",
      "HP:0011421"
    ],
    [
      "CCRD:90",
      "OMIM:261600",
      "ORPHA:716"
    ]
  ]
```
